# Reactivity of 2*H*‑Azirines in Copper-Catalyzed Azide–Alkyne Cycloaddition Reactions

**DOI:** 10.1021/acs.orglett.5c03150

**Published:** 2025-09-01

**Authors:** Lukáš Janecký, Andrea Madabeni, Eliška Jelínková, Blanka Klepetářová, Lubomír Rulíšek, Petr Beier

**Affiliations:** † Institute of Organic Chemistry and Biochemistry, 48311 Academy of Sciences of the Czech Republic, Flemingovo nám. 2, 166 10 Prague, Czech Republic; ‡ Department of Organic Chemistry, Faculty of Science, Charles University, Hlavova 2030/8, CZ-128 43 Prague 2, Czech Republic

## Abstract

A reaction between azide, alkyne and 2*H*-azirine resulted in C–C bond formation at position five of 1,2,3-triazole, instead of previously misidentified C–N bond connectivity. The reaction mechanism of this C–C bond formation on the triazole ring was fully explained by employing calibrated QM­(DFT-D3) calculations. Functionalization of primary products provided substituted pyrimidine, furan or 1,3-oxazepine.

## Graphical abstract

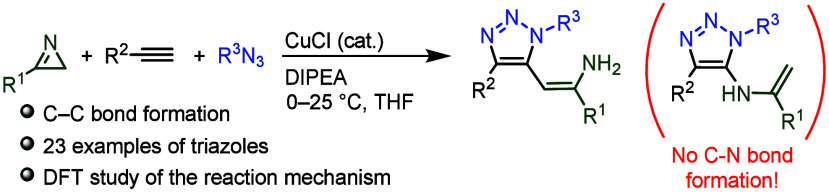

Copper­(I)-catalyzed azide–alkyne cycloaddition (CuAAC) is a widely recognized and robust method for the synthesis of 1,4-disubstituted 1,2,3-triazoles.^1^ Its three-component variant, involving an additional electrophile, also known as the interrupted click reaction, has undergone significant development in the past decade. Depending on the choice of electrophilic reagent, position five on the formed triazole ring can be modified with halogen atoms,^2–4^ nitrogen,^5^ sulfur,^6^ phosphorus,^7^ tin,^8^ or selenium moieties.^9^ Through this approach, it is also possible to attach carbon-containing functionalities in position five, for example allyl,^10^ perfluoroalkyl,^11^ benzyl,^12^ aryl,^13^ allenyl,^14^ acyl,^15,16^ vinyl,^17^ or alkynyl (Scheme 1).^18^

**Scheme 1:**
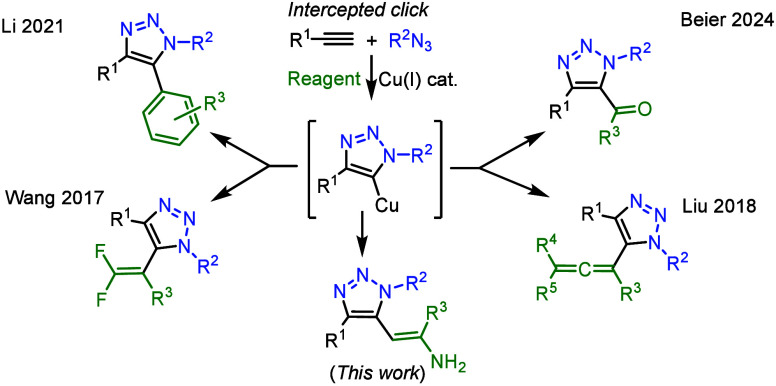
Examples of Interrupted Click Reactions

In 2017, Chen and co-workers reported the preparation of 5-enamine-functionalized 1,2,3-triazoles via interrupted click reaction of alkynes, azides and 2*H*-azirines and proposed that the reaction proceeded through Cu-catalyzed vinyl nitrene transfer (Scheme 2).^19^ In their work, an X-ray structure of a compound denoted as “**4a**” was published. In a subsequent study, Zhao and co-workers suggested, based on the DFT calculations, a reaction mechanism for the formation of the C–N bond during an interrupted click reaction of 2*H*-azirines, alkynes and azides.^20^ The first discrepancy was found in the ^1^H NMR characterization of the supposed terminal double bond of “**4a**” (3.55 ppm, s, 2H).^19^ We expected that the signals for the *cis* and *trans* hydrogen atoms should have different chemical shifts. Moreover, the checkCIF validation report of structure “**4a**” contains three B-alerts of type 2, all related to the Hirshfeld test. This may indicate a wrong scattering type assignment. The atoms involved are N4, C2, C3 and C4 (Scheme 2). Closer inspection of the thermal ellipsoids of these atoms revealed an unusual shape and size of the ellipsoids of atoms N4 and C4, which suggests that these atoms may have been assigned incorrectly. Using original diffraction data, we resolved the structure while changing the N4 atom to carbon and the C4 atom to nitrogen. The structure refined to R = 0.0550 and the checkCIF validation report no longer contains the B-alerts. Therefore, we propose a new structure with different connectivity (Scheme 2). To obtain additional evidence for the correct structure of “**4a**,” we synthesized it according to the original protocol by Chen^19^ and performed ^13^C APT NMR spectroscopy, which clearly showed a CH= carbon (81.86 ppm) instead of a CH_2_= carbon. With the correct characterization of “**4a**” as **4s**, the reaction mechanism for the transformation of triazoles to pyrazoles can now be easily understood (Scheme 3).

**Scheme 2:**
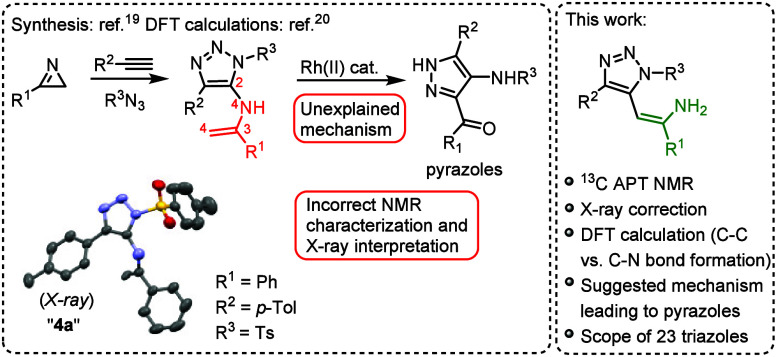
Preparation of Enamine-Functionalized 1,2,3-Triazoles and Their Application Leading to Pyrazoles Reported by Chen^19^

**Scheme 3:**
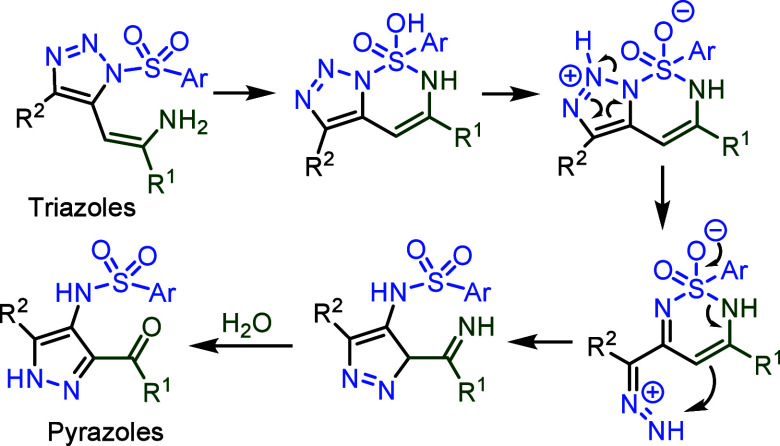
Proposed Mechanism of the Transformation of Enamine-Functionalized 1,2,3-Triazoles to Pyrazoles Reported by Chen.^19^

In an article related to Chen’s work, Zheng and Song published in 2022 a four-component interrupted click reaction of 2*H*-azirines with alkynes, azides and amines, described as Cu-catalyzed one-step formation of four C–N bonds toward polyfunctionalized triazoles.^21^ We prepared the compound denoted as “**5ad**” according to the described procedure.^21^ Once again, ^13^C APT NMR spectroscopy confirmed our hypothesis (see the characterization of compound **4r** in the SI) that triazoles reported by Zheng and Song were characterized incorrectly (Scheme 4).

**Scheme 4:**
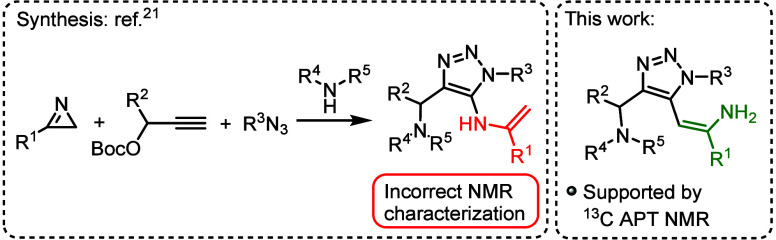
Preparation of 5-Enamine-Functionalized 1,2,3-Triazoles by Zheng and Song^21^

We expanded the scope of interrupted click reactions of 2*H*-azirines (**1**), alkynes (**2**) and azides (**3**), focusing not only on benzyl azide and tosyl azide, but also on organic fluorinated azides developed in our laboratory,^22–28^ toward a new series of triazoles **4**. The combination of 2*H*-azirine (1.0 equiv), alkyne (1.3 equiv), pentafluoroethyl azide (1.5 equiv), DIPEA (2.2 equiv), CuCl (10 mol %) and molecular sieves in THF proved to be the most effective combination for the preparation of triazole **4a** in 61% yield (See the SI for the optimization).

Having established the optimized reaction conditions, we investigated the substrate scope for 5-enamine-functionalized 1,2,3-triazoles (Scheme 5). Triazoles **4a**–**g** with electron-neutral, -donating, or -withdrawing functional groups located in *para*-position of the aromatic ring, were prepared in moderate to good yields. To demonstrate the scalability of the reaction, triazole **4a** was prepared on 1.29 g (6.54 mmol) scale. 4-Nitrophenyl acetylene as well as 2-methoxyphenylacetylene and 2-(trifluoromethyl)­phenylacetylene afforded only traces of the corresponding triazoles. Triazole **4h** with two methoxy groups in *meta*-positions, as well as heteroaryl-, alkenyl- or alkyl-substituted triazoles (**4i**–**l**) were also prepared in good to excellent yields. Triazoles **4m**–**o** were prepared from different fluoroalkylated azides in very good yields. In the case of N_3_CF_2_CO_2_Et, the 7-membered ring-fused-triazole **4p** was formed by the interrupted click reaction, followed by lactam formation. Azide N_3_CF_2_SO_2_Ph behaved in an unusual way. Triazolopyrimidine **4q** was isolated in a good yield by a process understood only after we looked at the stability of products **4**. The correct structures of **4r** and **4s** are discussed above. Various 2*H*-azirines that were utilized for the modification of position five on triazoles afforded **4t**–**w** in good yields.

**Scheme 5:**
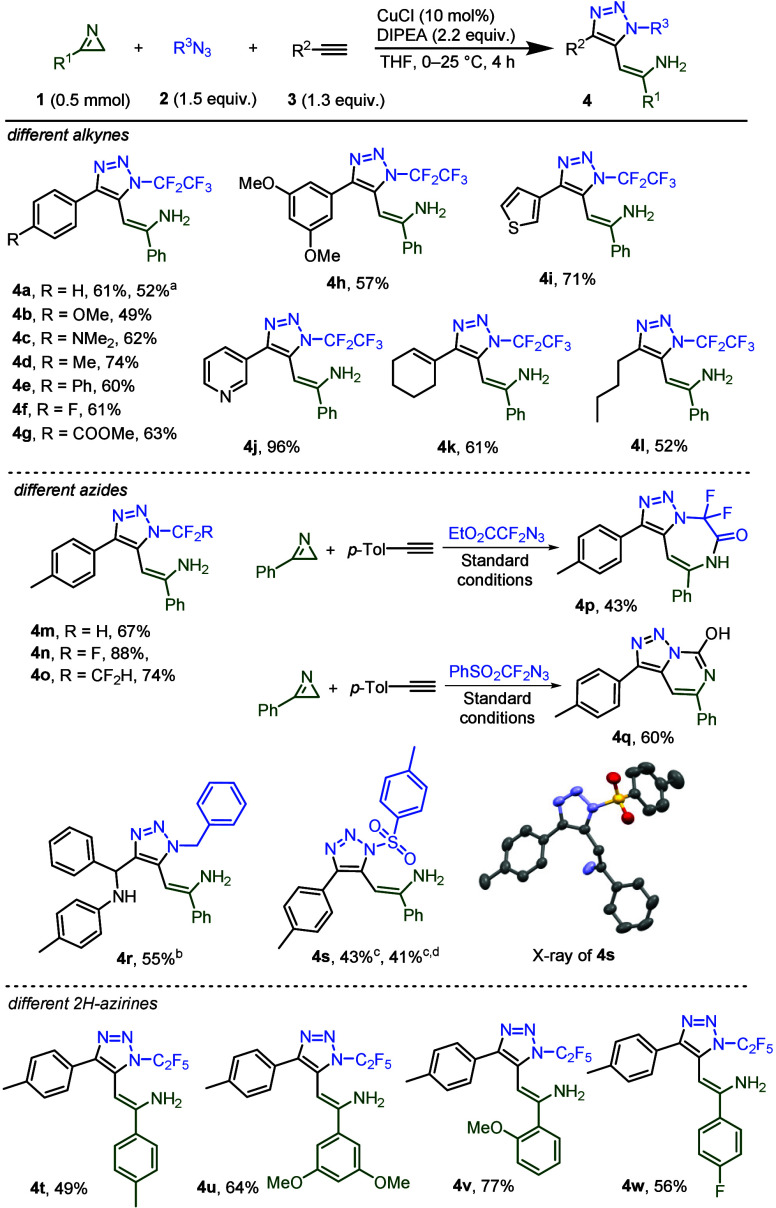
Substrate Scope of the Interrupted Click Reaction to Triazoles **4**

a 6.54 mmol scale.
b Prepared according to ref 21.
c Prepared according to ref 19.
d 12.8 mmol scale.

Triazoles **4** were found to be unstable during long-term storage in solution (however, they were stable neat in a freezer). For example, when triazoles **4d** or **4j** were left in the crude reaction mixture at room temperature they first transformed to triazolopyrimidines^29^ **5** (crystal structure of **5j** was obtained). However, these compounds are acid sensitive and finally formed pyrimidines **6** (**6a** was isolated). The proposed mechanism of this transformation is presented in Scheme 6 and involves HF elimination during cyclization to **5**, followed by triazole denitrogenation to **6**. A similar process can explain the formation of compound **4q** (Scheme 7).

**Scheme 6:**
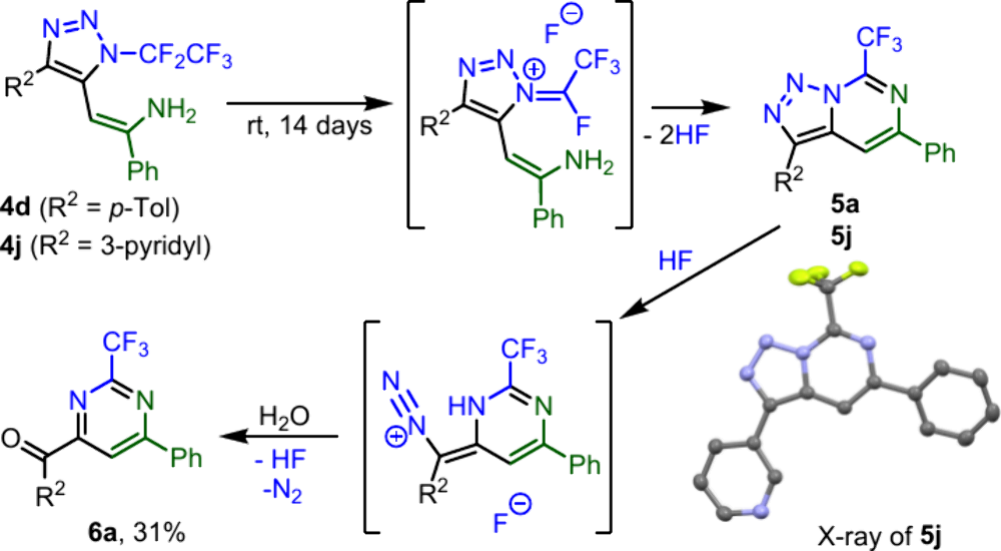
Decomposition of **4** to **5** and **6**

**Scheme 7:**
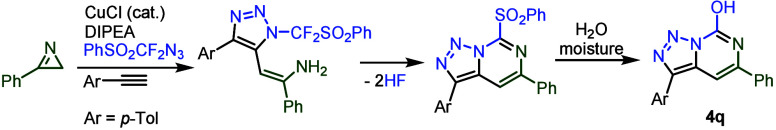
Formation of Compound **4q**

Strongly acidic hydrolysis of triazoles **4m** and **4u** afforded triazoles **7m** and **7u** (Scheme 8). Compound **7u** was subjected to further transformations of the triazole ring. A subsequent reaction with a Lewis acid afforded, after triazole denitrogenation, cyclization and hydrolysis, functionalized furan **8**. By contrast, microwave heating in the presence of a fluoride source led to denitrogenation, rearrangement to ketenimine, 1,3-fluoride shift and cyclization to 4-fluoro-1,3-oxazepine **9** (Scheme 8).

**Scheme 8:**
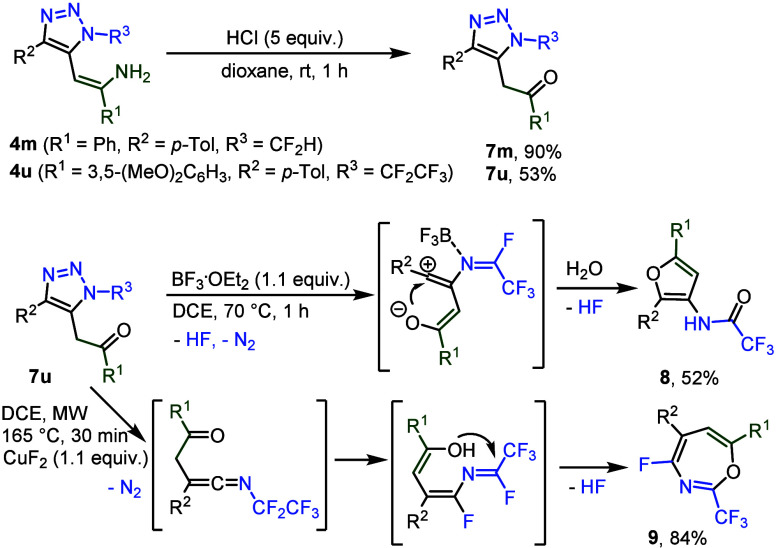
Postfunctionalization of triazoles **4** and **7u**

To provide a sound mechanistic comparison between the formation of the C–C bond versus the previously reported C–N adduct, mechanistic DFT investigations were carried out. Among the various models studied (see the SI for details), the most energetically accessible reaction pathways were obtained for the model involving two copper atoms (Figure 1). Species **R** attacks *2H*-azirine and breaks the C–N bond via **TS0**, producing a 4-membered anionic metalacyclic intermediate (**I1**). Compound **I1** is easily protonated by a proton source (Me_3_NH^+^ in our model) to produce a stable neutral metallacyclic intermediate (**I1** ^**+**^). Note that, in **TS0**, **I1** and **I1** ^**+**^, C–N coupling would proceed via similar structures where the CH_2_ and N exchange place in the roughly square planar copper complexes. (Figure 5 in the SI) Along the C–C coupling pathway, the triazolate and the C atom of *2H*-azirine are in *cis* position to each other, whereas for C–N coupling pathway they would take up *trans* position. The different stereoisomeric nature of these species leads to energetics which always favor the C–C coupling stationary points, regardless of the substituent on the triazolate ligand. Lastly, from **I1** ^**+**^, the C–C coupling occurs through a 5-membered ring transition state (**TS1**) in which the C–C bond is forming, while the C–Cu bond is breaking. The C–C bond formation proceeds with a lower activation energy, which at our level of theory is even lower than the one required for the formation of **I1** ^**+**^. Compound **I2** can finally evolve into the observed product via a tautomeric equilibrium. The final enamine product (**4a**) was found to be 3.9 kcal mol^–1^ more stable than the tautomeric imine. Similar results were obtained for an analogous reactant with the tosyl group instead of the pentafluoroethyl group.

**Figure 1:**
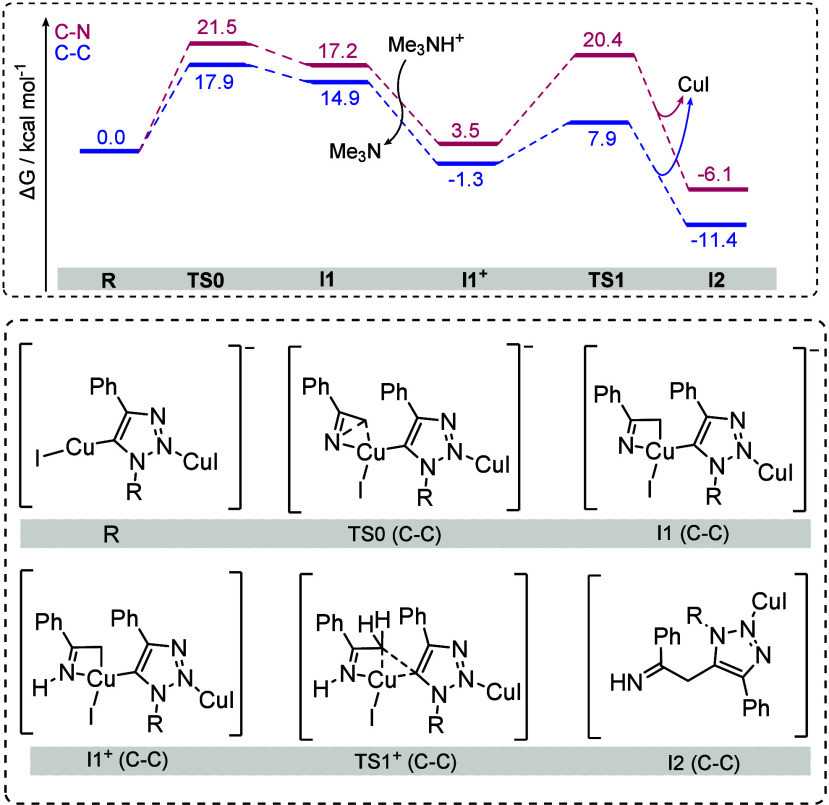
Computed reaction mechanism (COSMO-B3LYP-D3­(BJ)/def2-TZVP//COSMO-TPSS-D3­(BJ)/def2-SVP) for C–N and C–C couplings (R = CF_2_CF_3_).

In summary, we have described the reactivity of 2*H*-azirines in an interrupted click reaction with alkynes and azides by reinvestigating the structures **4r** and **4s**, which were previously published with incorrect atom connectivity. DFT calculations support the evidence that the C–C coupling should be favored over the C–N coupling, regardless of the substituent on the triazolate nitrogen. Additionally, we have expanded the scope of interrupted click reaction of 2*H*-azirines with fluorinated azides, leading to triazoles **4**, and applied these triazoles in the synthesis of 5-homoacyl triazoles, pyrimidines, furan and oxazepine.

## Supplementary Material



## Data Availability

The data underlying this study are available in the published article and its Supporting Information.

## Notes

Computational details (DFT), experimental part, NMR data and copies of NMR spectra, and crystal structures of **4s** (CCDC 1469919) and **5j** (CCDC 2428749) (PDF)
