# Supplementary material for: Reactivity of 2H‑Azirines in Copper-Catalyzed Azide–Alkyne Cycloaddition Reactions
Source: Org Lett. 2025 Sep 1;27(36):10077–81. doi: 10.1021/acs.orglett.5c03150 (PMC12442078; doi:10.1021/acs.orglett.5c03150)
Supplement: Supplementary file 1 [file ol5c03150_si_001.pdf]

Reactivity of 2H-Azirines in Copper-Catalyzed Azide-Alkyne Cycloaddition Reactions

Lukáš Janecký,[a,b] Andrea Madabeni,[a] Eliška Jelínková,[a] Blanka Klepetářová,[a] Lubomír Rulíšek[a] and Petr Beier[a,*]

[a]Institute of Organic Chemistry and Biochemistry, Academy of Sciences of the Czech Republic, Flemingovo nám. 2, 166 10 Prague, Czech Republic
[b]Department of Organic Chemistry, Faculty of Science, Charles University, Hlavova 2030/8, 128 43 Prague 2, Czech Republic

*Corresponding author: beier@uochb.cas.cz

Supporting information

Contents

General information S2

Computational details S2

X-ray crystallography S45

Preparation and characterization of 2*H*-azirines **1** S47

Preparation and characterization of vinyl azides **2** S48

Preparation and characterization of alkyne **3** S49

Optimization of the reaction conditions leading to triazoles **4** S51

Preparation and characterization of triazoles **4** S53

Preparation and characterization of heterocycles **6**, **7**, **8**, **9** S60

Copies of NMR spectra S63

References S169

General information

All commercially available chemicals were used as received unless stated otherwise. Flash column chromatography was performed using silica gel 60 (0.040–0.063 mm). Automated flash column chromatography was performed on Teledyne ISCO CombiFlash Rf⁺ Lumen Automated Flash Chromatography System with UV/Vis detection. ^{1}H, ^{13}C, and ^{19}F NMR spectra were measured at ambient temperature using 5 mm diameter NMR tubes. ^{13}C NMR spectra were proton decoupled. The chemical shift values (δ) are reported in ppm relative to internal Me_4Si (0 ppm for ^{1}H and ^{13}C NMR) or residual solvents and internal $CFCl_3$ (0 ppm for ^{19}F NMR). Coupling constants (J) are reported in Hertz. Structural elucidation was aided by additional acquisition of various 2D NMR spectra (^{1}H-^{1}H COSY, ^{1}H-^{13}C HSQC, ^{1}H-^{13}C HMBC). High resolution mass spectra (HRMS) were recorded on a Waters Micromass AutoSpec Ultima or Agilent 7890A GC coupled with Waters GCT Premier orthogonal acceleration time-of-flight detector using electron impact (EI) or chemical ionization (CI), on an LTQ Orbitrap XL using electrospray ionization (ESI), Q-Tof micro (Waters) is a quadrupole orthogonal acceleration time-of-flight tandem mass spectrometer using atmospheric-pressure chemical ionization (APCI), and on a Bruker solariX 94 ESI/MALDI-FT-ICR using dual ESI/MALDI ionization. Microwave experiments were done on CEM Focused Microwave™ Synthesis System, Model Discover (300 W). The temperature monitoring was provided by non-contact IR (iWave / floor-mounted IR): a sensor under the cavity floor reads the temperature at the bottom of the vial (volume-independent), and that reading was used by the on-board controller to control power/temperature (programmable 0–300 °C).

Computational details

All DFT calculations were performed with the turbomole 7.7 software.[1] The def2-SVP basis set, combined with the TPSS GGA functional was used in all geometry optimizations.[2-4] Dispersion was accounted for *via* Grimme D3 dispersion correction, with the Becke-Johnson (BJ) damping factor.[5-9] All optimizations were performed in implicit solvent (tetrahydrofuran), using the COSMO solvation model with standard parameters as implemented in Turbomole.[10] This level of theory is denoted COSMO-TPSS-D3(BJ)/def2-SVP. The nature of each structure (i.e., minimum or transition state) was assessed on the basis of frequency analysis. All minima display only positive frequencies, while transition states display one imaginary frequency. The minimum energy conformer for each structure was identified by combining the CREST routine with the xTB6.6 semi-empirical method by Grimme and coworkers[11-12] and then further reoptimized via DFT. Refined energies were obtained *via* single-point calculations in COSMO (THF), employing the B3LYP hybrid functional[13-14] with D3(BJ) dispersion correction, combined to the larger def2-TZVP basis set. All electronic energies were thus obtained at the COSMO-B3LYP-D3(BJ)/def2-TZVP // COSMO-TPSS-D3(BJ)/def2-SVP level of theory. This level will be denoted from now on as B3LYP // TPSS. B3LYP functional was previously found to provide a good description of copper mediated processes and organic reactivity and was used in pioneering theoretical studies on the copper catalyzed azide-alkyne cycloaddition (CuAAC).[15-18] The resolution of the identity (RI-J and RI-K) approximation, as implemented in Turbomole, were used to expedite the calculation of coulomb and exchange integrals respectively.[19] Thermodynamic corrections were obtained at the lower level of theory through

standard statistical thermodynamics relationships based on the partition function computed under perfect gas approximation at 298.15 K and 1 atm. Very low frequency modes (<100 cm^{-1}) have been treated as free rotors according to Grimme proposal, i.e., using the quasi-rigid rotor harmonic oscillator (quasi-RRHO) approximation.[20] All thermodynamic contributions were estimated with the *thermo* module by Grimme and coworkers.[21] A correction of +1.9 kcal mol^{-1} was applied to all structures to account for the change in standard states between the gas and condensed phase.[22] All energies described in the main text are Gibbs free energies in solvent.

Choice of the chemical model

The "true" nature of the active intermediate(s) in CuAAC (and, thus, also in the interrupted CuAAC) is still a matter of debate. While strong experimental and computational evidence support a bi-metallic mechanism, the intimate nature around [Cu] atoms is more complicated, due to the different species which can be produced in the reaction mixture, in which both the counter ion (in our case, I^-), the solvent (in our case THF) and the (protonated or deprotonated) acetylene can act as ancillary ligands for Cu. This issue has been addressed in both experimental and theoretical papers, such as the pioneering experimental[23] and computational[24] works of Fokin and coworkers. In the latter, both water and Cl^- have been tested as ancillary ligands for [Cu]. The plausible formation of oligomeric copper acetylide species, observed by Straub and coworkers[25] further complicates the picture. Most likely a plethora of ancillary ligands may coordinate to [Cu] in solution.

That said, in our mechanistic investigation, starting from the mono-metallic and bis-metallic reactants, we tried to dissociate one Cu–I bond, using THF to saturate the coordination sphere of Cu in the mono-metallic and bis-metallic intermediates. (Scheme S1) This process leads to the formation of neutral copper complexes. However, at our level of theory, such dissociation appeared to be strongly disfavored from the thermodynamic point of view, with Gibbs free energies of reaction of +15.7 and +19.0 kcal/mol for the mono-metallic and bis-metallic reactant, respectively. Thus, only the reactivity of species with the I^- counterion still bound to Cu was investigated in this work.

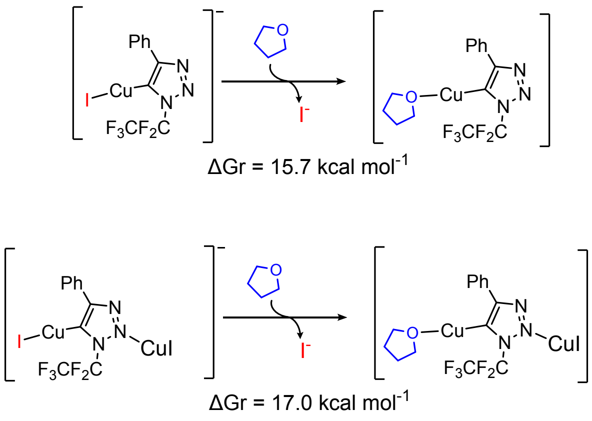

Scheme S1. Dissociation of iodide from the mono-metallic and bis-metallic plausible intermediates in the interrupted click reaction.

Alternative mechanisms

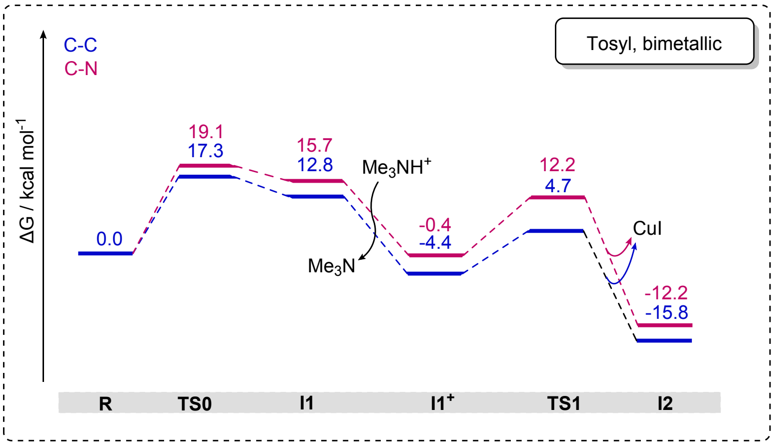

Figure 1. Bimetallic reaction mechanism (B3LYP // TPSS) for C–N and C–C cross-couplings. **R** is the copper triazolate produced by the click reaction toward compound **4a** with a tosyl substituent in place of perfluoroethyl, thus resembling compound **4s** in the current work.

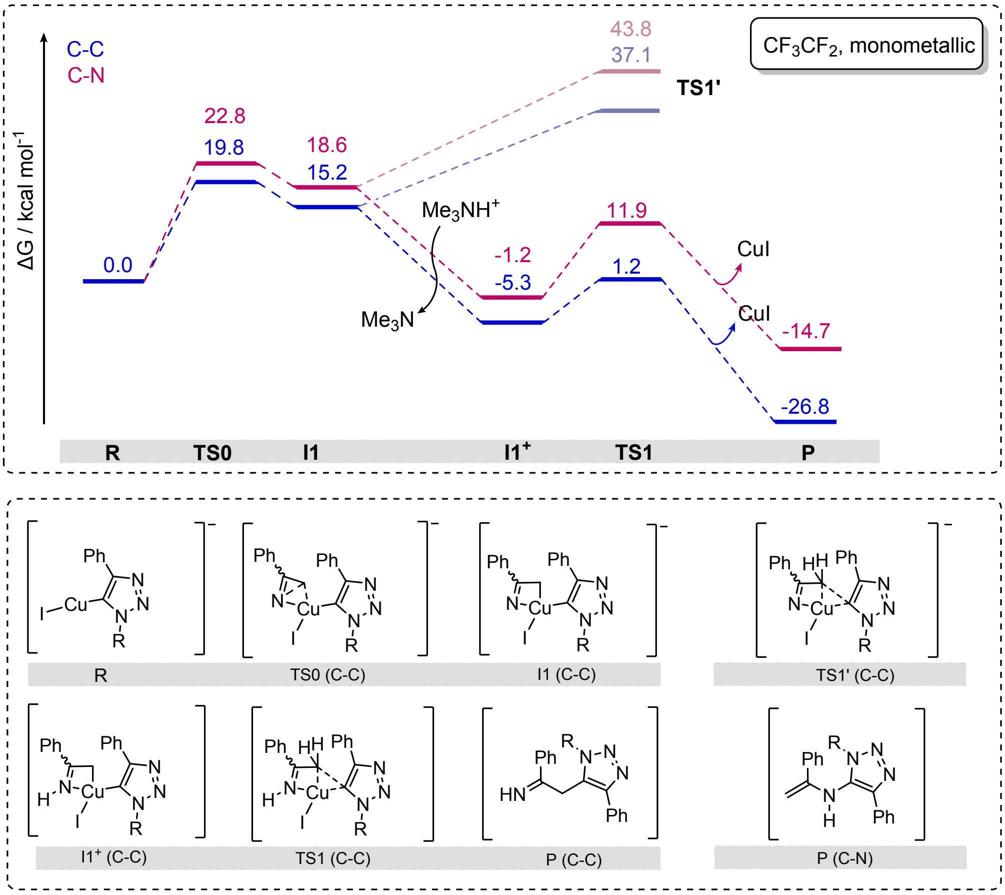

Figure 2. Monometallic reaction mechanism (B3LYP // TPSS) for C–N and C–C cross-couplings. **R** is the copper triazolate produced by the click reaction toward compound **4a.** TS1' is an alternative C–C / C–N cross coupling transition state occurring without prior protonation of **I1**.

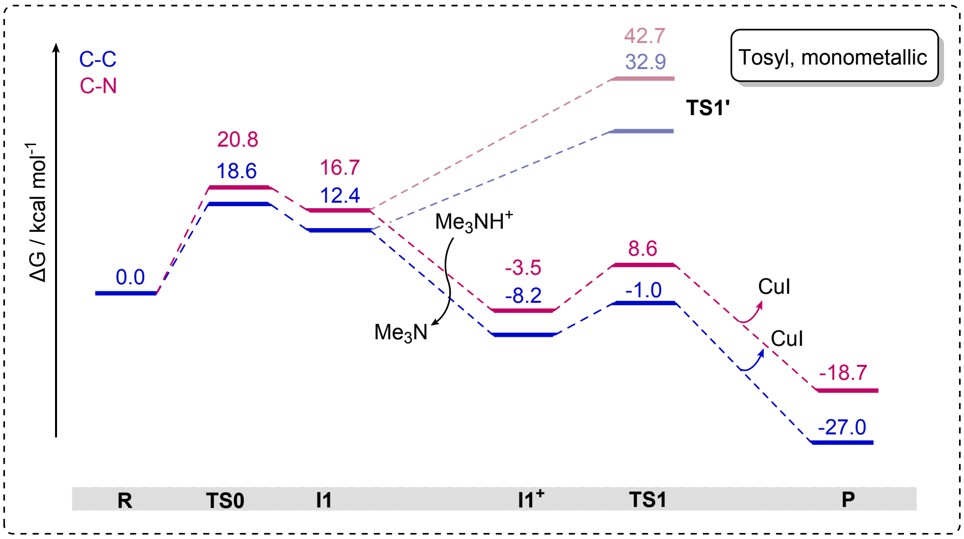

Figure 3. Monometallic reaction mechanism (B3LYP // TPSS) for C–N and C–C cross-couplings. **R** is the copper triazolate produced by the click reaction toward compound, **4a** with a tosyl substituent in place of perfluoroethyl, thus resembling compound **4s** in the current work. **TS1'** is an alternative C–C / C–N cross coupling transition state occurring without prior protonation of **I1**.

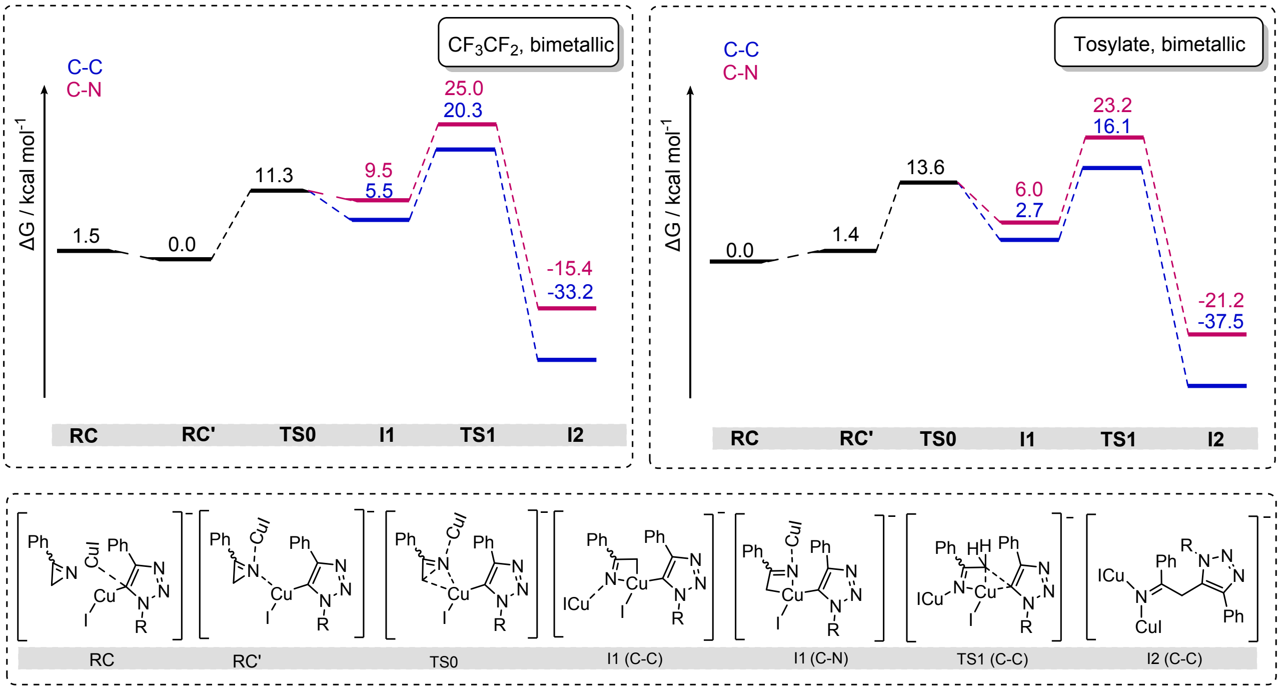

Figure 4. Bimetallic reaction mechanism (B3LYP // TPSS) for C–N and C–C cross-couplings. **RC** is the adduct (reactant complex) between the copper triazolate, 2*H*-azirine and one additional CuI unit, for **4a** (left panel) and **4a** with tosyl group in place of perfluoroethyl (right panel). The additional CuI unit is bonded to azirine nitrogen. The final product is obtained from **I2** after protonation, removal of the two CuI units and eventual tautomerization.

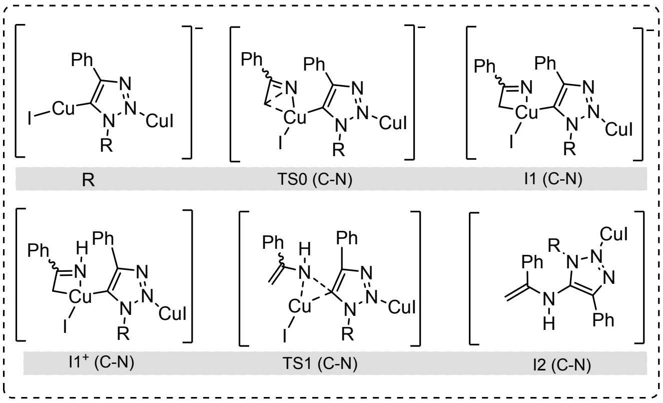

Figure 5. Structures along the C–N cross coupling reaction mechanism (bimetallic mechanism, Figure 1 in the main text).

Table 1. Energies (a.u.), cartesian coordinates (Å) for all optimized geometries.

2CuI, R= CF_2CF_3 (Figure 1 main text)

R

E=-4924.183524

C	-0.407453213	-0.169957007	0.333406515
N	0.065725523	-1.018002843	1.296517877
N	1.304248464	-0.710264337	1.566129831
N	1.626253500	0.380439834	0.764500215
C	0.592477914	0.756215585	-0.058754300
C	-1.785368081	-0.280633616	-0.167600882
C	-2.265940890	0.599395049	-1.161436801
C	-2.656408983	-1.271795089	0.336517098
C	-3.576471402	0.492254064	-1.639320183
H	-1.597487385	1.375896920	-1.559324616
C	-3.967642491	-1.376406973	-0.142778551
H	-2.291233378	-1.956984784	1.107581969
C	-4.435143377	-0.496499479	-1.132436192
H	-3.928041985	1.185673925	-2.410892005
H	-4.629697301	-2.150783483	0.259821423
H	-5.461335239	-0.580134790	-1.505647052
C	2.974135499	0.881721070	0.751573265
C	3.901842953	0.113911846	-0.234662768
F	3.510414990	0.764587497	1.993602599
F	2.972662756	2.183597051	0.400960525
F	5.144826996	0.611118009	-0.179796519
F	3.938577197	-1.187963247	0.099408414
F	3.438135072	0.229028597	-1.487147390
Cu	0.645264560	2.127453294	-1.345401523
I	0.633629994	3.871784815	-3.075144229
Cu	2.429329579	-1.593031411	2.796719669
I	3.824698726	-2.780620497	4.393603611

TS0 (C-C)

E=-5287.86412

C	0.470523499	-0.212903168	-2.035684650
C	0.215708934	1.016425291	-2.694353228
N	-0.481215082	0.815284305	-3.855169231
N	-0.695016538	-0.462029024	-4.008841717
N	-0.099737832	-1.094718902	-2.918331335
C	0.591944947	2.368343187	-2.258683150
C	1.559184807	2.549754867	-1.246290539
C	-0.004331708	3.507801552	-2.843330467
C	1.909271069	3.836439487	-0.819252040
H	2.054884403	1.670491863	-0.815881843
C	0.351274064	4.792719117	-2.415699115
H	-0.750630997	3.371637156	-3.632021953
C	1.306062335	4.964049821	-1.399290213
H	2.665830747	3.957560161	-0.036259001
H	-0.122006772	5.666855304	-2.876387674
H	1.581165912	5.970469052	-1.065771920
Cu	1.244500088	-0.743463291	-0.320494102
I	3.934414349	-0.652264697	-0.308485958
C	0.353193797	-0.914559457	2.271434729
C	0.285551768	0.209167573	1.345380091
H	-0.658716548	0.415126267	0.828026990
H	1.039960134	1.003718427	1.411466576
N	0.894948909	-1.608049102	1.335865679
C	-0.037524544	-1.209787451	3.640761289
C	-0.670174570	-0.213437119	4.414187867
C	0.215061882	-2.481649211	4.202968869
C	-1.047304808	-0.486112353	5.734580958
H	-0.862681313	0.769956138	3.971730100
C	-0.164856558	-2.747540424	5.521090424
H	0.710863043	-3.246274105	3.596015719
C	-0.795849996	-1.751526433	6.288764268
H	-1.537970075	0.287855203	6.333644843
H	0.030891973	-3.732487255	5.957460430
H	-1.090021533	-1.963987885	7.322073771
C	-0.305973583	-2.501859788	-2.713885082

C	-1.654190737	-2.826434609	-2.003480334
F	0.694217068	-2.998386609	-1.954776538
F	-0.306392220	-3.141647347	-3.909742104
F	-2.680477770	-2.372679277	-2.745472754
F	-1.784394745	-4.150675052	-1.847374494
F	-1.698674608	-2.238536307	-0.798753549
Cu	-1.672077782	-1.256500903	-5.411284371
I	-2.989233408	-2.166145002	-7.240455240

TS0 (C-N)

E=-5287.859241

C	0.182596967	0.265880328	-1.964651272
C	-0.342519371	1.485308108	-2.461134864
N	-1.214175653	1.261837344	-3.493294014
N	-1.294496076	-0.019791167	-3.714168719
N	-0.428523052	-0.629337667	-2.801331766
C	-0.052351139	2.846851914	-1.990720921
C	0.962510936	3.074390760	-1.036102383
C	-0.779684994	3.953172726	-2.483988370
C	1.238298749	4.369145202	-0.581919488
H	1.552320774	2.226897179	-0.666735309
C	-0.500923183	5.247349422	-2.028334726
H	-1.565195622	3.783120361	-3.226506893
C	0.507399244	5.462567031	-1.074060969
H	2.033481171	4.523972892	0.155322768
H	-1.075521594	6.094182336	-2.419380427
H	0.724067937	6.475899011	-0.719223558
Cu	1.420264530	-0.203517727	-0.474954110
I	3.915190349	0.161284178	-1.294202848
C	0.907345829	-1.165983133	2.005663687
C	1.733940125	-1.733462622	0.944453957
H	2.824022036	-1.655873060	1.026403061
H	1.307165368	-2.505657174	0.290439960
N	0.698086708	-0.109844547	1.302633526
C	0.505242598	-1.560434237	3.348749461
C	0.960682126	-2.789018282	3.872381574
C	-0.331593596	-0.731083918	4.128985566
C	0.586928235	-3.183080700	5.162935495
H	1.608988196	-3.426315464	3.261078729
C	-0.700790694	-1.129609603	5.416765155
H	-0.683740215	0.218340036	3.712581738
C	-0.242557263	-2.354616441	5.935734834
H	0.942505929	-4.136184257	5.567580163
H	-1.348632029	-0.487494400	6.022511591
H	-0.534722849	-2.662908497	6.945094698
C	-0.408348497	-2.061185226	-2.698827845
C	-1.523011625	-2.620475713	-1.765203135
F	0.785466165	-2.468877903	-2.216396978
F	-0.587109135	-2.611812429	-3.926146123
F	-2.728768506	-2.237815662	-2.215794198
F	-1.472864784	-3.959376801	-1.734591521
F	-1.356900779	-2.151251931	-0.517405680
Cu	-2.390240576	-0.881729256	-4.984660075
I	-3.833832738	-1.973461010	-6.609579772

I1 (C-C)

E=-5287.870535

C	0.071773342	1.853477707	0.802624581
C	-1.284728755	2.219064143	0.648217882
N	-1.385218547	3.527381662	0.261288329
N	-0.188425872	4.034085330	0.150944212
N	0.707054136	3.020327748	0.484915772
C	-2.472405780	1.377444107	0.837493052
C	-2.366202180	0.103919803	1.437859929
C	-3.743253770	1.823542036	0.411559883
C	-3.495608462	-0.708062087	1.594319957
H	-1.392085569	-0.242288775	1.807372798
C	-4.871610106	1.011789549	0.574611989
H	-3.830403717	2.810720382	-0.052081718
C	-4.753434334	-0.258857754	1.162175406

H	-3.392259820	-1.691826371	2.064152636
H	-5.849741996	1.371491116	0.237416550
H	-5.637479572	-0.893149448	1.286202185
Cu	0.874483734	0.153704860	1.300552838
I	1.041429781	0.762362073	3.856730748
C	1.277638630	-1.967511290	0.124274799
C	0.783242647	-0.713025998	-0.501977019
H	1.484085677	-0.156909136	-1.138550709
H	-0.267129292	-0.667054779	-0.824052625
N	1.506130800	-1.658927890	1.360293016
C	1.462650387	-3.291686270	-0.501966303
C	1.214327595	-3.457079524	-1.879097889
C	1.878296550	-4.399604265	0.267764666
C	1.379531563	-4.712078029	-2.481067649
H	0.892112667	-2.595497296	-2.474616365
C	2.042468962	-5.650561157	-0.334306751
H	2.063863901	-4.257427510	1.337930096
C	1.792756830	-5.809550357	-1.709864458
H	1.185655367	-4.835393737	-3.551967308
H	2.363324042	-6.509024806	0.265380071
H	1.919973744	-6.791200757	-2.179021895
C	2.120484521	3.260558324	0.365741317
C	2.623576157	3.297656261	-1.109401634
F	2.790830333	2.279094691	0.997893566
F	2.428795904	4.452414067	0.935278543
F	2.054764169	4.316684448	-1.777044963
F	3.952462824	3.454314513	-1.131029893
F	2.308058047	2.149529236	-1.730794848
Cu	0.191809976	5.827983712	-0.293819058
I	0.518405486	8.159171468	-0.902333737

I1 (C-N)

E=-5287.866131

C	-0.317061932	1.242959197	-1.171527201
C	-1.054981249	2.379093207	-0.766457382
N	-1.909755717	2.779576780	-1.757120889
N	-1.784258905	1.976540330	-2.777230773
N	-0.825427238	1.029771047	-2.421088447
C	-0.984798116	3.087596855	0.517627712
C	-0.237860872	2.557432023	1.591352876
C	-1.666927992	4.309976010	0.705419412
C	-0.167409865	3.231093378	2.815646636
H	0.285968210	1.602426979	1.460950511
C	-1.597082395	4.981701440	1.931633631
H	-2.248740559	4.724749094	-0.123227785
C	-0.846209898	4.447701103	2.991966884
H	0.418300881	2.802562971	3.635853810
H	-2.130784794	5.929647793	2.059872941
H	-0.792040617	4.975500365	3.950058740
Cu	1.077145966	0.166452769	-0.264917723
I	2.999543845	1.851057324	-0.736601798
C	1.008150858	-2.007791223	0.903034881
C	2.201231482	-1.135871268	0.742292163
H	2.564649953	-0.621626046	1.643909758
H	2.995557615	-1.484068055	0.065500168
N	0.041943973	-1.347571663	0.352249078
C	0.908954289	-3.340165290	1.532206365
C	2.046710751	-3.909393085	2.138176342
C	-0.309040039	-4.054236481	1.531024162
C	1.969689261	-5.174156562	2.737738796
H	2.990472709	-3.352573683	2.136832533
C	-0.383630562	-5.316243257	2.128436437
H	-1.185506844	-3.603294193	1.053181200
C	0.755622493	-5.878652020	2.733227930
H	2.856584806	-5.611439969	3.208723086
H	-1.329021613	-5.869186944	2.124192446
H	0.695271697	-6.867607528	3.200100339
C	-0.456526559	0.013149548	-3.371013834
C	0.475452431	0.518436226	-4.512455560
F	-1.577432391	-0.488627469	-3.951049973

F	0.187247303	-0.981184767	-2.729380054
F	-0.142681506	1.475633905	-5.230493955
F	0.779138380	-0.502638628	-5.327488511
F	1.602535529	1.020328645	-4.000355337
Cu	-2.778768551	2.086034514	-4.375596253
I	-4.134224216	2.356906625	-6.375203363

I1+ (C-C)

E=-5288.34792

C	0.464911447	1.800004660	0.640353530
C	-0.921100183	1.805929852	0.410595066
N	-1.339226944	3.091142132	0.194183680
N	-0.313380308	3.895952009	0.266599131
N	0.799084962	3.120347776	0.547405228
C	-1.823136105	0.650828552	0.339955666
C	-1.461234467	-0.574812335	0.938292509
C	-3.045294539	0.737027436	-0.360984774
C	-2.287153628	-1.697684368	0.817882101
H	-0.540693656	-0.638980003	1.533647416
C	-3.872227694	-0.386601548	-0.473336706
H	-3.329890943	1.686242683	-0.824773481
C	-3.493052604	-1.608401412	0.107075680
H	-1.987654683	-2.641586669	1.282721231
H	-4.815040640	-0.311086435	-1.024994651
H	-4.139192291	-2.486868259	0.009700762
Cu	1.639978844	0.363970736	1.022823124
I	1.671741971	0.492659287	3.544115566
C	1.763980074	-1.500789819	-0.197545151
C	1.527451390	-0.259244603	-0.923701656
H	2.409907837	0.302561363	-1.252229728
H	0.604088482	-0.103590301	-1.488362418
N	2.664547402	-1.271942788	0.764830575
C	0.941664373	-2.710707383	-0.351365929
C	0.203130740	-2.915354168	-1.537218362
C	0.844506196	-3.649527365	0.699738462
C	-0.619543225	-4.038091282	-1.667283212
H	0.280286915	-2.198749307	-2.360534976
C	0.022707966	-4.771197709	0.564592758
H	1.384790988	-3.488132409	1.638308094
C	-0.712028704	-4.964994391	-0.617494600
H	-1.191381188	-4.190356463	-2.587428845
H	-0.057875411	-5.489237905	1.386395581
H	-1.362386405	-5.839941791	-0.717261595
C	2.117133427	3.712227300	0.554907824
C	2.643515108	4.050909300	-0.873860797
F	2.976816925	2.827932450	1.103546241
F	2.100922149	4.842742469	1.287256655
F	1.866474630	4.977852776	-1.455044697
F	3.893986554	4.510889037	-0.796005398
F	2.626466412	2.936678822	-1.625471592
Cu	-0.380548162	5.776264438	0.122997090
I	-0.562809795	8.185516565	-0.088677921
H	2.806756786	-2.029800928	1.445652519

I+ (C-N)

E=-5288.339662

C	-1.741841509	0.172019936	-1.117619953
C	-1.625291148	1.245488226	-0.217567584
N	-2.870190395	1.718094424	0.097500793
N	-3.769908615	1.022264212	-0.540470481
N	-3.091031095	0.064178044	-1.286681608
C	-0.394168853	1.800440336	0.362010272
C	-0.412969717	2.446936080	1.615888875
C	0.831176621	1.679287530	-0.327688706
C	0.772746585	2.948155364	2.166242450
H	-1.360989112	2.544149472	2.153458913
C	2.014688668	2.179739799	0.226130690
H	0.847698828	1.219094663	-1.323784133
C	1.989091902	2.813407157	1.476877102
H	0.748882357	3.442217437	3.143045979

H	2.956702794	2.074357433	-0.320995868
H	2.914518538	3.200532602	1.915212503
Cu	-0.276628983	-0.946652112	-1.716640080
I	0.171579872	-0.085403626	-4.013939980
C	1.210156964	-1.844804232	-0.314280888
C	1.462008066	-2.077286605	-1.720008304
H	2.372515570	-1.718878183	-2.206592837
H	0.964940341	-2.942021549	-2.175246300
N	-0.107845130	-1.987862829	-0.085324059
C	2.189386388	-1.310814941	0.646847821
C	1.762643415	-0.791899977	1.889337263
C	3.564911222	-1.300841652	0.328402218
C	2.691800687	-0.275804144	2.794001703
H	0.698659029	-0.761120968	2.144145250
C	4.492258725	-0.773427277	1.233372511
H	3.911075042	-1.712635526	-0.623920564
C	4.057668402	-0.262176146	2.466086282
H	2.350082026	0.133401778	3.748966434
H	5.556444294	-0.765584594	0.978838834
H	4.784250926	0.151867854	3.172332925
C	-3.793882590	-0.868790665	-2.134415018
C	-3.381822523	-2.356191119	-1.878236975
F	-3.573994081	-0.584231982	-3.434467365
F	-5.120302486	-0.769598928	-1.890498505
F	-2.186089398	-2.619933989	-2.448012225
F	-4.291498065	-3.168229922	-2.421277152
F	-3.301729805	-2.593545341	-0.564393421
H	-0.433930155	-1.668050345	0.836512899
Cu	-5.621773050	1.369787213	-0.446450779
I	-7.960000552	1.960367090	-0.186698932

TS1 (C-C)

E=-5288.33285

C	0.253241617	-0.250879073	1.268854992
C	0.444246285	-1.537897016	1.816950155
N	-0.592456058	-1.827746044	2.656129498
N	-1.433839097	-0.827925780	2.673334333
N	-0.945417198	0.143493745	1.818093686
C	1.550674410	-2.466500310	1.582740955
C	2.778588721	-2.004379595	1.060787764
C	1.403174580	-3.839108668	1.881547599
C	3.830238868	-2.895896319	0.822076339
H	2.930907778	-0.931074414	0.880411124
C	2.459800861	-4.725324961	1.649221868
H	0.454168592	-4.197787366	2.290579104
C	3.672834164	-4.259334009	1.114475153
H	4.777538799	-2.520515912	0.422749829
H	2.336301769	-5.787540005	1.883472302
H	4.496607944	-4.957177406	0.933565261
Cu	1.568139493	0.955159018	0.583070559
I	2.720473837	2.000144262	2.536156038
C	0.760085125	1.077872669	-1.574568732
C	0.377247345	-0.143861720	-0.788237462
H	-0.676822877	-0.427316332	-0.828484971
H	1.053619480	-0.995324495	-0.941388492
N	1.982478710	1.483121706	-1.295731487
C	-0.192759923	1.746281328	-2.482424106
C	-1.315532333	1.053637927	-2.989541976
C	0.027434360	3.085017656	-2.881956370
C	-2.191457202	1.683103131	-3.880136656
H	-1.495127542	0.011820609	-2.710228229
C	-0.852237867	3.711548397	-3.767909880
H	0.876877356	3.646579143	-2.479472153
C	-1.963078483	3.011712298	-4.269136250
H	-3.053148757	1.134944430	-4.273028222
H	-0.678120256	4.750723703	-4.063425840
H	-2.652399952	3.505203123	-4.961771199
C	-1.629790700	1.405311843	1.672227978
C	-3.010532936	1.288948245	0.953880674
F	-0.851650940	2.211185619	0.912175345

F	-1.819565841	1.971989903	2.879008790
F	-3.874175871	0.574034455	1.689521470
F	-3.511377880	2.507535330	0.749748768
F	-2.839089957	0.675899606	-0.231469066
H	2.292298570	2.332330028	-1.785101069
Cu	-2.858000790	-0.704927193	3.901238976
I	-4.610396206	-0.667081555	5.571993602

TS1 (C-N)

E=-5288.312235

C	-0.693614091	-0.656441367	-1.001290826
C	0.669230443	-0.716519218	-0.594775331
N	1.309908184	0.418827466	-0.996507196
N	0.473506805	1.207790350	-1.618780998
N	-0.758073743	0.561979971	-1.641329299
C	1.343058394	-1.739306973	0.193381415
C	0.783993502	-3.028467232	0.342642849
C	2.568102353	-1.444039959	0.837566882
C	1.416788089	-3.993205126	1.130951580
H	-0.135482165	-3.295256957	-0.197937319
C	3.199981114	-2.413167658	1.620861347
H	3.008078606	-0.450174267	0.716154134
C	2.625255840	-3.686802179	1.775434678
H	0.971613797	-4.987815237	1.231291580
H	4.145982254	-2.175488590	2.117364063
H	3.123997394	-4.441973014	2.391246261
Cu	-1.951049908	-2.164333337	-0.898864096
I	-2.364457419	-4.074584602	-2.359684721
C	-1.231756716	-0.341809173	1.536941793
C	-0.937895409	-1.260347160	2.490663396
H	-0.345854585	-0.997758552	3.370908242
H	-1.319991430	-2.281127654	2.405477548
N	-1.997719185	-0.754122917	0.419220290
C	-0.688313011	1.046320664	1.501740846
C	0.614852751	1.311478856	1.986735814
C	-1.392999153	2.086773928	0.862898810
C	1.214177949	2.557438595	1.788466378
H	1.178595549	0.513930286	2.478865521
C	-0.801386036	3.347554080	0.664977700
H	-2.418025341	1.931412116	0.509583186
C	0.521772747	3.583443095	1.113061197
H	2.231966935	2.735122040	2.147805950
H	-1.384433053	4.161287781	0.220490581
H	0.969087943	4.576192868	1.006261541
C	-1.810856409	1.070243128	-2.480361484
C	-1.668575522	0.663574410	-3.978524689
F	-1.822173585	2.418849387	-2.400952759
F	-2.989124377	0.585013846	-2.014979593
F	-1.604305168	-0.671522300	-4.072672282
F	-2.723587912	1.106311154	-4.670604505
F	-0.548326610	1.197598957	-4.479781604
H	-2.569848099	0.015867529	0.053430766
Cu	0.600891583	3.130893400	-1.275400602
I	1.187006695	5.346359566	-2.231977043

I2 (C-C)

E=-3350.124099

C	-0.259360799	0.554341943	-0.611414962
C	-0.073164949	1.669106416	0.222202714
N	-1.246208939	1.920716046	0.883789096
N	-2.142332999	1.044213491	0.531556950
N	-1.565455010	0.182241891	-0.372674329
C	1.095405485	2.539426503	0.426709625
C	2.412363424	2.130427586	0.121082015
C	0.883595069	3.832336032	0.958631059
C	3.486001229	3.005897661	0.332698680
H	2.621518823	1.127089279	-0.260113134
C	1.961217169	4.697575666	1.169377233
H	-0.135179612	4.149879632	1.198312998
C	3.267227864	4.289106100	0.853860167

H	4.501875143	2.675036338	0.093963810
H	1.780772997	5.696988652	1.578424595
H	4.110541483	4.967872085	1.017571286
C	1.552597365	-1.221115935	-0.811676891
C	0.693356845	-0.157530546	-1.516487979
H	1.372354217	0.580691260	-1.968166105
N	2.819111340	-1.172116187	-1.009340840
C	0.843615895	-2.267274448	-0.010785431
C	0.163556241	-1.943600598	1.182090440
C	0.827953837	-3.598455376	-0.473852322
C	-0.537605795	-2.932034008	1.885457451
H	0.196106948	-0.922374877	1.574441163
C	0.120306280	-4.582245892	0.229885814
H	1.351090860	-3.854068172	-1.401294284
C	-0.570393579	-4.249930889	1.405106845
H	-1.060443637	-2.669796104	2.810921130
H	0.101806645	-5.610416090	-0.146286369
H	-1.130410876	-5.017712311	1.948820015
C	-2.410565135	-0.809604003	-1.018996918
C	-3.218380347	-0.208640505	-2.209631044
F	-3.274190519	-1.299223859	-0.106890690
F	-1.654564192	-1.813424712	-1.492028895
F	-2.357031813	0.329359570	-3.088661218
F	-3.923713537	-1.168017745	-2.810134204
F	-4.048120314	0.746356993	-1.765042066
H	0.146005827	-0.652808101	-2.333305107
Cu	-3.892793099	0.981359776	1.243119256
I	-6.092340508	0.982454711	2.253792280
H	3.283874671	-1.952087273	-0.515031832

I2 (C-N)

E=-3350.113562

C	0.719364546	-0.766868395	0.042389328
C	0.421637847	-2.135625723	-0.045374788
N	1.581788478	-2.843411383	0.100935376
N	2.582690787	-2.021942085	0.252057678
N	2.094904875	-0.729852025	0.213285731
C	-0.878043187	-2.792790622	-0.236869893
C	-1.067908251	-4.107643074	0.240255489
C	-1.938043191	-2.139006922	-0.902880509
C	-2.298676702	-4.749292919	0.064078644
H	-0.244648545	-4.615211730	0.751674026
C	-3.167825720	-2.786518613	-1.072595396
H	-1.795771802	-1.136244862	-1.317741088
C	-3.353379476	-4.090062297	-0.587340020
H	-2.436854448	-5.768166979	0.440230380
H	-3.980873083	-2.272676206	-1.595395473
H	-4.316256165	-4.593738038	-0.721161357
C	-0.108186693	1.344093941	-0.943794280
C	0.453974353	1.130870537	-2.154657685
H	0.534115574	1.943586074	-2.880258727
H	0.885316755	0.160995834	-2.423121959
N	-0.087989382	0.345611173	0.077351935
C	-0.737165218	2.615826906	-0.515012571
C	-0.688463286	3.012290185	0.839742351
C	-1.378791424	3.458990923	-1.448668703
C	-1.256048414	4.226846359	1.246998678
H	-0.179158374	2.372971398	1.567007720
C	-1.941236654	4.673297265	-1.039136395
H	-1.451151598	3.149135178	-2.496124967
C	-1.882786017	5.061864781	0.309693757
H	-1.202327024	4.524181630	2.299519883
H	-2.438939587	5.315146636	-1.773537101
H	-2.328476210	6.009903401	0.628569148
C	2.948717451	0.353685477	0.661951430
C	3.033599292	0.433820185	2.218727619
F	2.479448245	1.522782522	0.207125562
F	4.193323105	0.153755461	0.174783168
F	3.784914166	1.477363992	2.575859845
F	1.797097185	0.583164367	2.723831593

F	3.570090519	-0.692633833	2.709597232
Cu	4.387469790	-2.542017142	0.473450796
I	6.675161338	-3.276105718	0.788924340
H	-0.984613857	0.189624341	0.545629202

Imine product

E=-1411.857181

C	-1.326543374	-0.621645107	-0.551546426
C	-0.539692744	-1.340735983	0.347436052
N	-1.333271261	-1.736321089	1.405544146
N	-2.550979225	-1.336056352	1.231304481
N	-2.576045357	-0.655647917	0.038444513
C	0.905682878	-1.602511711	0.325941664
C	1.577666775	-1.915914605	-0.872684754
C	1.642044911	-1.500238400	1.525200704
C	2.966928829	-2.091854622	-0.877493229
H	1.011020206	-2.031465614	-1.801195621
C	3.028912433	-1.680290290	1.516589243
H	1.117193453	-1.254939433	2.453062953
C	3.695978298	-1.967816419	0.314471451
H	3.479922319	-2.328421070	-1.815188720
H	3.594916851	-1.584696593	2.449002845
H	4.782991600	-2.099410279	0.308177522
C	0.368227714	0.811779634	-1.811939513
C	-1.012681965	0.140779690	-1.807021778
H	-1.773151977	0.926599860	-1.955134850
N	1.138621535	0.811095306	-2.842087197
C	0.819885296	1.494398268	-0.567866963
C	-0.092257636	2.004564500	0.379427015
C	2.202949521	1.600353414	-0.314004627
C	0.370978065	2.602426946	1.558578794
H	-1.169607463	1.951510314	0.197313319
C	2.663906684	2.186905850	0.868058680
H	2.898628393	1.186282575	-1.048955767
C	1.748873296	2.687128720	1.809516531
H	-0.346691971	2.999556366	2.283755685
H	3.739574759	2.244442968	1.064424773
H	2.109252284	3.140158220	2.739163987
C	-3.774925877	-0.025998067	-0.424360380
C	-5.051698307	-0.365213244	0.406001716
F	-4.006831985	-0.379594669	-1.713266517
F	-3.623135383	1.332024101	-0.405280183
F	-6.089298135	0.237786290	-0.198951858
F	-5.274659574	-1.683222678	0.426526461
F	-4.955641480	0.102539233	1.654011711
H	0.713834088	0.263188605	-3.607155981
H	-1.080876476	-0.521526717	-

2.687819884

Enamine product

E=-1411.862296

C	-1.505481474	-0.117528116	-0.586984614
C	-0.734072214	-1.273881266	-0.378547215
N	-1.528359617	-2.241144235	0.191855543
N	-2.742949166	-1.813599710	0.334577261
N	-2.758987445	-0.516616857	-0.142004381
C	0.684769713	-1.512395984	-0.682383589
C	1.267570461	-1.017115788	-1.868520624
C	1.481694054	-2.248664289	0.217230727
C	2.621695032	-1.243427747	-2.139516764
H	0.652011956	-0.453091897	-2.576204894
C	2.837207610	-2.470191300	-0.055933261
H	1.030599844	-2.630065320	1.138007535
C	3.412677500	-1.965785150	-1.231803918
H	3.061853756	-0.853949607	-3.063736214
H	3.449118623	-3.032896000	0.657076956
H	4.474101594	-2.135671074	-1.441531196
C	-0.048276172	1.887822382	-0.748008262
C	-1.202007094	1.208268944	-1.078636108
H	-1.914577062	1.674403542	-1.768491554

N	0.367846954	2.998161227	-1.436763992
C	0.845366495	1.451238336	0.358095932
C	2.243297579	1.415935839	0.182389456
C	0.297183154	1.067377126	1.599714559
C	3.074635790	0.971572415	1.218028672
H	2.670323789	1.702904210	-0.783059441
C	1.130705698	0.628472626	2.634371205
H	-0.785740963	1.123902161	1.747401522
C	2.520519208	0.572528432	2.443283085
H	4.157291122	0.924206481	1.063041366
H	0.694999631	0.331911049	3.594154428
H	3.170845740	0.219080122	3.250339885
H	0.935568610	3.683012602	-0.939058815
C	-3.918019431	0.302758143	-0.023514548
C	-5.154412904	-0.409990951	0.603717719
F	-4.293167921	0.771409281	-1.246678430
F	-3.642845623	1.392188198	0.749661195
F	-5.522202931	-1.471548514	-0.121824898
F	-4.909668171	-0.782328748	1.865205843
F	-6.170610751	0.472815540	0.611497402
H	-0.250504976	3.389923899	-2.146447573

2CuI, R= Tosyl (Figure 3)

R

E=-5168.23126

C	-0.309330329	2.139400154	0.370579996
N	0.730193673	2.455371415	-0.466463847
N	0.595349775	1.776661660	-1.577490590
N	-0.562601278	1.039352125	-1.448542654
C	-1.171029801	1.186624731	-0.233204679
S	-0.987588267	-0.132191232	-2.695928939
O	-2.449831738	-0.261894988	-2.618346716
O	-0.285637850	0.371719469	-3.896686697
C	-0.197401723	-1.593633450	-2.092108930
C	-0.890339357	-2.404964484	-1.178640249
C	-0.222468328	-3.505060451	-0.636244421
C	1.113609145	-3.798208925	-0.990976765
C	1.772735885	-2.957189030	-1.911437839
C	1.129118241	-1.846978981	-2.468673773
C	1.813424189	-4.995462806	-0.395732735
H	-1.923271751	-2.170427919	-0.898845575
H	-0.748237491	-4.147697619	0.077811260
H	2.807613524	-3.172555872	-2.196892944
H	1.643584389	-1.188923682	-3.178772292
H	1.311986539	-5.930933175	-0.703951729
H	2.866547655	-5.045875007	-0.714590103
H	1.780844733	-4.958615541	0.707533829
C	-0.427629684	2.746339758	1.703886622
C	0.533421988	3.673346444	2.165554577
C	-1.504846317	2.408835697	2.552954188
C	0.418322392	4.241402105	3.440217141
H	1.369576432	3.940094269	1.512090140
C	-1.616001551	2.976352031	3.827096634
H	-2.258942319	1.693039044	2.198419783
C	-0.654742299	3.895909235	4.278082642
H	1.172231664	4.958844076	3.782362177
H	-2.458346963	2.700351035	4.470599927
H	-0.742130641	4.341196444	5.274894168
Cu	-2.653961406	0.158360463	0.308668656
I	-4.469755953	-1.249626377	1.189692106
Cu	1.800695723	1.511552613	-3.018306345
I	3.674839100	1.145486774	-4.538606025

TS0 (C-C)

E=-5531.91385

C	-0.976513089	0.499943555	-0.048231219
C	-1.618270256	1.582327588	-0.707957256
N	-2.972154925	1.377760301	-0.791677485
N	-3.252554111	0.227373114	-0.232616477

N	-2.058146500	-0.283254673	0.229846540
C	-0.995811021	2.789020340	-1.268414420
C	0.393854785	2.817569947	-1.519006388
C	-1.768124363	3.930060310	-1.579980734
C	0.997270222	3.963322247	-2.051061621
H	0.986069248	1.917391613	-1.317246021
C	-1.160902845	5.072857196	-2.115409156
H	-2.846200528	3.909808145	-1.392915855
C	0.224235705	5.097579618	-2.349246153
H	2.075691982	3.966475164	-2.243876552
H	-1.772030956	5.951804134	-2.348596742
H	0.696637715	5.993658480	-2.766212836
S	-2.028772213	-1.928935965	0.854522558
O	-0.651843159	-2.081604480	1.358283847
O	-3.215799705	-2.034938874	1.724727772
C	-2.279206698	-2.858230660	-0.629786987
C	-1.231983791	-2.923580154	-1.560620879
C	-3.538191543	-3.429968718	-0.867098521
C	-1.468547634	-3.585549867	-2.768008145
H	-0.261525797	-2.453422946	-1.356952596
C	-3.744848038	-4.088255119	-2.083716871
H	-4.330176528	-3.363743323	-0.114896974
C	-2.722208802	-4.171161938	-3.051573900
H	-0.662351224	-3.643582756	-3.507340534
H	-4.721968114	-4.540361798	-2.283364926
C	-2.973522473	-4.846129383	-4.377608582
H	-3.426027253	-4.128776510	-5.088389296
H	-2.035758125	-5.212649685	-4.826156121
H	-3.674963895	-5.690476460	-4.271631136
Cu	0.817629360	-0.035627135	0.514275671
I	2.227891339	-0.973043681	-1.624281134
C	2.781197544	0.639401333	2.335466964
C	2.035984313	1.504537278	1.432565828
H	1.201208179	2.098478472	1.822567355
H	2.491388914	1.789938697	0.475575036
N	2.068533811	-0.340216211	1.909793320
C	3.841921579	0.756167673	3.322507448
C	4.398481441	2.022660496	3.600627303
C	4.322195740	-0.387305555	4.000805207
C	5.421608947	2.145163181	4.548508710
H	4.021718320	2.903640871	3.069742218
C	5.343218450	-0.257952781	4.946003912
H	3.887302166	-1.366288600	3.773869955
C	5.894418947	1.006930497	5.221466064
H	5.852244078	3.128523247	4.763472685
H	5.715835557	-1.143276225	5.471614037
H	6.695423737	1.103523613	5.962004555
Cu	-4.893104832	-0.703311413	-0.097274279
I	-7.110453663	-1.724272198	-0.097097190

TS0 (C-N)

E=-5531.90964

C	0.587185362	0.877967737	0.149791357
C	1.040001785	2.055169995	0.805827246
N	2.328854921	2.357659707	0.445934001
N	2.744984964	1.456194816	-0.405079847
N	1.688606999	0.582675232	-0.595597054
C	0.300232248	2.894727943	1.758504852
C	-1.006172253	2.542385290	2.162819165
C	0.880528294	4.067433935	2.293171009
C	-1.712254005	3.338843955	3.071726141
H	-1.461086224	1.629244155	1.763948165
C	0.171474095	4.862214304	3.201990602
H	1.893334128	4.345287296	1.985919484
C	-1.128600515	4.503725145	3.595899718
H	-2.723221584	3.045022092	3.374323964
H	0.636481878	5.768075536	3.606367099
H	-1.681927680	5.126802794	4.306884228
S	1.982951202	-0.838243668	-1.591747970
O	0.656288476	-1.448737046	-1.773441992

O	2.789128986	-0.343964046	-2.726937964
C	2.996715282	-1.816171227	-0.521221090
C	2.391491006	-2.450885494	0.573207547
C	4.377264924	-1.879448516	-0.762344050
C	3.207290355	-3.172006845	1.448693579
H	1.313502674	-2.362555168	0.751421942
C	5.169005068	-2.609770461	0.129706407
H	4.815296408	-1.372075530	-1.627827283
C	4.601943720	-3.260847912	1.245771072
H	2.750153189	-3.669436284	2.310681428
H	6.248349984	-2.670518140	-0.045016514
C	5.474913901	-4.014277955	2.219459825
H	5.891436620	-3.319041416	2.972800527
H	4.901224931	-4.785412920	2.758682564
H	6.326385081	-4.492065634	1.706968519
Cu	-1.066763455	-0.216391949	0.235299485
I	-0.959476274	-1.636601720	2.496941233
C	-3.345211313	-0.334943632	-1.287309266
C	-2.449121862	-1.405578722	-0.865265100
H	-2.760127520	-2.056603245	-0.039986710
H	-1.651677229	-1.741369117	-1.540947856
N	-2.662479641	0.494046520	-0.582811903
C	-4.540130707	-0.195472073	-2.106668570
C	-5.081314804	-1.330233958	-2.746961912
C	-5.154785133	1.066266558	-2.274085818
C	-6.225268884	-1.205515788	-3.544840163
H	-4.598590948	-2.304403511	-2.613241176
C	-6.296172359	1.184140412	-3.071722754
H	-4.724394519	1.941415108	-1.776113454
C	-6.832920867	0.049862879	-3.708119641
H	-6.643951546	-2.086968824	-4.041398672
H	-6.772266528	2.161351032	-3.202725068
H	-7.726317296	0.147045541	-4.333917705
Cu	4.403450161	1.272453762	-1.298602740
I	6.635756501	1.249529056	-2.288808890

I1 (C-C)

E=-5531.92274

C	0.959724844	0.193328392	-3.276381017
N	0.699770857	1.273404551	-4.080369334
N	-0.522288458	1.681518536	-3.858045694
N	-1.048201635	0.846659370	-2.888216364
C	-0.172704141	-0.115493631	-2.486353681
S	-2.758991154	1.028708925	-2.455876245
O	-3.049218220	2.412152691	-2.906805996
O	-3.480364044	-0.124974006	-3.003802987
C	-2.690498261	0.946683270	-0.692734239
C	-2.343675954	2.103489078	0.025744639
C	-2.295207897	2.024713768	1.418616561
C	-2.589531774	0.818479119	2.096825910
C	-2.933422550	-0.316996404	1.338047171
C	-2.986061752	-0.270461627	-0.060360353
C	-2.538522836	0.762453463	3.603021662
H	-2.121354251	3.036736791	-0.499013018
H	-2.027197784	2.916925300	1.994071741
H	-3.151313939	-1.261318755	1.845660658
H	-3.254763500	-1.152222121	-0.649673681
H	-3.218964355	1.513133588	4.043328697
H	-2.821097580	-0.233337370	3.977212775
H	-1.519972384	0.989138238	3.964567745
C	2.269475863	-0.469247346	-3.270910101
C	3.363110798	0.088085492	-3.970984299
C	2.464204087	-1.672207122	-2.556853881
C	4.614393748	-0.538924902	-3.948395456
H	3.217730843	1.018811987	-4.527586009
C	3.718162818	-2.293630107	-2.531370285
H	1.616713739	-2.132936914	-2.033058406
C	4.800052885	-1.729907756	-3.226338371
H	5.452485247	-0.093492412	-4.495506566
H	3.846715485	-3.226281326	-1.972027675

H	5.780731504	-2.217177165	-3.208744444
Cu	-0.330035396	-1.324121018	-0.970518765
N	-0.185981850	-2.107856546	0.784474277
C	0.332689929	-1.080176999	1.375663812
C	0.613234554	-0.099354840	0.295087973
H	1.646443222	-0.046213137	-0.075926722
H	0.079120810	0.860543627	0.305200519
C	0.524441050	-0.901235620	2.828184905
C	0.034916081	-1.858439951	3.741961191
C	1.157974431	0.260508306	3.312232419
C	0.176159272	-1.652549885	5.117557358
H	-0.464897614	-2.751019500	3.350749735
C	1.298650752	0.466198454	4.691603138
H	1.532836102	1.003977359	2.599971447
C	0.806128941	-0.488801240	5.595365220
H	-0.209945368	-2.393941223	5.825171956
H	1.789391570	1.371998138	5.063156425
H	0.910518977	-0.326808774	6.673643018
I	-1.451447222	-3.317153481	-2.253098022
Cu	-1.436918919	3.229884278	-4.472002147
I	-2.103199569	5.348748453	-5.496167195

I1 (C-N)

E=-5531.91677

C	1.687429757	1.359278915	1.098934869
N	2.893363443	1.282041862	0.450911110
N	2.755441172	0.498551568	-0.591095013
N	1.441660820	0.082205794	-0.596642062
C	0.714086660	0.569745179	0.439513776
S	0.872098262	-1.005688034	-1.868307727
O	-0.535505677	-1.247931822	-1.502813209
O	1.265752959	-0.379234706	-3.142299440
C	1.872994767	-2.432771466	-1.566717871
C	1.601263733	-3.218937890	-0.435493634
C	2.439635438	-4.302975772	-0.171589877
C	3.533509380	-4.608028469	-1.014870999
C	3.770436904	-3.794978005	-2.140281026
C	2.946778862	-2.698767694	-2.428811197
C	4.418505111	-5.791657342	-0.710030480
H	0.764880066	-2.974197229	0.230104151
H	2.245462669	-4.924133411	0.709234440
H	4.616463295	-4.010169220	-2.800598954
H	3.119767896	-2.075329258	-3.310118985
H	3.853060733	-6.735108613	-0.822257798
H	5.288621734	-5.827691961	-1.384071677
H	4.779361204	-5.752940992	0.332909400
C	1.506493523	2.168901062	2.310388494
C	2.588663180	2.867380421	2.890403978
C	0.235270783	2.262653715	2.918577346
C	2.400473827	3.635902674	4.045900514
H	3.576305239	2.798971234	2.424089244
C	0.050484491	3.030822756	4.073564864
H	-0.609205387	1.723053810	2.473528095
C	1.132218954	3.721814558	4.643794737
H	3.250093377	4.171669936	4.483457743
H	-0.943370852	3.089544006	4.530137532
H	0.987930291	4.323684170	5.547404160
Cu	-1.192805032	0.098911540	0.664832487
N	-2.030125855	1.458341551	-0.426184919
C	-3.251465414	1.054647412	-0.300997110
C	-3.174657282	-0.091880507	0.642443185
H	-3.398972605	-1.092735954	0.244795962
H	-3.535664106	0.086823957	1.665603104
C	-4.432600651	1.632743947	-0.974146494
C	-4.295071037	2.706232504	-1.880810615
C	-5.712684713	1.105721891	-0.711883033
C	-5.422229443	3.242042436	-2.511071378
H	-3.294234105	3.104557526	-2.079085078
C	-6.841751909	1.643732494	-1.345169834
H	-5.815471947	0.272377269	-0.007682684

C	-6.698076057	2.711563680	-2.244852022
H	-5.312239087	4.074294357	-3.214600254
H	-7.834551149	1.230437640	-1.137754751
H	-7.579637583	3.131920331	-2.740930773
I	-0.733894321	-1.776494918	2.430175656
Cu	4.066415228	-0.009896070	-1.852783480
I	5.969290456	-0.389020857	-3.336752476

I1+ (C-C)

E=-5532.40052

C	2.093909588	-0.519626155	-1.537056164
N	1.485052912	-1.572731217	-2.169702915
N	0.253538702	-1.680845042	-1.727627262
N	0.065352189	-0.691149983	-0.805091071
C	1.183798352	0.057449508	-0.630216594
S	-1.513563836	-0.464772024	0.013788868
O	-1.179948751	0.542551566	1.040110070
O	-2.482087174	-0.203644178	-1.058599827
C	-1.730773686	-2.071199452	0.691305179
C	-0.924136640	-2.461091297	1.774347821
C	-1.012028484	-3.781667981	2.212820777
C	-1.879940577	-4.708486709	1.586253295
C	-2.669610527	-4.277101321	0.504001460
C	-2.602043277	-2.957837579	0.035940578
C	-1.956057428	-6.129939908	2.081733339
H	-0.237220483	-1.749755572	2.246013433
H	-0.389537916	-4.106189148	3.053024083
H	-3.339089827	-4.981176458	0.000989969
H	-3.235069749	-2.615626088	-0.787905779
H	-2.436813230	-6.163330472	3.076742550
H	-2.538224655	-6.764577149	1.395699940
H	-0.945655126	-6.559693282	2.194782087
C	3.480034203	-0.126922299	-1.814120348
C	4.229690132	-0.787048776	-2.811807461
C	4.084056245	0.921641538	-1.087195425
C	5.550370037	-0.403013759	-3.072226733
H	3.765179267	-1.599820792	-3.377590010
C	5.404148658	1.303649917	-1.350443186
H	3.517926460	1.432511285	-0.298831653
C	6.142735167	0.642488024	-2.344800011
H	6.121514555	-0.922642608	-3.848675024
H	5.857818379	2.116679933	-0.774538036
H	7.176217769	0.939797198	-2.551070931
Cu	1.200855335	1.467258860	0.632574433
N	0.959099838	3.253778711	1.375523432
C	-0.041079509	3.353745006	0.503365426
C	0.355745597	2.702316415	-0.745353438
H	1.297784474	3.030296136	-1.201759206
H	-0.404901208	2.317994657	-1.432345437
C	-1.402141716	3.833667778	0.780537027
C	-1.881722809	3.917862080	2.106113315
C	-2.243640607	4.205955721	-0.290405341
C	-3.178309352	4.374643560	2.354279317
H	-1.250137154	3.597744271	2.941134356
C	-3.539842285	4.666460142	-0.037294320
H	-1.874061455	4.153200137	-1.319204596
C	-4.007663132	4.750319158	1.283499893
H	-3.549707340	4.428710037	3.382164657
H	-4.186041424	4.961511564	-0.869554279
H	-5.024298825	5.105727547	1.480646754
I	2.170518488	0.188820797	2.587962991
Cu	-1.044412605	-2.948224639	-2.281112668
I	-2.454047726	-4.559433935	-3.453186626
H	0.758462165	3.540766276	2.342359291

I1+ (C-N)

E=-5532.39428

C	1.676896284	1.520237052	1.501402019
N	2.779435394	1.277252416	0.723721733
N	2.413118741	0.578525661	-0.325840427

N	1.064676883	0.374662822	-0.218179669
C	0.543936867	0.929284588	0.906252040
S	0.141141377	-0.493585268	-1.472846247
O	-1.155572284	-0.705762684	-0.793734820
O	0.254791503	0.304060067	-2.701693757
C	1.083122442	-1.970964791	-1.580950177
C	0.959475867	-2.924908051	-0.556155751
C	1.801219693	-4.035712371	-0.589988049
C	2.762244086	-4.198468037	-1.617265465
C	2.858664220	-3.217130179	-2.620626380
C	2.030577374	-2.085149041	-2.613820006
C	3.670005821	-5.401487023	-1.621317826
H	0.226603747	-2.789453610	0.245577553
H	1.717204719	-4.791846160	0.197427245
H	3.600203178	-3.323592093	-3.417568443
H	2.077174573	-1.344240170	-3.417924521
H	3.081407185	-6.335540372	-1.592517024
H	4.314903048	-5.416707178	-2.513702998
H	4.315705767	-5.397128434	-0.724383274
C	1.752921142	2.270539324	2.760196556
C	2.993397668	2.723191294	3.258771346
C	0.579693149	2.547099191	3.493652586
C	3.052917444	3.433255623	4.463750840
H	3.905414428	2.510054737	2.693607167
C	0.642460047	3.255076815	4.698634031
H	-0.387412068	2.203559031	3.109282377
C	1.880331443	3.701249607	5.188843777
H	4.021397194	3.778499866	4.840803288
H	-0.277586053	3.459918258	5.255717128
H	1.930695294	4.255923058	6.131637653
Cu	-1.326146137	0.668291667	1.322834202
N	-2.112905237	2.201995746	0.422424835
C	-3.171435259	1.388441009	0.282005831
C	-3.392234752	0.670100437	1.515770695
H	-3.964577819	-0.261414871	1.530728373
H	-3.351976431	1.245870175	2.448202163
C	-3.867571436	1.098358387	-0.983290411
C	-3.234664019	1.314053425	-2.226855644
C	-5.189240348	0.603630716	-0.950333778
C	-3.916585742	1.042645221	-3.416002444
H	-2.194250290	1.650890967	-2.267620250
C	-5.870810147	0.339172112	-2.143558011
H	-5.687837013	0.448113290	0.011561000
C	-5.235204269	0.557792168	-3.376005192
H	-3.416477391	1.196708113	-4.377008098
H	-6.898507693	-0.035514035	-2.113023233
H	-5.766508028	0.345873344	-4.309459342
I	-1.026202625	-1.299526266	2.824013945
Cu	3.587242906	-0.079630942	-1.666963457
I	5.485625302	-0.410310781	-3.170431273
H	-1.760899744	2.623746171	-0.447752418

TS1 (C-C)

E=-5532.38624

C	-1.735341571	0.265226794	1.440543683
C	-2.860835979	1.025748279	1.046669387
N	-2.640600991	2.339684106	1.358643739
N	-1.456071317	2.461663624	1.906124550
N	-0.890206486	1.212259411	1.949933200
C	-4.083324377	0.560569587	0.392079930
C	-4.390749721	-0.817720855	0.349429855
C	-4.964872649	1.480977136	-0.218388265
C	-5.542688081	-1.267785492	-0.305878734
H	-3.740463253	-1.536697556	0.863648205
C	-6.118493143	1.026081962	-0.865235293
H	-4.729936154	2.548817457	-0.184237333
C	-6.408917597	-0.347911814	-0.916842514
H	-5.769219019	-2.338559793	-0.327709519
H	-6.794259661	1.746768012	-1.337128027
H	-7.311414669	-0.699556815	-1.427412668

S	0.687948185	0.922354202	2.722763728
O	0.785887032	2.024015074	3.692337102
O	0.611390768	-0.503551189	3.091515540
C	1.840471878	1.130880169	1.401496770
C	1.978336717	2.390776384	0.796129691
C	2.634670031	0.032994691	1.037158557
C	2.916213118	2.531990200	-0.227466704
H	1.366097302	3.244114384	1.117913970
C	3.580092939	0.208101048	0.023716371
H	2.519868463	-0.926464189	1.547078771
C	3.727071268	1.447363715	-0.632190383
H	3.028710436	3.506043949	-0.713927610
H	4.209720843	-0.638309329	-0.266101843
C	4.713653786	1.602599259	-1.760815827
H	5.037964072	2.649613511	-1.871140343
H	5.598791807	0.963325404	-1.609413586
H	4.243648965	1.294964350	-2.714056811
Cu	-1.433769378	-1.565089081	1.834759029
I	-2.668332506	-2.102133198	3.963837865
C	-0.029277252	-2.075251992	-0.068244576
C	-0.887291931	-0.830742728	-0.087157468
H	-0.352823214	0.076862488	-0.387322520
H	-1.810428716	-0.965125432	-0.670119705
N	-0.474153750	-2.969451486	0.771244658
C	1.164278489	-2.182851970	-0.930236891
C	1.317205629	-1.323689754	-2.041851429
C	2.162647622	-3.145589034	-0.658127527
C	2.440505052	-1.432233510	-2.868696766
H	0.550528067	-0.578432706	-2.272504514
C	3.289084383	-3.242827795	-1.480028067
H	2.070628951	-3.801739030	0.213324721
C	3.429405499	-2.387569046	-2.587270151
H	2.544983582	-0.768801468	-3.732455224
H	4.064290119	-3.981497596	-1.254941857
H	4.313733085	-2.464654036	-3.227668486
Cu	-0.523261482	4.002322448	2.482962331
I	0.742542574	6.049937977	2.801857461
H	0.046362238	-3.851818723	0.839401529

TS1 (C-N)

E=-5532.37187

C	-0.148951077	-0.190521249	0.322493450
C	0.958770950	-0.176855362	1.209183061
N	1.091529265	-1.418627345	1.767565356
N	0.175864420	-2.216660456	1.271795566
N	-0.574957552	-1.486332452	0.388172988
C	1.877070716	0.913378127	1.525150596
C	1.520890290	2.255153399	1.266126537
C	3.148064701	0.629636181	2.073604462
C	2.421942121	3.292700410	1.534682138
H	0.516778721	2.484370542	0.888949023
C	4.042228527	1.668675859	2.341678088
H	3.426120123	-0.410375854	2.264638934
C	3.685088274	3.001766427	2.069292435
H	2.131499431	4.328521087	1.333081244
H	5.028530259	1.440685122	2.758391469
H	4.391182641	3.811642196	2.279800128
S	-1.840370616	-2.283307387	-0.591815610
O	-1.333443582	-2.212375982	-1.977085282
O	-2.007582468	-3.565721674	0.116269659
C	-3.191241625	-1.182646052	-0.381312865
C	-3.578145857	-0.378288019	-1.470039009
C	-3.861904847	-1.159847069	0.853727227
C	-4.669812464	0.476056693	-1.297971749
H	-3.053043569	-0.443504125	-2.426637549
C	-4.941884590	-0.288703824	0.994239444
H	-3.543543446	-1.803861166	1.678163209
C	-5.361504615	0.540427115	-0.070473190
H	-4.985121475	1.109438720	-2.132386300
H	-5.472778957	-0.249952494	1.950313066

C	-6.496309708	1.510693213	0.117685288
H	-7.203262326	1.161819085	0.887170511
H	-6.087769580	2.483474957	0.452271312
H	-7.039130328	1.683770598	-0.825604210
Cu	-1.232720925	1.292778922	-0.388779806
I	-2.597029957	2.919875591	0.831691485
C	1.372584117	0.830564517	-1.701427667
C	1.643363102	2.159015322	-1.833320831
H	2.662574211	2.536282321	-1.937582821
H	0.822356898	2.882239703	-1.858651648
N	0.015436228	0.475159377	-1.586639986
C	2.404193589	-0.235150686	-1.588353700
C	3.744982735	0.094102862	-1.291289007
C	2.058214118	-1.600323701	-1.694446629
C	4.703246510	-0.906711239	-1.103732156
H	4.035615980	1.140513449	-1.165534073
C	3.016859129	-2.602285867	-1.501722832
H	1.032127230	-1.904930027	-1.922921970
C	4.343410394	-2.260106097	-1.203189566
H	5.733420674	-0.628483252	-0.859357138
H	2.721972271	-3.653486764	-1.580506774
H	5.091781545	-3.043030915	-1.043240027
Cu	-0.183472353	-4.029281337	1.682962788
I	-0.224551963	-6.318476606	2.508012575
H	-0.189165291	-0.472894790	-1.933089641

I2 (C-C)

E=-3594.179214829

C	-1.503327513	-0.551720144	1.232067822
C	-2.688099721	-0.152084849	0.594220901
N	-2.974301989	-1.038971261	-0.408625389
N	-2.057283089	-1.971157578	-0.419426885
N	-1.152018773	-1.698044031	0.566189751
C	-3.516693890	1.042099370	0.822068585
C	-3.762878120	1.531186238	2.123207946
C	-4.075015805	1.716633658	-0.286222104
C	-4.536271327	2.684816817	2.308455311
H	-3.377765170	0.990995732	2.993652172
C	-4.851322232	2.865168774	-0.094047346
H	-3.887959789	1.334251160	-1.294219106
C	-5.079319402	3.355299187	1.202073293
H	-4.725528506	3.052438599	3.322080845
H	-5.277099132	3.382948167	-0.959701856
H	-5.684935417	4.255380566	1.349473453
C	0.773760355	0.393123099	1.892685053
C	-0.679323725	0.101322875	2.298299910
H	-0.669882873	-0.518790839	3.209589484
N	1.778450077	0.012338047	2.600669159
C	1.016173471	1.147854630	0.630780131
C	-0.010288012	1.842574269	-0.044188894
C	2.318819755	1.167910900	0.086584330
C	0.252871525	2.510355363	-1.247653696
H	-1.021527130	1.887861612	0.368093096
C	2.579178929	1.827865552	-1.116726229
H	3.111625036	0.646408333	0.627699540
C	1.544235449	2.494718600	-1.794066819
H	-0.555378679	3.044830852	-1.757305877
H	3.591861022	1.822304189	-1.533143614
H	1.746440288	3.006613197	-2.740691191
H	-1.172902383	1.053495551	2.554782178
S	0.134322327	-2.937163505	0.889998356
O	0.394435083	-2.850433793	2.330875427
O	-0.462914042	-4.129677474	0.259195064
C	1.499797077	-2.359556756	-0.067148192
C	2.752757709	-2.248334551	0.554039332
C	1.316086144	-2.134791548	-1.440000057
C	3.846040002	-1.890335334	-0.237845865
H	2.862616888	-2.430246309	1.624994945
C	2.421986606	-1.755026456	-2.201286095
H	0.329892453	-2.252364030	-1.909160334

C	3.699982223	-1.628698304	-1.616856260
H	4.831415365	-1.797956784	0.230460782
H	2.290479117	-1.558208189	-3.269758342
C	4.891048515	-1.211854161	-2.441113034
H	4.615055244	-1.045961497	-3.494045111
H	5.329486778	-0.279473901	-2.042331071
H	5.681479117	-1.982730265	-2.402139241
Cu	-1.822504814	-3.399921649	-1.657355665
I	-1.524130166	-4.813435572	-3.611569836
H	1.458375145	-0.533856557	3.414391242

I2 (C-N)

E=-3594.17081

C	0.005910566	0.806705130	-0.164460093
C	-1.064174821	0.185640335	-0.847317815
N	-0.701889028	-1.098891777	-1.130158449
N	0.512551865	-1.325793091	-0.698820857
N	0.982403144	-0.163353901	-0.123428628
C	-2.369332284	0.741584129	-1.232136253
C	-3.535740241	-0.037295780	-1.088997951
C	-2.470555231	2.055626241	-1.736476466
C	-4.783773757	0.497681449	-1.427764179
H	-3.455771546	-1.052827646	-0.690331938
C	-3.721812537	2.585446028	-2.073147316
H	-1.567341779	2.659560568	-1.864698625
C	-4.881339427	1.810917349	-1.914485879
H	-5.686421111	-0.108930298	-1.300922844
H	-3.791317736	3.607050039	-2.460869784
H	-5.860151691	2.229589078	-2.171128248
S	2.374932075	-0.195438280	0.989573153
O	2.924764691	1.166179923	0.849109847
O	1.883289719	-0.689716192	2.282926009
C	3.396592950	-1.406870031	0.205996309
C	3.840631444	-2.495356526	0.980292134
C	3.908466733	-1.143875450	-1.082693761
C	4.804842643	-3.345074346	0.433230142
H	3.434706151	-2.662280538	1.981169577
C	4.856665763	-2.023357072	-1.605304969
H	3.571382902	-0.274019430	-1.653711164
C	5.324356422	-3.128745094	-0.859048067
H	5.153591775	-4.199854571	1.020558366
H	5.246677356	-1.846471109	-2.612326308
C	6.319887683	-4.085181594	-1.457238999
H	5.774891392	-4.864715200	-2.022876215
H	6.998847072	-3.573603743	-2.158916250
H	6.913774594	-4.589873969	-0.678313322
C	-0.905896656	2.799740562	0.967011594
C	-0.814162223	4.149517847	0.966845729
H	-1.560027751	4.752879661	1.489905653
H	0.025249270	4.662110051	0.486022001
N	0.156253924	2.021719550	0.435808728
C	-2.036578176	2.044025256	1.566496535
C	-1.808578894	0.827949292	2.246815576
C	-3.356532368	2.526592396	1.454266219
C	-2.880264052	0.107190829	2.787575935
H	-0.787292225	0.450615162	2.363718734
C	-4.424914905	1.806515593	1.999017918
H	-3.543363510	3.449556999	0.897885055
C	-4.191613836	0.590355484	2.660050622
H	-2.689722027	-0.835223050	3.311525998
H	-5.446463409	2.183934502	1.887059335
H	-5.029874655	0.018131947	3.071229762
H	1.112732308	2.365833744	0.544500838
Cu	1.588916279	-2.864383054	-1.029551587
I	2.252587154	-5.091517400	-1.793465801

Imine product

E=-1655.896024

C	0.337146311	-0.286606330	1.061272997
C	0.909584217	0.929708782	0.673142592

N	-0.065982386	1.695207988	0.071444929
N	-1.201624113	1.052921506	0.064656134
N	-0.971751314	-0.146158899	0.664357529
C	2.306727314	1.380518844	0.747891456
C	2.828049727	2.184102024	-0.289372339
C	3.150926581	1.002914151	1.812776353
C	4.170503086	2.576913711	-0.273916256
H	2.171302427	2.476221968	-1.114103048
C	4.496168102	1.394250963	1.821786671
H	2.758345193	0.402639393	2.637738628
C	5.011624293	2.176631708	0.777504524
H	4.566303535	3.190163246	-1.090383961
H	5.142886566	1.088637019	2.650944772
H	6.064631468	2.477582181	0.784245406
C	2.278932552	-1.919729597	1.109426717
C	0.895653000	-1.552380525	1.644853466
H	0.206761235	-2.386975986	1.424860736
N	3.134511523	-2.370482959	1.958968670
C	2.532710246	-1.726690808	-0.347903894
C	3.819635907	-1.354811873	-0.788988979
C	1.497803751	-1.870410846	-1.296987750
C	4.069950115	-1.135821647	-2.147880316
H	4.615801439	-1.193782732	-0.054588897
C	1.754074955	-1.664639252	-2.658303422
H	0.489262354	-2.151514446	-0.977264474
C	3.037957814	-1.292738212	-3.086644374
H	5.068460019	-0.827456215	-2.474348999
H	0.946046109	-1.788112252	-3.386949547
H	3.231809663	-1.117004832	-4.150130285
H	4.009576395	-2.626989615	1.471955838
H	0.967071011	-1.495353830	2.743625035
S	-2.249193455	-1.402511394	0.762768142
O	-1.923175705	-2.369974954	-0.300874500
O	-2.267741976	-1.807070007	2.177664852
C	-3.683711254	-0.467010805	0.344420551
C	-4.392173573	0.168581966	1.375411045
C	-4.080658181	-0.392800844	-0.998948926
C	-5.535363115	0.896966381	1.038554447
H	-4.057334867	0.084117593	2.412570454
C	-5.228151965	0.341534376	-1.307773516
H	-3.507227502	-0.904933246	-1.776003321
C	-5.968337945	0.999257219	-0.301318771
H	-6.105631013	1.392694901	1.830847090
H	-5.557728284	0.404489529	-2.349882485
C	-7.186644513	1.815277060	-0.652898688
H	-7.911067227	1.833013956	0.177838134
H	-6.893201240	2.861911733	-0.859829767
H	-7.683517279	1.425703907	-1.556230654

Enamine product

E=-1655.902772

C	0.192941198	0.456488887	0.786272585
C	0.638778047	-0.876608735	0.723546428
N	-0.398512959	-1.669865402	0.293886295
N	-1.472157761	-0.954071543	0.101204949
N	-1.135491054	0.337038798	0.419709403
C	1.970310596	-1.422912497	1.028047771
C	2.526167271	-2.422669407	0.204157809
C	2.713528924	-0.953999321	2.132286503
C	3.803617467	-2.930899645	0.470539332
H	1.953535591	-2.782436804	-0.655924508
C	3.989087105	-1.465760336	2.396426901
H	2.285589619	-0.182969376	2.780788818
C	4.540972903	-2.452268632	1.563797355
H	4.229892188	-3.697992744	-0.184795360
H	4.555702473	-1.092603982	3.256520522
H	5.542559110	-2.846064503	1.767447704
C	2.104717251	2.035588201	0.647100943
C	0.840439437	1.716204922	1.090713696
H	0.301251507	2.422899075	1.730844305

N	2.823740228	3.081512767	1.175675058
C	2.800158760	1.255610932	-0.411367543
C	2.104689961	0.870588798	-1.576676658
C	4.155889733	0.897832972	-0.268447610
C	2.748465553	0.117866895	-2.565237912
H	1.060257845	1.173633925	-1.702255593
C	4.795628391	0.140824474	-1.257751122
H	4.693654298	1.186221603	0.639741494
C	4.092923220	-0.254151723	-2.405462006
H	2.200314932	-0.176392607	-3.466548315
H	5.842304468	-0.151989263	-1.125619903
H	4.592151724	-0.850785059	-3.176413781
H	3.494393630	3.546905844	0.564350601
H	2.346984444	3.704547223	1.827286262
S	-2.185183456	1.695584101	-0.021569939
O	-2.209242560	2.574945505	1.160494892
O	-1.689284006	2.183870049	-1.320708163
C	-3.742255402	0.879492037	-0.219694544
C	-4.106134442	0.404952305	-1.487906125
C	-4.579556574	0.750165284	0.897557473
C	-5.348853103	-0.217527484	-1.630370773
H	-3.431312617	0.528963055	-2.338711397
C	-5.817092625	0.123084351	0.728840629
H	-4.269084147	1.141951414	1.869685635
C	-6.219305178	-0.372386619	-0.530056240
H	-5.651114169	-0.588604638	-2.615208451
H	-6.486602601	0.021050021	1.589336509
C	-7.544930351	-1.074410687	-0.691803771
H	-7.945081124	-0.946605116	-1.711085688
H	-7.422797662	-2.160245353	-0.517039135
H	-8.286656082	-0.703601964	0.034394666

1 CuI, R= CF_2CF_3 (Figure 1)

R

E=-2985.898308

C	-0.155607232	-0.340278246	0.624109951
N	0.315623634	-1.179946762	1.613827417
N	1.547382582	-0.893114050	1.896325854
N	1.873544157	0.175733845	1.072139670
C	0.851756363	0.564166797	0.226827765
C	-1.532209979	-0.449148551	0.122789377
C	-2.019652615	0.434794845	-0.866336467
C	-2.403994698	-1.444114820	0.620745114
C	-3.330610899	0.327519889	-1.342991712
H	-1.352212436	1.215130326	-1.258332496
C	-3.716059767	-1.549314090	0.142729429
H	-2.033453929	-2.131558625	1.387432154
C	-4.188284100	-0.665673382	-0.841514586
H	-3.684730878	1.025445690	-2.109849814
H	-4.375722446	-2.327960081	0.542034432
H	-5.214996649	-0.749234508	-1.213882049
C	3.218901161	0.664028594	1.067068272
C	4.124112754	-0.031312388	0.009808926
F	3.785666816	0.463062744	2.278834559
F	3.230438078	1.995875151	0.800632319
F	5.377482070	0.449797393	0.084691243
F	4.157286349	-1.354856518	0.234211052
F	3.655806667	0.185057096	-1.230195542
Cu	0.934920516	1.929946688	-1.066334128
I	0.934614482	3.685952965	-2.794770740

TS0 (C-C)

E=-3349.577606

C	0.348132555	-0.310842316	-2.370615068
C	0.106766244	0.922034922	-3.012261235
N	-0.592972328	0.727996480	-4.188158251

N	-0.824565340	-0.533428381	-4.368361247
N	-0.244733456	-1.169479138	-3.274464009
C	0.482000090	2.269635589	-2.566544155
C	1.448207769	2.453525120	-1.551822804
C	-0.118753484	3.412996256	-3.141447296
C	1.787742733	3.739092287	-1.112953070
H	1.948942666	1.574043793	-1.127688414
C	0.226598483	4.697376870	-2.703040038
H	-0.861564601	3.275204099	-3.933343627
C	1.177625139	4.868766888	-1.682950289
H	2.542339739	3.858556483	-0.327310308
H	-0.252566333	5.571762547	-3.158026357
H	1.444325232	5.874356905	-1.339686514
Cu	1.116886652	-0.855639160	-0.659950770
I	3.829295811	-0.735137019	-0.564147503
C	0.251345860	-0.993542004	1.965194827
C	0.200543592	0.124022090	1.033780618
H	-0.739482517	0.346273635	0.515467409
H	0.977185023	0.898138345	1.079823223
N	0.768087111	-1.685990360	1.014797780
C	-0.139351515	-1.286567291	3.334496309
C	-0.762744817	-0.284544216	4.108807349
C	0.097910117	-2.561594638	3.896614570
C	-1.146856448	-0.554763496	5.427647739
H	-0.943859517	0.701224909	3.666807718
C	-0.288010850	-2.824678455	5.213741885
H	0.586331853	-3.331256612	3.290067521
C	-0.910731880	-1.823399181	5.981332872
H	-1.631425127	0.223744195	6.025954286
H	-0.103852492	-3.812235564	5.649452690
H	-1.210471253	-2.034115952	7.013458927
C	-0.464648736	-2.570940332	-3.097519305
C	-1.734700150	-2.892950559	-2.256507315
F	0.596138640	-3.131571367	-2.458867075
F	-0.613886304	-3.172468828	-4.299188049
F	-2.821797205	-2.373360048	-2.850588016
F	-1.896641396	-4.223736263	-2.150966366
F	-1.632789558	-2.376510235	-1.021038643

TS0 (C-N)

E=-3349.572102

C	0.014227582	0.179819967	-2.268297681
C	-0.490338029	1.402407644	-2.755393907
N	-1.342554248	1.186437009	-3.822476117
N	-1.431632745	-0.080100529	-4.074656008
N	-0.594448168	-0.694176607	-3.142885399
C	-0.209722865	2.757039919	-2.263885417
C	0.789664034	2.981228919	-1.289992624
C	-0.932585357	3.869284405	-2.753116217
C	1.050550405	4.271375173	-0.814358151
H	1.377004393	2.131438628	-0.921791405
C	-0.668438058	5.159449698	-2.276711548
H	-1.703884945	3.701901988	-3.511213831
C	0.322318843	5.368990855	-1.302865454
H	1.833062095	4.420450008	-0.061979741
H	-1.241385801	6.008323014	-2.666806264
H	0.527502653	6.378740045	-0.930839665
Cu	1.220057215	-0.324214440	-0.767773414
I	3.754059918	0.022449617	-1.546875096
C	0.744573371	-1.245919331	1.722160151
C	1.523446799	-1.853103859	0.645300427
H	2.617422354	-1.811573607	0.698551633
H	1.053127827	-2.613939045	0.007930590
N	0.544736718	-0.186478604	1.020584201
C	0.372158686	-1.611167496	3.083002062
C	0.803951578	-2.845760650	3.612334599
C	-0.411098922	-0.743296149	3.877138475
C	0.460400231	-3.207329154	4.920885480
H	1.411519224	-3.513155599	2.991302237
C	-0.750574973	-1.109298821	5.182675284

H	-0.744896689	0.211090391	3.456848331
C	-0.315822790	-2.340565074	5.706966159
H	0.798567706	-4.165158512	5.329516886
H	-1.356570159	-0.436419647	5.798432129
H	-0.584044087	-2.623398766	6.730401377
C	-0.583262063	-2.120906361	-3.069092679
C	-1.677086428	-2.698077842	-2.123906986
F	0.619508347	-2.555864954	-2.607594876
F	-0.789818385	-2.653341242	-4.295813950
F	-2.893528181	-2.304166371	-2.527099819
F	-1.634625121	-4.042438717	-2.126532256
F	-1.481541964	-2.270575900	-0.862071516

I1 (C-C)

E=-3349.585262

C	-0.017501216	2.086465333	0.758503037
C	-1.345142311	2.519591682	0.597370216
N	-1.362365084	3.826254575	0.152040302
N	-0.151867653	4.267966154	0.010659429
N	0.672823128	3.210793164	0.378612659
C	-2.579998672	1.767475126	0.842886907
C	-2.540387572	0.469073188	1.399590769
C	-3.839474763	2.325421253	0.525769834
C	-3.719078421	-0.251231902	1.624931620
H	-1.572515720	0.030510404	1.674501460
C	-5.017409672	1.604478419	0.755618942
H	-3.875743569	3.331381937	0.096684268
C	-4.964993714	0.311908221	1.304013884
H	-3.662892192	-1.254772383	2.060216388
H	-5.984493404	2.053985087	0.503993644
H	-5.887670736	-0.250353986	1.483140459
Cu	0.759591101	0.390005989	1.289540112
I	0.873462703	0.989919171	3.850189285
C	1.293795170	-1.678562452	0.090719952
C	0.756515008	-0.436000677	-0.528599679
H	1.439180396	0.165327383	-1.144758547
H	-0.285481653	-0.440311413	-0.879519613
N	1.430548980	-1.412327162	1.349934133
C	1.606124892	-2.964038272	-0.570316588
C	1.412224004	-3.109100456	-1.958318433
C	2.094126672	-4.055646606	0.179861925
C	1.700098125	-4.327503119	-2.589430194
H	1.034002459	-2.260343417	-2.539363397
C	2.381852470	-5.269774527	-0.450762919
H	2.238631696	-3.928131337	1.258246759
C	2.184612310	-5.408785850	-1.837073879
H	1.546397704	-4.434846208	-3.668599513
H	2.760040740	-6.114845444	0.134657516
H	2.408968101	-6.361586356	-2.328907463
C	2.090283299	3.391017079	0.296682584
C	2.612634083	3.575186888	-1.158634602
F	2.712744265	2.300617708	0.805886975
F	2.471874941	4.481105700	1.007807607
F	2.161270528	4.714025993	-1.696900642
F	3.956677340	3.602629307	-1.157187202
F	2.208536235	2.543021806	-1.923687997

I1 (C-N)

E=-3349.579463

C	-0.480596330	1.352290495	-1.391773889
C	-1.221172376	2.482687472	-0.999986372
N	-2.099298427	2.848712501	-2.001492399
N	-1.990528057	2.037223041	-3.008234619
N	-1.017237927	1.118718658	-2.634735337
C	-1.143415304	3.221213740	0.266245376
C	-0.378798702	2.726086105	1.346545588
C	-1.833317926	4.443145829	0.436287136
C	-0.299738361	3.429970337	2.553657174
H	0.154343340	1.774133405	1.231536928
C	-1.755257682	5.145885836	1.645491162

H	-2.429323806	4.832446568	-0.394929576
C	-0.987188691	4.645323509	2.710203241
H	0.299194796	3.026406710	3.377348720
H	-2.296165644	6.092023350	1.757175188
H	-0.926759883	5.196383595	3.654725219
Cu	0.914385411	0.273686978	-0.496118392
I	2.857176142	1.963127702	-0.939401818
C	0.835813410	-1.908046009	0.654766562
C	2.029658775	-1.031967197	0.518631836
H	2.372791315	-0.527838516	1.434060007
H	2.839854968	-1.380672724	-0.139373544
N	-0.129161544	-1.235357677	0.116133453
C	0.736200364	-3.256637413	1.247816162
C	1.872851729	-3.839232342	1.843942324
C	-0.479327410	-3.975151470	1.218216615
C	1.796945317	-5.121736662	2.405115352
H	2.815007724	-3.280108589	1.865319758
C	-0.552887832	-5.254580424	1.777811790
H	-1.355603441	-3.515875084	0.747808598
C	0.585400040	-5.830288867	2.372336418
H	2.682611545	-5.569781768	2.867963870
H	-1.495856188	-5.810927811	1.751334055
H	0.526328329	-6.833106650	2.808558174
C	-0.641419637	0.107764351	-3.579508140
C	0.271463191	0.627661091	-4.726721692
F	-1.749337331	-0.425410167	-4.150163962
F	0.035885190	-0.877370989	-2.942490300
F	-0.361815937	1.564685219	-5.448398769
F	0.604487677	-0.391226288	-5.538570421
F	1.393809174	1.155740155	-4.217131480

I1+ (C-C)

E=-3350.069787

C	0.438723051	2.111606853	0.635977027
C	-0.938745383	2.118985315	0.393127267
N	-1.337663067	3.415444389	0.143283302
N	-0.321016691	4.221098483	0.206613134
N	0.771788786	3.435643266	0.512118723
C	-1.852297017	0.972914181	0.339180034
C	-1.498566920	-0.257381074	0.934492565
C	-3.084276607	1.068457664	-0.344308244
C	-2.336900574	-1.372317512	0.825449367
H	-0.569926933	-0.331014528	1.515933619
C	-3.925155265	-0.046211396	-0.444234299
H	-3.364252514	2.021657107	-0.802828727
C	-3.551792394	-1.272308276	0.131156495
H	-2.039953137	-2.319104818	1.286572365
H	-4.875068560	0.039420358	-0.982598221
H	-4.208285688	-2.144275681	0.043256909
Cu	1.619379878	0.691661877	1.053950867
I	1.633582541	0.821989728	3.576562590
C	1.760899257	-1.162195986	-0.172969058
C	1.528231985	0.085132767	-0.891437262
H	2.412001304	0.650344387	-1.210446847
H	0.611200862	0.240635121	-1.465804634
N	2.657279421	-0.946574323	0.795418990
C	0.934450707	-2.369552496	-0.341023673
C	0.205457458	-2.565237637	-1.533979882
C	0.825825333	-3.315389485	0.702347754
C	-0.618119855	-3.685651779	-1.679078241
H	0.290402346	-1.842785479	-2.351347861
C	0.003058269	-4.434754273	0.552676860
H	1.358287265	-3.161398699	1.646702703
C	-0.721649454	-4.619701651	-0.636824669
H	-1.182332663	-3.830202641	-2.605238741
H	-0.085948276	-5.158026201	1.369106945
H	-1.372769810	-5.492731803	-0.748189907
C	2.082895376	4.026055182	0.532628835
C	2.610925894	4.391586980	-0.886303264
F	2.952646837	3.127361327	1.061185667

F	2.081484579	5.139888665	1.287360236
F	1.849234398	5.332049232	-1.454005779
F	3.870725990	4.839836295	-0.799526957
F	2.595083961	3.289454157	-1.661656465
H	2.791155311	-1.714407598	1.466700477

I1+ (C-N)

E=-3350.062848

C	-0.377709232	-0.087141460	-2.109632731
C	0.107463472	1.180274275	-1.763328663
N	-0.282524441	2.090532679	-2.723630532
N	-0.978305248	1.496450311	-3.647062070
N	-1.054815331	0.170636063	-3.270762741
C	0.881561841	1.551301728	-0.571255570
C	0.890484986	2.880489528	-0.096620724
C	1.614943335	0.572982514	0.135384448
C	1.605311192	3.212709437	1.060979834
H	0.326995739	3.643508364	-0.642349798
C	2.325770050	0.906859922	1.293076557
H	1.647946297	-0.454964316	-0.246824644
C	2.320264420	2.228465242	1.763006208
H	1.599645999	4.246800439	1.421848036
H	2.883375990	0.132306086	1.829310059
H	2.868916983	2.490904828	2.673360580
Cu	-0.366269292	-1.685573982	-1.031415619
I	1.516673403	-3.007774214	-1.983201635
C	-1.070108332	-1.655343438	1.073946322
C	-0.547491054	-2.927612078	0.641465257
H	0.325514381	-3.382522699	1.116102003
H	-1.239293565	-3.601321589	0.122358891
N	-1.841673571	-1.143515720	0.092551753
C	-0.629477888	-0.922210482	2.274685926
C	-0.897670875	0.457959422	2.408422119
C	0.081645769	-1.589807244	3.294890918
C	-0.460544665	1.155695207	3.535471895
H	-1.420411625	1.002541526	1.616113658
C	0.525266424	-0.885808975	4.419643372
H	0.279790769	-2.662788988	3.214203914
C	0.254075934	0.485658742	4.541883093
H	-0.660416635	2.227549278	3.621641738
H	1.079125661	-1.409321768	5.205022500
H	0.604483909	1.035685387	5.421203486
C	-1.741600547	-0.765104122	-4.119922881
C	-0.967949497	-1.082181152	-5.432572966
F	-2.955981008	-0.279803316	-4.457003279
F	-1.904920544	-1.927005007	-3.441705532
F	-0.938778583	-0.005086880	-6.227938515
F	-1.577172369	-2.086321518	-6.082155269
F	0.289381462	-1.448426281	-5.143834163
H	-2.115523712	-0.159675749	0.214644765

TS1 (C-C)

E=-3350.058236

C	0.092626521	-0.279039983	1.482981648
C	0.270431687	-1.560305245	2.031841018
N	-0.801827778	-1.843673746	2.847963043
N	-1.643212562	-0.855269301	2.851323869
N	-1.122747888	0.104128634	2.012512622
C	1.375033742	-2.494632158	1.818615089
C	2.612181007	-2.045087114	1.305524622
C	1.216533582	-3.866256288	2.118352704
C	3.657410661	-2.946152863	1.073359729
H	2.772390907	-0.973123026	1.124265659
C	2.266972389	-4.762232441	1.894119538
H	0.261188103	-4.214284775	2.521947277
C	3.487322143	-4.308364136	1.365387094
H	4.610283475	-2.579379498	0.678702637
H	2.132766833	-5.823256402	2.129005752
H	4.305898620	-5.013713803	1.189283925
Cu	1.399067339	0.934434419	0.784735657

I	2.596760103	2.053037495	2.679569358
C	0.605035693	1.040621722	-1.364879164
C	0.226924259	-0.177496027	-0.575200799
H	-0.826405972	-0.462807369	-0.614882751
H	0.906119751	-1.027628313	-0.722794652
N	1.833501084	1.442381294	-1.104059633
C	-0.361341371	1.718718107	-2.253898535
C	-1.483586017	1.026522745	-2.761813246
C	-0.154716802	3.064734852	-2.634036463
C	-2.371315953	1.663052657	-3.635868844
H	-1.653743248	-0.020256340	-2.495330026
C	-1.046253062	3.698878755	-3.503089336
H	0.694126308	3.625769341	-2.229259951
C	-2.155897483	2.998992966	-4.006556190
H	-3.232483740	1.114642866	-4.029842736
H	-0.882098722	4.743893972	-3.783645787
H	-2.854374724	3.497919474	-4.686120772
C	-1.807457751	1.358445897	1.859787855
C	-3.206701979	1.217158112	1.189388136
F	-1.054642306	2.153401857	1.053307005
F	-1.963801678	1.966054264	3.051178509
F	-4.041341806	0.514948403	1.957999852
F	-3.723681819	2.432842284	0.971981675
F	-3.065867291	0.587629051	0.004481401
H	2.130925745	2.294749661	-1.596336789

TS1 (C-N)

E=-3350.040957

C	-0.692996039	-0.375173880	-1.271801604
C	0.675842657	-0.387800563	-0.928137448
N	1.308479559	0.621593052	-1.620751262
N	0.460222105	1.277941129	-2.347811077
N	-0.775652298	0.709211248	-2.123280123
C	1.392821348	-1.267770061	-0.014100834
C	0.834204511	-2.499358091	0.397069119
C	2.661395453	-0.890170189	0.483284324
C	1.516074327	-3.326693055	1.297036026
H	-0.121453194	-2.832955644	-0.029555610
C	3.339335858	-1.719547212	1.380038536
H	3.091399915	0.063936613	0.166714129
C	2.768053018	-2.936712059	1.794177518
H	1.071814892	-4.279660068	1.600834351
H	4.317969667	-1.415557747	1.765167950
H	3.303732143	-3.582212600	2.497803898
Cu	-2.008000353	-1.817943659	-1.041148396
I	-2.332852241	-3.919762779	-2.243743533
C	-1.317565020	-0.178772106	1.362574654
C	-1.264843552	-1.225949720	2.229062000
H	-0.716820203	-1.167175089	3.172113792
H	-1.807009353	-2.148355119	2.002376416
N	-2.069773906	-0.362912191	0.186186731
C	-0.570656727	1.095462052	1.532553108
C	0.588503300	1.141680391	2.337696674
C	-0.961683689	2.267752618	0.850112104
C	1.331779298	2.321077739	2.452507327
H	0.931964760	0.237454279	2.847682892
C	-0.216679075	3.447483322	0.963453332
H	-1.859640188	2.278578077	0.222472942
C	0.934763457	3.477762403	1.763536375
H	2.235494220	2.332266595	3.070260198
H	-0.537198640	4.343674618	0.423093105
H	1.522607423	4.397490879	1.846421271
C	-1.895826437	1.135558577	-2.921985776
C	-1.932701669	0.499797396	-4.343819390
F	-1.873599213	2.472057766	-3.044401653
F	-3.035683473	0.764413588	-2.272904128
F	-1.949023285	-0.836405285	-4.233515211
F	-3.035023171	0.901343535	-4.990507203
F	-0.852366266	0.871293653	-5.040387476
H	-2.459409921	0.513057584	-0.176378050

TS1’ (C-C) *no proton source*

E=-3349.551106

C	-0.044491443	-0.049058884	1.783014534
C	0.196983033	-1.416239523	2.065438225
N	-0.743575836	-1.868584528	2.956654296
N	-1.574354310	-0.922567360	3.272074101
N	-1.173438644	0.195450625	2.553562954
C	1.250600481	-2.269621737	1.527972471
C	2.326206361	-1.718175441	0.793330164
C	1.212629354	-3.670000441	1.726931240
C	3.321652726	-2.543381663	0.257190820
H	2.395597157	-0.629685364	0.661468683
C	2.215832637	-4.488855132	1.199673491
H	0.384109662	-4.098196816	2.299080967
C	3.272391533	-3.931681615	0.457865774
H	4.145891685	-2.095921953	-0.308179453
H	2.173396460	-5.570966322	1.364554084
H	4.055386198	-4.576134644	0.044452977
Cu	1.161950621	1.310026042	1.219396442
I	2.668757663	1.724511445	3.230174997
C	0.416284986	1.530057387	-0.978345434
C	-0.224542300	0.419251829	-0.101224672
H	-1.313397394	0.524081055	-0.011623580
H	0.031406301	-0.586045023	-0.467186676
N	1.392035049	2.227413052	-0.512927644
C	-0.152075879	1.693545275	-2.363999039
C	-1.221370850	0.913428726	-2.850015166
C	0.413137851	2.671943612	-3.209409673
C	-1.714580661	1.109092340	-4.149558673
H	-1.677684377	0.144008174	-2.218387924
C	-0.076826255	2.867248877	-4.504014896
H	1.242843964	3.264490534	-2.807630008
C	-1.145285967	2.085175654	-4.980073356
H	-2.546620249	0.495925024	-4.513319056
H	0.371674255	3.630892984	-5.149734893
H	-1.530776899	2.237637753	-5.994167690
C	-1.840202216	1.441124227	2.763014198
C	-3.332746796	1.433989293	2.317605389
F	-1.209864085	2.398964571	2.035188626
F	-1.806316940	1.797125813	4.070507100
F	-4.042146367	0.531501640	3.003011829
F	-3.869236038	2.648136899	2.513796927
F	-3.409234473	1.140093616	1.003837544

TS1’ (C-N) *no proton source*

E= -3349.540605

C	-0.861322121	-0.516590249	-1.494680023
C	0.553147166	-0.574377891	-1.552527874
N	0.964397475	-0.214199118	-2.815312847
N	-0.050334491	0.041763291	-3.580168775
N	-1.183019863	-0.172041866	-2.790161628
C	1.498354064	-0.894086647	-0.484995386
C	1.061612148	-1.494661063	0.716562315
C	2.867218990	-0.563432870	-0.613795106
C	1.951453363	-1.713732728	1.772935945
H	0.009698063	-1.788289172	0.818223854
C	3.757381845	-0.795627449	0.439710845
H	3.211914328	-0.100773305	-1.543786182
C	3.303748542	-1.361504642	1.643480294
H	1.584608961	-2.162812534	2.701991663
H	4.812559848	-0.521706601	0.326946164
H	4.000130484	-1.528393103	2.472392550
Cu	-2.121088386	-1.269512320	-0.199532249
I	-2.377817975	-3.766190736	-0.159983392
C	-1.681657419	0.475182513	1.093815358
C	-2.590914175	-0.405732540	1.731175729
H	-2.420070696	-0.834431856	2.726585405
H	-3.611702746	-0.475911211	1.333531076
N	-1.848320297	0.640815184	-0.270131152

C	-0.540530399	1.125876342	1.791860944
C	-0.397083163	1.102550085	3.195629933
C	0.450181332	1.764386414	1.016596231
C	0.732002406	1.665395881	3.802781002
H	-1.171206065	0.639253042	3.816312652
C	1.572209354	2.336509353	1.623947323
H	0.321871773	1.771241027	-0.069781117
C	1.722690445	2.279793799	3.018932160
H	0.839129427	1.629663649	4.892659958
H	2.341678061	2.813947611	1.007799145
H	2.608088874	2.715009086	3.495411116
C	-2.478073004	0.088698125	-3.306893231
C	-2.592829998	-0.094164490	-4.851042466
F	-2.893424822	1.361492333	-3.037130276
F	-3.378244564	-0.757067920	-2.736918508
F	-2.115080669	-1.286964497	-5.234331322
F	-3.898198011	-0.035946913	-5.183225676
F	-1.953158086	0.876573984	-5.514884451

2CuI, R= CF_2CF_3 (Figure 4)

RC’

E=-5287.9002606

C	-2.335536445	0.312774792	0.212233336
C	-1.908960538	-0.614485174	1.196798693
N	-2.998802574	-1.216116220	1.786204330
N	-4.096807132	-0.724913382	1.291633226
N	-3.717181966	0.191878991	0.344321077
C	-0.539300142	-0.934402806	1.619816963
C	-0.260359294	-2.145699422	2.290901516
C	0.520549057	-0.032680454	1.381440894
C	1.046467602	-2.451512516	2.691112853
H	-1.080568093	-2.843862205	2.483476063
C	1.825242697	-0.340944884	1.779827796
H	0.310578860	0.915391202	0.868559558
C	2.095877630	-1.554307180	2.433068539
H	1.248180707	-3.399092478	3.202244052
H	2.633587350	0.368907728	1.577008843
H	3.118185991	-1.797184203	2.741789448
C	1.538196219	-1.132550041	-1.494936080
C	0.996068640	-2.478213220	-1.333241241
H	0.994574911	-3.185050943	-2.174791054
H	0.899715574	-2.909785947	-0.327045078
N	0.262492519	-1.158645857	-1.559578764
Cu	-1.491883976	-0.362661673	-1.558138444
Cu	-1.469489454	1.854442911	-0.545033132
C	2.619952320	-0.185677086	-1.497626736
C	3.959870989	-0.615599177	-1.379081921
C	2.318181695	1.194296594	-1.564633899
C	4.987504652	0.332744101	-1.330661115
H	4.179532883	-1.686043319	-1.317951335
C	3.349053055	2.133261597	-1.509658009
H	1.273024469	1.516901865	-1.625001820
C	4.684254125	1.703841722	-1.395498159
H	6.028200670	0.006369680	-1.237297789
H	3.110203754	3.201049264	-1.547900229
H	5.492038524	2.442196234	-1.352224994
I	-3.086451245	-0.963532730	-3.462853472
I	-0.458105290	4.011419450	-1.158199951
C	-4.728540591	1.021401384	-0.250950200
C	-5.217002411	2.149398356	0.706693306
F	-5.800067954	0.274793103	-0.594431123
F	-4.233681938	1.629308667	-1.350332720
F	-4.171059389	2.915482319	1.067614452
F	-6.121003792	2.921816983	0.082329699
F	-5.776732670	1.635283979	1.809992622

TS0

E=-5287.878420754

C	-2.306280334	0.505000526	0.286587401

C	-1.997079442	-0.339431478	1.378346582
N	-3.143817090	-0.697031391	2.052237749
N	-4.172452962	-0.127237101	1.497111037
N	-3.680685260	0.591449294	0.431263159
C	-0.662451360	-0.796679014	1.779730428
C	-0.479032877	-2.032530266	2.438860024
C	0.474143934	-0.010005594	1.482284802
C	0.808939264	-2.477652022	2.762298501
H	-1.356673825	-2.641257854	2.677140673
C	1.759866922	-0.460866685	1.801389162
H	0.337836977	0.975095402	1.013797529
C	1.933459282	-1.700168506	2.436914076
H	0.937443183	-3.442320171	3.265055231
H	2.626749030	0.161070306	1.557083386
H	2.939369248	-2.055597168	2.683817297
C	1.113210706	-0.546945949	-1.626936841
C	0.225163967	-1.679111939	-1.439301874
H	0.051219413	-2.375966045	-2.266924397
H	-0.040178791	-1.981904918	-0.416301311
N	0.000397380	0.057057826	-1.883937311
Cu	-1.770221927	-0.672070548	-1.510484575
Cu	-1.056059633	1.570477260	-0.772737590
C	2.506947514	-0.193243993	-1.557232096
C	3.452068807	-1.186964806	-1.213546026
C	2.928876547	1.136961320	-1.790705713
C	4.805233229	-0.852934529	-1.107103990
H	3.111112515	-2.209733425	-1.023501418
C	4.282849493	1.461060347	-1.675425865
H	2.185295149	1.902306578	-2.036990427
C	5.221744290	0.470100041	-1.336333096
H	5.538594709	-1.620923774	-0.840873209
H	4.611130275	2.490963826	-1.848688250
H	6.281931050	0.730143168	-1.249954430
I	-3.686760988	-2.057564643	-2.359102456
I	0.073598284	3.786866492	-1.116943661
C	-4.573217237	1.415237362	-0.333077472
C	-4.811007760	2.805994707	0.324801472
F	-5.773256092	0.810362248	-0.460328327
F	-4.050772534	1.629837722	-1.563231972
F	-3.643526463	3.447214267	0.486692188
F	-5.611336536	3.555460630	-0.451358857
F	-5.392370056	2.655482499	1.525610467

I1 (C-C)

E=-5287.888890803

C	0.886682144	2.637594482	1.110688800
N	1.370402562	3.857999128	0.678949832
N	2.077880452	3.708802243	-0.394708845
N	2.066168855	2.346563309	-0.664294447
C	1.330418562	1.626505197	0.246214111
C	0.047017063	2.511694500	2.306859344
C	-0.283267930	3.649622886	3.076212177
C	-0.452350440	1.254668335	2.713950107
C	-1.087780953	3.530064586	4.215698489
H	0.102254969	4.625352428	2.765500256
C	-1.256235579	1.137116052	3.852994020
H	-0.197744762	0.357867047	2.135450968
C	-1.578695532	2.274667627	4.610683808
H	-1.332636206	4.423071658	4.801192072
H	-1.629410174	0.151456445	4.150847749
H	-2.207123126	2.182905914	5.503003489
Cu	1.067993978	-0.276739644	0.207552275
N	0.429140136	-2.069535454	-0.061844843
C	-0.510545823	-1.792232882	-0.916237944
Cu	1.207080539	-3.423110041	0.928928044
C	-0.441435528	-0.310113887	-1.106182814
H	-1.267713026	0.291091435	-0.699177750
H	-0.049313074	0.060507438	-2.063829838
C	-1.431582272	-2.747230046	-1.546794328
C	-2.377496542	-2.290277595	-2.488724295

C	-1.378635825	-4.122329771	-1.224372523
C	-3.256077585	-3.193665019	-3.100061843
H	-2.417361213	-1.224490582	-2.739592774
C	-2.256916105	-5.020289476	-1.835564680
H	-0.639326921	-4.467635805	-0.489590116
C	-3.197303663	-4.558252991	-2.774705492
H	-3.987675651	-2.834881839	-3.831604823
H	-2.212566354	-6.084681354	-1.582343776
H	-3.884818796	-5.264378262	-3.252626735
I	1.794374499	-5.585948758	1.932942154
I	2.915042371	-0.936477710	1.930576811
C	2.656697554	1.840259906	-1.858057932
C	3.864305216	2.675025467	-2.379985628
F	1.739955145	1.775814040	-2.873032299
F	3.091967468	0.572816252	-1.641278160
F	4.760000779	2.870445363	-1.403068321
F	3.468753693	3.855559425	-2.869342589
F	4.457877098	1.984799957	-3.371221710

I1 (C-N)

E=-5287.883833

C	2.282228014	-0.332691898	0.192619326
N	3.604980766	-0.163114707	-0.164521715
N	3.956324623	-1.088227097	-1.003013499
N	2.826722233	-1.875643578	-1.192486924
C	1.748001048	-1.459925632	-0.459725694
C	1.554610382	0.590114714	1.077542956
C	1.931117048	1.948131173	1.174013309
C	0.427536211	0.141751254	1.803290126
C	1.184798899	2.834000595	1.961357991
H	2.796680150	2.298948773	0.605328852
C	-0.319349444	1.032072588	2.581397065
H	0.148845961	-0.919270834	1.759827980
C	0.055537754	2.382971755	2.662440472
H	1.479115792	3.887618609	2.014824767
H	-1.197751709	0.670518059	3.125906783
H	-0.534773934	3.081829617	3.264409793
Cu	-0.144353457	-1.971469743	-0.475180523
N	-0.657864112	-0.307375792	-1.321998541
C	-1.846517936	-0.367783363	-0.761689087
Cu	0.674987490	0.954665508	-1.612875384
C	-2.165080736	-1.786232074	-0.538849017
H	-2.344819596	-2.382934976	-1.442689729
H	-2.718242201	-2.102775047	0.354861765
C	-2.628137120	0.799039797	-0.311127267
C	-2.015469945	2.062748787	-0.175891867
C	-3.996584108	0.656318486	0.000882567
C	-2.761295917	3.167579198	0.242078924
H	-0.940329656	2.159436373	-0.379958372
C	-4.740447244	1.762100506	0.430982832
H	-4.475119165	-0.323160652	-0.108866425
C	-4.124748423	3.019151489	0.548725266
H	-2.275958052	4.143157808	0.349164791
H	-5.802910399	1.646787997	0.670181143
H	-4.706560938	3.883246123	0.886957856
I	2.057526193	2.699993591	-2.655535517
I	-0.125010964	-4.150688450	0.855087223
C	2.890764416	-2.960661512	-2.129091203
C	3.770713467	-4.149797935	-1.646407030
F	3.400256869	-2.525232173	-3.307546542
F	1.642283295	-3.433285718	-2.347256304
F	5.061207412	-3.789412391	-1.587767996
F	3.651381649	-5.176960994	-2.507182829
F	3.375705381	-4.555538232	-0.432220324

TS1 (C-N)

E=-5287.859043

C	-0.264875997	-1.378904689	0.509887738
N	-0.716766582	-1.699484862	1.773334633
N	0.220124755	-1.509520424	2.651302184

N	1.322981212	-1.065209706	1.947141933
C	1.074249594	-0.965568153	0.596864185
C	-1.128178448	-1.433242846	-0.669794393
C	-2.529913809	-1.508128799	-0.524804378
C	-0.579953340	-1.362562229	-1.968772103
C	-3.357945345	-1.496523311	-1.652248661
H	-2.955621914	-1.553144022	0.481417434
C	-1.410186857	-1.346276544	-3.093700122
H	0.509692895	-1.325866266	-2.094160635
C	-2.804157732	-1.409940753	-2.940041072
H	-4.444944778	-1.543903644	-1.525962925
H	-0.965590426	-1.286753810	-4.092786830
H	-3.455904957	-1.394602860	-3.820145690
Cu	2.441181971	-0.779736097	-0.778989814
N	1.895567023	0.894885931	-0.082699350
C	0.960622821	1.505607787	-0.919205872
Cu	3.724099525	1.278611274	-0.293855518
C	1.383358299	2.074675400	-2.096320493
H	2.385459973	1.845129777	-2.478762893
H	0.787160174	2.828326957	-2.622253494
C	-0.424769482	1.690111286	-0.407459758
C	-0.672408152	1.670762854	0.980256076
C	-1.509758914	1.856785081	-1.292728147
C	-1.976160899	1.806402810	1.470916158
H	0.172049040	1.535781223	1.662065144
C	-2.811928472	2.000640265	-0.801701611
H	-1.333544819	1.826696728	-2.372315505
C	-3.050037588	1.970605554	0.581726248
H	-2.155591053	1.776575177	2.550911686
H	-3.647640578	2.106742544	-1.501189623
H	-4.071878823	2.063978288	0.964946321
I	6.047607754	2.024086158	-0.480679306
I	3.446333404	-2.625188386	-2.096272983
C	2.495351182	-0.634932331	2.638039445
C	2.772916730	-1.413106317	3.961685363
F	2.428579349	0.687350754	2.957087031
F	3.571039392	-0.805075460	1.825108480
F	1.927056068	-1.043470526	4.930288379
F	2.677247001	-2.734900379	3.763514428
F	4.025080802	-1.127713434	4.360358313

TS1(C-C)

E=-5287.867396

C	-1.655241619	-0.040206806	-1.030750057
N	-2.935087120	0.367114594	-1.332936202
N	-3.796221383	-0.224554519	-0.563028875
N	-3.065019232	-1.057051412	0.265734095
C	-1.709720283	-0.981483350	0.018834954
C	-0.470073667	0.502478705	-1.699095293
C	-0.515633830	1.780567755	-2.300546529
C	0.751601132	-0.204783853	-1.699591884
C	0.641512264	2.344451426	-2.847171500
H	-1.461953386	2.329965547	-2.307327242
C	1.910653063	0.366931673	-2.234320409
H	0.801695499	-1.214852819	-1.273593044
C	1.861281638	1.647381093	-2.803723579
H	0.597012344	3.342082246	-3.296965993
H	2.851349831	-0.192467547	-2.197646502
H	2.768792030	2.101582742	-3.215213976
Cu	-0.350299732	-2.307839301	0.402217369
N	1.372842382	-1.947576589	1.376804401
C	0.910078600	-0.723000417	1.505337669
Cu	2.991883326	-2.444570171	0.644724650
C	-0.579424893	-0.652092000	1.679107356
I	5.081095614	-3.097382296	-0.456615894
H	-1.062273937	0.326864582	1.741737112
H	-0.953841621	-1.340133848	2.449988946
I	-0.402296073	-4.229964573	-1.218976989
C	1.703419146	0.527502012	1.331436042
C	1.093615303	1.755405957	0.995689616

C	3.111340135	0.478013908	1.420776202
C	1.870828452	2.883714780	0.705595279
H	0.006087491	1.829639122	0.912989590
C	3.887345384	1.608274558	1.150944401
H	3.593253927	-0.471091925	1.691856634
C	3.269575431	2.814665166	0.779057358
H	1.379450758	3.816198070	0.410018886
H	4.978441480	1.547637349	1.223209529
H	3.877147952	3.696380182	0.548906503
C	-3.699649661	-1.774707588	1.322702611
C	-5.165729557	-2.205110241	1.015155075
F	-3.720227175	-1.028125338	2.466543114
F	-2.979569176	-2.892507123	1.592433281
F	-5.990028503	-1.151531947	1.014060186
F	-5.233961247	-2.830817414	-0.166432300
F	-5.564051085	-3.055000392	1.978075409

I2 (C-C)

E=-5287.956063

C	-2.235528902	-0.216186638	0.218869446
N	-3.459939425	-0.736820559	0.564543952
N	-3.465009918	-1.088208894	1.813527346
N	-2.217447158	-0.787828038	2.312785781
C	-1.392274089	-0.262414540	1.337757902
C	-1.876011489	0.170132059	-1.155975459
C	-1.148708395	1.346234400	-1.413751136
C	-2.104863748	-0.749367581	-2.202522563
C	-0.613867497	1.580310983	-2.687536074
H	-0.976584703	2.066685750	-0.607683319
C	-1.553006044	-0.520240006	-3.469596292
H	-2.673007312	-1.662218049	-1.997758011
C	-0.792400637	0.636370391	-3.709056073
H	-0.030149982	2.487524878	-2.872095443
H	-1.691014205	-1.260718864	-4.263115684
H	-0.336993581	0.795884349	-4.691346037
Cu	-0.531662193	-2.436501884	-0.347124145
N	0.751890460	-1.075693591	-0.409959639
C	0.915523248	-0.030747163	0.318986603
Cu	1.178926656	-1.632739900	-2.127947747
C	0.018783317	0.209685061	1.548476001
I	1.780720947	-2.515865068	-4.339538007
H	-0.011091022	1.282617410	1.806970977
H	0.465430495	-0.301112005	2.416116366
I	-2.056340905	-4.363164942	-0.342336021
C	1.964279980	0.996146197	0.041307717
C	2.523563956	1.106738603	-1.250749333
C	2.433717961	1.873354548	1.045379957
C	3.512769342	2.052474334	-1.532386000
H	2.152526809	0.439558870	-2.041670116
C	3.435450164	2.815329744	0.768641589
H	2.035553868	1.811354205	2.062957082
C	3.976659863	2.912668689	-0.521568305
H	3.919102996	2.126721034	-2.546936463
H	3.793059433	3.475018321	1.566639191
H	4.751611160	3.655168590	-0.740351119
C	-1.909604039	-1.173001584	3.668257981
C	-1.258427664	-2.584782175	3.769764848
F	-3.039730764	-1.184002508	4.395408429
F	-1.049943667	-0.272734556	4.208090300
F	-0.943305839	-2.848614746	5.047541615
F	-2.109218856	-3.512793960	3.326907738
F	-0.133438620	-2.624221166	3.032072167

I2 (C-N)

E=-5287.925091698

C	0.108543311	0.825632420	2.246462017
N	0.428407258	1.752323854	3.206170224
N	0.569747786	2.936151644	2.708279036
N	0.329887031	2.808244435	1.332311836
C	0.070039741	1.478769201	0.983167383

C	-0.173104331	-0.566329914	2.605202063
C	0.390486641	-1.113299687	3.781698060
C	-1.021135619	-1.382816628	1.822765358
C	0.128802804	-2.437539114	4.150540027
H	1.039827141	-0.482747643	4.396784395
C	-1.279971879	-2.707766999	2.195058439
H	-1.502978332	-0.970156940	0.932404776
C	-0.702590293	-3.245095220	3.355920141
H	0.582275730	-2.845334884	5.060685076
H	-1.940684706	-3.322282140	1.573729536
H	-0.900198454	-4.283854772	3.641290069
Cu	1.858172097	0.034402032	-2.159615743
N	-0.203125819	1.085512662	-0.277756839
C	0.196472509	-0.192960212	-0.745373404
Cu	0.116012002	1.846417715	-2.029271926
C	1.355138206	-0.852547774	-0.319113378
H	1.962056758	-0.408926956	0.476686075
H	1.480411805	-1.924482499	-0.493443200
C	-0.733011446	-0.800355654	-1.748133702
C	-2.017179468	-0.245612881	-1.946441784
C	-0.334242922	-1.891939169	-2.555416932
C	-2.869255856	-0.755368017	-2.936171982
H	-2.334186974	0.588359728	-1.313047291
C	-1.188542373	-2.396657523	-3.540056755
H	0.664416100	-2.326318056	-2.436184421
C	-2.458572985	-1.828075970	-3.739636112
H	-3.859273143	-0.308991020	-3.078799236
H	-0.856980407	-3.233496103	-4.163924534
H	-3.120611703	-2.220171585	-4.518950057
I	1.122255351	1.810344392	-4.243233335
I	4.160515125	-0.830949742	-2.818217274
C	0.671283905	3.912543680	0.498048963
C	2.146229013	3.880549467	-0.010415887
F	0.507673819	5.065238398	1.178402316
F	-0.148323160	3.953506256	-0.597502002
F	2.343768534	4.829859785	-0.935016816
F	2.980960690	4.085697113	1.017054758
F	2.430586513	2.680524317	-0.556937938

2CuI, R= tosyl (Figure 4)

RC'

E=-5531.94228

C	0.904130405	1.124165602	-0.360703548
C	0.527662544	1.503876257	0.953296230
N	1.561013126	2.184127209	1.564071236
N	2.588292717	2.240309220	0.759487938
N	2.199525871	1.623438641	-0.391024496
C	-0.733673183	1.240476796	1.657495247
C	-1.122323747	2.033663823	2.759570164
C	-1.571278509	0.175933774	1.258289422
C	-2.323383931	1.775217080	3.431666959
H	-0.473357973	2.857057077	3.072970713
C	-2.769430874	-0.080776113	1.932622362
H	-1.273576009	-0.439700144	0.400828781
C	-3.153746064	0.719772854	3.020595577
H	-2.615598603	2.404806187	4.279339302
H	-3.407277680	-0.908702385	1.605871532
H	-4.093786280	0.520628667	3.546328331
S	3.393067906	1.247683259	-1.628018908
O	2.600524824	0.700513320	-2.746192192
O	4.257531118	2.428305632	-1.753149784
C	4.275274818	-0.082508264	-0.844171168
C	3.745909165	-1.378710534	-0.937858529
C	5.437659498	0.197564494	-0.114456935
C	4.406814347	-2.416814203	-0.276395383
H	2.831002546	-1.565077651	-1.512744495
C	6.084920971	-0.860153592	0.534208483
H	5.819354652	1.220333726	-0.057516459
C	5.583625930	-2.176864307	0.466106001

H	4.000815790	-3.432130995	-0.337763945
H	6.995116113	-0.658042889	1.108709397
C	6.300243686	-3.314425032	1.152731015
H	6.975390124	-2.946769079	1.942255230
H	5.583514189	-4.024695121	1.598533669
H	6.910686814	-3.879581808	0.423226851
C	-3.502138689	1.449063798	-0.979883927
C	-3.425081099	2.802826354	-0.440136340
H	-3.874647341	3.647184099	-0.980898604
H	-3.232133947	2.951689830	0.631692660
N	-2.369879691	2.000801034	-1.199493644
Cu	-0.488877717	1.987228298	-1.607691564
Cu	0.435227004	-0.279998138	-1.606199728
C	-4.165492899	0.186398662	-1.159051572
C	-5.499991497	0.002366185	-0.734857222
C	-3.440916295	-0.892450573	-1.716440903
C	-6.100868125	-1.253468743	-0.872072760
H	-6.048035546	0.841932135	-0.295825492
C	-4.046527172	-2.143044582	-1.843623500
H	-2.397069942	-0.745111222	-2.015479184
C	-5.377040705	-2.324481905	-1.424142431
H	-7.134890694	-1.402952702	-0.544664729
H	-3.476894964	-2.978090712	-2.263945971
H	-5.850839657	-3.306862009	-1.523925117
I	0.492037916	3.820011792	-3.101022147
I	0.059416762	-2.395963104	-2.830546422

TS0

E=-5531.921037184

C	0.917439108	1.097913984	0.002801380
C	0.525659195	1.496817682	1.301962894
N	1.569103903	2.128615187	1.944893893
N	2.615882845	2.150177125	1.163506758
N	2.225791965	1.538660820	0.005276839
C	-0.792475648	1.309751664	1.917258948
C	-1.269681841	2.198238006	2.906650238
C	-1.625276707	0.243975950	1.503846691
C	-2.551007970	2.034848253	3.447952342
H	-0.627561484	3.022914008	3.231204099
C	-2.907416503	0.087991170	2.042500670
H	-1.253400977	-0.468505381	0.754279631
C	-3.378143354	0.985235841	3.013969461
H	-2.911025366	2.737083113	4.207890181
H	-3.539435808	-0.739237868	1.703714301
H	-4.382494397	0.865097775	3.433885111
S	3.345937249	1.311874125	-1.326512492
O	2.476345023	0.948809578	-2.463367532
O	4.233113316	2.482456614	-1.343489749
C	4.246143122	-0.126141249	-0.794078568
C	3.614304906	-1.375169656	-0.870836137
C	5.546693450	0.023476882	-0.293505949
C	4.313449297	-2.502266667	-0.430361546
H	2.589144025	-1.460195989	-1.249213481
C	6.228652888	-1.120682382	0.133290535
H	6.005376830	1.014592655	-0.242235343
C	5.627877142	-2.396556905	0.071370479
H	3.826274081	-3.482054639	-0.478421946
H	7.246587616	-1.021010687	0.525175680
C	6.387659577	-3.623691321	0.513769079
H	5.707673590	-4.469649139	0.703942436
H	7.103155858	-3.935123753	-0.270464363
H	6.972452640	-3.423157793	1.427580544
C	-2.803031023	0.924385470	-1.458772994
C	-2.410874088	2.268746529	-1.080037376
H	-2.646425726	3.110397967	-1.741093627
H	-2.138738280	2.468494980	-0.033652272
N	-1.594502836	0.882360285	-1.915840282
Cu	-0.221439999	2.210036918	-1.475192921
Cu	0.118457585	-0.214313160	-1.216969767
C	-3.921306823	0.020708517	-1.372891553

C	-5.101374878	0.445266047	-0.719601438
C	-3.836938929	-1.289056107	-1.901650901
C	-6.183726541	-0.431017165	-0.597024414
H	-5.150399195	1.456763020	-0.303802326
C	-4.923055456	-2.158227308	-1.770141280
H	-2.912629027	-1.613369180	-2.390242777
C	-6.095980528	-1.732748740	-1.120584146
H	-7.096633542	-0.104002144	-0.088938498
H	-4.857703676	-3.174038066	-2.173268759
H	-6.944111529	-2.418448360	-1.021714650
I	0.757978126	4.457152999	-2.030410964
I	-0.124361208	-2.644179505	-1.782404138

I1 (C-C)

E=-5531.93941

C	3.520758130	1.053763273	-0.675610066
N	4.270196277	2.083563055	-0.140785453
N	3.503525536	3.093754864	0.147691227
N	2.225885244	2.695747573	-0.197314049
C	2.170289195	1.438575577	-0.739848009
S	0.943881430	3.905575737	-0.141934709
O	1.196738663	4.660088376	1.097564627
O	0.918329163	4.581670097	-1.449850319
C	-0.523037101	2.907985908	0.035985077
C	-0.800290602	2.300200531	1.272524561
C	-2.030175782	1.656852011	1.441234673
C	-2.998373295	1.647597674	0.415024036
C	-2.689351243	2.273101521	-0.814340867
C	-1.456178946	2.907968043	-1.012718098
C	-4.347383680	1.009891099	0.618169016
H	-0.069483206	2.339560605	2.085643028
H	-2.251198699	1.165912927	2.394232806
H	-3.439814951	2.306576297	-1.611008880
H	-1.221647064	3.410212757	-1.953501183
H	-4.612066511	0.395414736	-0.260259068
H	-4.365713608	0.379466756	1.520381693
H	-5.126815261	1.787959272	0.719466244
C	4.118690919	-0.228708905	-1.062508573
C	5.451788864	-0.540423067	-0.711145581
C	3.372319621	-1.183837236	-1.789473926
C	6.012288976	-1.773426207	-1.066227483
H	6.036146156	0.197290194	-0.152603093
C	3.932823531	-2.418757331	-2.136689602
H	2.346883726	-0.942518322	-2.098452098
C	5.255875076	-2.721182955	-1.775675200
H	7.046557426	-1.999223604	-0.783647209
H	3.335249566	-3.144073921	-2.699553606
H	5.695210517	-3.686491303	-2.049126901
Cu	0.568230021	0.443958185	-1.127430747
N	-0.989492882	-0.660952423	-0.961547760
C	-0.765783268	-1.109699842	0.237132211
Cu	-2.240345807	-0.059884766	-2.202833138
C	0.618985362	-0.660622433	0.555284481
H	1.423217477	-1.385397843	0.362066471
H	0.782911081	-0.024073344	1.434173337
C	-1.716174701	-1.842084716	1.082119513
C	-3.000160167	-2.170009461	0.591655662
C	-1.356572287	-2.209114938	2.395891534
C	-3.906850642	-2.852850938	1.405660502
H	-3.268137593	-1.871632843	-0.428775666
C	-2.271690597	-2.886395057	3.212045813
H	-0.358882030	-1.958209793	2.771853385
C	-3.546315441	-3.208847708	2.718521125
H	-4.900886045	-3.106639248	1.023022629
H	-1.991576892	-3.165351605	4.233151459
H	-4.261113351	-3.739441486	3.356613353
I	-4.415051786	-0.128500792	-3.406675117
I	0.173781477	1.035665018	-3.677572061

I1 (C-N)

E= -5531.934235

C	2.046785413	2.421384188	-0.964758771
N	1.667164243	3.112683224	-2.101071219
N	1.421340421	2.281952556	-3.070857898
N	1.687050090	1.027224530	-2.550486407
C	2.058986945	1.041230889	-1.228361536
S	1.099376953	-0.407135569	-3.355531186
O	1.267359213	-0.177394155	-4.797389060
O	1.759627846	-1.526538342	-2.650202972
C	-0.622386135	-0.376137459	-2.912774439
C	-1.475989565	0.546608058	-3.535858766
C	-2.809204252	0.598675913	-3.125001868
C	-3.299684524	-0.255224249	-2.112896807
C	-2.419895062	-1.183091307	-1.526689655
C	-1.072439559	-1.247344676	-1.909341536
C	-4.717502922	-0.119119660	-1.623196887
H	-1.094378588	1.222023087	-4.306117958
H	-3.481641341	1.327644365	-3.588370817
H	-2.785692943	-1.865133419	-0.752728402
H	-0.389961437	-1.978589855	-1.472449989
H	-5.425373541	0.007614421	-2.459915481
H	-5.024685929	-0.989937825	-1.021925507
H	-4.792724070	0.781795003	-0.984475975
C	2.307326411	3.094095349	0.310634227
C	2.586896688	2.336901368	1.469744204
C	2.243759751	4.499703271	0.420120357
C	2.796871526	2.964742855	2.702035722
H	2.630449455	1.243909753	1.393495684
C	2.455147234	5.126557414	1.654171750
H	2.017598391	5.087859356	-0.474088889
C	2.732890865	4.364529346	2.801039792
H	3.010056778	2.358612099	3.589290822
H	2.400489417	6.218765497	1.723389023
H	2.896398550	4.857437791	3.765618886
Cu	2.403652537	-0.610138189	-0.198410214
N	0.665702762	-0.711201044	0.627662562
C	0.812563408	-1.874174079	1.185256034
Cu	-0.411993676	0.689926149	0.084291692
C	2.243107106	-2.239317864	0.956320467
H	2.450279189	-3.150839796	0.376007789
H	2.911506330	-2.096385042	1.818217540
C	-0.252712905	-2.629655351	1.860925517
C	0.021241112	-3.899548027	2.409823421
C	-1.556241391	-2.092755664	1.964781010
C	-0.994301368	-4.624412397	3.047114507
H	1.033344496	-4.311628684	2.333501886
C	-2.565394226	-2.816180042	2.605187299
H	-1.756223675	-1.102669209	1.536576686
C	-2.286918409	-4.085210010	3.145552161
H	-0.777827460	-5.610836845	3.470411817
H	-3.572807569	-2.394646464	2.686833803
H	-3.079451013	-4.652268195	3.645297592
I	-1.856720702	2.653198803	-0.138633151
I	4.895179127	-0.837561865	-0.721766857

TS1 (C-N)

E= -5531.907143806

C	-1.701953580	-1.021456348	0.232611900
N	-2.381724419	-1.229644070	1.413055432
N	-1.610597450	-0.974327582	2.431372828
N	-0.401275444	-0.596642385	1.912380272
C	-0.386895644	-0.608848383	0.537046048
S	0.847497550	-0.044539302	3.061566250
O	0.845963671	1.428522633	3.043891950
O	0.536022116	-0.783529774	4.295121284
C	2.351522437	-0.654380850	2.337950055
C	3.215738660	0.249923993	1.696695690
C	4.452050952	-0.220553219	1.236671884
C	4.828663411	-1.568254129	1.403767216
C	3.937095627	-2.444794563	2.061591667

C	2.701636935	-2.000312487	2.538812296
C	6.140885581	-2.071923758	0.854808320
H	2.937537217	1.304734439	1.600859630
H	5.127169249	0.478554246	0.732005914
H	4.217098454	-3.493681795	2.204914245
H	2.022927964	-2.679194817	3.062822458
H	6.001271213	-2.398792128	-0.193521986
H	6.909314657	-1.280900807	0.855957598
H	6.514571578	-2.937362915	1.426860572
C	-2.349935110	-1.251007545	-1.064780945
C	-3.741792366	-1.499588983	-1.124867603
C	-1.617401569	-1.234095272	-2.272456385
C	-4.374542741	-1.717094051	-2.353773402
H	-4.315236968	-1.513629925	-0.193435344
C	-2.253323763	-1.451155333	-3.501707113
H	-0.533188583	-1.069732854	-2.244598132
C	-3.635250314	-1.692135770	-3.549290724
H	-5.453708333	-1.905401302	-2.381075643
H	-1.663315657	-1.437229710	-4.424691247
H	-4.132850031	-1.862268580	-4.510508336
Cu	1.203254571	-0.647843912	-0.616039384
N	0.570886686	1.101732338	-0.272266418
C	-0.391079028	1.732008898	-1.062693420
Cu	2.335246525	1.540354346	-0.883432047
C	-0.053519472	2.122806012	-2.338567154
H	0.835327874	1.697633662	-2.822495577
H	-0.602089635	2.907918291	-2.871226185
C	-1.669483865	2.175071191	-0.430297410
C	-1.736736842	2.403038803	0.961508198
C	-2.823215406	2.401445080	-1.212488757
C	-2.924890968	2.853672676	1.551255049
H	-0.845280858	2.223812552	1.570165448
C	-4.009413312	2.855953536	-0.621295395
H	-2.795148620	2.194228488	-2.287517273
C	-4.064192210	3.085143261	0.763133106
H	-2.961643728	3.026599463	2.632670657
H	-4.897961091	3.019263225	-1.241357066
H	-4.993399146	3.435552074	1.226526863
I	4.406250043	2.680579754	-1.602720498
I	2.383113183	-2.628226413	-1.568919384

TS1(C-C)

E= -5531.919455874

C	-3.116569671	-0.627486102	0.025338199
N	-3.993250883	-0.128843223	0.961440501
N	-3.358723695	0.587969710	1.845606147
N	-2.035145650	0.557698233	1.485121398
C	-1.810706439	-0.197261756	0.350425549
S	-0.864737643	1.560457834	2.361507355
O	-0.463241130	2.624184171	1.422526175
O	-1.529238199	1.865918835	3.635831531
C	0.477026157	0.427632966	2.583780998
C	1.632204146	0.601202783	1.807355721
C	2.686165448	-0.303154910	1.967886937
C	2.597869857	-1.363374590	2.891180375
C	1.433915638	-1.479931915	3.682601172
C	0.365333141	-0.593777922	3.540484474
C	3.695616002	-2.388837896	2.983476023
H	1.701987273	1.437733345	1.108080740
H	3.590042260	-0.187983791	1.359047000
H	1.357504152	-2.294560337	4.409720351
H	-0.541241111	-0.697815933	4.142464231
H	3.767463694	-2.816384807	3.997063638
H	3.470372918	-3.215099344	2.282333707
H	4.670300022	-1.967598265	2.690368057
C	-3.562765594	-1.444766709	-1.102932136
C	-4.935048619	-1.498980414	-1.442308707
C	-2.642927085	-2.185674956	-1.879350280
C	-5.366970757	-2.264570315	-2.530204454
H	-5.650318190	-0.929669670	-0.841139956

C	-3.078650187	-2.943828018	-2.972612029
H	-1.580498925	-2.194160954	-1.603747209
C	-4.441691099	-2.986884374	-3.303508269
H	-6.432851492	-2.296185991	-2.780693815
H	-2.349727514	-3.513508132	-3.558604932
H	-4.783014963	-3.584045046	-4.155798831
Cu	-0.141427763	-1.042769319	-0.097804996
N	1.314583535	-0.415439001	-1.298306618
C	0.724328775	0.749370285	-1.306700041
Cu	3.058202762	-0.985263607	-1.137317811
C	-0.783640223	0.706299349	-1.107581143
I	5.368556395	-1.775789915	-0.890415775
H	-1.315163475	0.251636749	-1.955273108
H	-1.238438558	1.651530398	-0.793426828
I	0.077883160	-3.376675533	0.864997067
C	1.407687119	2.071788257	-1.441705286
C	0.688580679	3.248095554	-1.746689683
C	2.806391415	2.164433500	-1.276424137
C	1.350023898	4.474529614	-1.888210004
H	-0.395378281	3.207482753	-1.888477350
C	3.468474637	3.387682383	-1.413215391
H	3.372418688	1.251333042	-1.036244791
C	2.741227271	4.550176863	-1.720116305
H	0.776327274	5.375278814	-2.130417614
H	4.553796641	3.437190592	-1.274530423
H	3.257084192	5.510696711	-1.824879422

I2 (C-C)

E= -5532.00791

C	-2.960566954	0.487101057	0.413319371
N	-3.619192309	0.287717384	-0.783923931
N	-2.889166750	0.713847915	-1.770891833
N	-1.736838894	1.210885355	-1.228461962
C	-1.723170182	1.079589072	0.146269852
S	-0.446507095	1.722232051	-2.350226433
O	-1.186872777	2.177718997	-3.533611683
O	0.415067835	2.624870366	-1.570234098
C	0.398856547	0.206402151	-2.701528749
C	1.772266768	0.118939225	-2.427229793
C	2.448781988	-1.062935271	-2.774616611
C	1.765882662	-2.139992568	-3.388355672
C	0.378603287	-2.018019940	-3.617238797
C	-0.314020878	-0.849153610	-3.295309942
C	2.495049630	-3.401476146	-3.759161511
H	2.296786390	0.962341314	-1.971304200
H	3.530378256	-1.125975436	-2.616467131
H	-0.169670781	-2.853887850	-4.061403287
H	-1.387985991	-0.763046321	-3.482843441
H	3.525648532	-3.186670333	-4.087236564
H	2.570730566	-4.057130191	-2.869522953
H	1.966319080	-3.950358594	-4.554761358
C	-3.545035007	0.042346227	1.688913917
C	-4.265978560	-1.171455160	1.720747761
C	-3.406851958	0.787685916	2.879458786
C	-4.817164319	-1.635854125	2.920890177
H	-4.361080375	-1.752674843	0.799038923
C	-3.954718353	0.315235767	4.079960657
H	-2.891336515	1.752993888	2.863716508
C	-4.658686184	-0.898475691	4.105524481
H	-5.365165830	-2.584087477	2.933405225
H	-3.839356313	0.903709846	4.996351930
H	-5.085304040	-1.266774113	5.044707515
Cu	1.928203582	-1.699484852	-0.303430902
N	0.679224628	-0.412170236	0.267431151
C	0.702176190	0.758596977	0.794193739
Cu	-0.661656350	-1.573353689	-0.315407518
C	-0.607212950	1.533471088	1.026756441
I	-2.215113523	-3.359950588	-0.964749001
H	-0.443699000	2.612459502	0.873281533
H	-0.918080323	1.397127036	2.076044854

I	3.489787781	-3.610106879	-0.040638598
C	1.963742923	1.430780722	1.227829918
C	1.949006029	2.584132240	2.045389867
C	3.220096338	0.917114976	0.837198812
C	3.143032912	3.191233279	2.458887306
H	0.999578652	3.012515981	2.379233577
C	4.412334804	1.524818087	1.239924800
H	3.250079667	0.017009557	0.205688052
C	4.380557375	2.668103441	2.055998808
H	3.104230426	4.078877652	3.099601475
H	5.371063499	1.106383329	0.914780886
H	5.312945862	3.146793516	2.374009645

I2 (C-N)

E= -5531.980738

C	-1.042887267	0.578856737	2.386399939
N	-0.790261571	1.417366925	3.444995887
N	-0.524687144	2.626689471	3.069993445
N	-0.637321656	2.623995393	1.659238923
C	-0.916989667	1.329659536	1.185423773
S	0.338025836	3.757231443	0.780289118
O	-0.371559589	4.065855457	-0.496434580
O	0.649954335	4.842463254	1.715947599
C	1.744594221	2.735724789	0.395182686
C	2.161771368	2.618460987	-0.941147457
C	3.218066934	1.744831472	-1.238805464
C	3.865019585	1.011496079	-0.218902932
C	3.420283207	1.164926816	1.111238668
C	2.360432083	2.019929481	1.431641852
C	4.992282245	0.073830631	-0.557343696
H	1.700343294	3.227008602	-1.725076316
H	3.554557309	1.652037006	-2.275720070
H	3.907551099	0.597656236	1.910919827
H	2.008091515	2.114512942	2.463547944
H	5.781140574	0.595937410	-1.127175457
H	5.438903578	-0.365489332	0.348574868
H	4.612223471	-0.738385887	-1.206760783
C	-1.399490305	-0.825343643	2.601485815
C	-0.996059098	-1.478705250	3.789964560
C	-2.160473596	-1.555458585	1.660252324
C	-1.326377895	-2.819513282	4.016559037
H	-0.415285719	-0.917466683	4.528424056
C	-2.484956219	-2.898378922	1.888645491
H	-2.524545655	-1.064368165	0.754383144
C	-2.066390067	-3.540657594	3.063975497
H	-0.997450243	-3.308839270	4.940144358
H	-3.073214663	-3.443592049	1.142387239
H	-2.316669756	-4.592587976	3.239068954
Cu	0.949703683	0.108073994	-2.113913077
N	-1.033752788	1.084247901	-0.128001171
C	-0.675628783	-0.169124500	-0.681789138
Cu	-0.507811240	2.150120027	-1.678304663
C	0.473614497	-0.862583071	-0.288925809
H	1.082950047	-0.456361455	0.525972657
H	0.602652628	-1.921232429	-0.527356912
C	-1.626513336	-0.705448221	-1.702086938
C	-2.867109773	-0.064713889	-1.909859478
C	-1.302338764	-1.829170302	-2.496324186
C	-3.752554991	-0.524899884	-2.892393621
H	-3.119032965	0.803713749	-1.294264874
C	-2.190234231	-2.286148524	-3.475144452
H	-0.337015011	-2.329523773	-2.374181961
C	-3.417848934	-1.635007403	-3.681590989
H	-4.708140944	-0.010971946	-3.042872474
H	-1.917203760	-3.151596149	-4.088407560
H	-4.107161337	-1.991056493	-4.454747184
I	0.096431210	1.927400493	-4.067215602
I	2.648374246	-1.385402155	-3.369910817

X-ray crystallography

The single-crystal diffraction data of **5j** was collected using Bruker D8 VENTURE system equipped with a Photon 100 CMOS detector, a multilayer monochromator, and a MoKα Incoatec microfocus sealed tube (λ = 0.71073 Å) at 180 K. The frames were integrated with the with Bruker SAINT[26] software package. The structure was solved by direct methods with SIR92[27] (**5j**) and refined by full-matrix least-squares on F^2 with CRYSTALS.[28] The positional and anisotropic thermal parameters of all non-hydrogen atoms were refined. All hydrogen atoms were located in a difference Fourier map and then they were repositioned geometrically. They were initially refined with soft restraints on the bond lengths and angles to regularise their geometry, then their positions were refined with riding constraints.

Crystal data for (*E*)-1-phenyl-2-(4-(*p*-tolyl)-1-tosyl-1*H*-1,2,3-triazol-5-yl)ethen-1-amine **4s** (colorless, 0.38 x 0.40 x 0.48 mm): $C_{24}H_{22}N_4O_2S$, triclinic, space group *P*-1, a = 7.1962(5) Å, b = 9.4529(7) Å, c = 16.5643(12) Å, α = 99.095(6)°, β = 99.786(6)°, γ = 91.947(6)°, V = 1094.28(14) Å^3, Z = 2, M = 430.53, 8090 reflections measured, 5120 independent reflections. Final R = 0.0550, wR = 0.1294, GoF = 1.0064 for 3382 reflections with I > 2σ(I) and 281 parameters. CCDC 1469919.

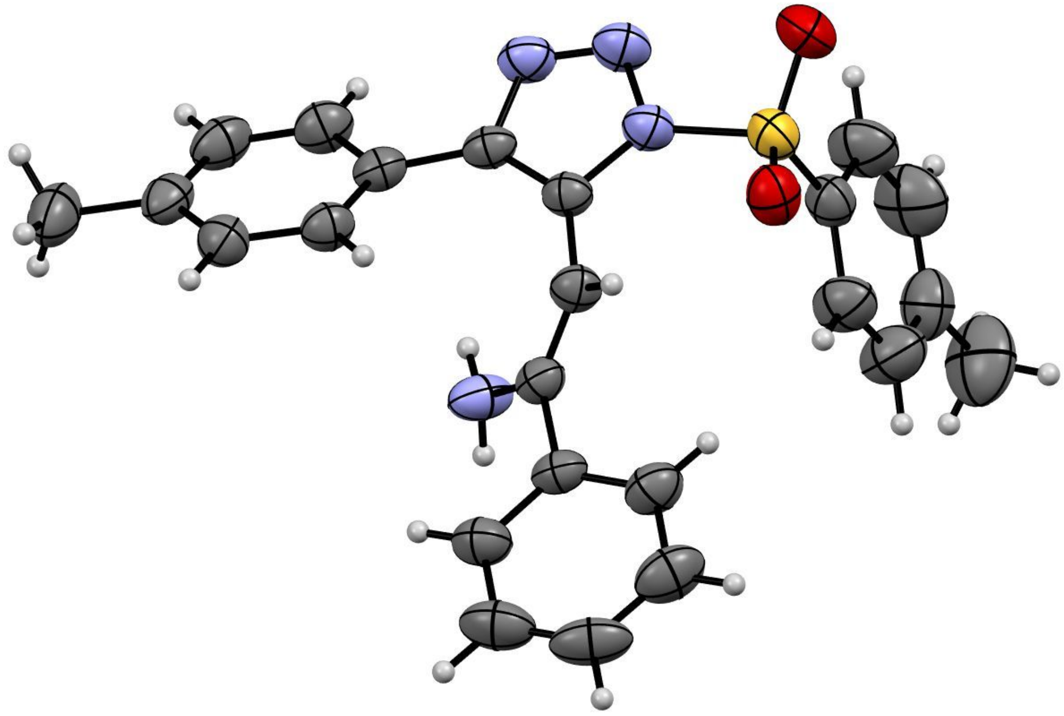

Figure 6. ORTEP[29] diagram of **4s**, displacement ellipsoids shown with 50 % probability.

Crystal data for 5-phenyl-3-(pyridin-3-yl)-7-(trifluoromethyl)-[1,2,3]triazolo[1,5-*c*]pyrimidine **5j** (colorless, 0.047 x 0.083 x 0.250 mm): Single crystals suitable for X-ray diffraction were grown by slow evaporation from the mixture Et_2O/pentane (1:1) over 14 days at 25 °C. $C_{17}H_{10}F_3N_5$, monoclinic, space group $P2_1/c$, a = 14.1044(4) Å, b = 5.13870(10) Å, c = 20.0650(5) Å, β = 90.0240(11)°, V = 1454.28(6) Å^3, Z = 4, M = 341.29, 22469 reflections measured, 2781 independent reflections. Final R = 0.0386, wR = 0.1003, GoF = 0.9852 for 2426 reflections with I > 2σ(I) and 226 parameters. CCDC 2428749.

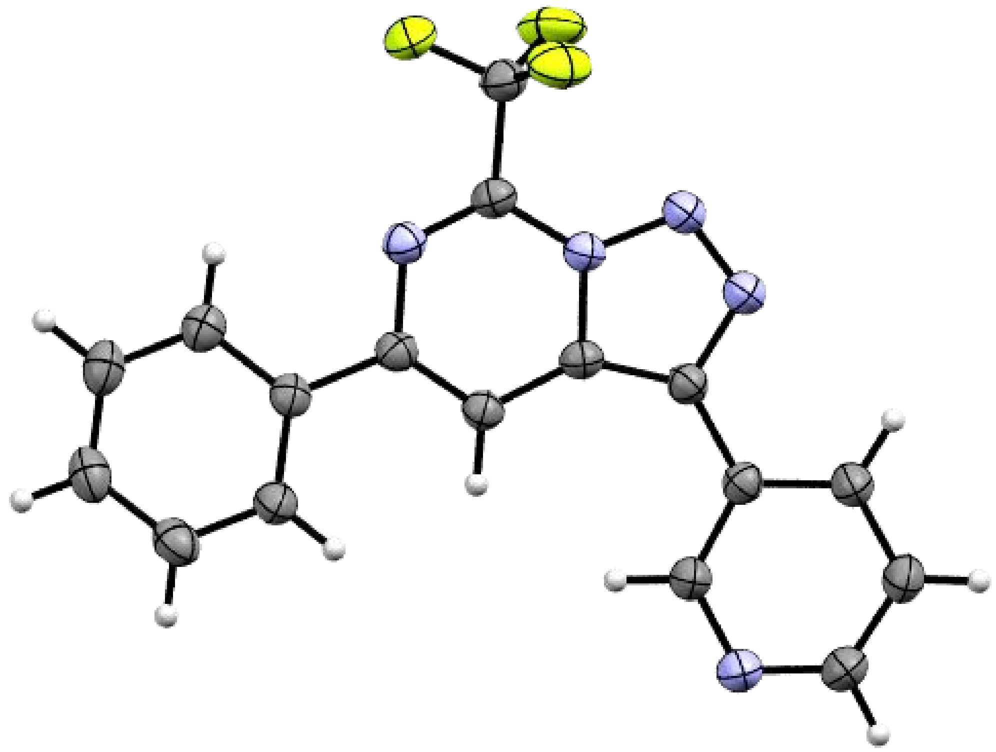

Figure 7. ORTEP[29] diagram of **5j**, displacement ellipsoids shown with 50 % probability.

Preparation and characterization of 2*H*-azirines **1**

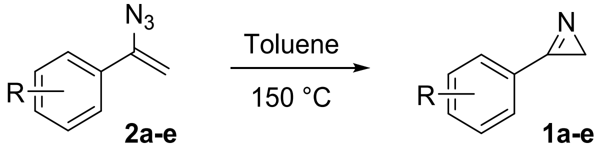

3-phenyl-2H-azirine (**1a**): Under air atmosphere, to the 250 mL round-bottom flask charged with vinyl azide

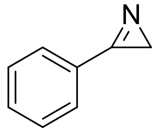

2a (2.78 g, 19.15 mmol) in toluene (95 mL). The reaction flask was equipped with reflux condenser, stirred and heated on a heating block at 150 °C for 3 h. Then the solvent was evaporated and azirine **1a** was purified by column chromatography (PET/EtOAc 9:1). Yield: 1.529 g pale yellow oil; 68%; ^{1}H NMR (400 MHz, $CDCl_3$) δ 7.93 – 7.90 (m, 2H), 7.60 – 7.54 (m, 3H), 1.79 (d, *J* = 0.5 Hz, 2H); ^{13}C NMR (101 MHz, $CDCl_3$) δ 165.9, 133.0, 129.7, 129.2, 125.6, 19.8; HRMS (EI) *m/z* calcd for C_8H_7N $[M]^+$: 117.0573, found 117.0571.

3-(p-tolyl)-2H-azirine (**1b**): Under air atmosphere, to the 50 mL round-bottom flask charged with vinyl azide

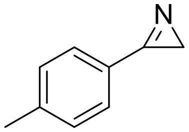

2b (220 mg, 1.38 mmol) in toluene (15 mL). The reaction flask was equipped with reflux condenser, stirred and heated on a heating block at 150 °C for 3 h. Then the solvent was evaporated and azirine **1b** was purified by column chromatography (PET/EtOAc 9:1). Yield: 100.0 mg pale pink oil; 55%; ^{1}H NMR (401 MHz, $CDCl_3$) δ 7.83 – 7.78 (m, 2H), 7.39 – 7.35 (m, 2H), 2.46 (s, 3H), 1.76 (s, 2H); ^{13}C NMR (101 MHz, $CDCl_3$) δ 165.3, 143.8, 129.9, 129.7, 122.9, 21.9, 19.5; HRMS (ESI$^+$) *m/z* calcd for $C_9H_{10}N$ $[M+H]^+$: 132.0808, found 132.0809.

3-(3,5-dimethoxyphenyl)-2H-azirine (**1c**): Under air atmosphere, to the 50 mL round-bottom flask charged

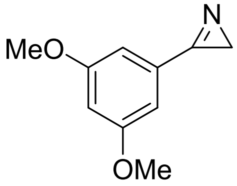

with vinyl azide **2c** (174 mg, 0.85 mmol) in toluene (10 mL). The reaction flask was equipped with reflux condenser, stirred and heated on a heating block at 150 °C for 3 h. Then the solvent was evaporated and azirine **1c** was purified by column chromatography (PET/EtOAc 9:1). Yield: 116.0 mg white solid; 77%; ^{1}H NMR (400 MHz, $CDCl_3$) δ 6.96 (d, *J* = 2.3 Hz, 2H), 6.59 (t, *J* = 2.3 Hz, 1H), 3.78 (s, 6H), 1.72 (s, 2H); ^{13}C NMR (101 MHz, $CDCl_3$) δ 166.0, 161.1, 127.1, 106.9, 105.5, 55.5, 20.1; HRMS (EI) *m/z* calcd for $C_{10}H_{11}NO_2$ $[M]^+$: 177.0784, found 177.0783.

3-(2-methoxyphenyl)-2H-azirine (**1d**): Under air atmosphere, to the 50 mL round-bottom flask charged with

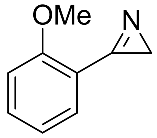

vinyl azide **2d** (149 mg, 0.85 mmol) in toluene (10 mL). The reaction flask was equipped with reflux condenser, stirred and heated on a heating block at 150 °C for 3 h. Then the solvent was evaporated and azirine **1d** was purified by column chromatography (PET/EtOAc 9:1). Yield: 76.0 mg pale yellow oil; 61%; ^{1}H NMR (400 MHz, $CDCl_3$) δ 7.71 – 7.67 (m, 1H), 7.55 – 7.50 (m, 1H), 7.10 (td, *J* = 7.5, 1.0 Hz, 1H), 7.02 – 6.99 (m, 1H), 3.96 (s, 3H), 1.61 (s,

2H); ^{13}C NMR (101 MHz, $CDCl_3$) δ 162.5, 159.5, 134.4, 133.0, 120.7, 114.4, 111.2, 55.9, 17.1; HRMS (ESI^+) *m/z* calcd for $C_9H_{10}NO$ $[M+H]^+$: 148.0757, found 148.0756.

3-(4-fluorophenyl)-2H-azirine (**1e**):

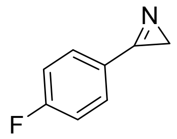

Under air atmosphere, to the 50 mL round-bottom flask charged with vinyl azide **2e** (247 mg, 1.51 mmol) in toluene (15 mL). The reaction flask was equipped with reflux condenser, stirred and heated on a heating block at 150 °C for 2 h. Then the solvent was evaporated and azirine **1e** was purified by column chromatography (PET/EtOAc 9:1). Yield: 136.0 mg pale yellow oil; 67%; ^1H NMR (400 MHz, $CDCl_3$) δ 7.90 – 7.85 (m, 2H), 7.24 – 7.18 (m, 2H), 1.75 (dd, *J* = 1.7, 0.8 Hz, 2H); ^{13}C NMR (101 MHz, $CDCl_3$) δ 165.5 (d, *J* = 254.9 Hz), 164.8, 131.9 (d, *J* = 9.3 Hz), 122.0 (d, *J* = 3.1 Hz), 116.6 (d, *J* = 22.4 Hz), 19.8; ^{19}F NMR (376 MHz, $CDCl_3$) δ -105.17 – (-105.27) (m, 1F); HRMS (EI) *m/z* calcd for C_8H_6NF $[M]^+$: 135.0479, found 135.0476.

Preparation and characterization of vinyl azides 2

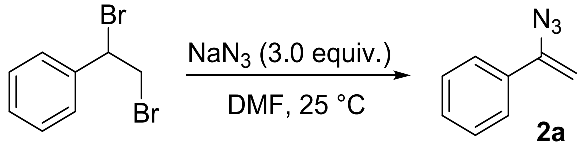

2a

(1-azidovinyl)benzene (**2a**): Under air atmosphere, to the 250 mL round-bottom flask charged with (1,2-

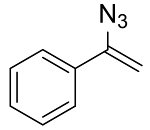

dibromoethyl)benzene (8.00 g, 30.01 mmol, 1.0 equiv.) and NaN_3 (5.91 g, 90.92 mmol, 3.0 equiv.) was added 50 mL DMF and the reaction mixture was stirred at room temperature for 16 h. Then it was filtered via paper, extracted by H_2O / EtOAc and organic phase was dried over anhydrous $MgSO_4$, evaporated with silica gel and purified by Combiflash chromatography (cyclohexane/EtOAc) to obtain **2a**. Yield: 3.736 g pale yellow oil; 85%; ^1H NMR (400 MHz, $CDCl_3$) δ 7.60 – 7.56 (m, 2H), 7.39 – 7.35 (m, 3H), 5.44 (d, *J* = 2.4 Hz, 1H), 4.97 (d, *J* = 2.4 Hz, 1H); ^{13}C NMR (101 MHz, $CDCl_3$) δ 145.2, 134.4, 129.2, 128.6, 125.7, 98.1; HRMS (ESI^+) *m/z* calcd for $C_8H_8N_3$ $[M+H]^+$: 146.0713, found 146.0712.

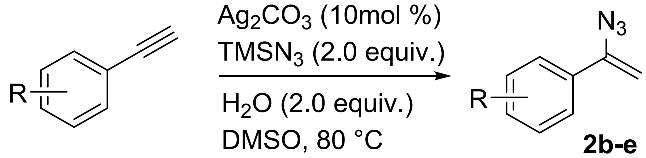

2b-e

General procedure: In inert atmosphere, to the 50 mL round-bottom flask charged with alkyne (2.0 mmol, 1.0 equiv.) in DMSO (10 mL, dried on sieves and degassed!) and H_2O (4.0 mmol, 2.0 equiv.) was added powdered Ag_2CO_3 (0.2 mmol, 0.1 equiv.) and $TMSN_3$ (4 mmol, 2 equiv.). The reaction mixture was stirred

at 80 °C for 20 h. Then it was filtered via paper, washed by Et_2O and extracted by H_2O / EtOAc and the organic phase was dried over anhydrous $MgSO_4$, evaporated and purified by column chromatography (PET/EtOAc, 9:1) to obtain vinyl azide **2b-e**.

1-(1-azidovinyl)-4-methylbenzene (**2b**): Prepared according to the general procedure. Yield: 146 mg

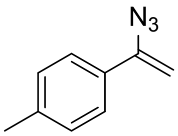

colorless oil; 46%; ^{1}H NMR (400 MHz, $CDCl_3$) δ 7.47 – 7.44 (m, 2H), δ 7.19 – 7.15 (m, 2H), 5.39 (d, *J* = 2.3 Hz, 1H), 4.91 (d, *J* = 2.3 Hz, 1H), 2.37 (s, 3H); ^{13}C NMR (101 MHz, $CDCl_3$) δ 145.2, 139.3, 131.7, 129.3, 125.6, 97.3, 21.4; HRMS (EI) *m/z* calcd for $C_9H_9N_3$ $[M]^+$: 159.0796, found 159.0792.

1-(1-azidovinyl)-3,5-dimethoxybenzene (**2c**): Prepared according to the general procedure. Yield: 202 mg

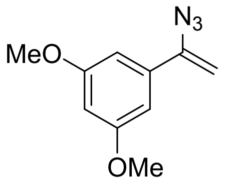

colorless oil; 49%; ^{1}H NMR (400 MHz, $CDCl_3$) δ 6.71 (d, *J* = 2.2 Hz, 2H), 6.46 (t, *J* = 2.3 Hz, 1H), 5.41 (d, *J* = 2.3 Hz, 1H), 4.95 (d, *J* = 2.4 Hz, 1H), 3.81 (s, 6H); ^{13}C NMR (101 MHz, $CDCl_3$) δ 160.9, 145.0, 136.4, 103.9, 101.4, 98.6, 55.5; HRMS (ESI^+) *m/z* calcd for $C_{10}H_{12}N_3O_2$ $[M+H]^+$: 206.0924, found 206.0928.

1-(1-azidovinyl)-2-methoxybenzene (**2d**): Prepared according to the general procedure. Yield: 149 mg

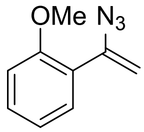

colorless oil; 43%; ^{1}H NMR (401 MHz, $CDCl_3$) δ 7.40 – 7.33 (m, 2H), 7.01 – 6.94 (m, 2H), 5.04 (d, *J* = 1.0 Hz, 1H), 4.94 (d, *J* = 1.0 Hz, 1H), 3.90 (s, 3H); ^{13}C NMR (101 MHz, $CDCl_3$) δ 156.9, 143.2, 130.8, 130.5, 123.8, 120.9, 111.0, 103.2, 55.8; HRMS (ESI^+) *m/z* calcd for $C_9H_{10}N_3O$ $[M+H]^+$: 176.0824, found 176.0823.

1-(1-azidovinyl)-4-fluorobenzene (**2e**):

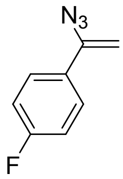

Prepared according to the general procedure. Yield: 287.0 mg pale yellow oil; 44%; ^{1}H NMR (401 MHz, CDCl3) δ 7.56 – 7.51 (m, 2H), 7.07 – 7.00 (m, 2H), 5.38 (d, *J* = 2.5 Hz, 1H), 4.94 (d, *J* = 2.5 Hz, 1H); ^{13}C NMR (101 MHz, $CDCl_3$) δ 164.6, 162.2, 144.3, 130.6 (d, *J* = 3.3 Hz), 127.6 (d, *J* = 8.2 Hz), 115.5 (d, *J* = 21.7 Hz), 97.8 (d, *J* = 1.6 Hz); ^{19}F NMR (377 MHz, $CDCl_3$) δ -112.8 (tt, *J* = 8.4, 5.3 Hz, 1F); HRMS (ESI^+) *m/z* calcd for $C_8H_7N_3F$ $[M+H]^+$: 164.06185, found 164.06192.

Preparation and characterization of alkyne **3**

tert-butyl (1-phenylprop-2-yn-1-yl) carbonate (**3a**): Under air atmosphere, di-tert-butyl dicarbonate (1.6 mL,

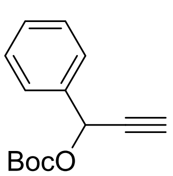

6.8 mmol, 1.5 equiv.) was added to a 50 mL round-bottom flask with a stirred solution of 1-phenylprop-2-yn-1-ol (594 mg, 4.53 mmol, 1.0 equiv.) in THF (9 mL) at 0°C, followed by the addition of DMAP (55 mg, 0.45 mmol, 0.1 equiv.) Then the reaction was allowed to proceed at room temperature for 22 h. The reaction mixture was filtrated via paper, washed by Et_2O, evaporated with Celite and purified by column chromatography (PET/EtOAc 5:1) to obtain alkyne

3a. Yield: 951 mg yellow oil; 97%; ^{1}H NMR (400 MHz, $CDCl_3$) δ 7.57 – 7.53 (m, 2H), 7.42 – 7.36 (m, 3H), 6.24 (d, *J* = 2.3 Hz, 1H), 2.69 (d, *J* = 2.3 Hz, 1H), 1.49 (s, 9H); ^{13}C NMR (101 MHz, $CDCl_3$) δ 152.6, 136.3, 129.3, 128.8, 127.8, 83.3, 80.1, 76.1, 68.3, 27.9; HRMS (ESI$^+$) *m/z* calcd for $C_{14}H_{16}O_3Na$ $[M+Na]^+$: 255.0992, found 255.0995.

Optimization of the reaction conditions leading to triazoles **4**

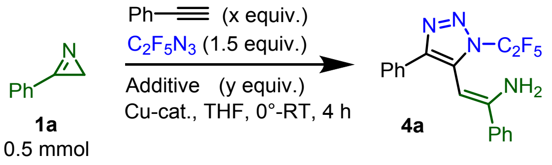

Entry	Alkyne (x equiv.)	Additive (y equiv.)	Catalyst (10 mol%)	Yield of **4a**[a]
1[c]	1.0	DIPEA (1.2)	–	21[b]
2	1.3	DIPEA (2.2)	CuI	46 [b]
3	1.3	DIPEA (4.4)	CuI	41
4	1.3	DIPEA (2.2)	CuI[d]	39
5	1.1	DIPEA (2.2)	CuI	43
6	1.5	DIPEA (2.2)	CuI	43
7	1.3	Et_3N (2.2)	CuI	43
8	1.3	Pyridine (2.2)	CuI	31
9	1.3	NaOEt (2.2)	CuI	31
10	1.3	KF (2.2)	CuI	25
11	1.3	$NaNH_2$ (2.2)	CuI	0
12	1.3	tBuOK (2.2)	CuI	0
13	1.3	NaOH (2.2)	CuI	0
16[e]	1.3	DIPEA (2.2)	CuI	49
17[e]	1.3	DIPEA (2.2)	CuCl	57
18[e]	1.3	DIPEA (2.2)	CuBr	22
19[e]	1.3	DIPEA (2.2)	$CuCO_3$	1
20[e]	1.3	DIPEA (1.2)	CuCl	39
21[e]	1.3	DIPEA (2.2)	CuCl	60 (**61**[b])
22[e]	1.3	DIPEA (3.2)	CuCl	27
23[e]	1.3	DIPEA (2.2)	CuBr	61

[a] ^{19}F NMR yield. [b] Isolated yield. [c] Copper(I) acetylide instead of alkyne. [d] 50mol%. [e] 720mg 3 Å molecular sieves.

Preparation and characterization of triazoles **4**

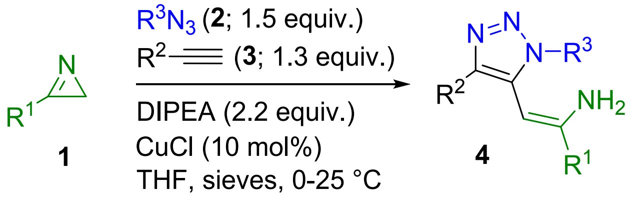

Safety note: Fluorinated azides used in this study are stable commercial-available compounds, which passed safety tests for commercialization and can be safely handled in solution at ambient temperature.

General procedure: Under air atmosphere, 10 mL screw-cap glass tube was charged with corresponding 2*H*-azirine **1** (0.5 mmol, 1.0 equiv.) and alkyne **3** (0.65 mmol, 1.3 equiv.). The reaction was cooled to 0°C and a solution of azide **2** in THF (~0.75 mmol, 4 mL) was added carefully. Finally, 3Å molecular sieves (720 mg), CuCl (0.050 mmol, 0.10 equiv.) and DIPEA (1.1 mmol, 2.2 equiv.) were added to the reaction solution and stirred at 0°C for 30 minutes. Then, the reaction mixture was stirred at room temperature for another 3.5 hours. After the reaction was finished, the suspension was filtered via paper, washed by Et_2O (2 x 10 mL), evaporated and purified by column chromatography (pentane/DCM; 4:1) to obtain triazole **4**.

(Z)-2-(1-(perfluoroethyl)-4-phenyl-1H-1,2,3-triazol-5-yl)-1-phenylethen-1-amine (**4a**):

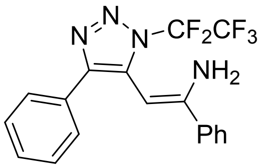

Prepared according to the general procedure. Yield: 115.5 mg colorless oil; 61%. Scale-up reaction: Under air atmosphere, 10 mL screw-cap glass tube was charged with corresponding 2*H*-azirine **1** (6.54 mmol, 1.0 equiv.) and alkyne **3** (8.50 mmol, 1.3 equiv.). The reaction was cooled to 0°C and a solution of azide **2** in THF (~9.81 mmol, 55 mL) was added carefully. Finally, 3Å molecular sieves (9.5 g), CuCl (0.050 mmol, 0.10 equiv.) and DIPEA (14.39 mmol, 2.2 equiv.) were added to the reaction solution and stirred at 0°C for 30 minutes. Then, the reaction mixture was stirred at room temperature for another 3.5 hours. After the reaction was finished, the suspension was filtered via paper, washed by Et_2O (2 x 50 mL), evaporated and purified by column chromatography (pentane/DCM; 4:1) to obtain triazole **4a**; yield: 1.293 g yellow oil; 52%. ^{1}H NMR (400 MHz, $CDCl_3$) δ 7.97 – 7.92 (m, 1H), 7.60 – 7.56 (m, 1H), 7.47 – 7.42 (m, 3H), 7.43 – 7.34 (m, 1H), 5.44 (t, *J* = 2.2 Hz, 1H), 3.80 (bs, 2H); ^{13}C NMR (101 MHz, $CDCl_3$) δ 148.7, 143.0, 138.2, 131.7 (d, *J* = 2.1 Hz), 130.0, 129.1, 129.0, 128.9, 126.9, 126.4, 117.1 (qt, *J* = 288.1, 39.9 Hz), 111.5 (tq, *J* = 268.4, 42.5 Hz), 109.7, 79.7 (t, *J* = 3.7 Hz); ^{19}F NMR (376 MHz, $CDCl_3$) δ –82.3 (s, 3F), –96.4 (d, *J* = 2.3 Hz, 2F); HRMS (ESI$^+$) *m/z* calcd for $C_{18}H_{14}F_5N_4$ [M+H]$^+$: 381.1133, found 381.1131.

(Z)-2-(4-(4-methoxyphenyl)-1-(perfluoroethyl)-1H-1,2,3-triazol-5-yl)-1-phenylethen-1-amine (**4b**):

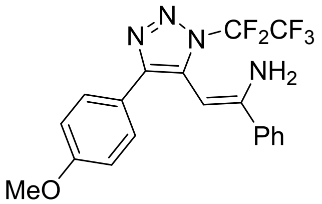

Prepared according to the general procedure. Yield: 101.0 mg yellow oil; 49%; ^{1}H NMR (400 MHz, CDCl$_3$) δ 7.92 – 7.84 (m, 2H), 7.60 – 7.56 (m, 2H), 7.47 – 7.43 (m, 3H), 6.98 – 6.95 (m, 2H), 5.42 (t, *J* = 2.2 Hz, 1H), 3.84 (s, 3H), 3.81 (bs, 2H); ^{13}C NMR (101 MHz, CDCl$_3$) δ 160.1, 148.4, 142.9, 138.2, 130.8 (t, *J* = 2.0 Hz), 129.9, 129.0, 128.4, 126.4, 122.4, 117.5 (qt, *J* = 287.9, 40.2 Hz), 114.3, 111.5 (tq, *J* = 268.6, 42.6 Hz), 79.9 (t, *J* = 3.5 Hz), 55.4; ^{19}F NMR (376 MHz, CDCl$_3$) δ –82.3 (s, 3F), –96.4 (d, *J* = 2.3 Hz, 2F); HRMS (ESI$^+$) *m/z* calcd for $C_{19}H_{16}F_5N_4O$ [M+H]$^+$: 411.1239, found 411.1241.

(Z)-4-(5-(2-amino-2-phenylvinyl)-1-(perfluoroethyl)-1H-1,2,3-triazol-4-yl)-N,N-dimethylaniline (**4c**):

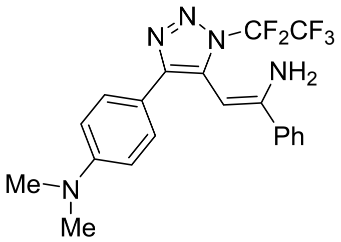

Prepared according to the general procedure. Yield: 130.3 mg yellow oil; 62%; ^{1}H NMR (401 MHz, CDCl$_3$) δ 7.84 – 7.80 (m, 2H), 7.61 – 7.57 (m, 2H), 7.46 – 7.43 (m, 3H), 6.77 – 6.74 (m, 2H), 5.44 (t, *J* = 2.1 Hz, 1H), 3.83 (bs, 2H), 3.00 (s, 6H); ^{13}C NMR (101 MHz, CDCl$_3$) δ 150.5, 147.9, 143.3, 138.3, 129.8 (t, *J* = 1.8 Hz), 129.6, 128.9, 127.9, 126.3, 117.4 (qt, *J* = 287.9, 40.2 Hz), 117.4, 112.1, 111.4 (tq, *J* = 42.5 Hz), 80.4 (t, *J* = 3.7 Hz), 40.3; ^{19}F NMR (377 MHz, CDCl$_3$) δ –82.3 (s, 3F), –96.2 (d, *J* = 2.1 Hz, 2F); HRMS (ESI$^+$) *m/z* calcd for $C_{20}H_{19}F_5N_5$ [M+H]$^+$: 424.1555, found 424.1557.

(Z)-2-(1-(perfluoroethyl)-4-(p-tolyl)-1H-1,2,3-triazol-5-yl)-1-phenylethen-1-amine (**4d**):

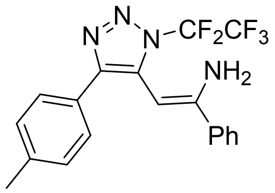

Prepared according to the general procedure. Yield: 145.5 mg yellow oil; 74%; ^{1}H NMR (400 MHz, CDCl$_3$) δ 7.88 – 7.79 (m, 2H), 7.65 – 7.56 (m, 2H), 7.47 – 7.43 (m, 3H), 7.28 – 7.26 (m, 2H), 5.43 (t, *J* = 2.1 Hz, 1H), 3.80 (bs, 2H), 2.39 (s, 3H); ^{13}C NMR (101 MHz, CDCl$_3$) δ 148.5, 143.1, 138.9, 138.3, 131.3, 129.9, 129.6, 129.0, 127.1, 126.8, 126.4, 117.5 (qt, *J* = 287.6, 40.0 Hz), 111.5 (tq, *J* = 268.3, 42.5 Hz), 79.9 (t, *J* = 3.7 Hz), 21.5; ^{19}F NMR (376 MHz, CDCl$_3$) δ –82.3 (s, 3F), –96.4 (d, *J* = 2.3 Hz, 2F); HRMS (ESI$^+$) *m/z* calcd for $C_{19}H_{16}F_5N_4$ [M+H]$^+$: 395.1290, found 395.1289.

(Z)-2-(4-([1,1'-biphenyl]-4-yl)-1-(perfluoroethyl)-1H-1,2,3-triazol-5-yl)-1-phenylethen-1-amine (**4e**):

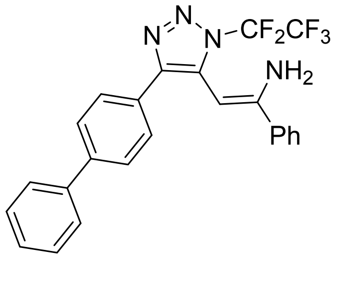

Prepared according to the general procedure. Yield: 136.0 mg yellow oil; 60%; ^{1}H NMR (401 MHz, CDCl$_3$) δ 8.07 – 8.02 (m, 2H), 7.71 – 7.68 (m, 2H), 7.66 – 7.60 (m, 4H), 7.49 – 7.43 (m, 5H), 7.40 – 7.35 (m, 1H), 5.47 (t, *J* = 2.1 Hz, 1H), 3.88 (bs, 2H); ^{13}C NMR (101 MHz, CDCl$_3$) δ 148.7, 142.7, 141.5, 140.4, 138.1, 131.6 (t, *J* = 1.8 Hz), 130.0, 129.1, 129.0, 128.8, 127.8, 127.4, 127.3, 127.1, 126.4, 117.7 (qt, *J* = 287.7, 40.0 Hz), 111.5 (tq, *J* = 268.9, 42.7

Hz), 79.7 (t, J = 3.5 Hz); ^{19}F NMR (376 MHz, $CDCl_3$) δ –82.3 (s, 3F), –96.4 (d, J = 1.7 Hz, 2F); HRMS (ESI$^+$) *m/z* calcd for $C_{24}H_{18}F_5N_4$ [M+H]$^+$: 457.1446, found 457.1448.

(Z)-2-(4-(4-fluorophenyl)-1-(perfluoroethyl)-1H-1,2,3-triazol-5-yl)-1-phenylethen-1-amine (**4f**):

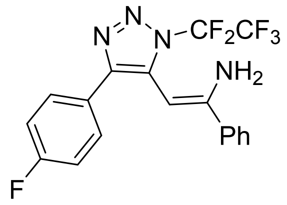

Prepared according to the general procedure. Yield: 121.6 mg yellow oil; 61%; ^{1}H NMR (401 MHz, $CDCl_3$) δ 7.97 – 7.92 (m, 2H), 7.60 – 7.56 (m, 2H), 7.48 – 7.43 (m, 3H), 7.16 – 7.10 (m, 2H), 5.41 (t, J = 2.1 Hz, 1H), 3.83 (bs, 2H); ^{13}C NMR (101 MHz, $CDCl_3$) δ 163.1 (d, J = 248.7 Hz), 148.7, 142.3, 138.0, 131.4, 130.1, 129.1, 128.8 (d, J = 8.4 Hz), 126.3, 126.1 (d, J = 3.3 Hz), 117.5 (qt, J = 287.9, 40.0 Hz), 115.9 (d, J = 21.6 Hz), 111.4 (tq, J = 268.7, 42.7 Hz), 79.4 (t, J = 3.5 Hz); ^{19}F NMR (377 MHz, $CDCl_3$) δ –82.4 (s, 3F), –96.5 (d, J = 2.5 Hz, 2F), –112.5 (tt, J = 8.6, 5.4 Hz, 1F); HRMS (ESI$^+$) *m/z* calcd for $C_{18}H_{13}F_6N_4$ [M+H]$^+$: 399.1039, found 399.1042.

methyl (Z)-4-(5-(2-amino-2-phenylvinyl)-1-(perfluoroethyl)-1H-1,2,3-triazol-4-yl)benzoate (**4g**):

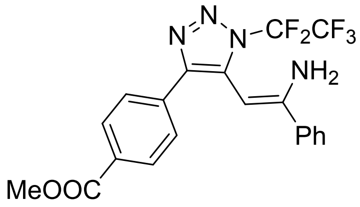

Prepared according to the general procedure. Yield: 139.0 mg pale yellow oil; 63%; ^{1}H NMR (400 MHz, $CDCl_3$) δ 8.11 – 8.08 (m, 2H), 8.05 – 8.02 (m, 2H), 7.60 – 7.56 (m, 2H), 7.49 – 7.42 (m, 3H), 5.43 (t, J = 2.2 Hz, 1H), 3.92 (s, 3H), 3.86 (bs, 2H); ^{13}C NMR (101 MHz, $CDCl_3$) δ 166.7, 149.1, 141.9, 137.8, 134.2, 132.5 (t, J = 1.8 Hz), 130.1, 129.9, 129.0, 126.5, 126.2, 117.3 (qt, J = 287.9, 40.0 Hz), 111.3 (tq, J = 269.2, 42.7 Hz), 79.1 (t, J = 3.5 Hz), 52.2; ^{19}F NMR (376 MHz, $CDCl_3$) δ –82.4 (s, 3F), –96.6 (d, J = 2.3 Hz, 2F); HRMS (ESI$^+$) *m/z* calcd for $C_{20}H_{16}O_2F_5N_4$ [M+H]$^+$: 439.1188, found 439.1187.

(Z)-2-(4-(3,5-dimethoxyphenyl)-1-(perfluoroethyl)-1H-1,2,3-triazol-5-yl)-1-phenylethen-1-amine (**4h**):

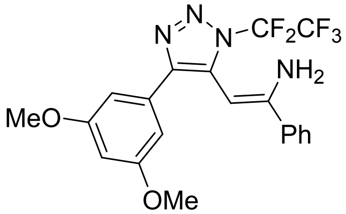

Prepared according to the general procedure. Yield: 125.5 mg yellow oil; 57%; ^{1}H NMR (401 MHz, $CDCl_3$) δ 7.59 – 7.56 (m, 2H), 7.46 – 7.43 (m, 3H), 7.15 (d, J = 2.3 Hz, 2H), 6.49 (t, J = 2.0 Hz, 1H), 5.41 (t, J = 2.2 Hz, 1H), 3.88 (bs, 2H), 3.78 (s, 6H); ^{13}C NMR (101 MHz, $CDCl_3$) δ 161.1, 149.0, 143.0, 138.2, 131.9, 131.6, 130.0, 129.1, 126.4, 117.5 (qt, J = 288.2, 40.1 Hz), 111.5 (tq, J = 268.2, 42.6 Hz), 104.6, 101.8, 79.6 (t, J = 3.7 Hz), 55.5; ^{19}F NMR (377 MHz, $CDCl_3$) δ –82.3 (s, 3F), –96.4 (d, J = 2.1 Hz, 2F); HRMS (ESI$^+$) *m/z* calcd for $C_{20}H_{18}F_5N_4O_2$ [M+H]$^+$: 441.1344, found 441.1344.

(Z)-2-(1-(perfluoroethyl)-4-(thiophen-3-yl)-1H-1,2,3-triazol-5-yl)-1-phenylethen-1-amine (**4i**):

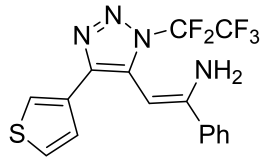

Prepared according to the general procedure. Yield: 136.5 mg pale yellow oil; 71%; ^{1}H NMR (400 MHz, $CDCl_3$) δ 7.79 (dt, *J* = 2.9, 1.0 Hz, 1H), 7.74 (dt, *J* = 5.0, 0.9 Hz, 1H), 7.66 – 7.62 (m, 2H), 7.51 – 7.47 (m, 3H), 7.41 (ddd, *J* = 5.1, 3.0, 0.6 Hz, 1H), 5.42 (t, *J* = 2.1 Hz, 1H), 3.93 (bs, 2H); ^{13}C NMR (101 MHz, $CDCl_3$) δ 149.1, 140.4, 138.0, 130.8 (t, *J* = 1.8 Hz), 130.5, 130.1, 129.1, 126.6, 126.4, 126.0, 123.6, 117.5 (qt, *J* = 287.9, 40.3 Hz), 111.4 (tq, *J* = 269.0, 42.6 Hz), 79.7 (t, *J* = 3.3 Hz); ^{19}F NMR (376 MHz, $CDCl_3$) δ –82.6 (s, 3F), –96.7 (d, *J* = 1.7 Hz, 2F); HRMS (ESI$^+$) *m/z* calcd for $C_{16}H_{12}F_5N_4S$ $[M+H]^+$: 387.0697, found 387.0698.

(Z)-2-(1-(perfluoroethyl)-4-(pyridin-3-yl)-1H-1,2,3-triazol-5-yl)-1-phenylethen-1-amine (**4j**):

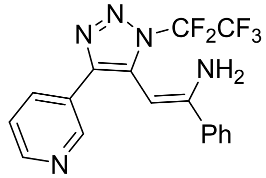

Prepared according to the general procedure. Yield: 182.3 mg colorless oil; 96%; ^{1}H NMR (400 MHz, $CDCl_3$) δ 9.16 – 9.14 (m, 1H), 8.60 (dd, *J* = 4.9, 1.7 Hz, 1H), 8.27 (ddd, *J* = 7.9, 2.3, 1.6 Hz, 1H), 7.59 – 7.56 (m, 2H), 7.47 – 7.43 (m, 3H), 7.38 (ddd, *J* = 7.9, 4.8, 0.9 Hz, 1H), 5.43 (t, *J* = 2.1 Hz, 1H), 3.91 (bs, 2H); ^{13}C NMR (101 MHz, $CDCl_3$) δ 149.8, 149.2, 148.1, 140.3, 137.7, 134.0, 132.4, 130.2, 129.1, 126.4, 126.2, 123.6, 117.4 (qt, *J* = 287.9, 39.8 Hz), 111.4 (tq, *J* = 269.3, 42.8 Hz), 79.0 (t, *J* = 3.5 Hz); ^{19}F NMR (376 MHz, $CDCl_3$) δ –82.4 (s, 3F), –96.6 (s, 2F); HRMS (ESI$^+$) *m/z* calcd for $C_{17}H_{13}F_5N_5$ $[M+H]^+$: 382.1086, found 382.1088.

(Z)-2-(4-(cyclohex-1-en-1-yl)-1-(perfluoroethyl)-1H-1,2,3-triazol-5-yl)-1-phenylethen-1-amine (**4k**):

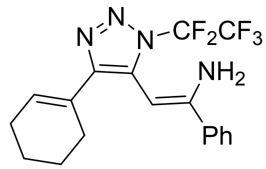

Prepared according to the general procedure. Yield: 118.4 mg yellow oil; 61%; ^{1}H NMR (400 MHz, $CDCl_3$) δ 7.59 – 7.53 (m, 2H), 7.46 – 7.42 (m, 3H), 6.35 (tt, *J* = 3.9, 1.8 Hz, 1H), 5.34 (t, *J* = 2.1 Hz, 1H), 4.09 (bs, 2H), 2.54 – 2.49 (m, 2H), 2.28 – 2.22 (m, 2H), 1.80 – 1.73 (m, 2H), 1.72 – 1.66 (m, 2H); ^{13}C NMR (101 MHz, $CDCl_3$) δ 148.3, 145.1, 138.4, 130.7 (t, *J* = 1.7 Hz), 129.8, 129.7, 129.0, 128.4, 126.4, 117.5 (qt, *J* = 287.3, 40.3 Hz), 111.5 (tq, *J* = 267.8, 42.2 Hz), 80.6 (t, *J* = 3.9 Hz), 26.4, 25.9, 22.8, 22.1; ^{19}F NMR (376 MHz, $CDCl_3$) δ –82.3 (s, 3F), –96.3 (d, *J* = 2.3 Hz, 2F); HRMS (ESI$^+$) *m/z* calcd for $C_{18}H_{18}F_5N_4$ $[M+H]^+$: 385.1446, found 385.1449.

(Z)-2-(4-butyl-1-(perfluoroethyl)-1H-1,2,3-triazol-5-yl)-1-phenylethen-1-amine (**4l**):

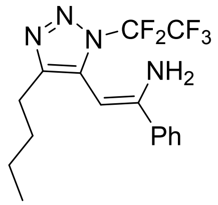

Prepared according to the general procedure. Yield: 92.7 mg pale yellow oil; 52%; ^{1}H NMR (400 MHz, $CDCl_3$) δ 7.64 – 7.60 (m, 2H), 7.48 – 7.44 (m, 3H), 5.19 (t, *J* = 2.0 Hz, 1H), 3.93 (bs, 2H), 2.75 (dd, *J* = 8.5, 6.9 Hz, 2H), 1.81 – 1.73 (m, 2H), 1.45 – 1.36 (m, 2H), 0.95 (t, *J* = 7.4 Hz, 3H); ^{13}C NMR (101 MHz, $CDCl_3$) δ 148.7, 146.2, 137.9, 131.8, 129.7, 128.8, 126.2, 117.3 (qt, *J* = 287.6, 41.1 Hz), 111.1 (tq, *J* = 267.8, 42.6 Hz), 80.3

(t, J = 2.9 Hz), 30.6, 25.3, 22.4, 13.8; ^{19}F NMR (376 MHz, $CDCl_3$) δ –82.9 (s, 3F), –97.0 (s, 2F); HRMS (ESI$^+$) *m/z* calcd for $C_{16}H_{18}F_5N_4$ [M+H]$^+$: 361.1446, found 361.1447.

(Z)-2-(1-(difluoromethyl)-4-(p-tolyl)-1H-1,2,3-triazol-5-yl)-1-phenylethen-1-amine (**4m**):

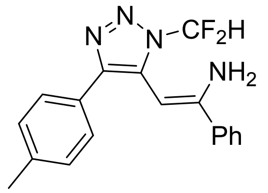

Prepared according to the general procedure. Yield: 110.0 mg pale yellow oil; 67%; ^{1}H NMR (400 MHz, $CDCl_3$) δ 7.83 – 7.80 (m, 2H), 7.64 – 7.60 (m, 2H), 7.54 (t, J = 58.0 Hz, 1H), 7.47 – 7.43 (m, 3H), 7.26 – 7.22 (m, 2H), 5.53 (s, 1H), 3.81 (s, 2H), 2.39 (s, 3H); ^{13}C NMR (101 MHz, $CDCl_3$) δ 148.2, 143.4, 138.6, 138.3, 129.8, 129.6 (t, J = 1.8 Hz), 129.5, 129.0, 127.6, 126.9, 126.4, 110.9 (t, J = 253.1 Hz), 80.3 (t, J = 2.0 Hz), 21.5; ^{19}F NMR (376 MHz, $CDCl_3$) δ –98.0 (d, J = 57.8 Hz); HRMS (ESI$^+$) *m/z* calcd for $C_{18}H_{17}N_4F_2$ [M+H]$^+$: 327.1416, found 327.1415.

(Z)-1-phenyl-2-(4-(p-tolyl)-1-(trifluoromethyl)-1H-1,2,3-triazol-5-yl)ethen-1-amine (**4n**):

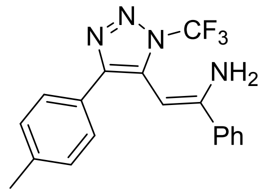

Prepared according to the general procedure. Yield: 152.0 mg colorless oil; 88%; ^{1}H NMR (401 MHz, $CDCl_3$) δ 7.84 – 7.80 (m, 2H), 7.60 – 7.57 (m, 2H), 7.45 (dd, J = 5.1, 1.9 Hz, 3H), 7.27 – 7.23 (m, 2H), 5.41 (d, J = 1.6 Hz, 1H), 3.82 (bs, 2H), 2.39 (s, 3H); ^{13}C NMR (101 MHz, $CDCl_3$) δ 148.7, 143.2, 138.9, 138.2, 130.1, 130.0, 129.6, 129.0, 127.2, 126.9, 126.4, 118.4 (q, J = 268.3 Hz), 79.5 (q, J = 1.8 Hz), 21.5; ^{19}F NMR (377 MHz, $CDCl_3$) δ –58.3 (d, J = 1.4 Hz); HRMS (ESI$^+$) *m/z* calcd for $C_{18}H_{16}N_4F_3$ [M+H]$^+$: 345.1322, found 345.1321.

(Z)-1-phenyl-2-(1-(1,1,2,2-tetrafluoroethyl)-4-(p-tolyl)-1H-1,2,3-triazol-5-yl)ethen-1-amine (**4o**):

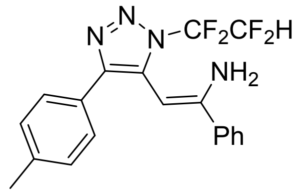

Prepared according to the general procedure. Yield: 140.0 mg colorless oil; 74%; ^{1}H NMR (400 MHz, $CDCl_3$) δ 7.81 (d, J = 8.2 Hz, 2H), 7.60 (ddd, J = 5.7, 2.4, 1.3 Hz, 2H), 7.47 – 7.42 (m, 3H), 7.27 – 7.23 (m, 2H), 6.83 (tt, J = 52.3, 4.8 Hz, 1H), 5.52 (t, J = 2.0 Hz, 1H), 3.80 (bs, 2H), 2.39 (s, 3H); ^{13}C NMR (101 MHz, $CDCl_3$) δ 148.2, 143.2, 138.9, 138.3, 131.1 (t, J = 2.0 Hz), 129.9, 129.5, 129.0, 127.2, 126.9, 126.4, 113.3 (tt, J = 264.12, 28.4 Hz), 107.9 (tt, J = 253.5, 32.1 Hz), 80.3 (t, J = 3.3 Hz), 21.5; ^{19}F NMR (376 MHz, $CDCl_3$) δ –98.6 (q, J = 8.6 Hz, 2F), –137.2 (dt, J = 52.6, 8.6 Hz, 2F); HRMS (ESI$^+$) *m/z* calcd for $C_{19}H_{17}N_4F_4$ [M+H]$^+$: 377.1384, found 377.1383.

8,8-difluoro-5-phenyl-3-(p-tolyl)-6H-[1,2,3]triazolo[1,5-d][1,4]diazepin-7(8H)-one (**4p**):

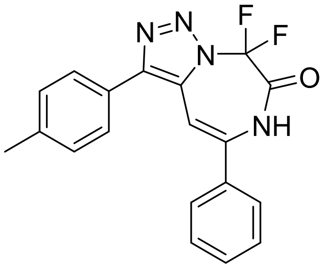

Prepared according to the general procedure from $N_3CF_2CO_2Et$. Then it was evaporated with Celite and purified by Combi flash chromatography (cyclohexane/EtOAc) to obtain **4p**. Yield: 73.0 mg white solid; 43%; melting point: 166–167 °C; ^{1}H NMR (401 MHz, Acetone-d_6) δ 10.33 (bs, 1H), 7.87 – 7.82 (m, 4H), 7.55 – 7.52 (m, 3H), 7.40 (dt, J = 7.9, 0.8 Hz, 2H), 6.88 (s, 1H), 2.42 (s, 3H); ^{13}C NMR (101 MHz, Acetone-d_6) δ 157.6 (t, J = 35.2 Hz), 145.2, 140.3, 139.7, 136.5, 131.4, 130.7, 129.9, 128.8, 128.4, 128.3, 128.2, 110.3 (t, J = 264.6 Hz), 99.4, 21.4; ^{19}F NMR (377 MHz, Acetone-d_6) δ –93.0 (s); HRMS (ESI$^+$) *m/z* calcd for $C_{19}H_{15}F_2N_4O$ [M+H]$^+$: 353.1208, found 353.1210.

5-phenyl-3-(p-tolyl)-[1,2,3]triazolo[1,5-c]pyrimidin-7-ol (**4q**):

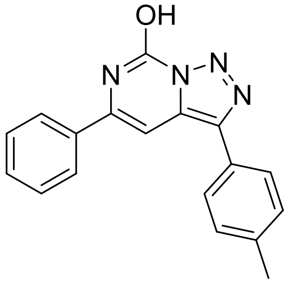

Prepared according to the general procedure from $N_3CF_2SO_2Ph$. Then it was evaporated with Celite and purified by Combi flash chromatography (cyclohexane/EtOAc) to obtain **4q**. Yield: 90.0 mg white solid; 60%; melting point: 95.5–96.0 °C; ^{1}H NMR (401 MHz, DMSO-d_6) δ 12.17 (s, 1H), 7.91 – 7.85 (m, 4H), 7.54 – 7.51 (m, 3H), 7.31 (d, J = 8.2 Hz, 2H), 7.23 (s, 1H), 2.36 (s, 3H); ^{13}C NMR (101 MHz, DMSO-d_6) δ 144.3, 141.8, 138.8, 137.5, 132.0, 131.6, 130.2, 129.6, 128.8, 127.5, 127.4, 126.1, 93.3, 20.9; HRMS (ESI$^+$) *m/z* calcd for $C_{18}H_{15}ON_4$ [M+H]$^+$: 303.1240, found 303.1238.

(Z)-N-((5-(2-amino-2-phenylvinyl)-1-benzyl-1H-1,2,3-triazol-4-yl)(phenyl)methyl)-4-methylaniline (**4r**):

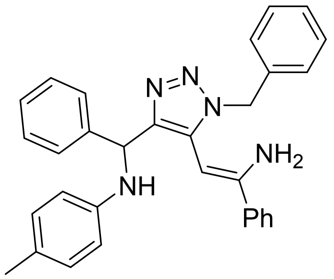

Prepared according to literature[30] from azirine **1a** (1.067 mmol, 2.0 equiv.), *p*-toluidine (0.534 mmol, 1.0 equiv.), alkyne **3a** (0.534 mmol), benzyl azide (0.64 mmol, 1.2 equiv.), DIPEA (1.067 mmol, 2.0 equiv.) and CuI (0.053 mmol, 0.1 equiv.) in MeOH (5.5 mL). Yield: 138.5 mg yellow oil; 55%; ^{1}H NMR (400 MHz, $CDCl_3$) δ 7.53 – 7.48 (m, 5H), 7.43 (dd, J = 5.0, 2.0 Hz, 3H), 7.30 – 7.28 (m, 4H), 7.27 (s, 1H), 7.19 – 7.16 (m, 3H), 6.91 (d, J = 7.8 Hz, 2H), 6.58 (d, J = 8.6 Hz, 2H), 5.77 (s, 1H), 5.47 (d, J = 15.2 Hz, 1H), 5.39 (d, J = 15.2 Hz, 1H), 4.92 (s, 1H), 3.92 (s, 2H), 2.19 (s, 3H); ^{13}C NMR (101 MHz, $CDCl_3$) δ 149.3, 146.0, 144.6, 141.5, 138.2, 135.5, 130.7, 129.8, 129.6, 128.9 (2C), 128.6, 128.2, 127.7, 127.4, 127.3, 127.2, 126.2, 114.1, 81.1, 54.3, 52.1, 20.5: HRMS (ESI$^+$) *m/z* calcd for $C_{31}H_{29}N_5$ [M+Na]$^+$: 494.2315, found 494.2313.

(Z)-1-phenyl-2-(4-(p-tolyl)-1-tosyl-1H-1,2,3-triazol-5-yl)ethen-1-amine (**4s**):

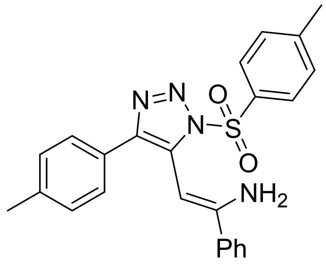

Prepared according to literature[31] from azirine **1a** (2.00 mmol, 1.0 equiv.), alkyne (2.60 mmol, 1.3 equiv.), tosyl azide (2.40 mmol, 1.2 equiv.), DIPEA (2.40 mmol, 1.2 equiv.) and CuI (0.2 mmol, 0.1 equiv.) in THF (16.0 mL). Yield: 373.0 mg yellow oil; 43%; <u>Scale-up reaction:</u> from azirine **1a** (12.80 mmol, 1.0 equiv.), alkyne (16.65 mmol, 1.3 equiv.), tosyl azide (15.37 mmol, 1.2 equiv.), DIPEA (28.17 mmol, 1.2 equiv.) and CuI (1.28 mmol, 0.1 equiv.) in THF (96.0 mL). Yield: 2.250 g yellow oil; 41%. ^{1}H NMR (401 MHz, $CDCl_3$) δ 7.93 – 7.89 (m, 2H), 7.79 – 7.75 (m, 2H), 7.66 – 7.58 (m, 2H), 7.47 (dt, J = 4.4, 2.8 Hz, 3H), 7.32 – 7.26 (m, 2H), 7.19 (d, J = 7.9 Hz, 2H), 5.81 (s, 1H), 3.56 (s, 2H), 2.41 (s, 3H), 2.35 (s, 3H); ^{13}C NMR (101 MHz, $CDCl_3$) δ 147.4, 146.6, 142.4, 138.6, 138.3, 134.2, 131.2, 130.2, 129.8, 129.4, 129.0, 128.5, 127.2, 126.7, 126.2, 81.9, 21.9, 21.5; HRMS (ESI$^+$) *m/z* calcd for $C_{24}H_{22}O_2N_4NaS$ [M+Na]$^+$: 453.1356, found 453.1355.

(Z)-2-(1-(perfluoroethyl)-4-(p-tolyl)-1H-1,2,3-triazol-5-yl)-1-(p-tolyl)ethen-1-amine (**4t**):

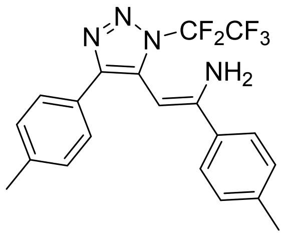

Prepared according to the general procedure. Yield: 99.0 mg yellow oil; 49%; ^{1}H NMR (400 MHz, $CDCl_3$) δ 7.85 – 7.81 (m, 2H), 7.49 – 7.46 (m, 2H), 7.26 – 7.22 (m, 4H), 5.41 (t, J = 2.2 Hz, 1H), 3.77 (s, 2H), 2.41 (s, 3H), 2.39 (s, 3H); ^{13}C NMR (101 MHz, $CDCl_3$) δ 148.3, 142.9, 140.0, 138.7, 135.2, 131.3, 129.6, 129.4, 127.0, 126.7, 126.1, 118.8 (qt, J = 289.0, 40.2 Hz), 111.3 (tq, J = 267.1, 42.5 Hz), 79.2 (t, J = 3.7 Hz), 21.4, 21.3; ^{19}F NMR (376 MHz, $CDCl_3$) δ –82.3 (s, 3F), –96.4 (d, J = 2.3 Hz, 2F); HRMS (ESI$^+$) *m/z* calcd for $C_{20}H_{18}F_5N_4$ [M+H]$^+$: 409.1446, found 409.1444.

(Z)-1-(3,5-dimethoxyphenyl)-2-(1-(perfluoroethyl)-4-(p-tolyl)-1H-1,2,3-triazol-5-yl)ethen-1-amine (**4u**):

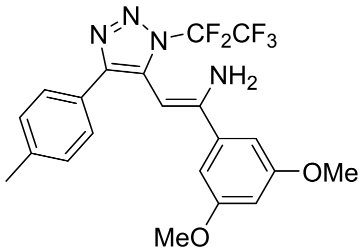

Under air atmosphere, 10 mL screw-cap glass tube was charged with corresponding 2*H*-azirine **1** (0.63 mmol, 1.0 equiv.) and alkyne **3** (0.82 mmol, 1.3 equiv.). The reaction mixture was cooled to 0°C and a solution of azide **2** in THF (~0.95 mmol, 4 mL) was added carefully. Finally, 3Å molecular sieves (720 mg), CuCl (0.063 mmol, 0.10 equiv.) and DIPEA (1.39 mmol, 2.2 equiv.) were added to the reaction solution and stirred at 0°C for 30 minutes. Then, the reaction mixture was stirred at room temperature for another 3.5 hours. After the reaction was finished, the suspension was filtered via paper, washed by Et_2O (2 x 10 mL), evaporated and purified by column chromatography (pentane/DCM; 4:1) to obtain triazole **4u**. Yield: 183.0 mg yellow oil; 64%; ^{1}H NMR (401 MHz, $CDCl_3$) δ 7.85 – 7.82 (m, 2H), 7.28 – 7.25 (m, 2H), 6.73 (d, J = 2.2 Hz, 2H), 6.55 (t, J = 2.3 Hz, 1H), 5.45 (t, J = 2.2 Hz, 1H), 3.86 (s, 6H), 3.81 (s, 2H), 2.41 (s, 3H); ^{13}C

NMR (101 MHz, $CDCl_3$) δ 161.2, 148.4, 143.1, 140.4, 138.9, 131.2, 129.6, 127.0, 126.8, 117.5 (qt, *J* = 287.9, 40.0 Hz), 111.4 (tq, *J* = 269.2, 42.6 Hz), 104.7, 101.3, 79.9 (t, *J* = 3.7 Hz), 55.6, 21.5; ^{19}F NMR (377 MHz, $CDCl_3$) δ –82.3 (s, 3F), 0–96.3 (d, *J* = 2.1 Hz, 2F); HRMS (ESI$^+$) *m/z* calcd for $C_{21}H_{20}O_2F_5N_4$ [M+H]$^+$: 455.1501, found 455.1499.

(Z)-2-(1-(perfluoroethyl)-4-(p-tolyl)-1H-1,2,3-triazol-5-yl)-1-(2-(trifluoromethyl)phenyl)ethen-1-amine (**4v**):

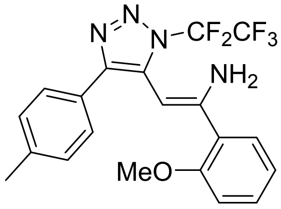

Prepared according to the general procedure. Yield: 163.5 mg yellow oil; 77%; ^1H NMR (401 MHz, $CDCl_3$) δ 7.93 (d, *J* = 8.2 Hz, 2H), 7.44 (dd, *J* = 7.5, 1.8 Hz, 1H), 7.37 (ddd, *J* = 8.4, 7.5, 1.8 Hz, 1H), 7.27 – 7.24 (m, 2H), 7.02 (td, *J* = 7.5, 1.0 Hz, 1H), 6.95 (dd, *J* = 8.4, 1.0 Hz, 1H), 5.31 (t, *J* = 2.2 Hz, 1H), 4.00 (bs, 2H), 3.87 (s, 3H), 2.39 (s, 3H); ^{13}C NMR (101 MHz, $CDCl_3$) δ 156.9, 147.5, 143.1, 138.8, 131.1, 130.6, 129.7, 129.4, 127.3, 127.1, 127.0, 121.2, 117.5 (qt, *J* = 287.9, 40.2 Hz), 114.1 (tq, *J* = 267.9, 42.2 Hz), 111.6, 81.9 (t, *J* = 3.7 Hz), 55.8, 21.5; ^{19}F NMR (377 MHz, $CDCl_3$) δ –82.3 (s, 3F), –96.43 (d, *J* = 2.5 Hz, 2F); HRMS (ESI$^+$) *m/z* calcd for $C_{20}H_{18}OF_5N_4$ [M+H]$^+$: 425.1395, found 425.1394.

(Z)-1-(4-fluorophenyl)-2-(1-(perfluoroethyl)-4-(p-tolyl)-1H-1,2,3-triazol-5-yl)ethen-1-amine (**4w**):

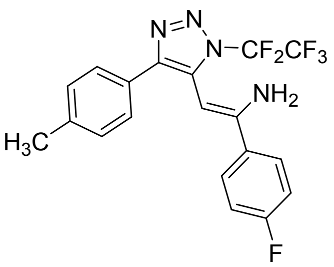

Prepared according to the general procedure. Yield: 115.0 mg pale yellow oil; 56%; ^1H NMR (401 MHz, $CDCl_3$) δ 7.84 – 7.81 (m, 2H), 7.60 – 7.55 (m, 2H), 7.28 – 7.24 (m, 2H), 7.17 – 7.11 (m, 2H), 5.38 (t, *J* = 2.2 Hz, 1H), 3.77 (s, 2H), 2.41 (s, 3H); ^{13}C NMR (101 MHz, $CDCl_3$) δ 163.8 (d, *J* = 249.8 Hz), 147.5, 143.2, 139.0, 134.4 (d, *J* = 3.3 Hz), 131.1, 129.6, 128.3 (d, *J* = 8.3 Hz), 127.0, 126.8, 117.5 (qt, *J* = 287.7, 40.3 Hz), 116.0 (d, *J* = 21.7 Hz), 111.5 (qt, *J* = 268.1, 42.6 Hz), 80.1 (t, *J* = 3.6 Hz), 21.5; ^{19}F NMR (377 MHz, $CDCl_3$) δ -82.3 (s, 3F), -96.4 (d, *J* = 2.1 Hz, 2F), -111.55 (tt, *J* = 8.5, 5.3 Hz, 1F); HRMS (ESI$^+$) *m/z* calcd for $C_{19}H_{15}N_4F_6$ [M+H]$^+$: 413.1195, found 413.1193.

Preparation and characterization of heterocycles **6**, **7**, **8**, **9**

(5-phenyl-2-(trifluoromethyl)pyrimidin-4-yl)(p-tolyl)methanone (**6a**):

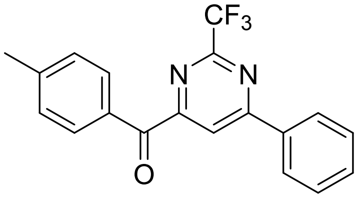

The crude reaction mixture of triazole **4d** in THF was left at room temperature for 14 days, then it was filtered via paper, washed on filter with Et_2O, evaporated and purified by column chromatography (pentane/DCM; 4:1) to obtain **6a**. Yield: 35.0 mg white solid; 31%; melting point: 92.0–93.0 °C; ^1H NMR (400 MHz, $CDCl_3$) δ 8.44 (s, 1H), 8.29 – 8.24 (m, 2H), 8.15 – 8.11 (m, 2H), 7.61 – 7.56 (m, 3H), 7.36 – 7.33 (m, 2H), 2.47 (s, 3H); ^{13}C NMR (101 MHz, $CDCl_3$) δ 190.6, 167.4, 163.5, 156.3 (q, *J* = 37.0 Hz), 145.6, 135.0, 132.6, 132.1, 131.5, 129.5, 129.5, 127.8,

119.7 (q, J = 276.2 Hz), 117.5, 22.0; ^{19}F NMR (376 MHz, $CDCl_3$) δ –70.8 (s); HRMS (EI) m/z calcd for $C_{19}H_{13}F_3N_2O$ [M+H]$^+$: 342.0970, found 342.0980.

2-(1-(difluoromethyl)-4-(p-tolyl)-1H-1,2,3-triazol-5-yl)-1-phenylethan-1-one (**7m**):

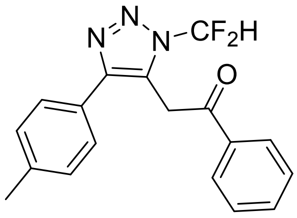

Under air atmosphere, a 10 mL screw-cap glass tube was charged with triazole **4m** (0.352 mmol, 1.0 equiv.) in 1,4-dioxane (3.5 mL) and 11M HCl (1.76 mmol, 5.0 equiv.) was added. The reaction was stirred at room temperature for 1 h. The suspension was filtered via paper, washed by Et_2O, evaporated with silica gel and purified by Combi flash chromatography (cyclohexane/EtOAc) to obtain triazole **7m**. Yield: 104.0 mg pale yellow oil; 90%; ^{1}H NMR (401 MHz, $CDCl_3$) δ 8.05 – 8.00 (m, 2H), 7.69 – 7.64 (m, 1H), 7.62 (t, J = 58.5 Hz, 1H), 7.56 – 7.51 (m, 2H), 7.49 – 7.46 (m, 2H), 7.23 (d, J = 7.7 Hz, 2H), 4.70 (s, 2H), 2.37 (s, 3H); ^{13}C NMR (101 MHz, $CDCl_3$) δ 193.0, 148.8, 138.9, 135.5, 134.2, 129.7, 129.0, 128.4, 127.6, 126.6, 126.2 (t, J = 2.4 Hz), 111.2 (t, J = 254.0 Hz), 33.4, 21.3; ^{19}F NMR (377 MHz, $CDCl_3$) δ –96.9 (d, J = 58.3 Hz); HRMS (ESI$^+$) m/z calcd for $C_{18}H_{16}ON_3F_2$ [M+H]$^+$: 328.1256, found 328.1257.

1-(3,5-dimethoxyphenyl)-2-(1-(perfluoroethyl)-4-(p-tolyl)-1H-1,2,3-triazol-5-yl)ethan-1-one (**7u**):

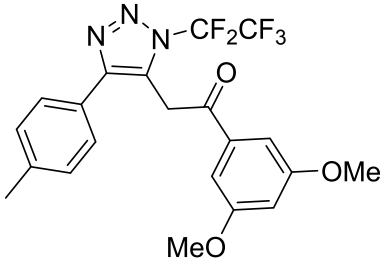

Under air atmosphere, a 10 mL screw-cap glass tube was charged with triazole **4u** (0.404 mmol, 1.0 equiv.) in 1,4-dioxane (4 mL) and 11M HCl (2.02 mmol, 5.0 equiv.) was added. The reaction was stirred at room temperature for 1 h. The suspension was filtered via paper, washed by Et_2O, evaporated with silica gel and purified by Combi flash chromatography (cyclohexane/EtOAc) to obtain triazole **7u**. Yield: 97.0 mg pale pink oil; 53%; ^{1}H NMR (400 MHz, $CDCl_3$) δ 7.44 – 7.41 (m, 2H), 7.25 – 7.22 (m, 2H), 7.12 (d, J = 2.3 Hz, 2H), 6.73 (t, J = 2.3 Hz, 1H), 4.60 (t, J = 1.8 Hz, 2H), 3.85 (s, 6H), 2.38 (s, 3H); ^{13}C NMR (101 MHz, $CDCl_3$) δ 192.5, 161.3, 148.5, 139.4, 137.1, 129.9, 128.2 (t, J = 2.2 Hz), 128.1, 126.1, 117.3 (qt, J = 287.9, 40.3 Hz), 111.40 (tq, J = 269.2, 42.9 Hz), 106.31, 106.27, 55.8, 34.4 (t, J = 3.3 Hz), 21.4; ^{19}F NMR (376 MHz, $CDCl_3$) δ –82.4 (s, 3F), –96.7 (s, 2F); HRMS (ESI$^+$) m/z calcd for $C_{21}H_{19}O_3F_5N_3$ [M+H]$^+$: 456.1341, found 456.1338.

N-(5-(3,5-dimethoxyphenyl)-2-(p-tolyl)furan-3-yl)-2,2,2-trifluoroacetamide (**8**):

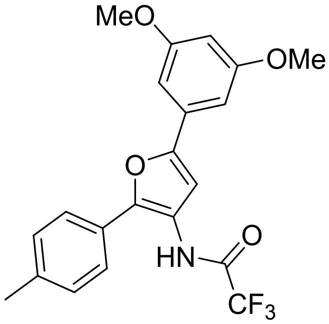

Under air atmosphere, a 10 mL screw-cap glass tube was charged with triazole **7u** (25.8 mg, 0.057 mmol, 1.0 equiv.) in DCE (1 mL) and $BF_3{\cdot}OEt_2$ (0.062 mmol, 1.1 equiv.) was added. The reaction mixture was heated on a heating block to 70 °C for 1 h. After the reaction was finished, Celite was added and DCE was evaporated from the reaction mixture. Then it was purified by Combi flash chromatography (cyclohexane/EtOAc; 4:1) to obtain **8**. Yield: 12.0 mg white solid; 52%; melting point: 138.0–140.0 °C; ^{1}H NMR (400 MHz, $CDCl_3$) δ 7.90 (bs, 1H), 7.50 – 7.47 (m, 2H), 7.32 – 7.30 (m, 2H), 7.29 (s, 1H), 6.87 (d, *J* = 2.2 Hz, 2H), 6.43 (t, *J* = 2.3 Hz, 1H), 3.85 (s, 6H), 2.42 (s, 3H); ^{13}C NMR (101 MHz, $CDCl_3$) δ 161.3, 154.91 (q, *J* = 37.8 Hz), 152.1, 142.7, 138.8, 131.7, 130.3, 126.5, 125.3, 118.9, 115.9 (q, *J* = 288.3 Hz), 104.0, 102.2, 100.6, 55.6, 21.5; ^{19}F NMR (376 MHz, $CDCl_3$) δ –76.0 (s); HRMS (ESI$^+$) *m/z* calcd for $C_{21}H_{19}O_4NF_3$ [M+H]$^+$: 406.1261, found 406.1260.

7-(3,5-dimethoxyphenyl)-4-fluoro-5-(p-tolyl)-2-(trifluoromethyl)-1,3-oxazepine (**9**):

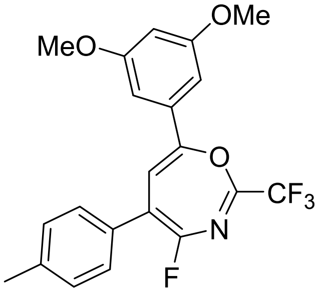

Under air atmosphere, a 10 mL microwave glass tube was charged with triazole **7u** (58.5 mg, 0.128 mmol, 1.0 equiv.) in DCE (2 mL) and CuF_2 (1.1 equiv., pre-dried on vacuum at 90 °C for 2 h). The mixture was heated in microwave in a sealed glass tube under microwave irradiation (300 W) to 165 °C for 30 min. The suspension was filtered via paper, washed on filter with Et_2O (2 x 10 mL) and evaporated to obtain **9**. Yield: 44.0 mg red oil; 84%; ^{1}H NMR (401 MHz, $CDCl_3$) δ 8.06 – 7.90 (m, 2H), 7.31 – 7.24 (m, 2H), 6.94 (d, *J* = 3.9 Hz, 1H), 6.87 (d, *J* = 2.2 Hz, 2H), 6.44 (t, *J* = 2.3 Hz, 1H), 3.86 (s, 6H), 2.41 (s, 3H); ^{13}C NMR (101 MHz, $CDCl_3$) δ 161.3, 152.0, 151.1 (d, *J* = 4.0 Hz), 139.1, 134.6 (dq, *J* = 362.0, 44.6 Hz), 131.6, 129.5, 126.5, 126.2, 122.3 (d, *J* = 8.8 Hz), 115.9 (qd, *J* = 274.0, 66.2 Hz), 104.8 (d, *J* = 5.5 Hz), 102.3, 100.5, 55.6, 21.6; ^{19}F NMR (377 MHz, $CDCl_3$) δ –53.7 (qd, *J* = 6.4, 3.9 Hz, 1F), –72.3 (d, *J* = 6.4 Hz, 3F); HRMS (ESI$^+$) *m/z* calcd for $C_{21}H_{18}O_3NF_4$ [M+H]$^+$: 408.1217, found 408.1217.

Copies of NMR spectra

Figure 8. ^1H NMR spectrum of **1a** ($CDCl_3$, 101 MHz)

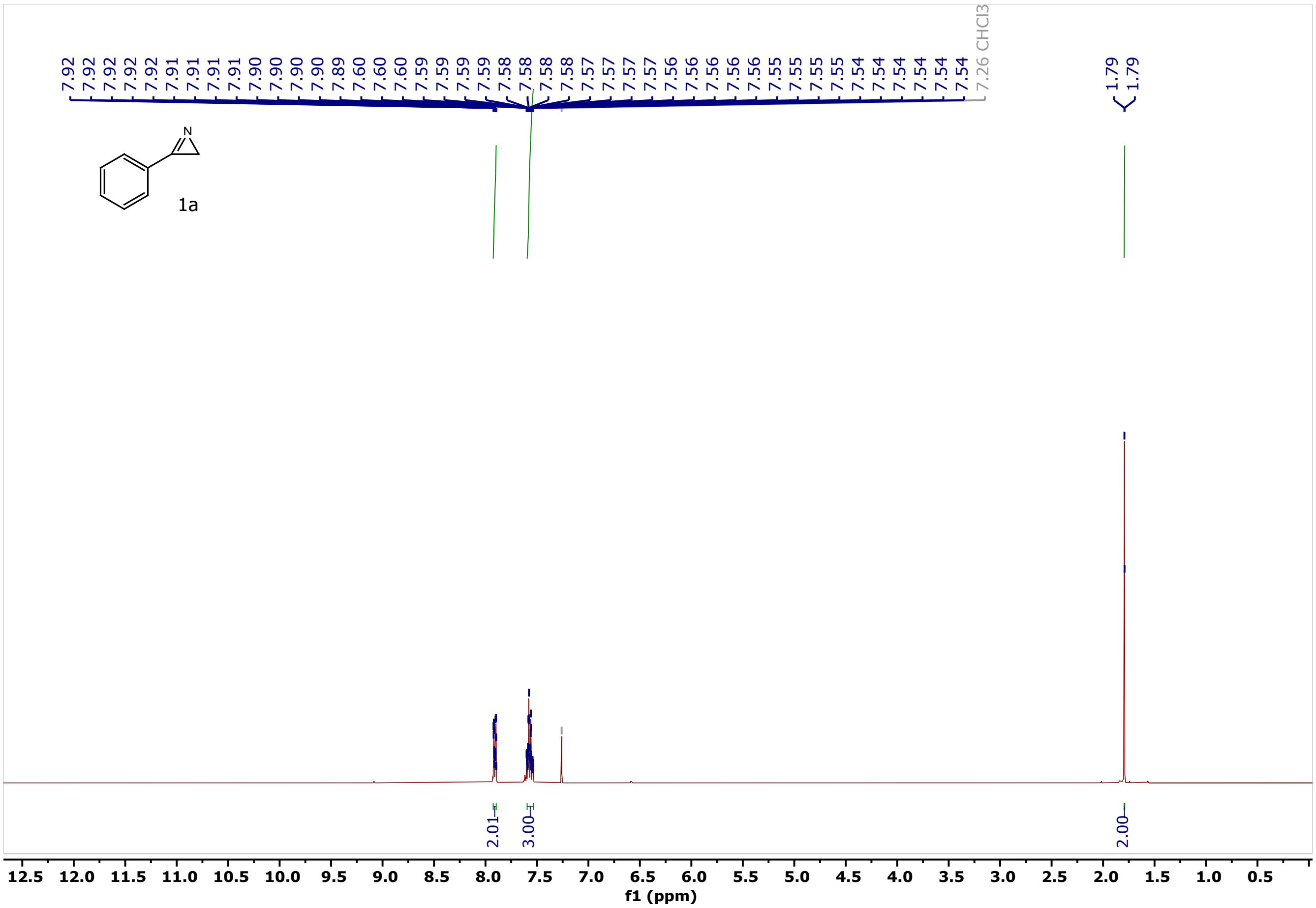

Figure 9. ^{13}C NMR spectrum of **1a** ($CDCl_3$, 101 MHz)

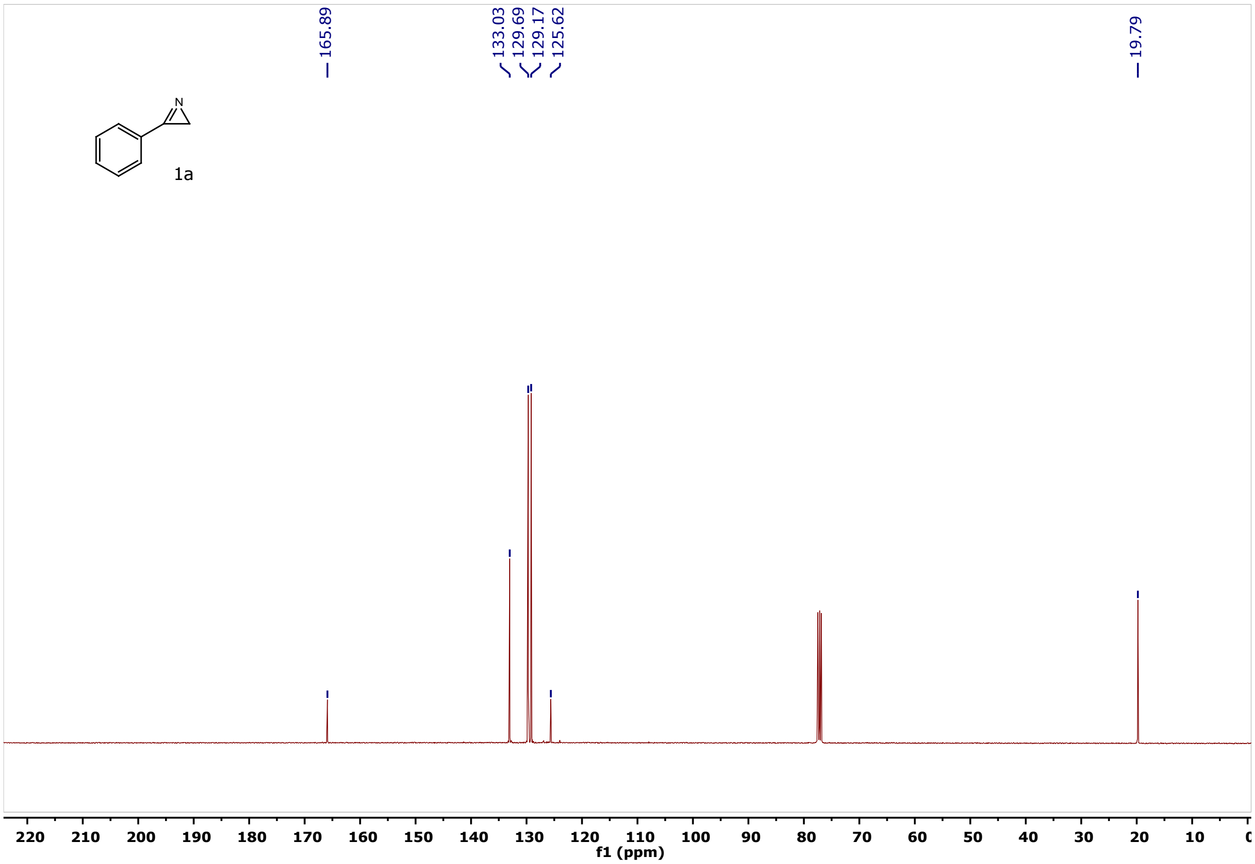

Figure 10. ^{1}H NMR spectrum of **1b** ($CDCl_3$, 400 MHz)

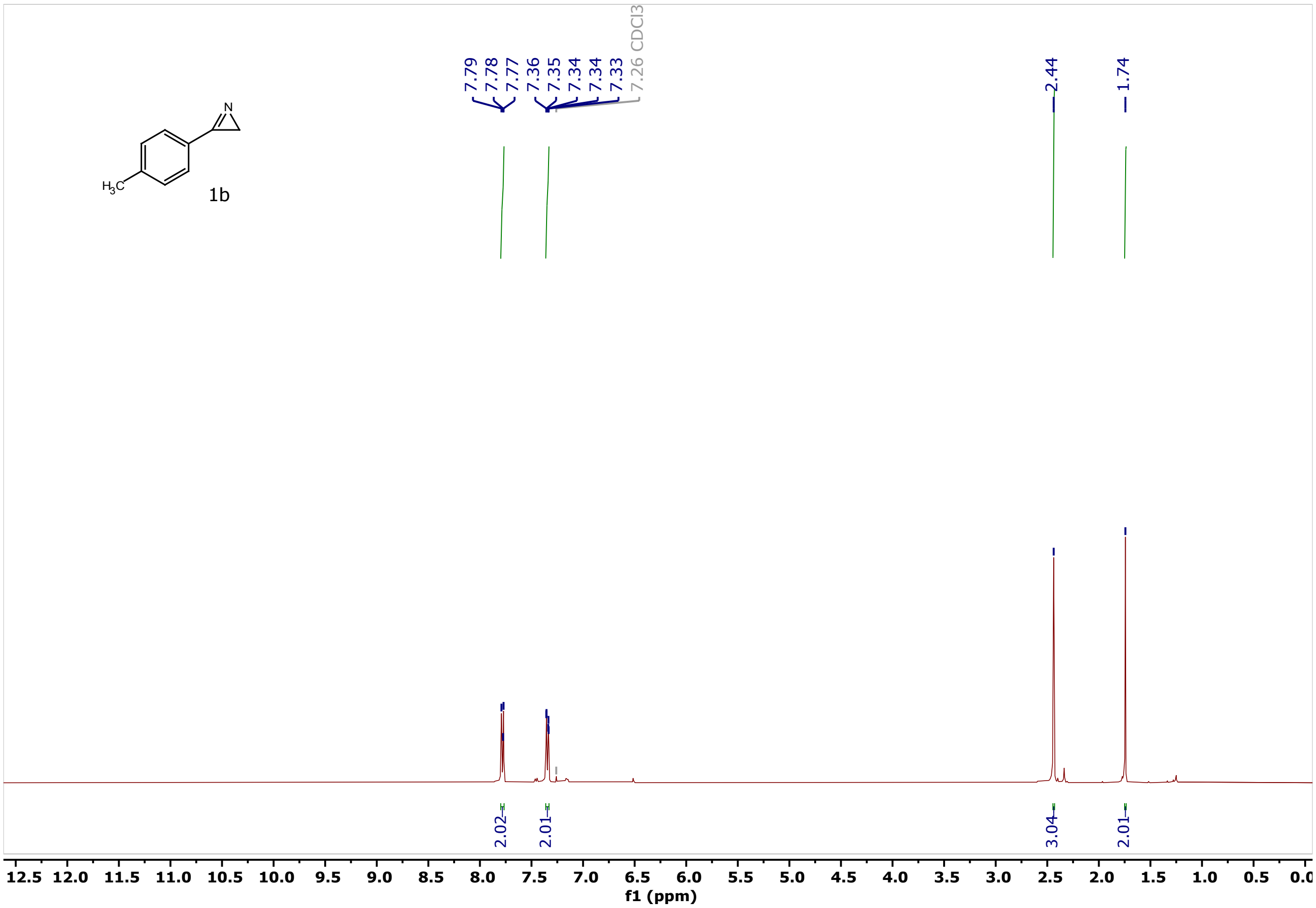

Figure 11. ^{13}C NMR spectrum of **1b** ($CDCl_3$, 101 MHz)

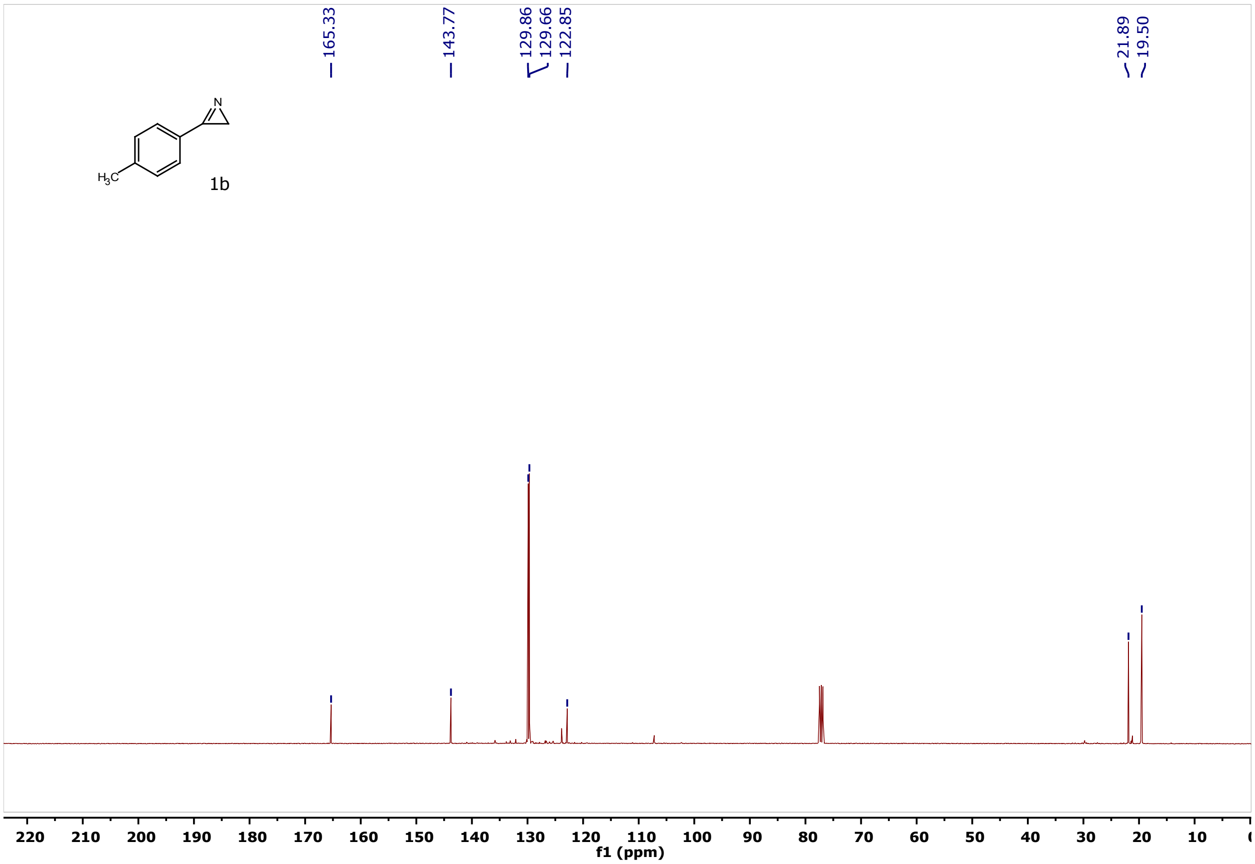

Figure 12. ^{1}H NMR spectrum of **1c** ($CDCl_3$, 400 MHz)

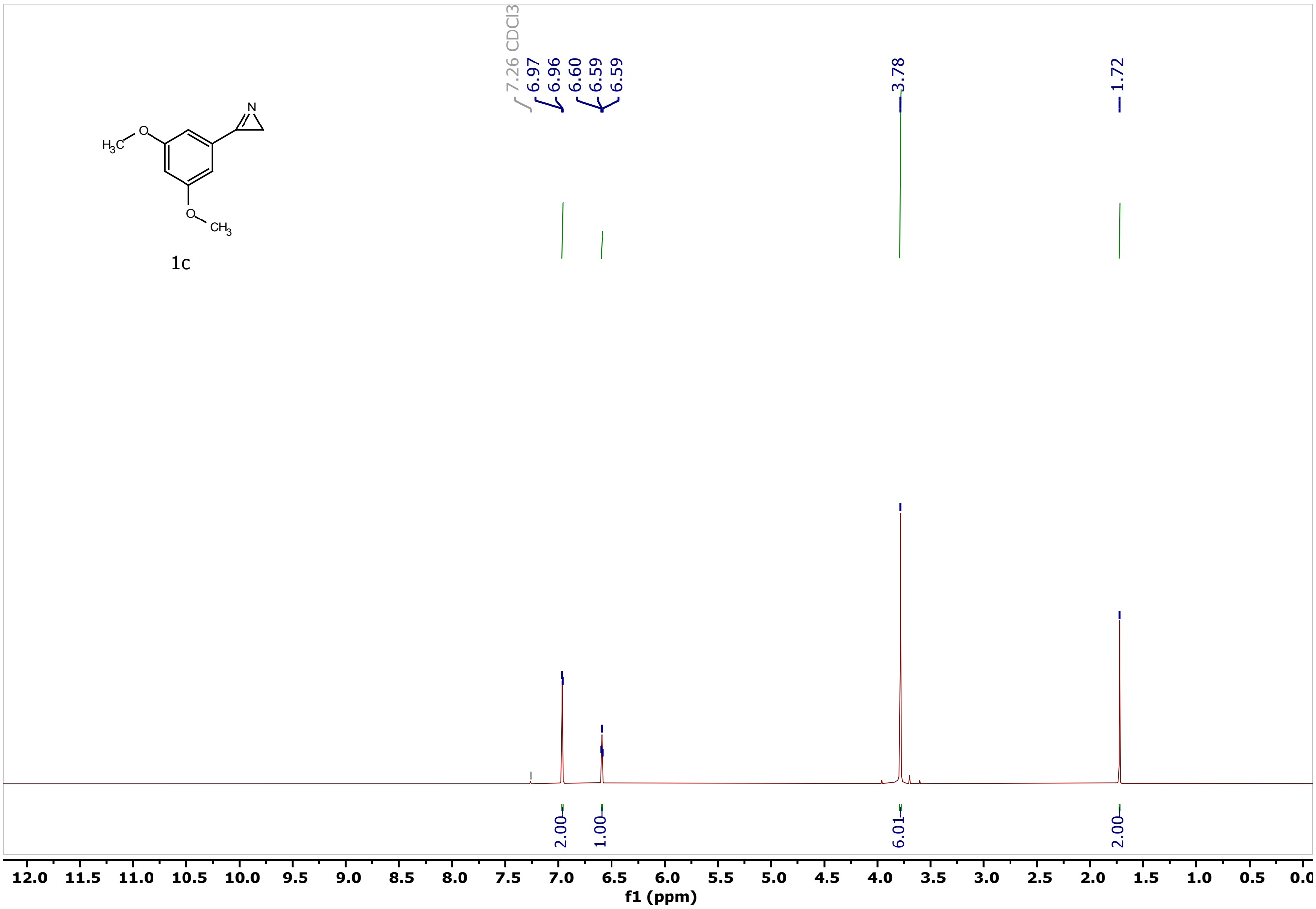

Figure 13. ^{13}C NMR spectrum of **1c** ($CDCl_3$, 101 MHz)

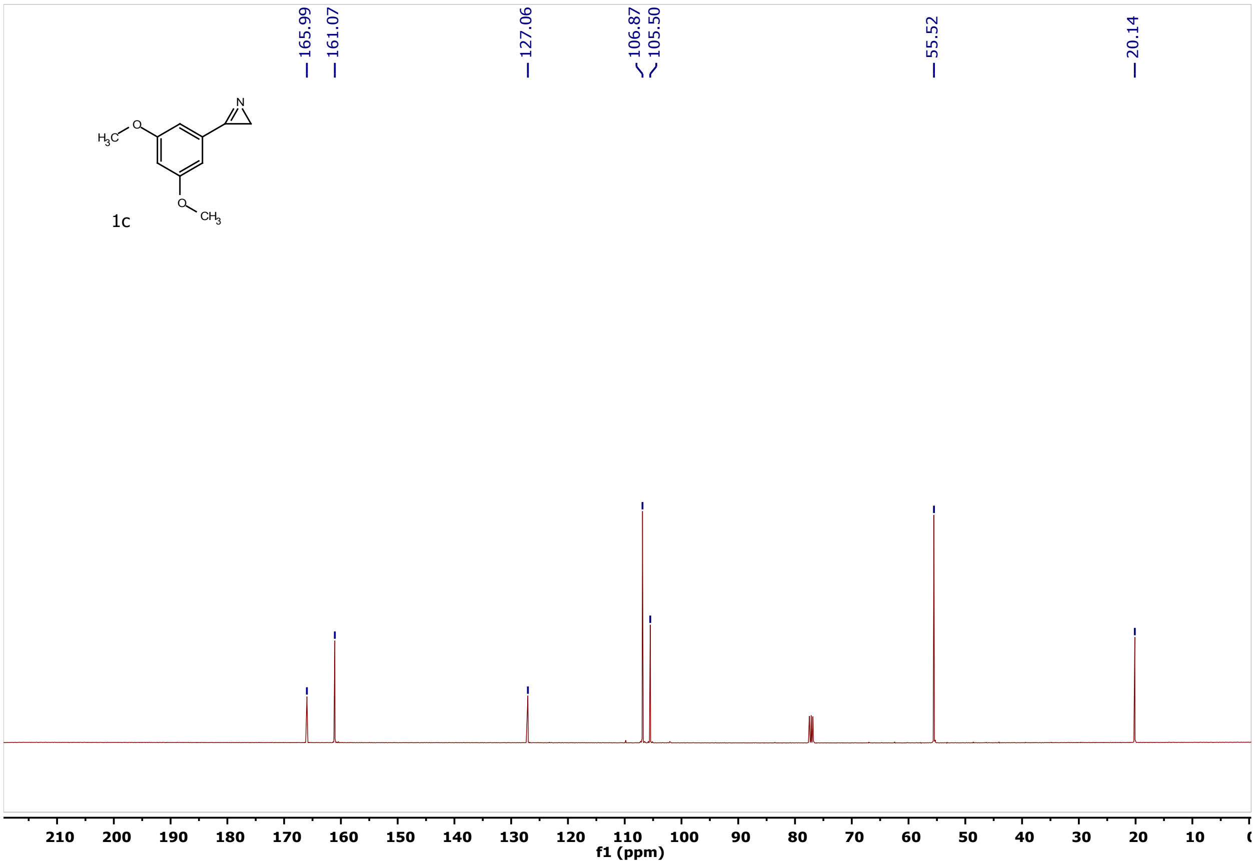

Figure 14. ^{1}H NMR spectrum of **1d** ($CDCl_3$, 400 MHz)

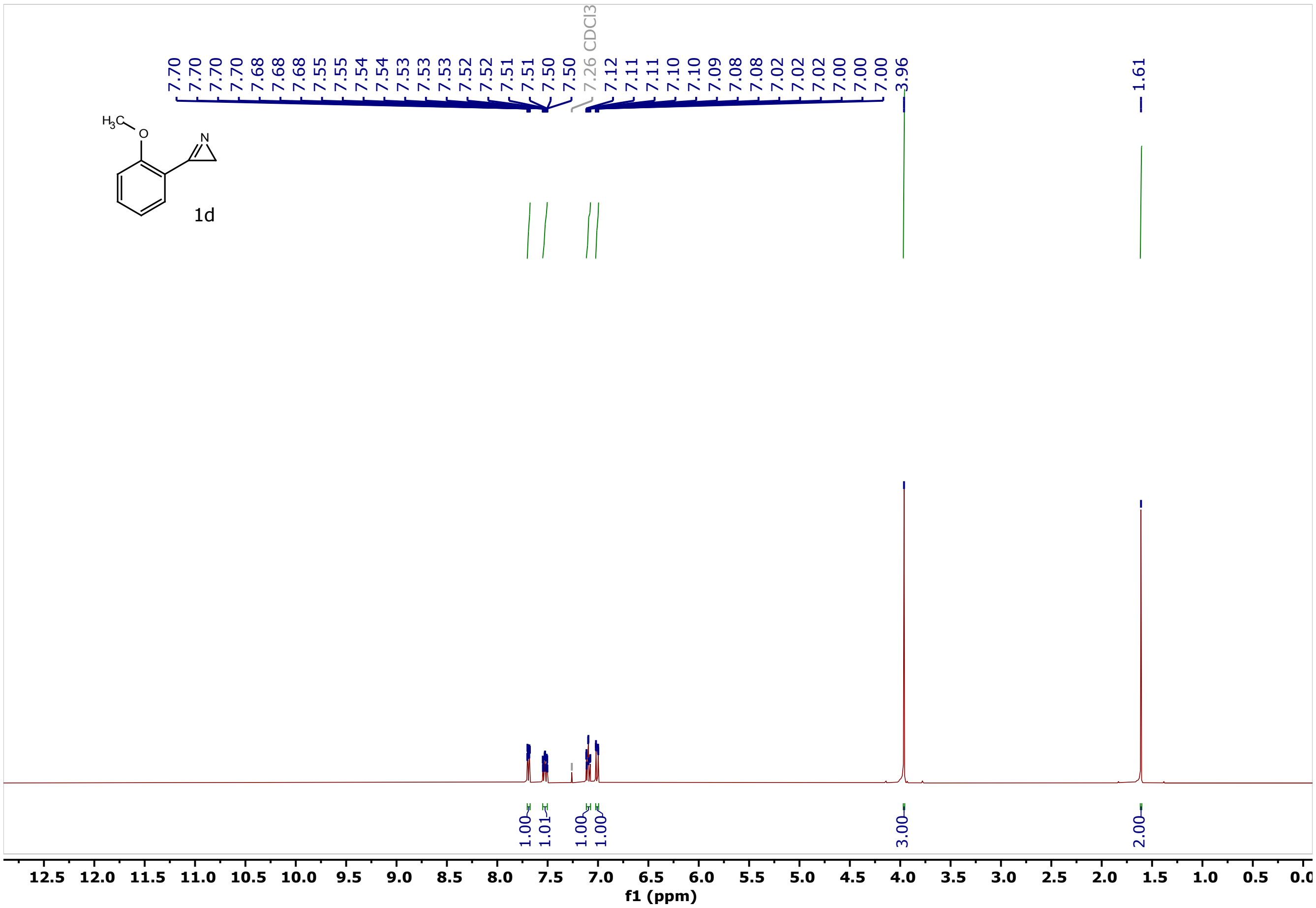

Figure 15. ^{13}C NMR spectrum of **1d** ($CDCl_3$, 101 MHz)

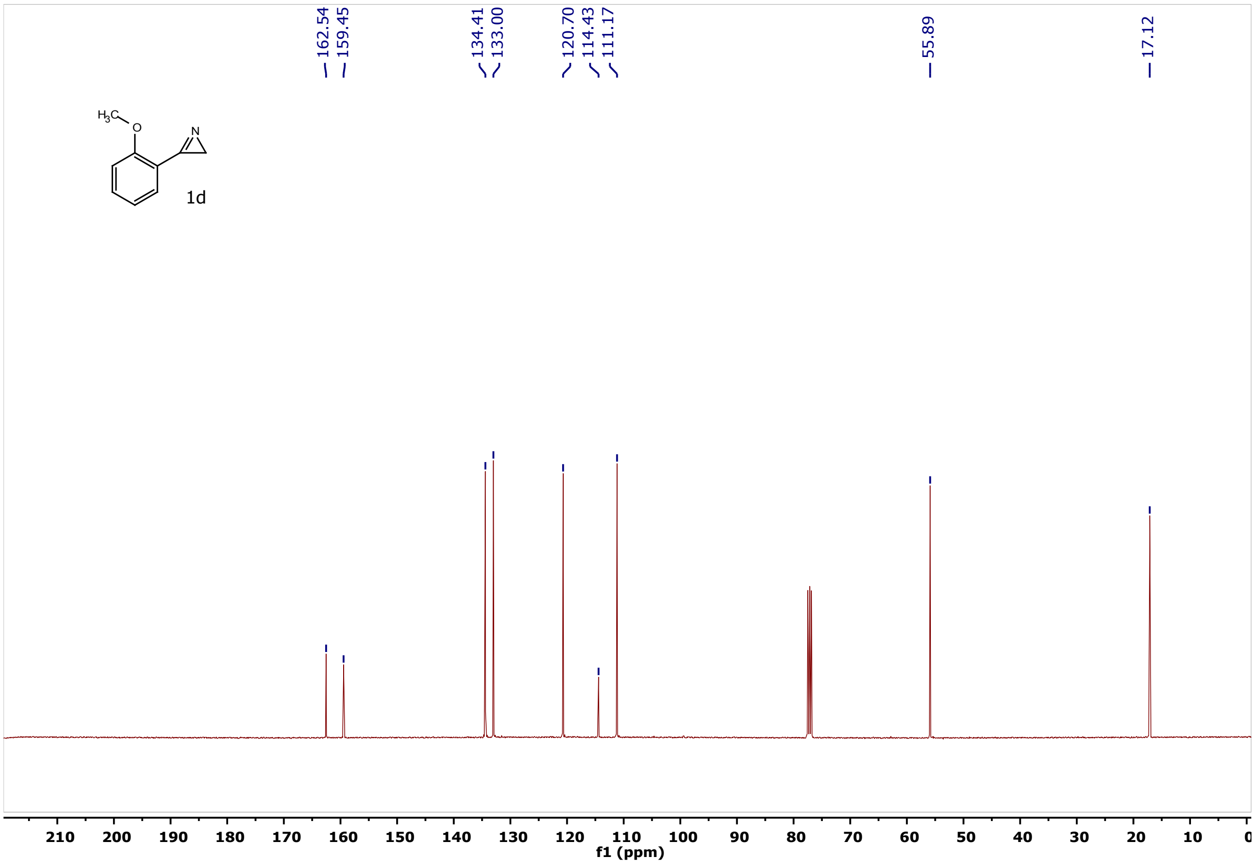

Figure 16. ^{1}H NMR spectrum of **1e** ($CDCl_3$, 400 MHz)

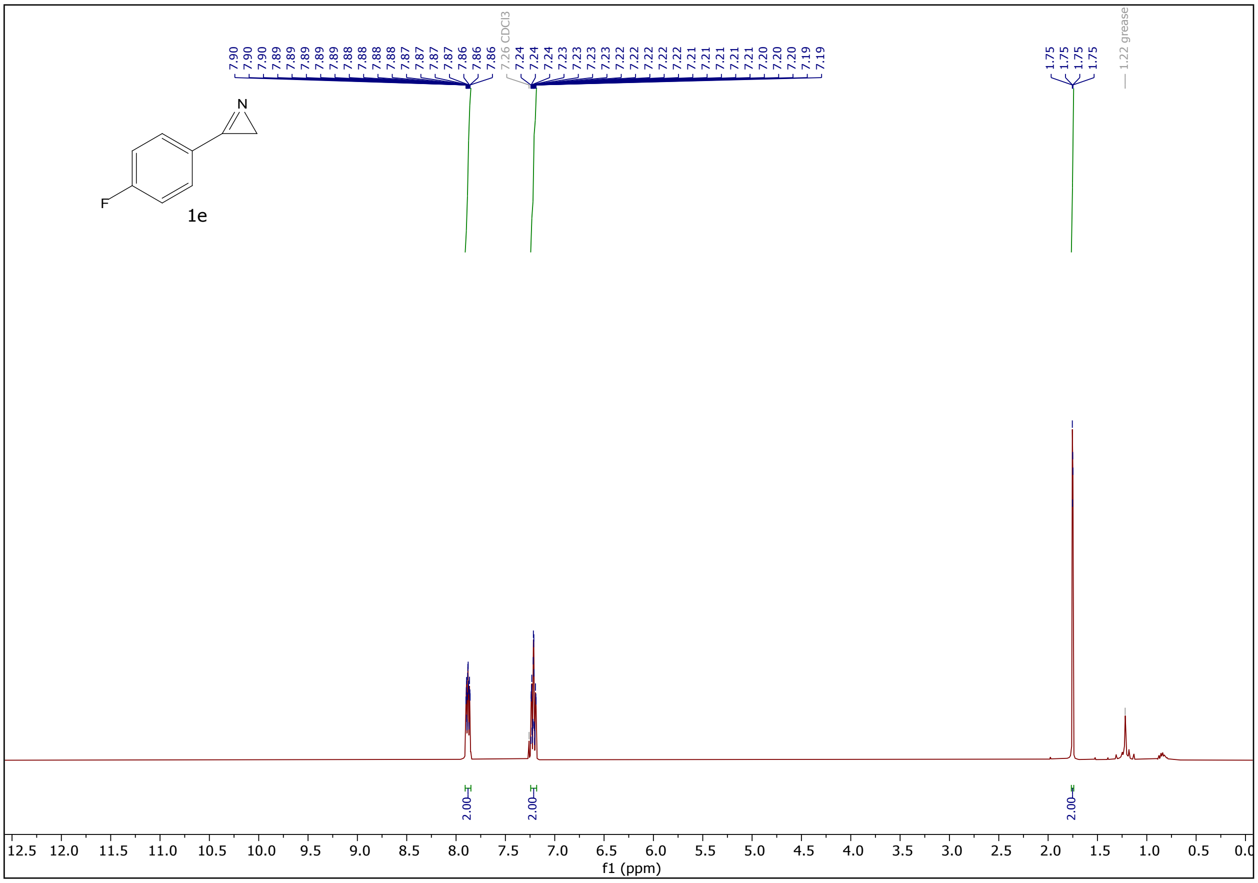

Figure 17. ^{13}C NMR spectrum of **1e** ($CDCl_3$, 101 MHz)

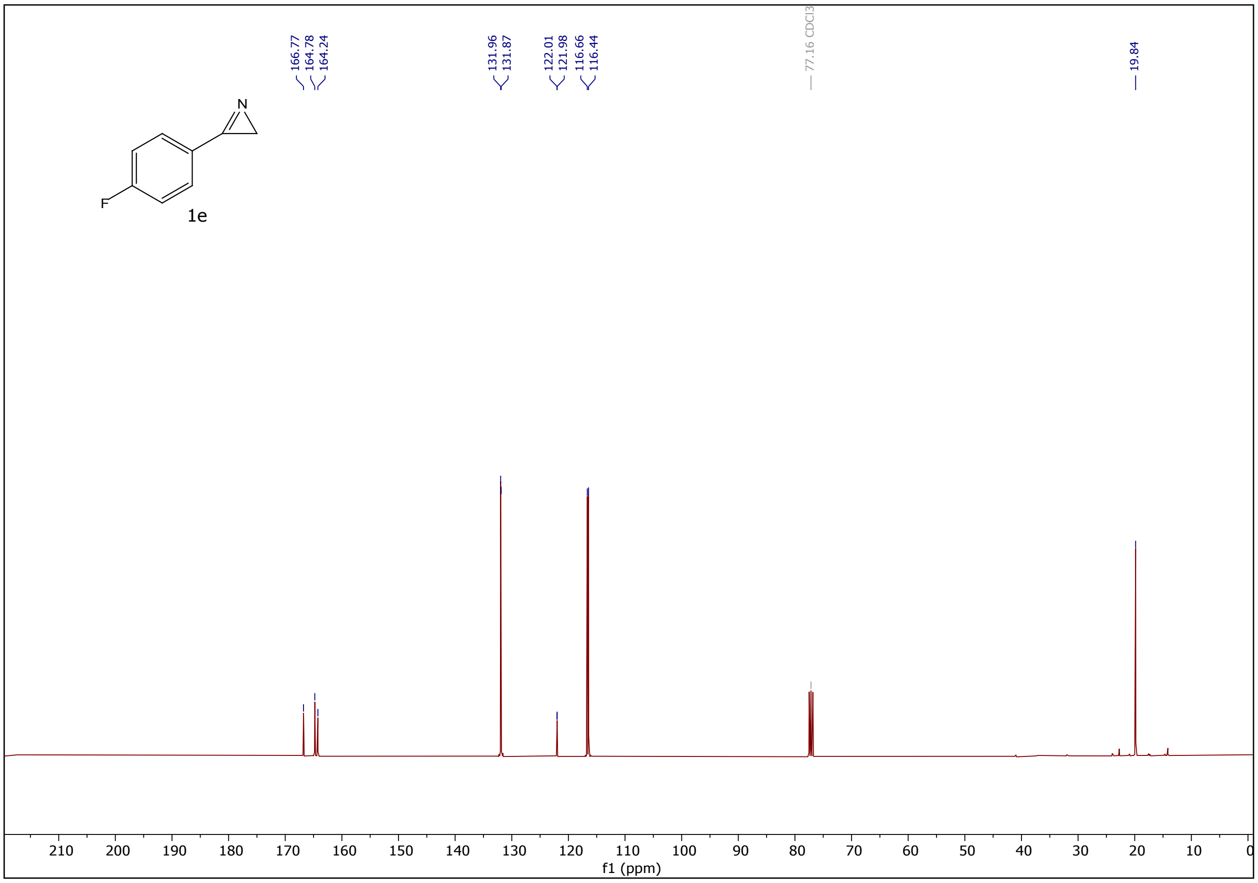

Figure 18. ^{19}F NMR spectrum of **1e** ($CDCl_3$, 377 MHz)

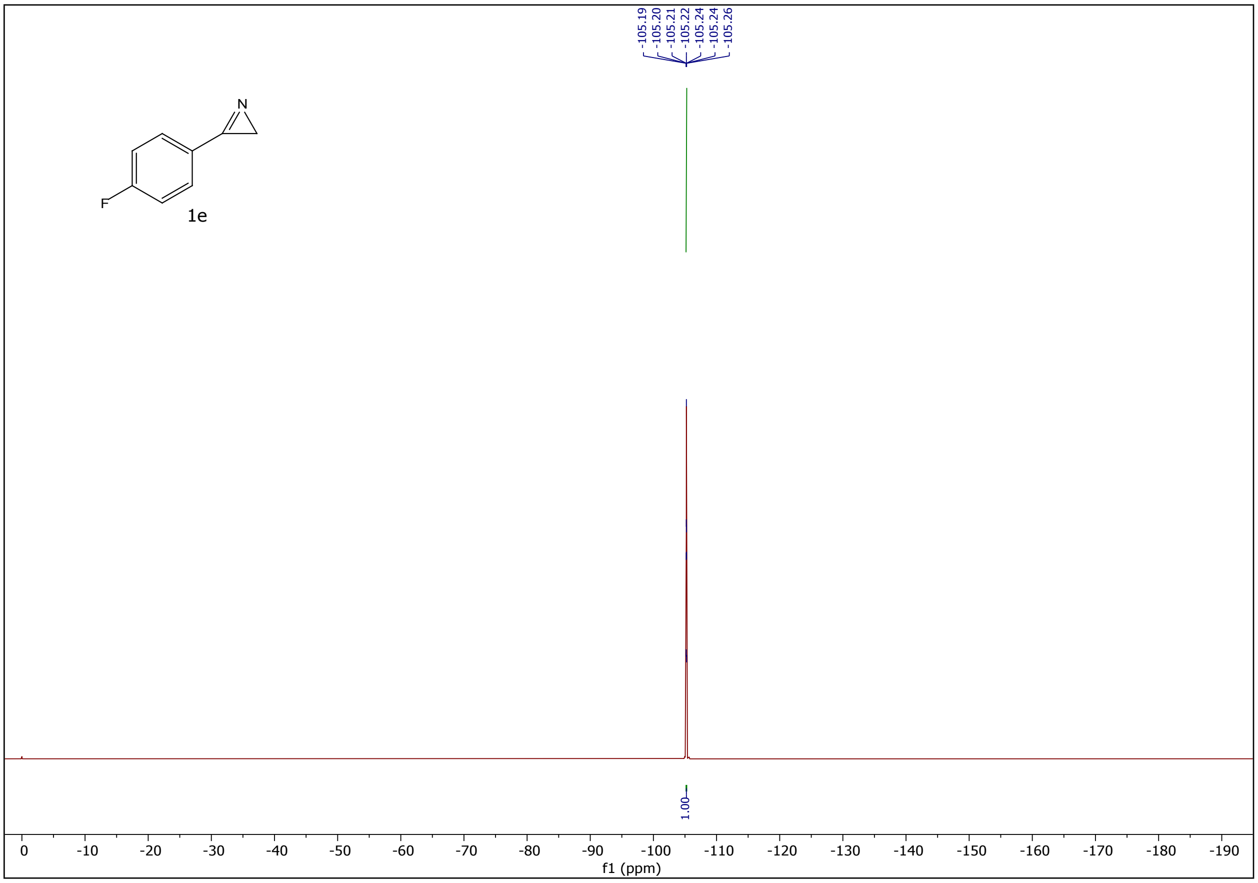

Figure 19. ^{1}H NMR spectrum of **2a** ($CDCl_3$, 400 MHz)

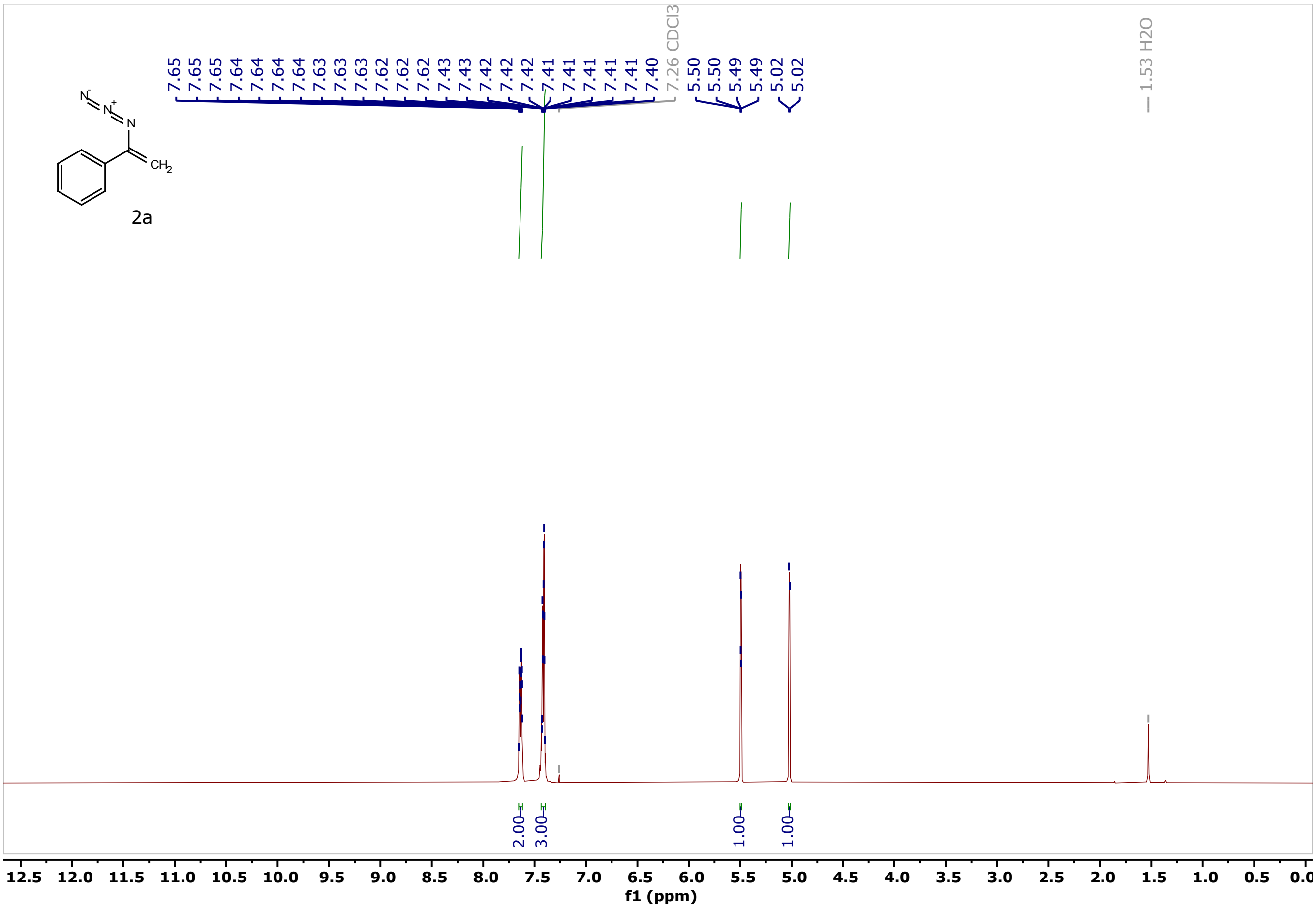

Figure 20. ^{13}C NMR spectrum of **2a** ($CDCl_3$, 101 MHz)

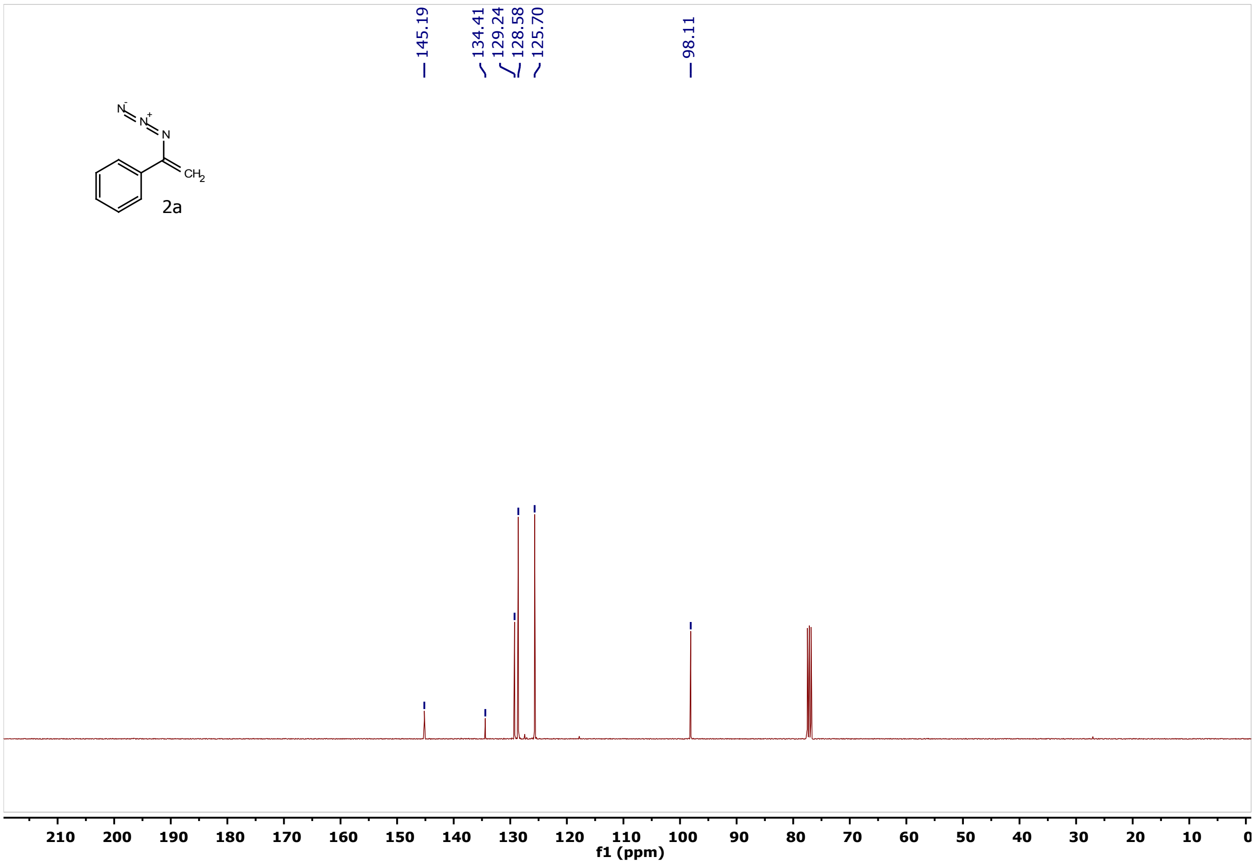

Figure 21. ^{1}H NMR spectrum of **2b** ($CDCl_3$, 400 MHz)

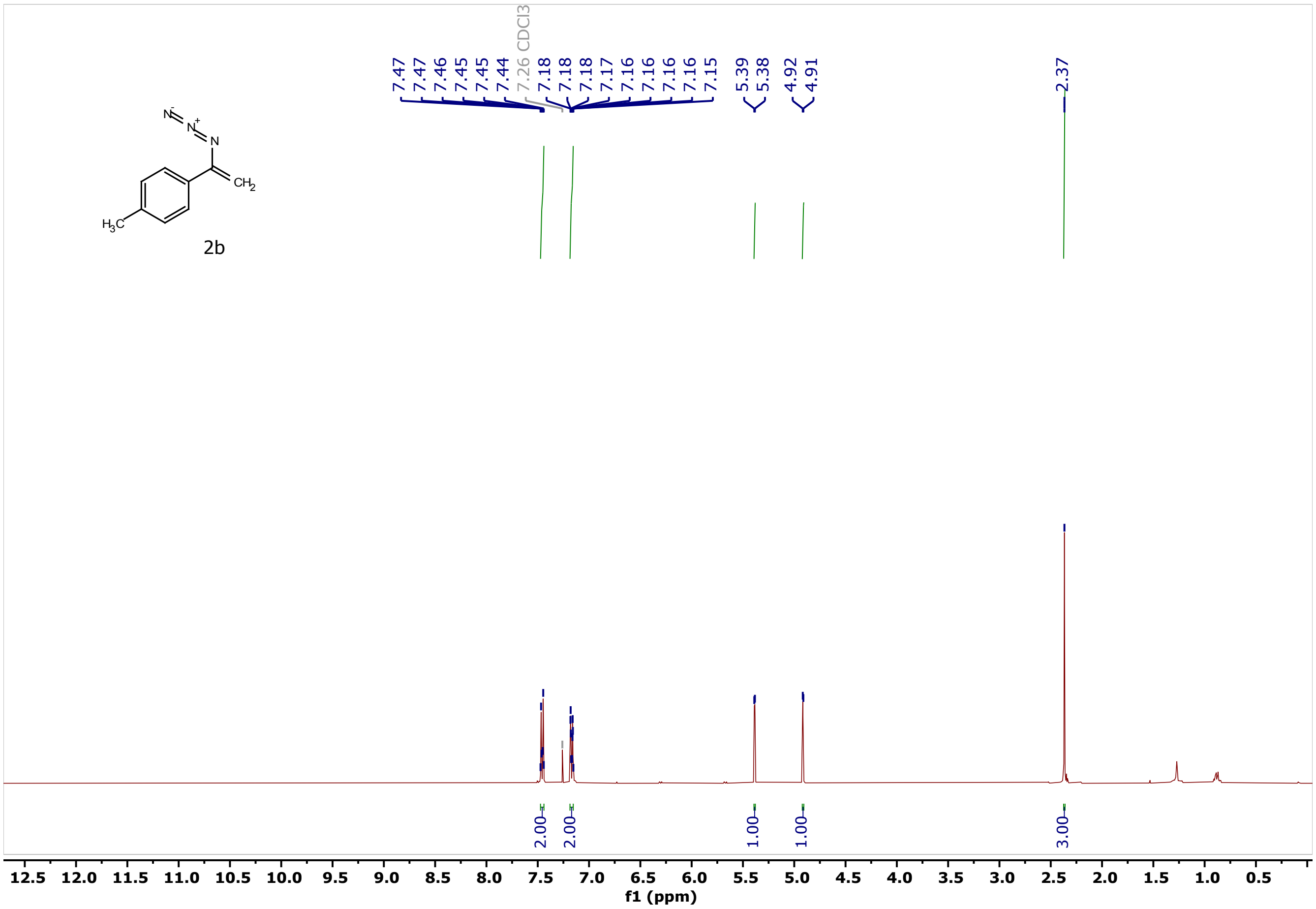

Figure 22. ^{13}C NMR spectrum of **2b** ($CDCl_3$, 101 MHz)

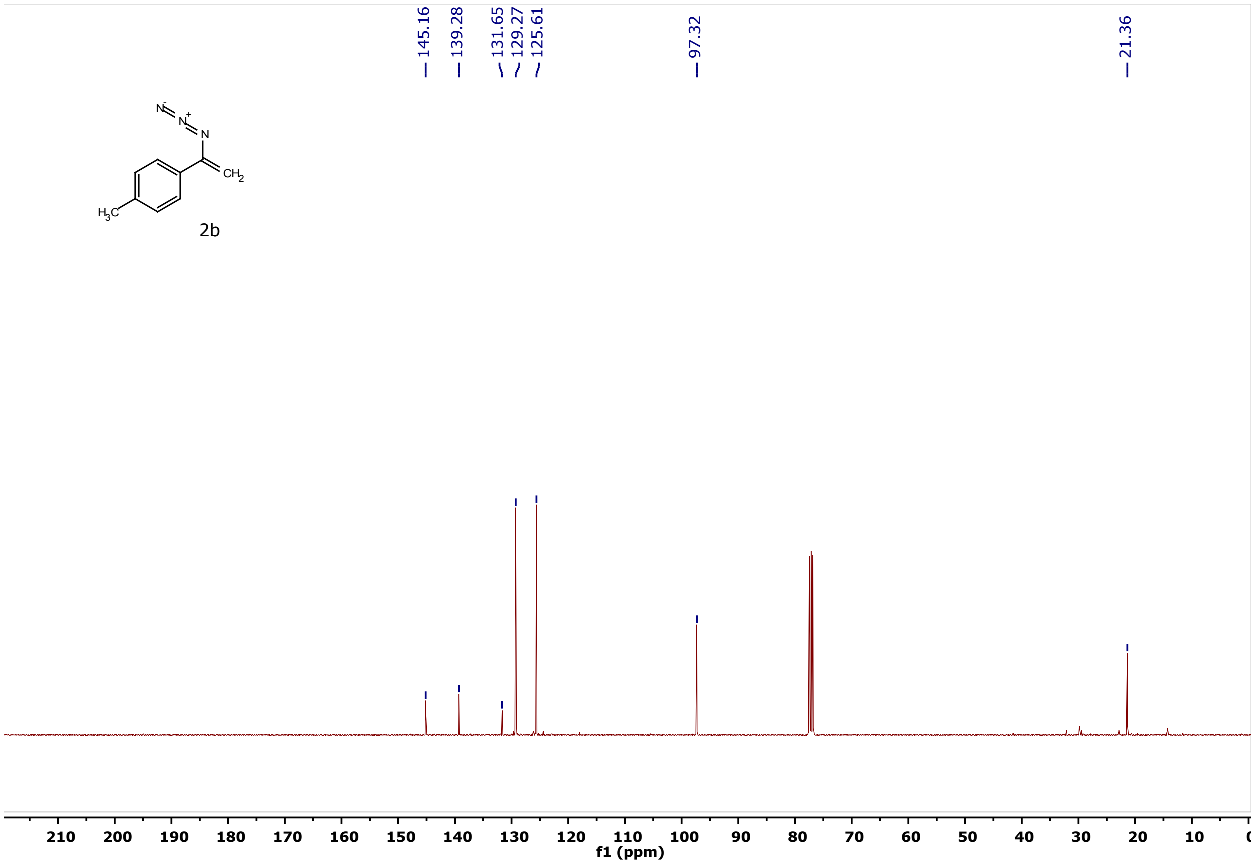

Figure 23. ^{1}H NMR spectrum of **2c** ($CDCl_3$, 400 MHz)

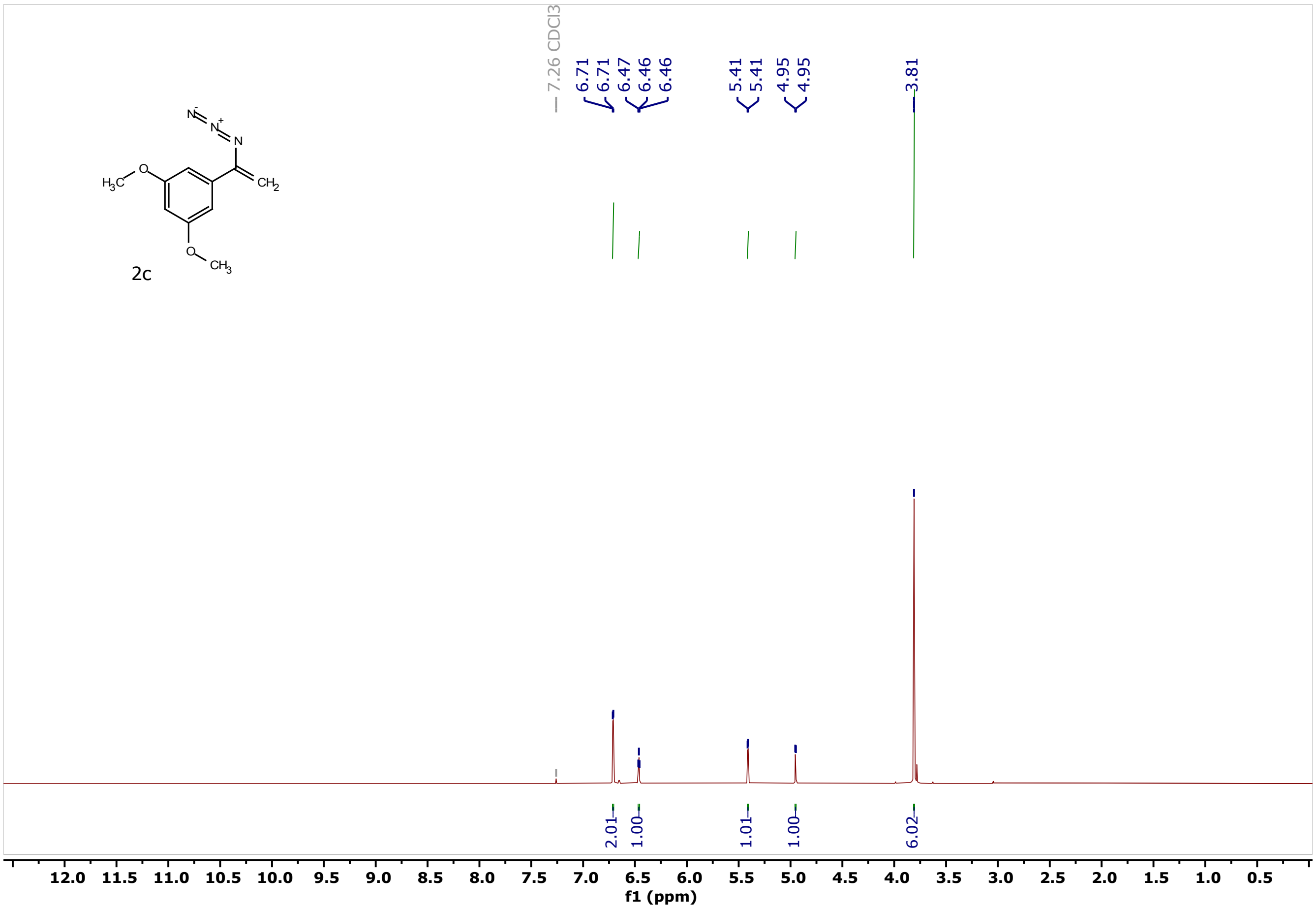

Figure 24. ^{13}C NMR spectrum of **2c** ($CDCl_3$, 101 MHz)

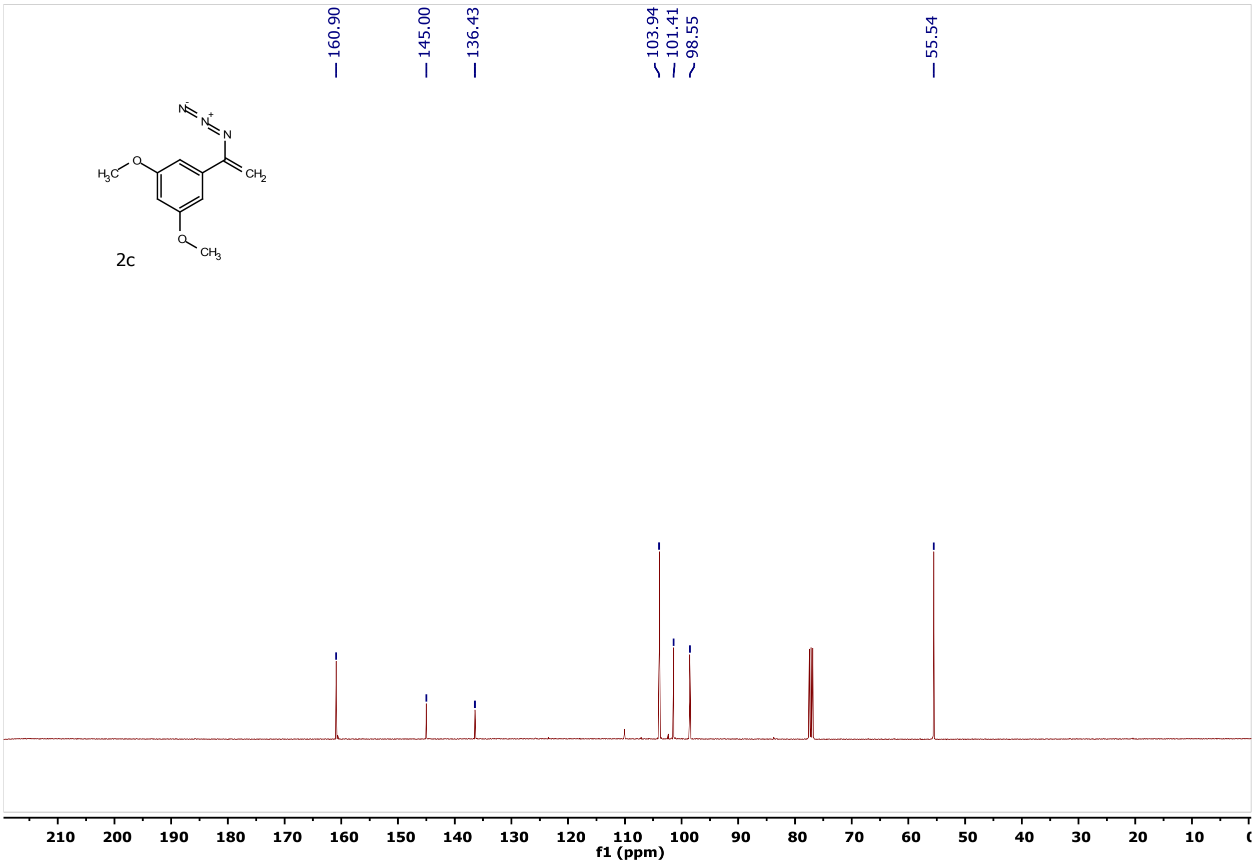

Figure 25. ^{1}H NMR spectrum of **2d** ($CDCl_3$, 400 MHz)

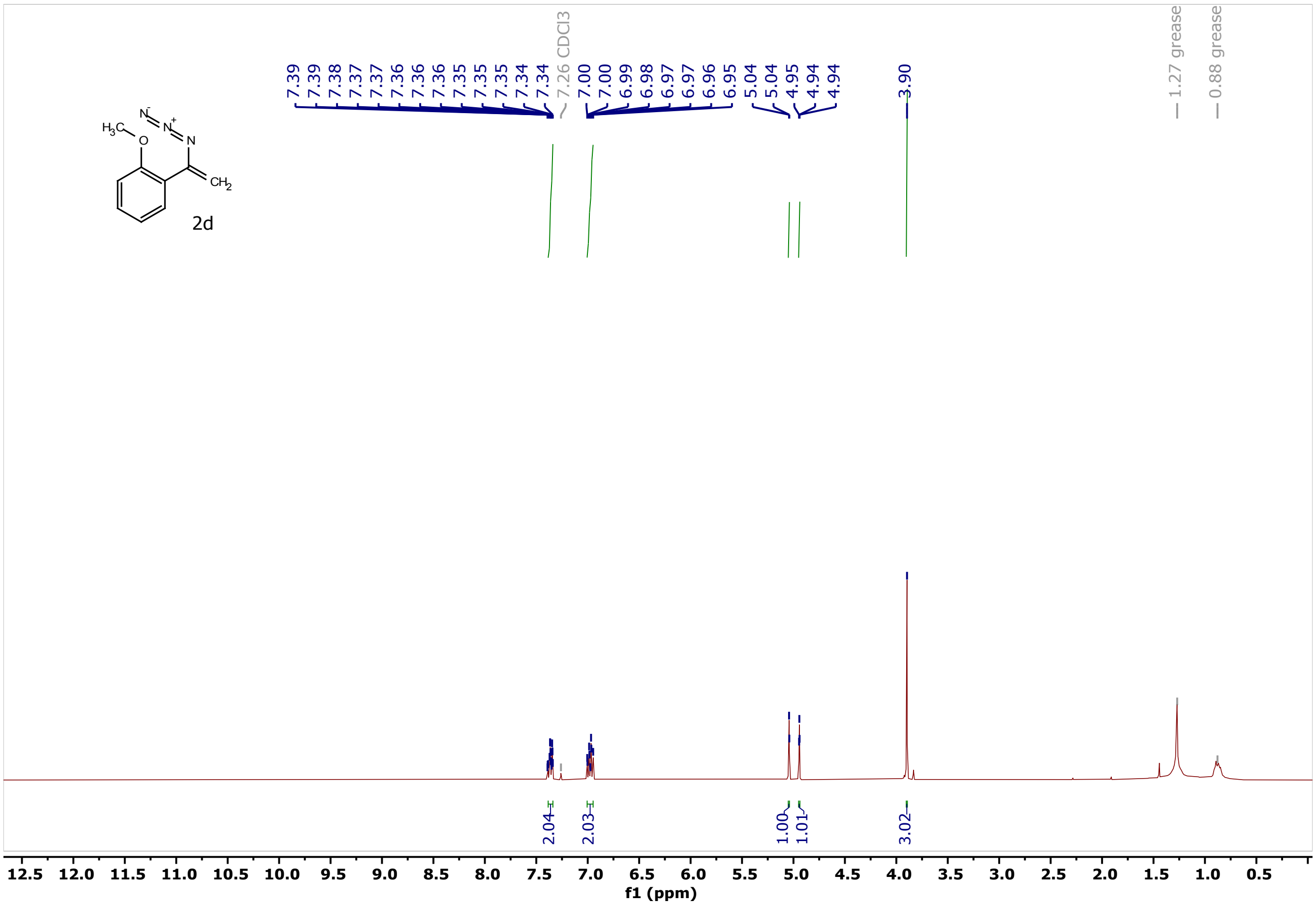

Figure 26. ^{13}C NMR spectrum of **2d** ($CDCl_3$, 101 MHz)

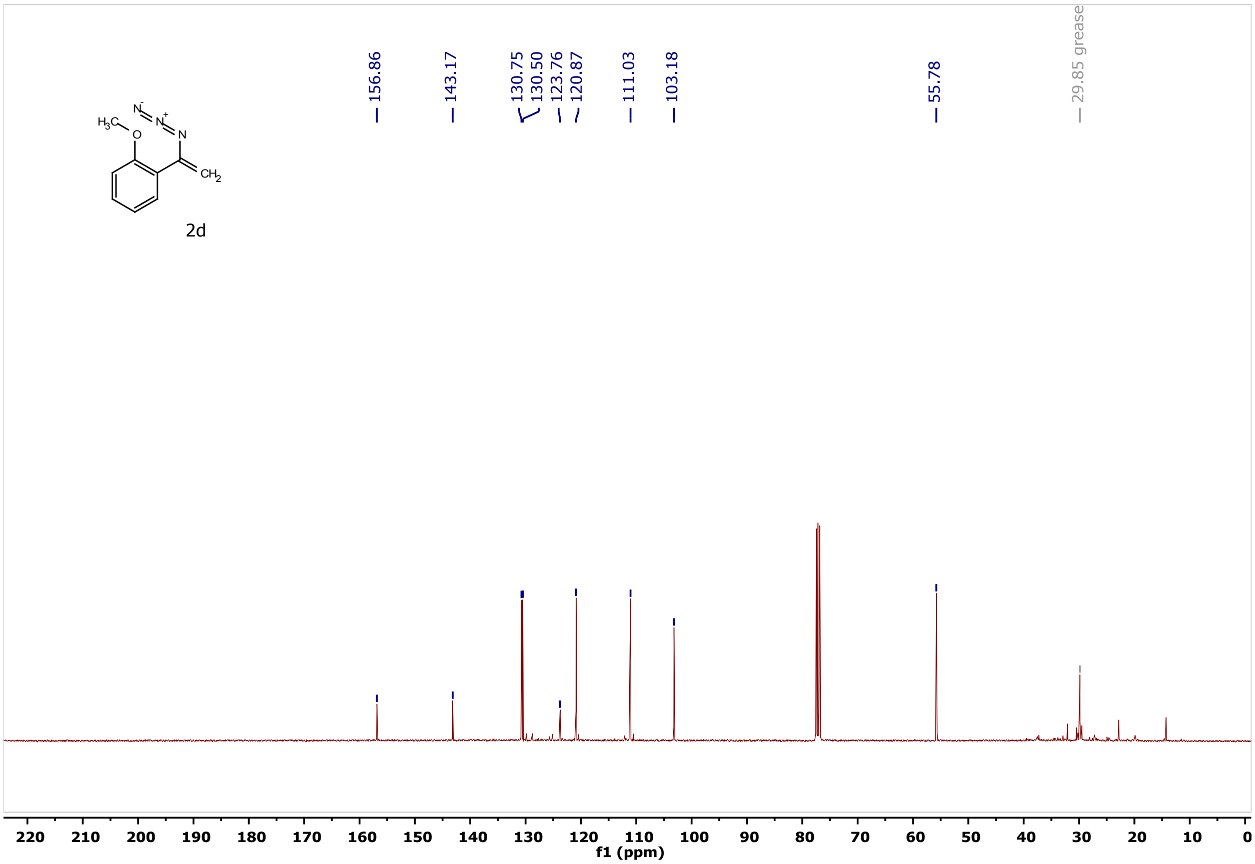

Figure 27. ^{1}H NMR spectrum of **2e** ($CDCl_3$, 400 MHz)

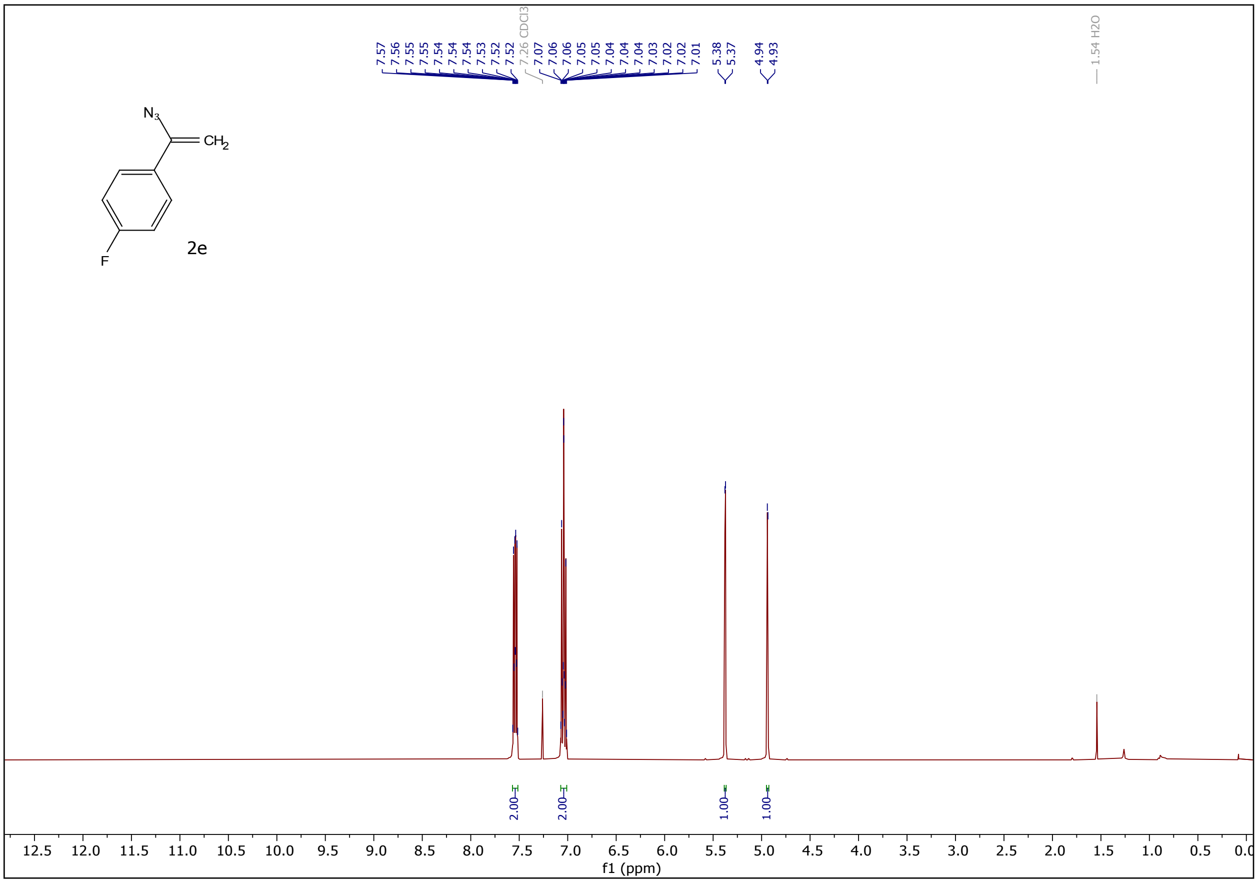

Figure 28. ^{13}C NMR spectrum of **2e** ($CDCl_3$, 101 MHz)

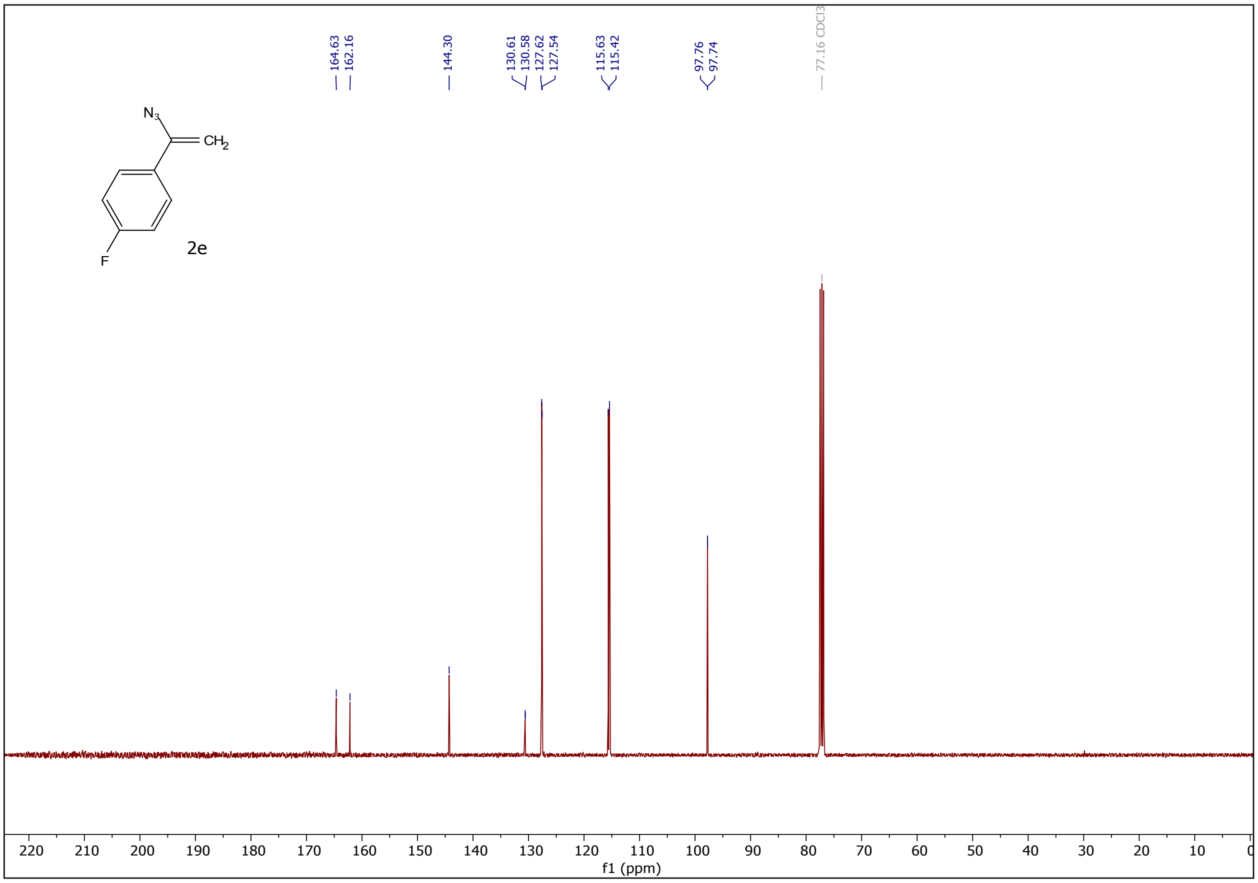

Figure 29. ^{19}F NMR spectrum of **2e** ($CDCl_3$, 377 MHz)

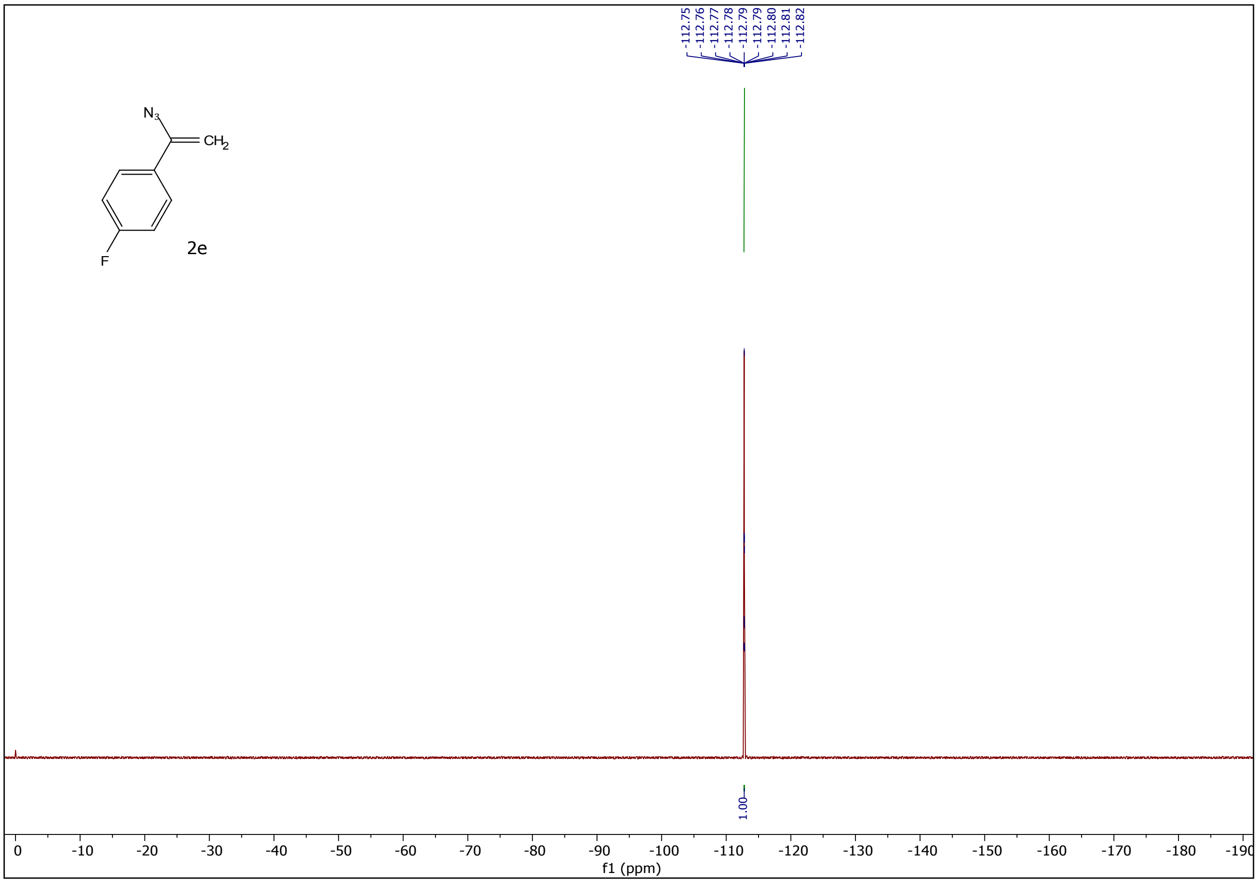

Figure 30. ^{1}H NMR spectrum of **3a** ($CDCl_3$, 400 MHz)

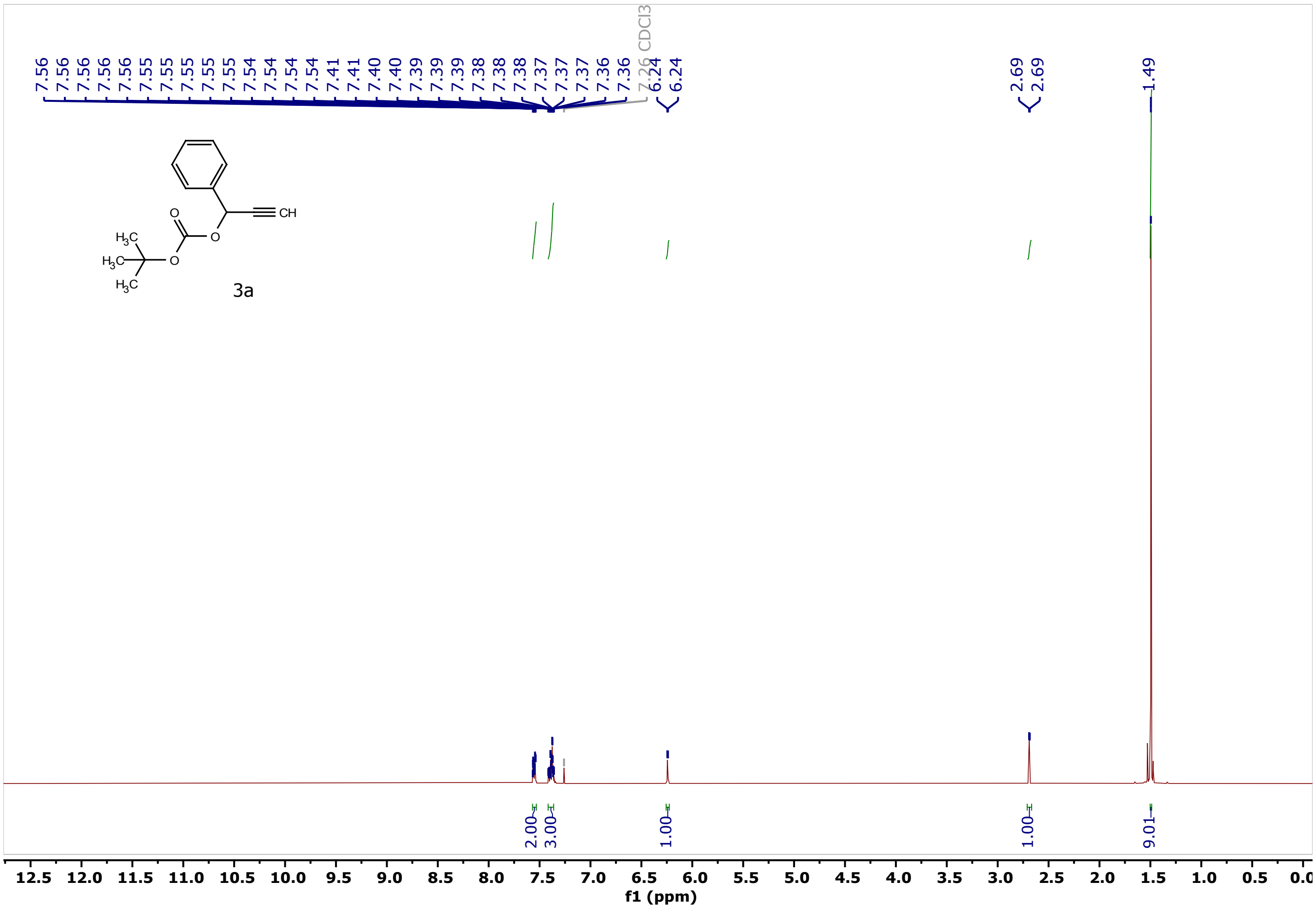

Figure 31. ^{13}C NMR spectrum of **3a** ($CDCl_3$, 101 MHz)

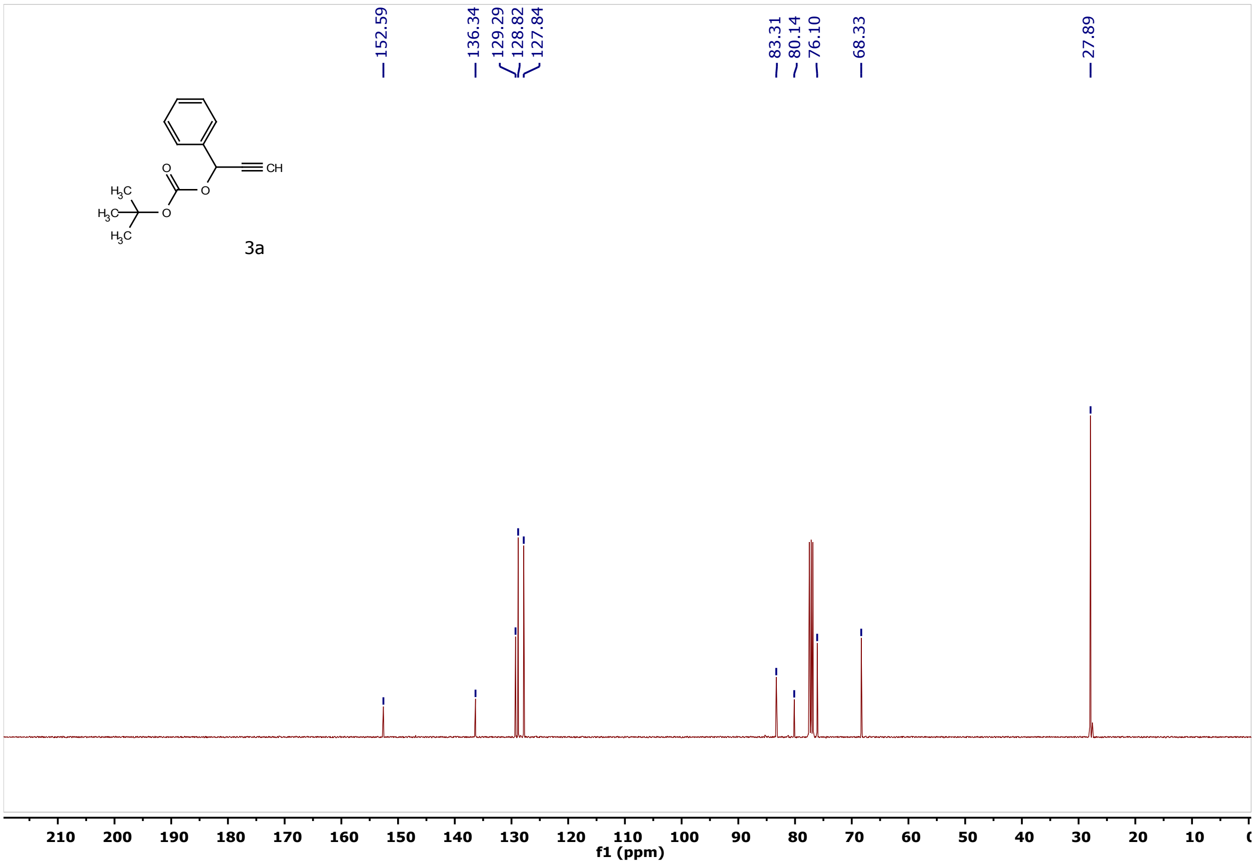

Figure 32. ^{1}H NMR spectrum of **4a** ($CDCl_3$, 400 MHz)

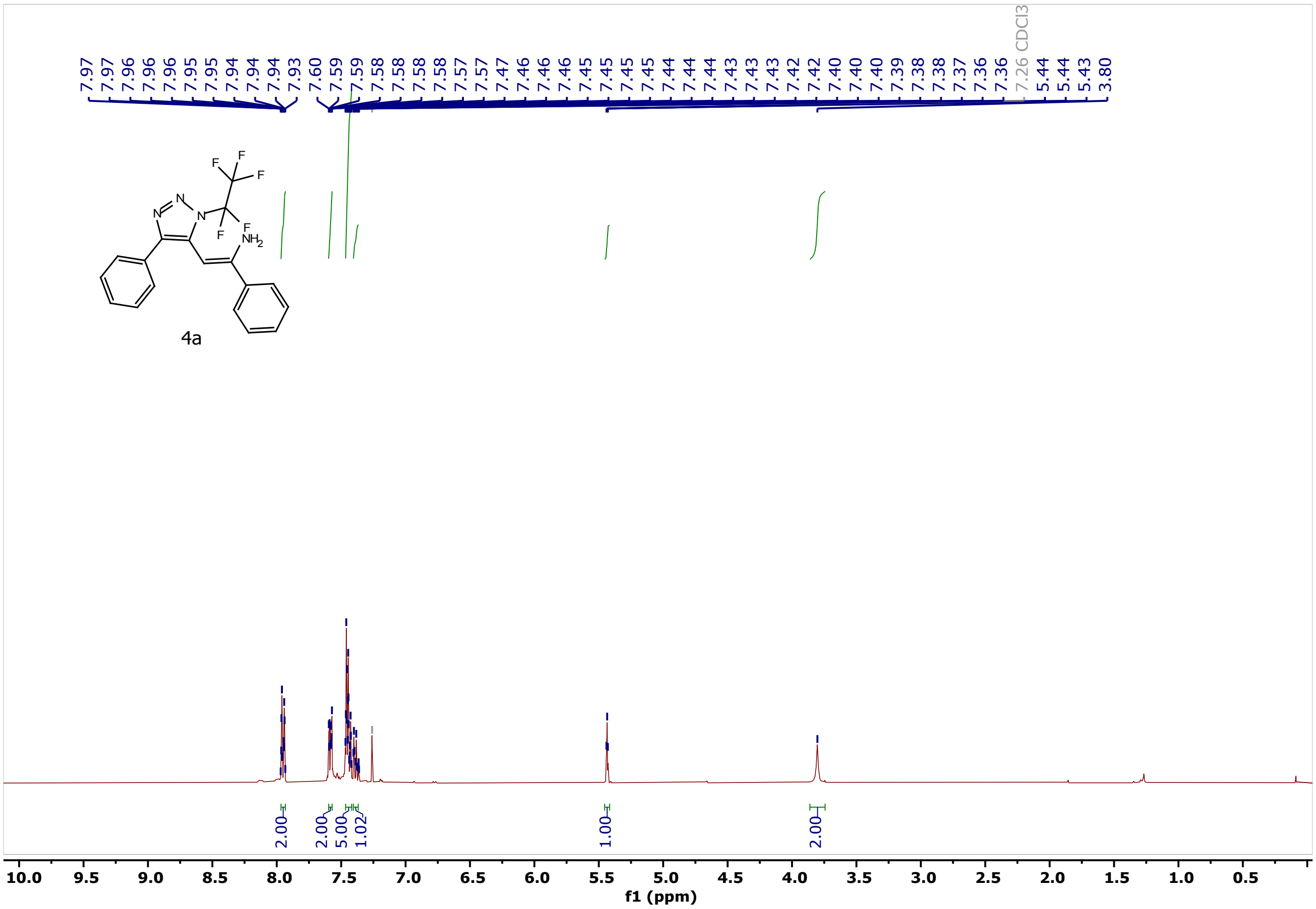

Figure 33. ^{13}C NMR spectrum of **4a** ($CDCl_3$, 101 MHz)

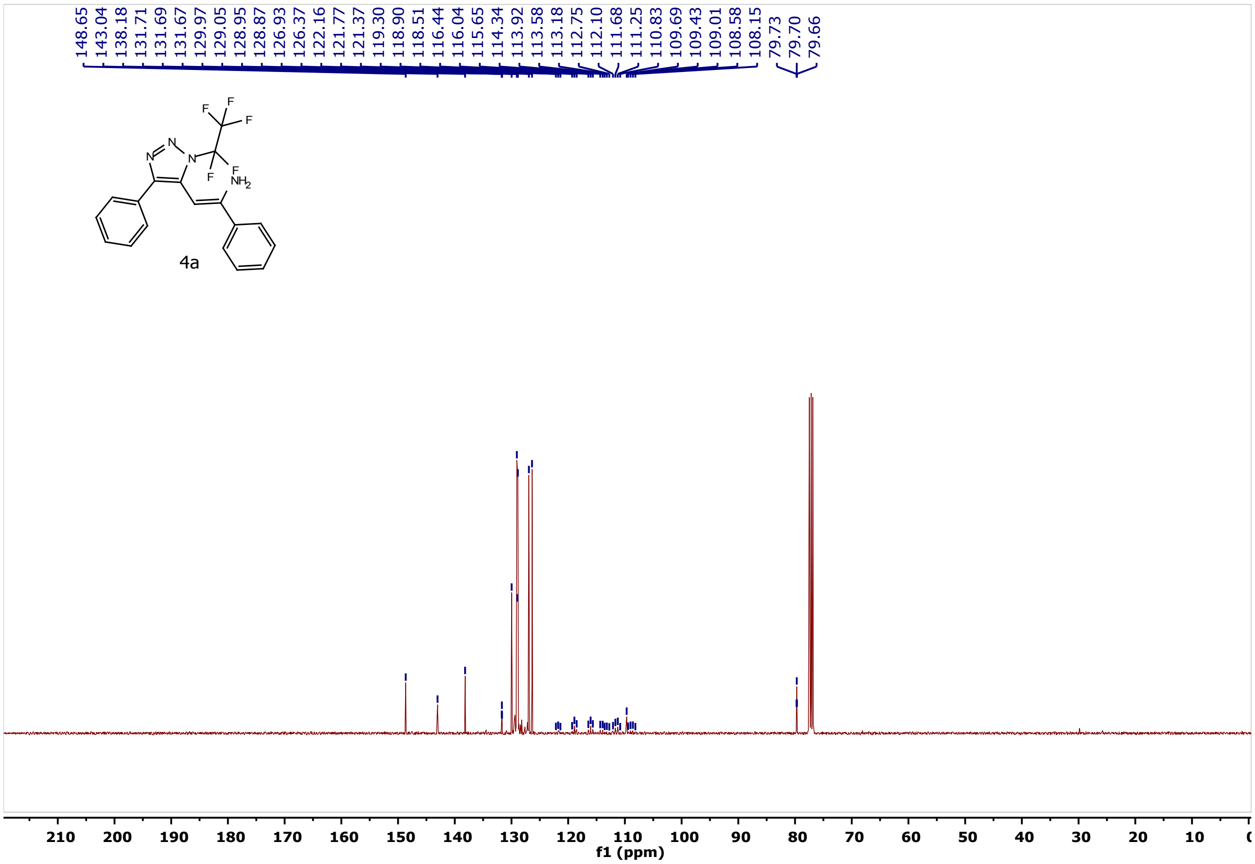

Figure 34. ^{19}F NMR spectrum of **4a** ($CDCl_3$, 377 MHz)

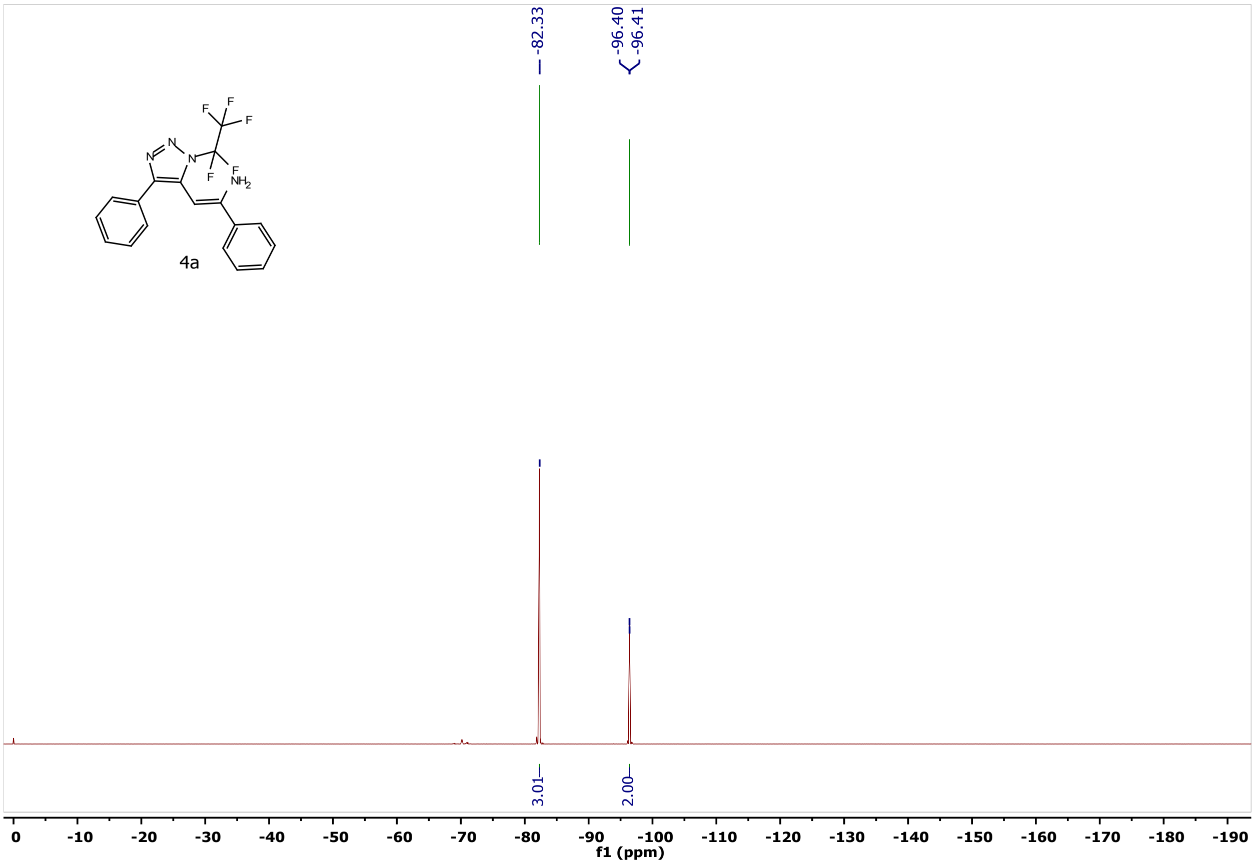

Figure 35. ^1H NMR spectrum of **4b** ($CDCl_3$, 400 MHz)

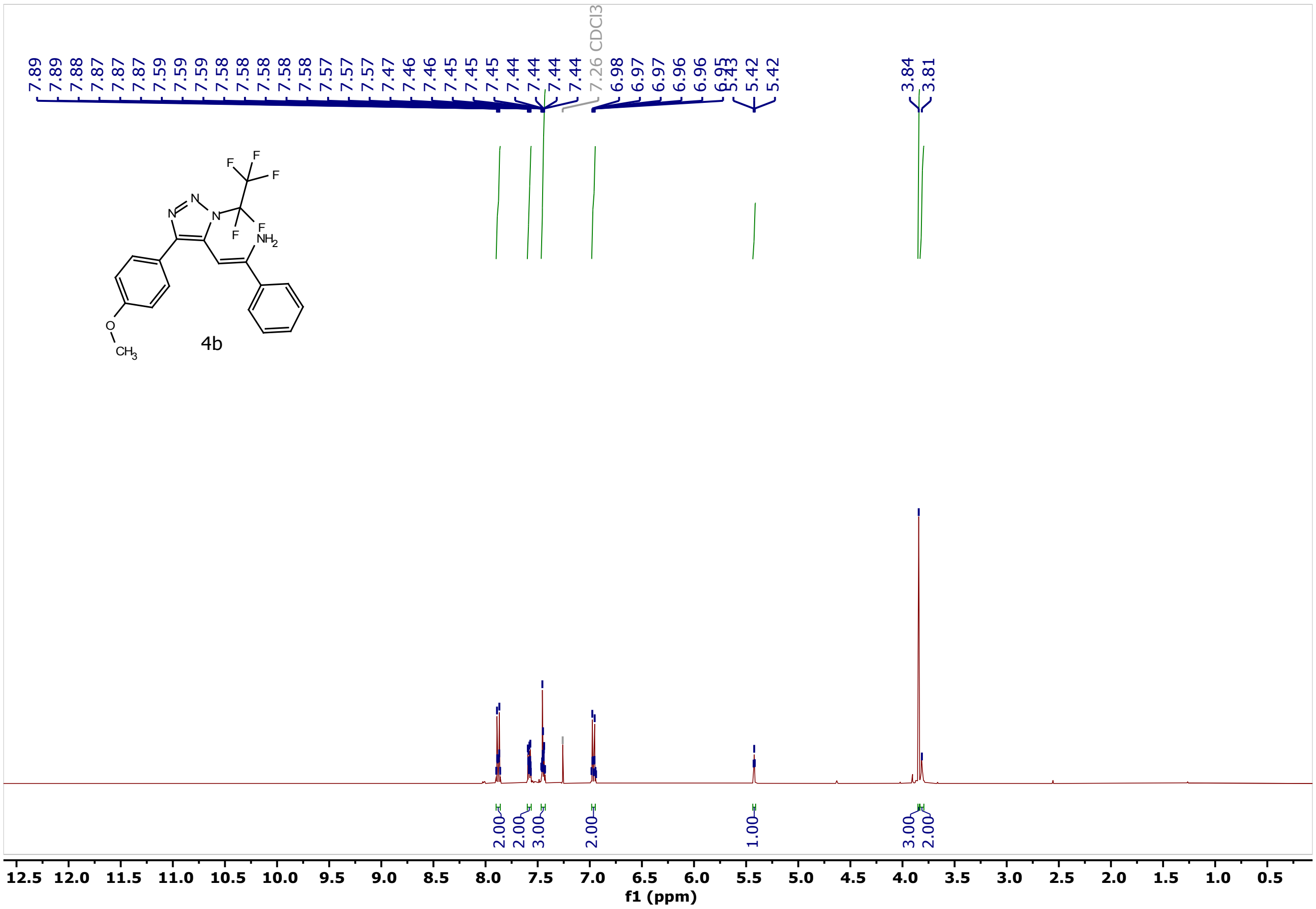

Figure 36. ^{13}C NMR spectrum of **4b** ($CDCl_3$, 101 MHz)

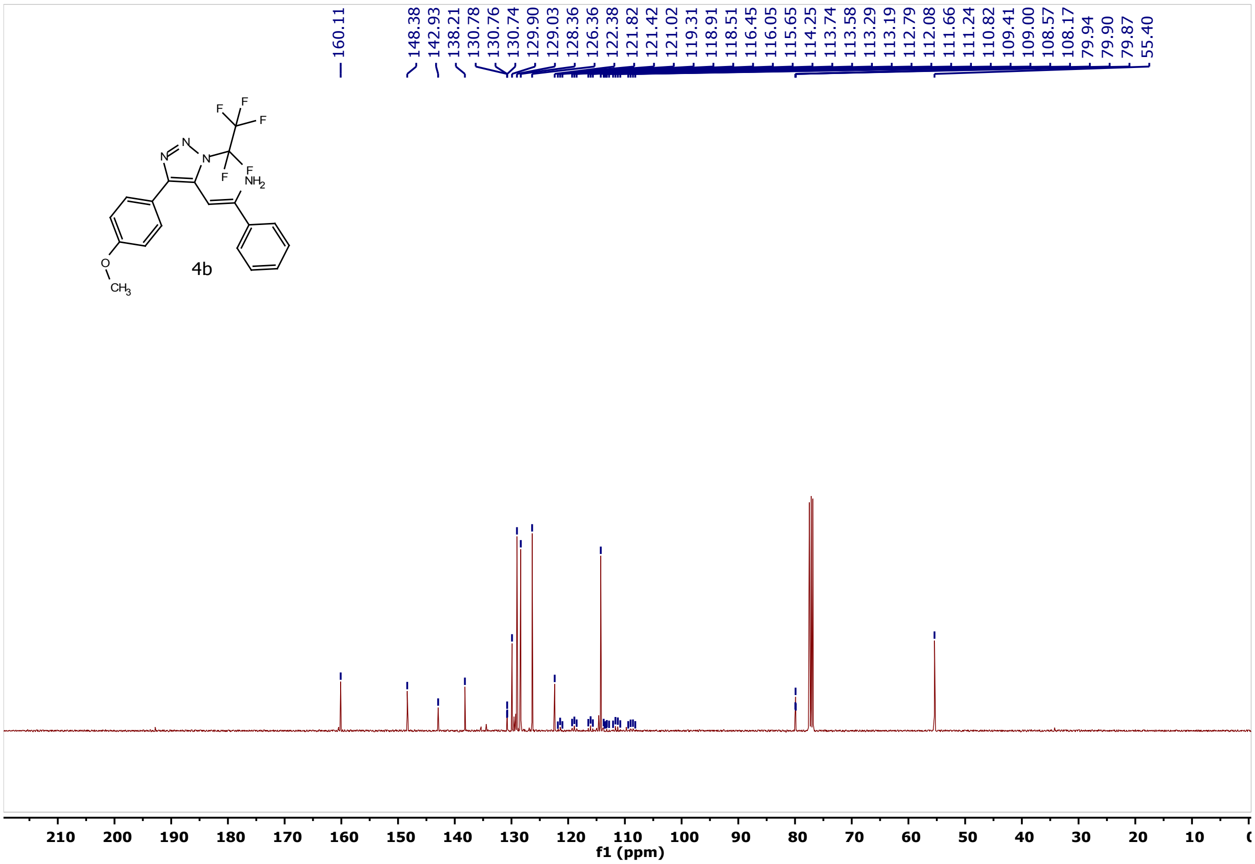

Figure 37. ^{19}F NMR spectrum of **4b** ($CDCl_3$, 377 MHz)

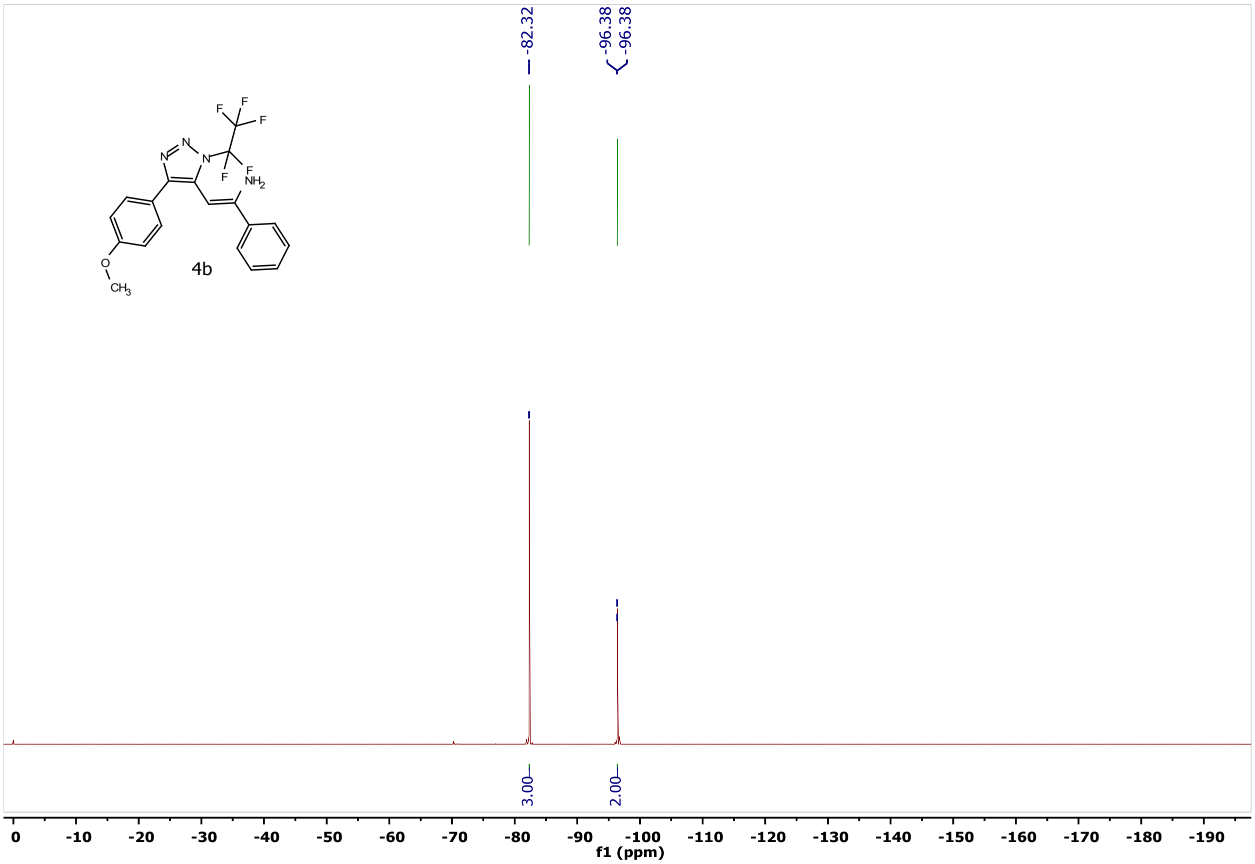

Figure 38. ^{1}H NMR spectrum of **4c** ($CDCl_3$, 400 MHz)

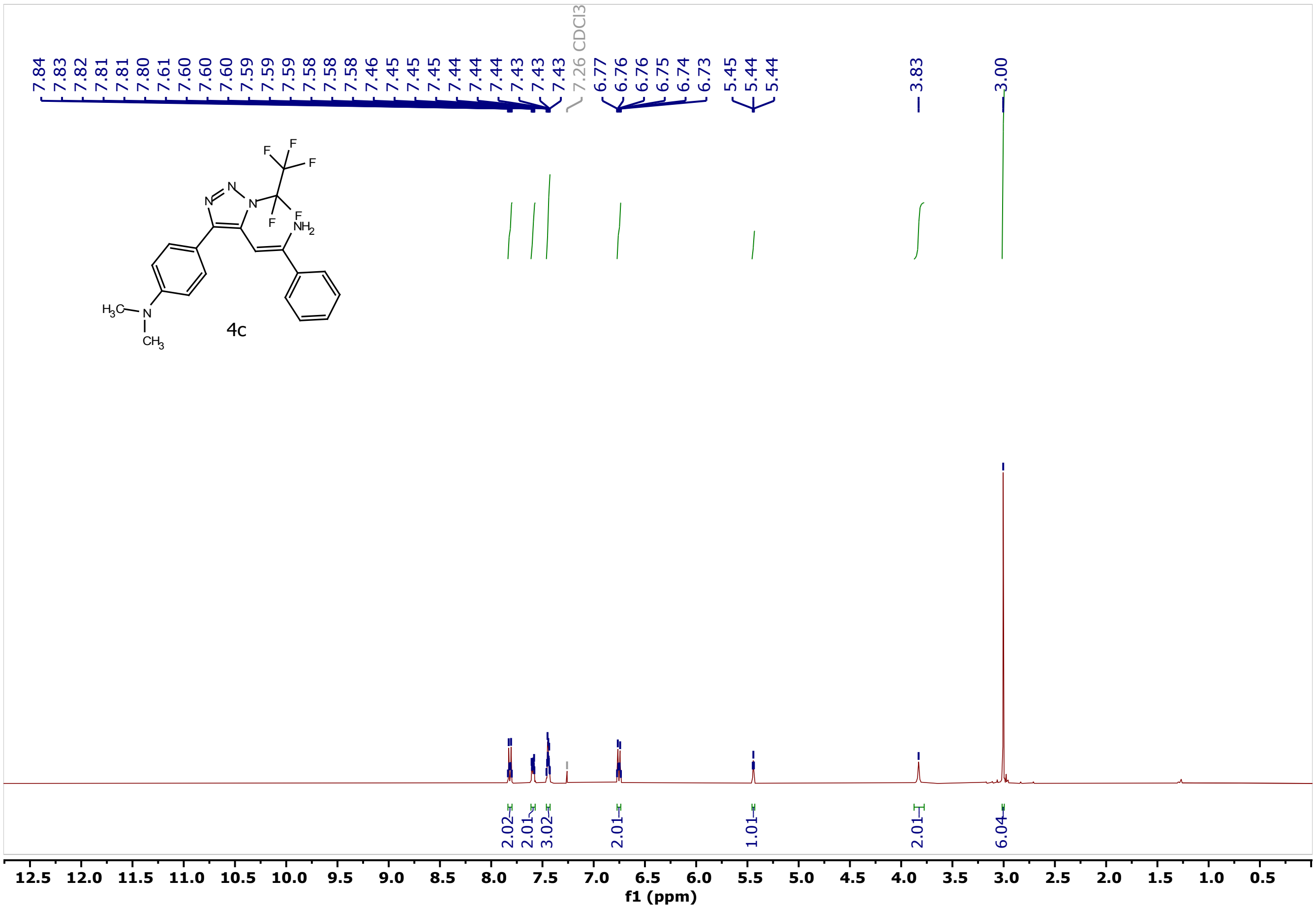

Figure 39. ^{13}C NMR spectrum of **4c** ($CDCl_3$, 101 MHz)

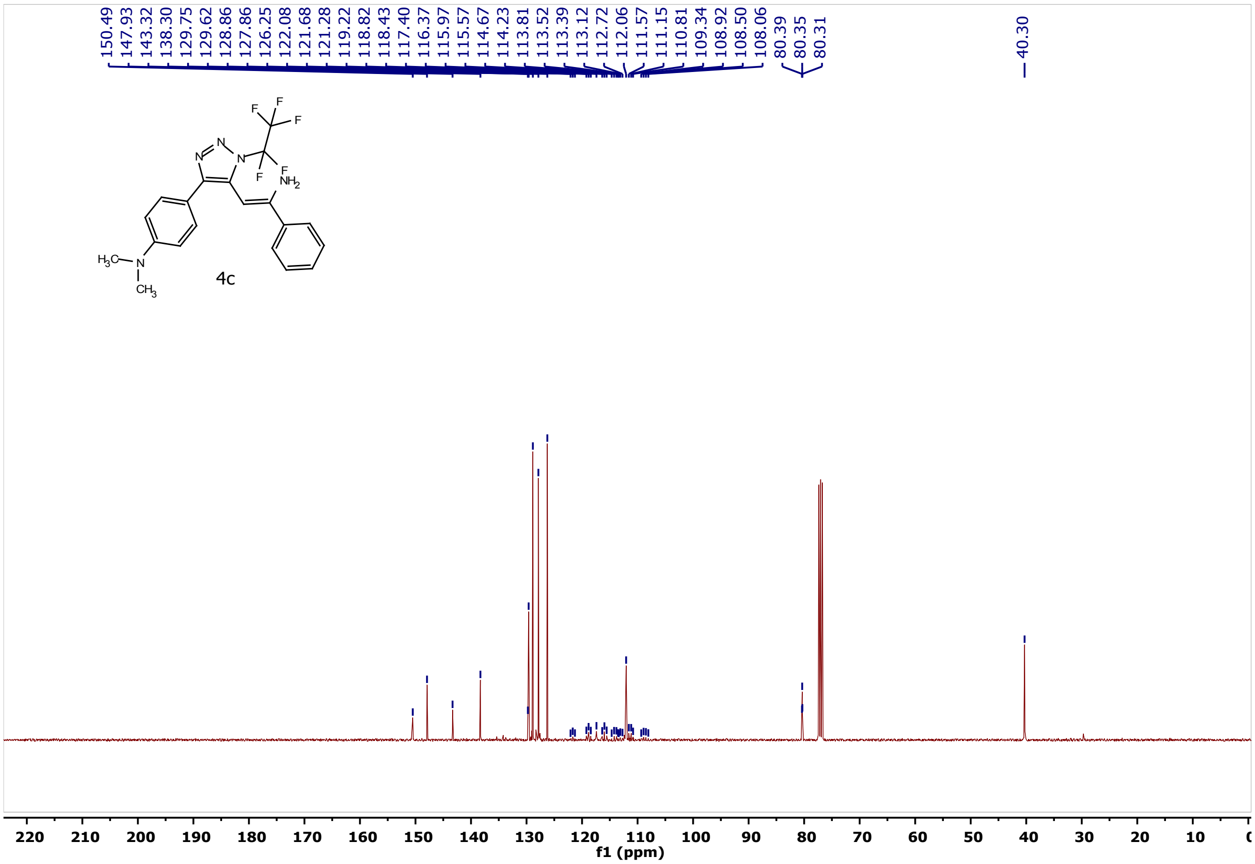

Figure 40. ^{19}F NMR spectrum of **4c** ($CDCl_3$, 377 MHz)

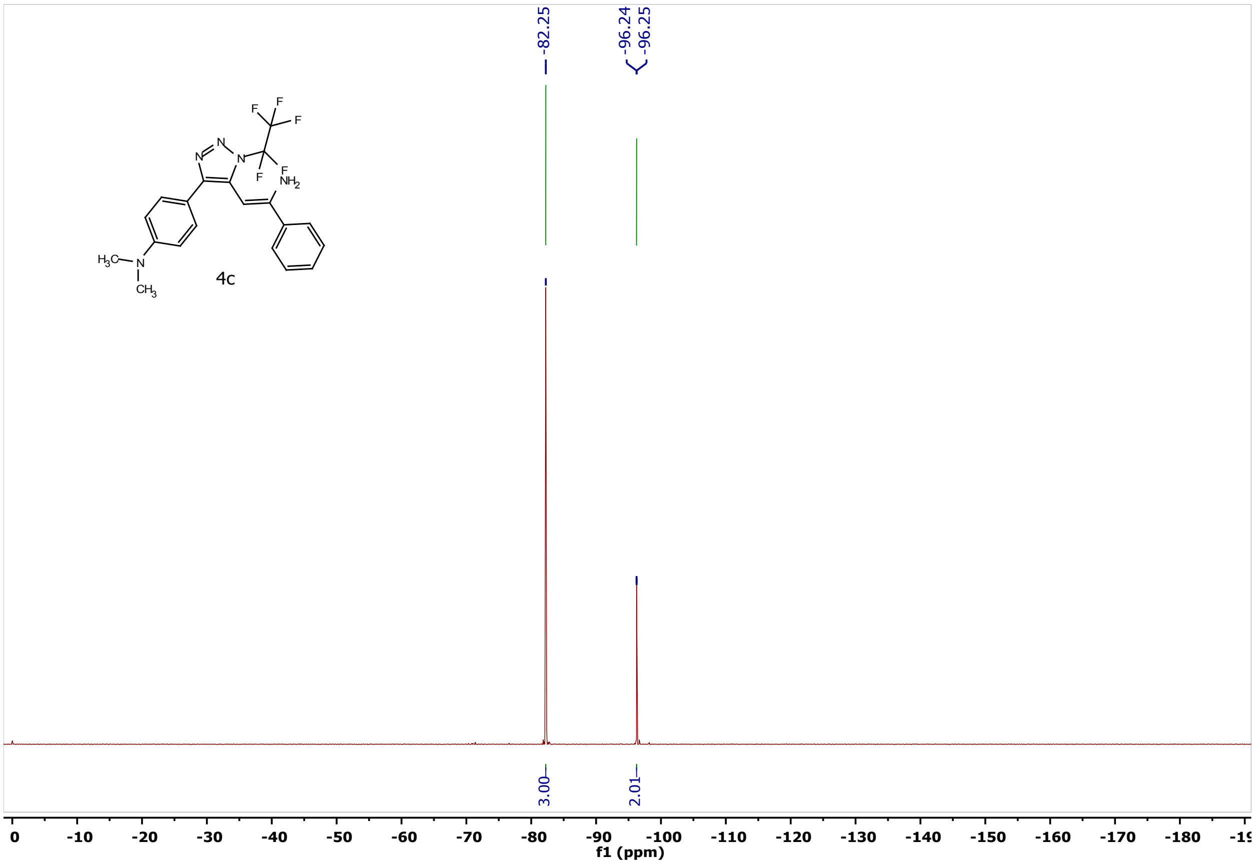

Figure 41. ^{1}H NMR spectrum of **4d** ($CDCl_3$, 400 MHz)

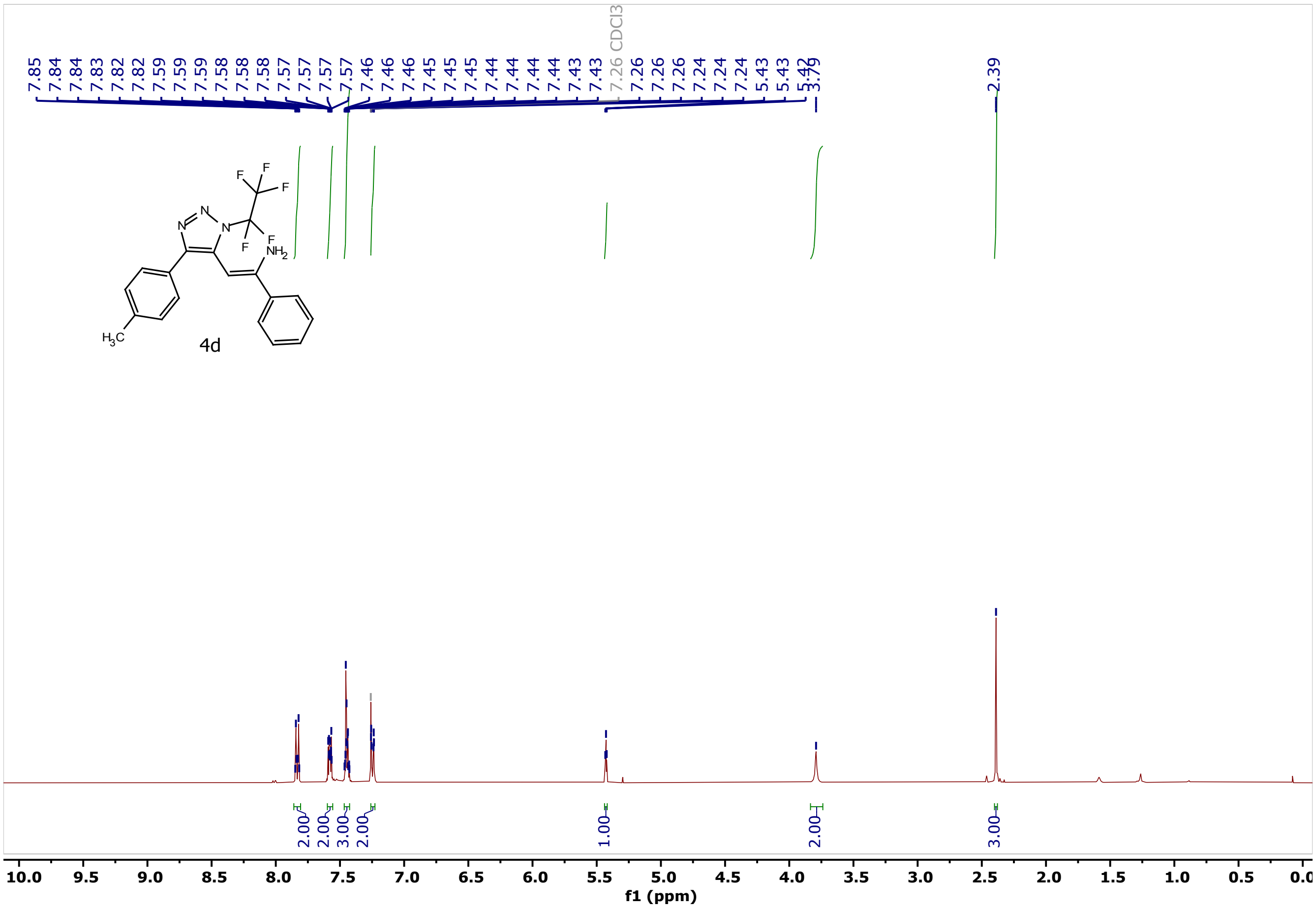

Figure 42. ^{13}C NMR spectrum of **4d** ($CDCl_3$, 101 MHz)

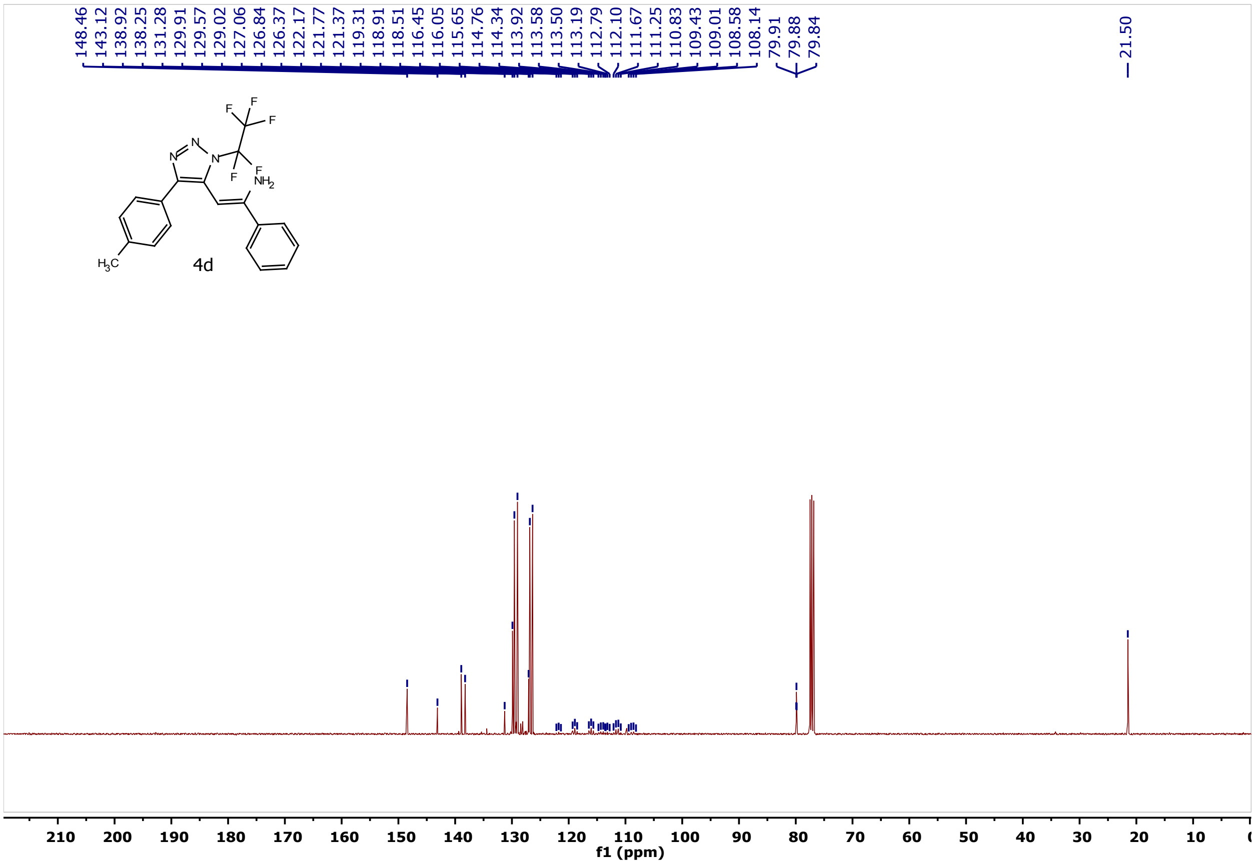

Figure 43. ^{19}F NMR spectrum of **4d** ($CDCl_3$, 377 MHz)

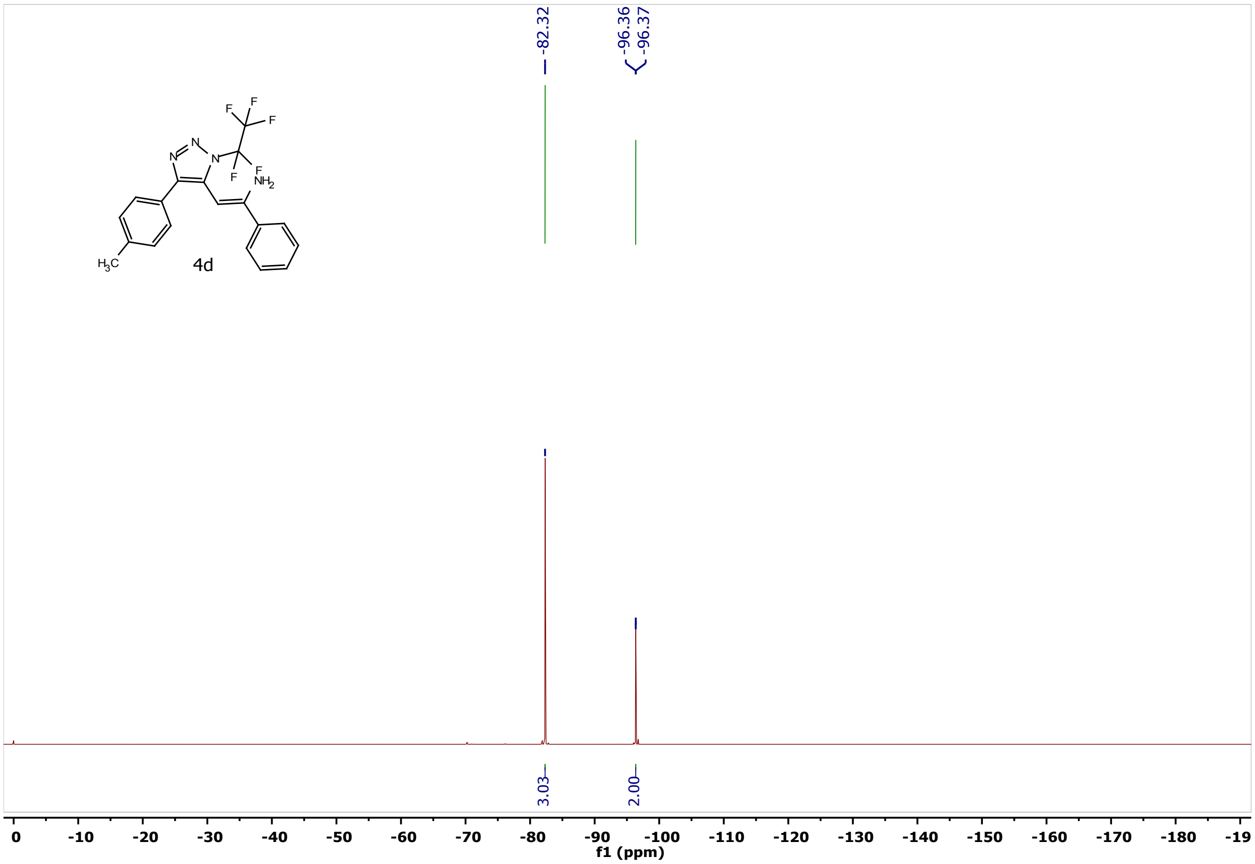

Figure 44. ^{1}H NMR spectrum of **4e** ($CDCl_3$, 400 MHz)

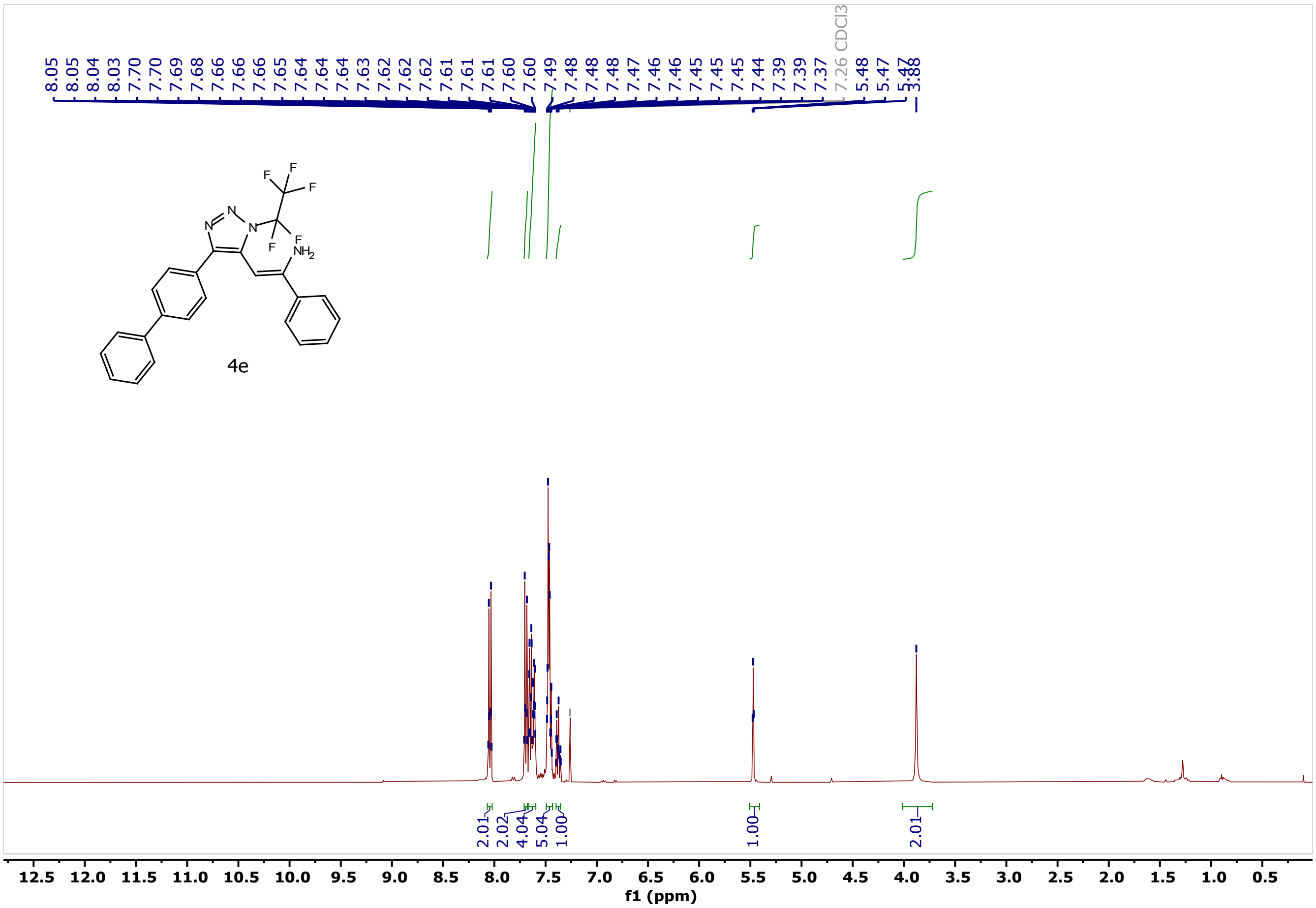

Figure 45. ^{13}C NMR spectrum of **4e** ($CDCl_3$, 101 MHz)

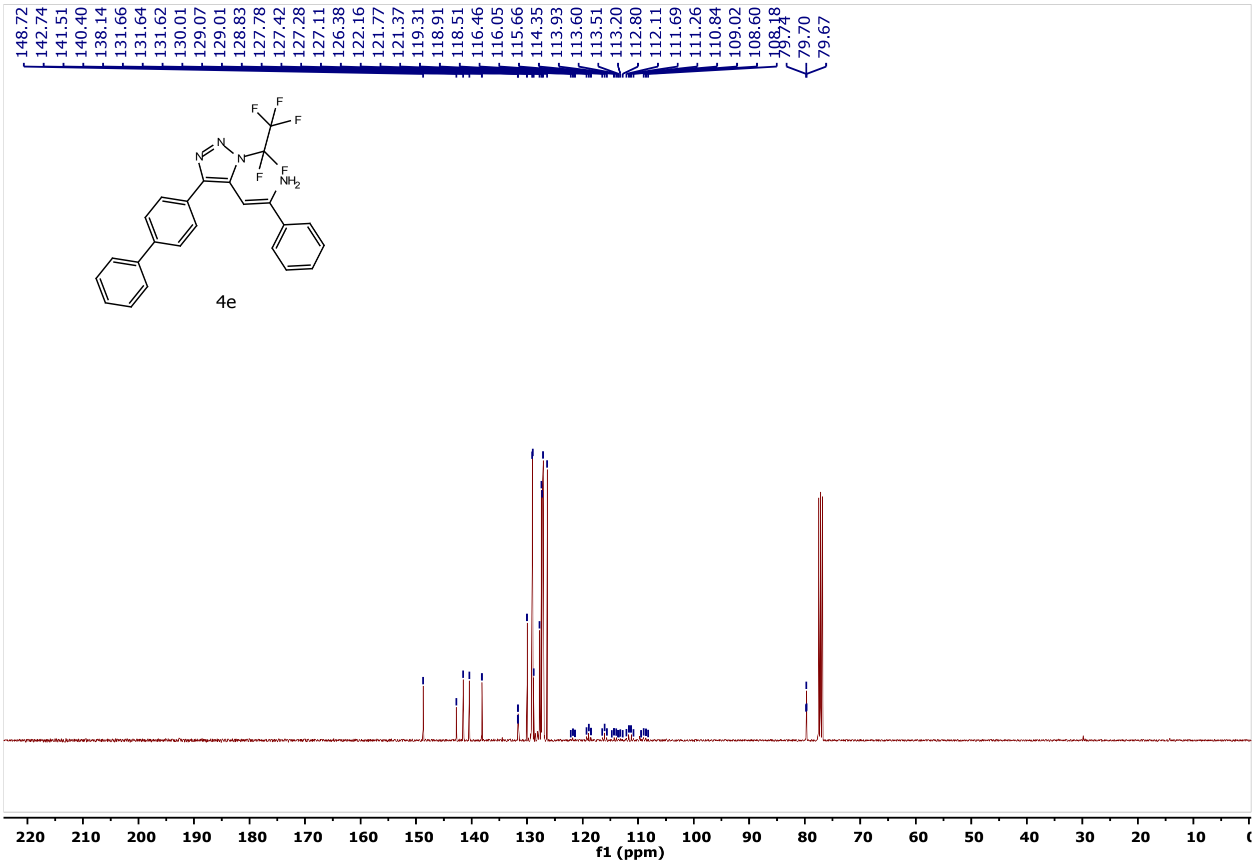

Figure 46. ^{19}F NMR spectrum of **4e** ($CDCl_3$, 377 MHz)

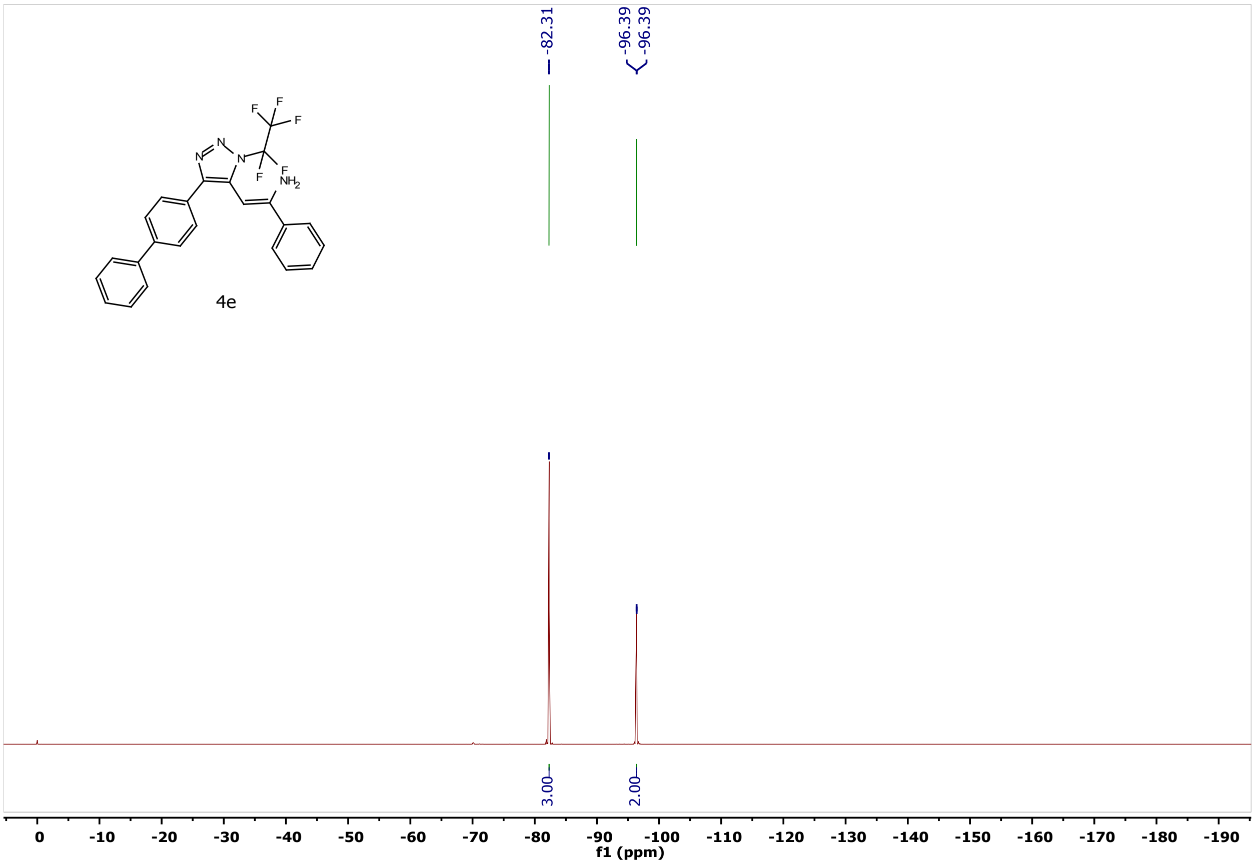

Figure 47. ^{1}H NMR spectrum of **4f** ($CDCl_3$, 400 MHz)

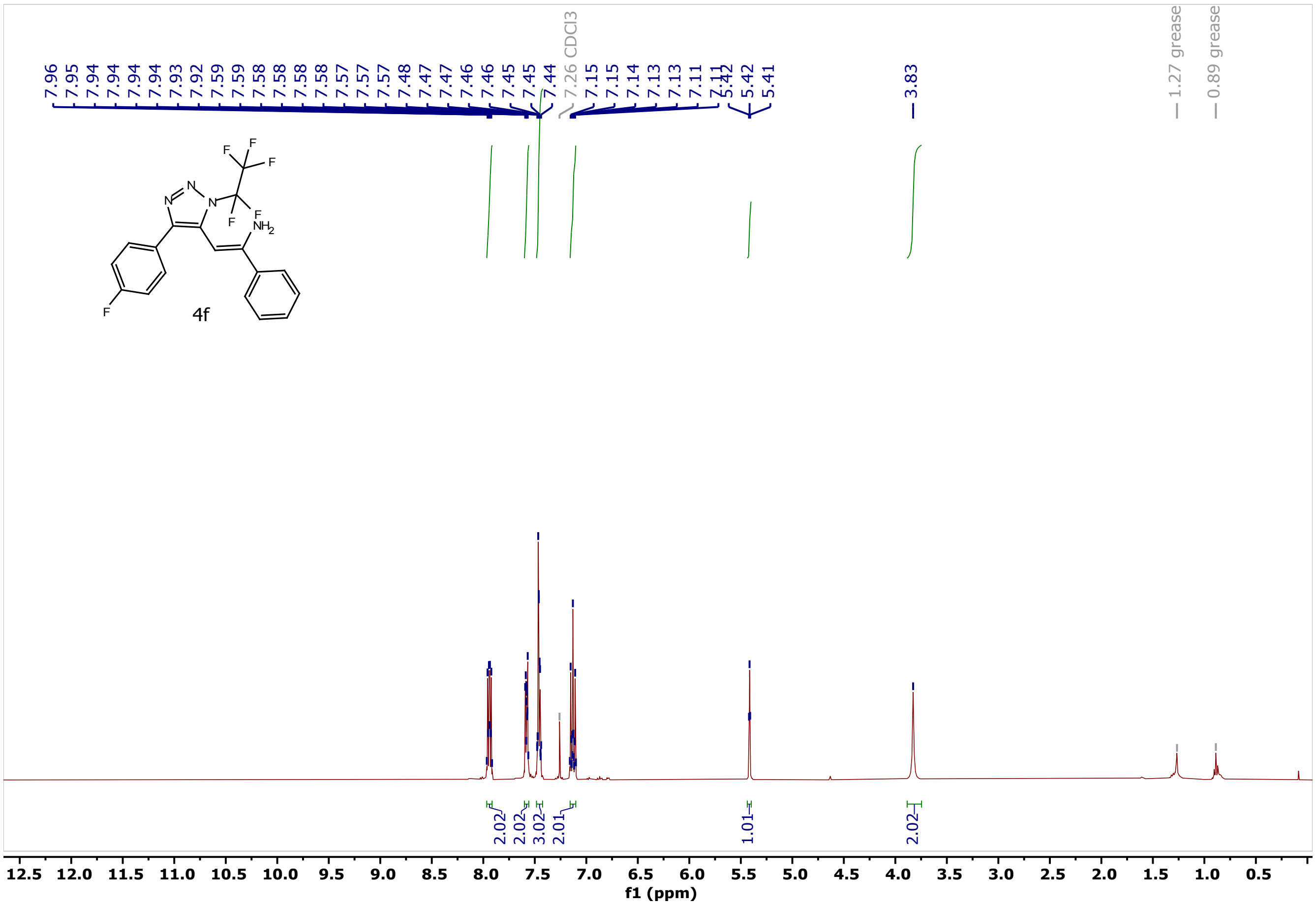

Figure 48. ^{13}C NMR spectrum of **4f** ($CDCl_3$, 101 MHz)

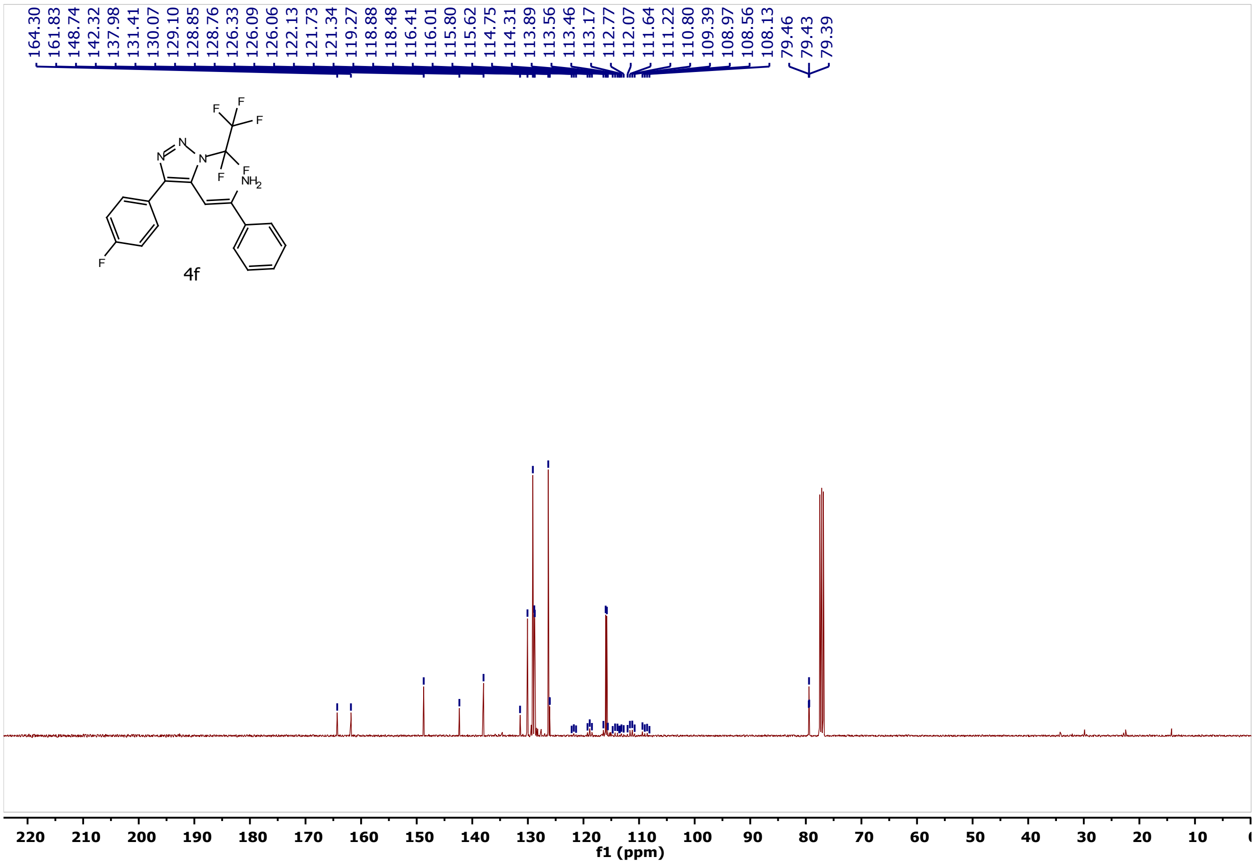

Figure 49. ^{19}F NMR spectrum of **4f** ($CDCl_3$, 377 MHz)

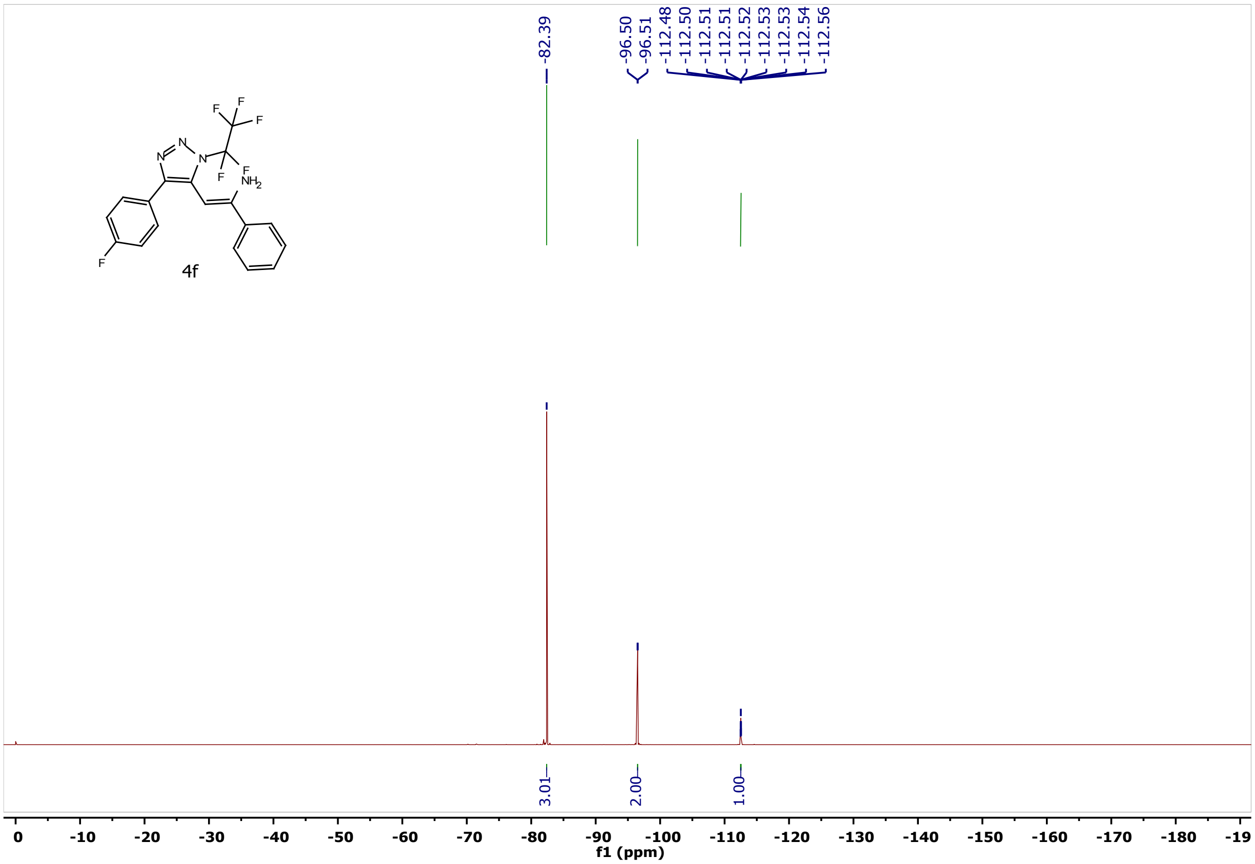

Figure 50. ^{1}H NMR spectrum of **4g** ($CDCl_3$, 400 MHz)

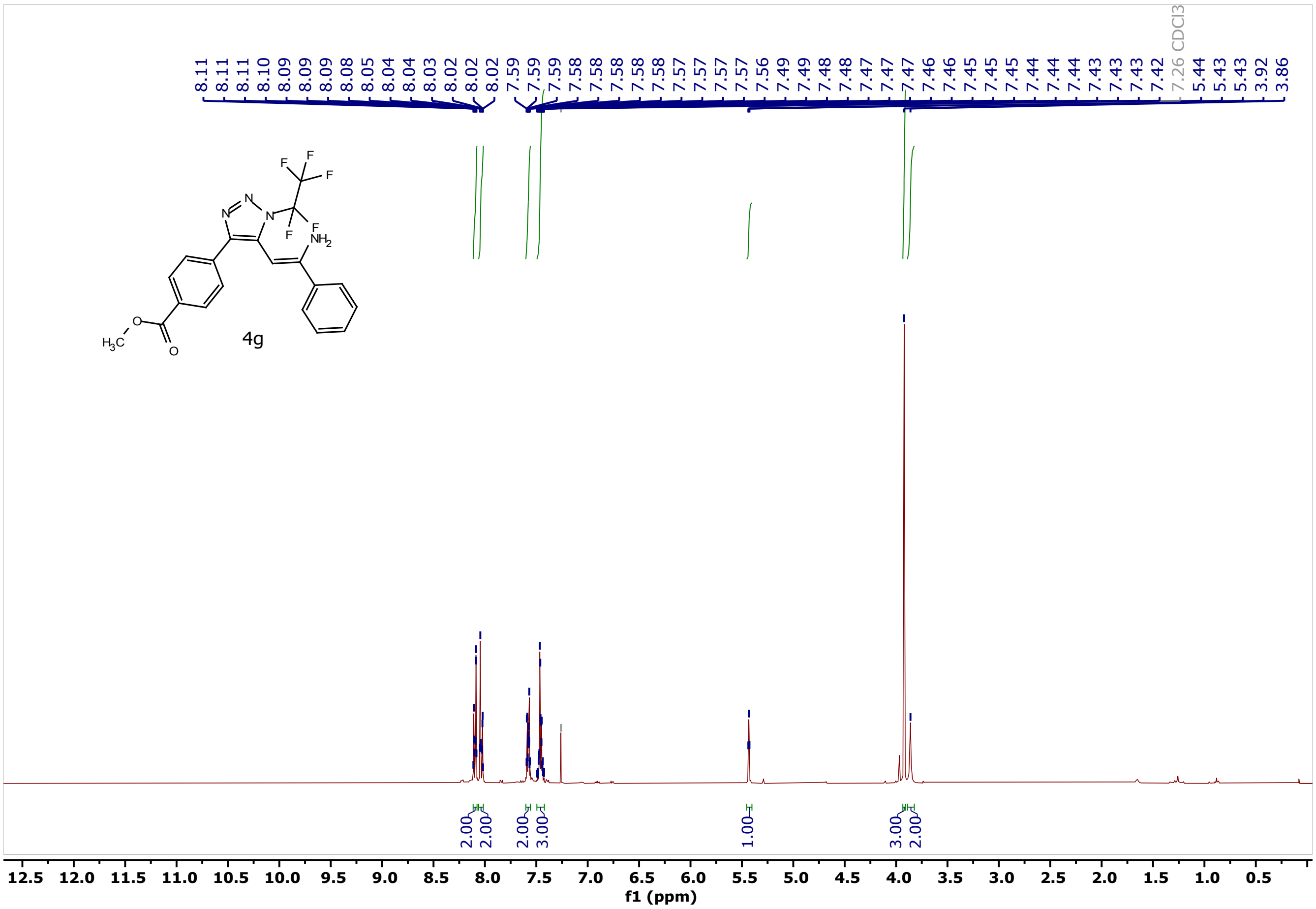

Figure 51. ^{13}C NMR spectrum of **4g** ($CDCl_3$, 101 MHz)

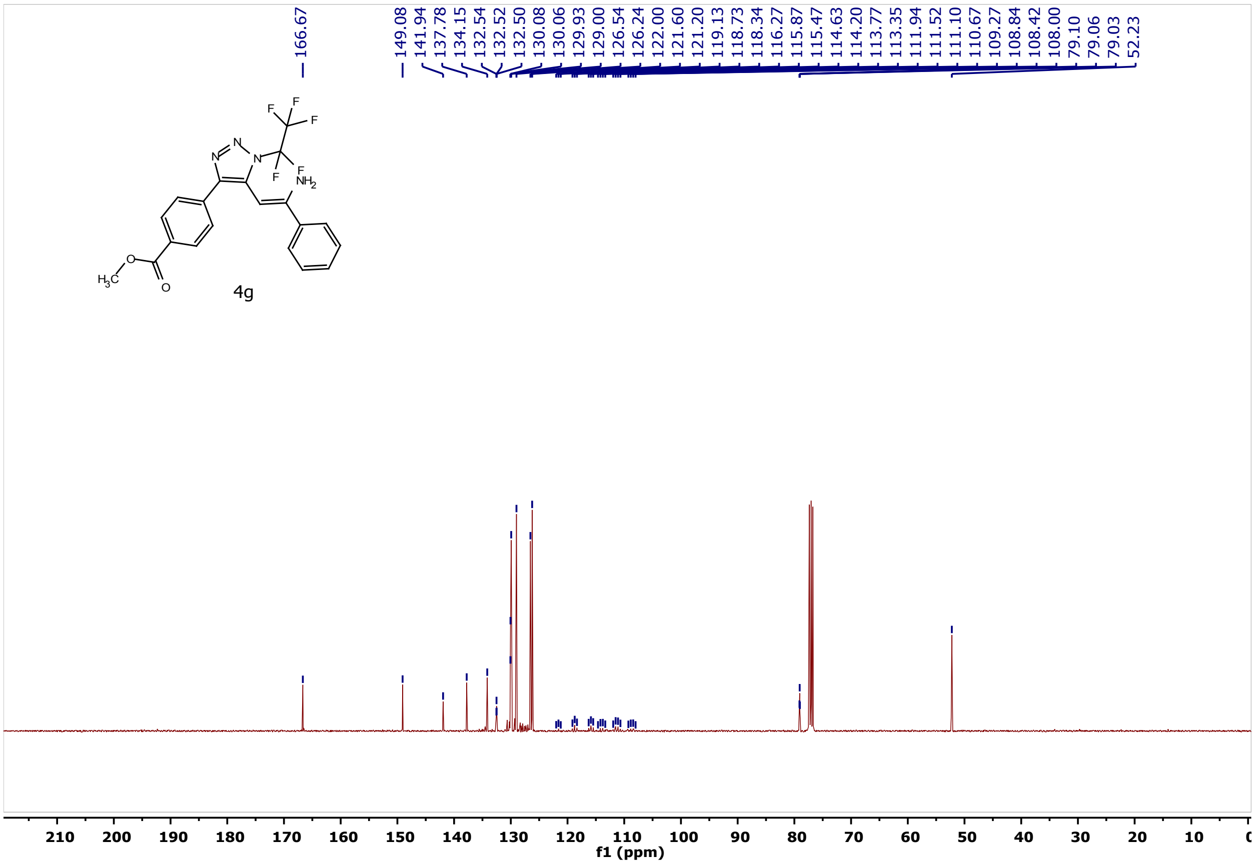

Figure 52. ^{19}F NMR spectrum of **4g** ($CDCl_3$, 377 MHz)

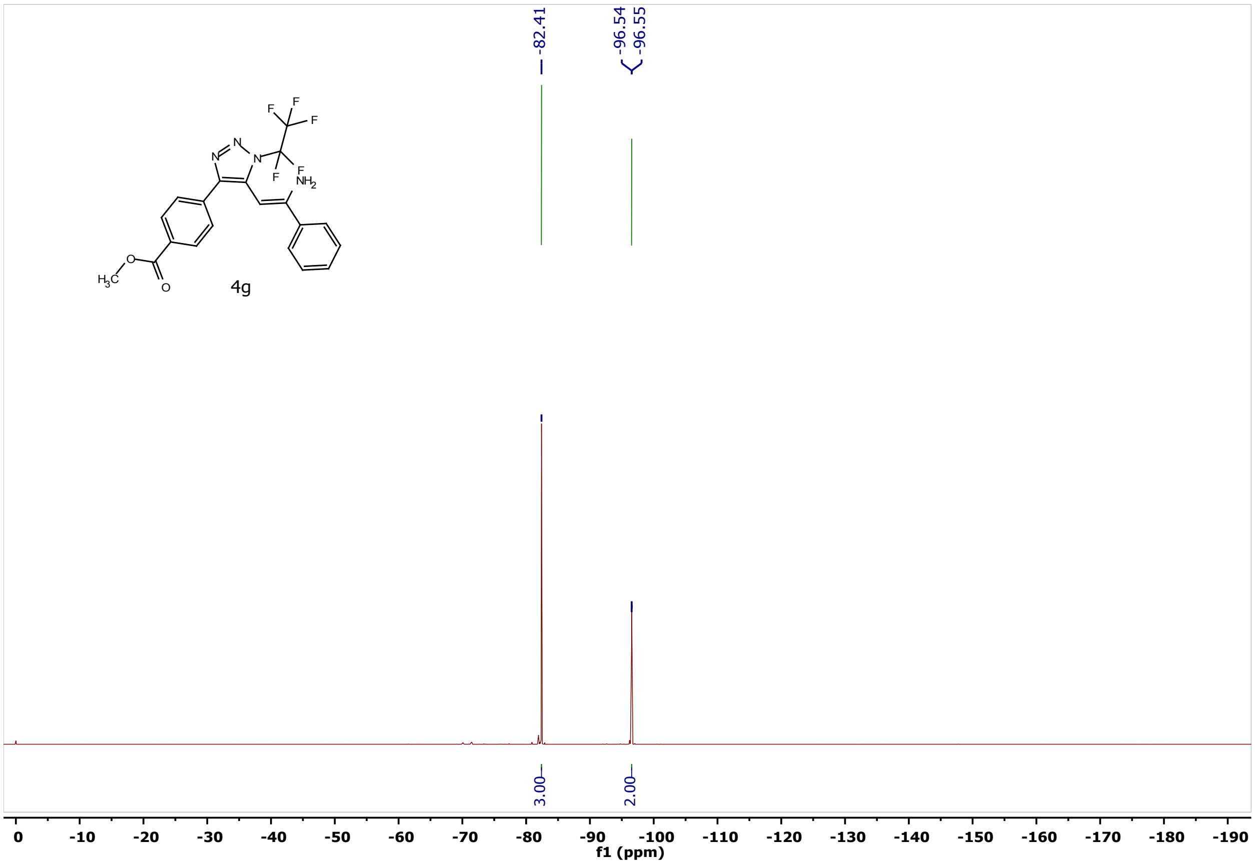

Figure 53. ^{1}H NMR spectrum of **4h** ($CDCl_3$, 400 MHz)

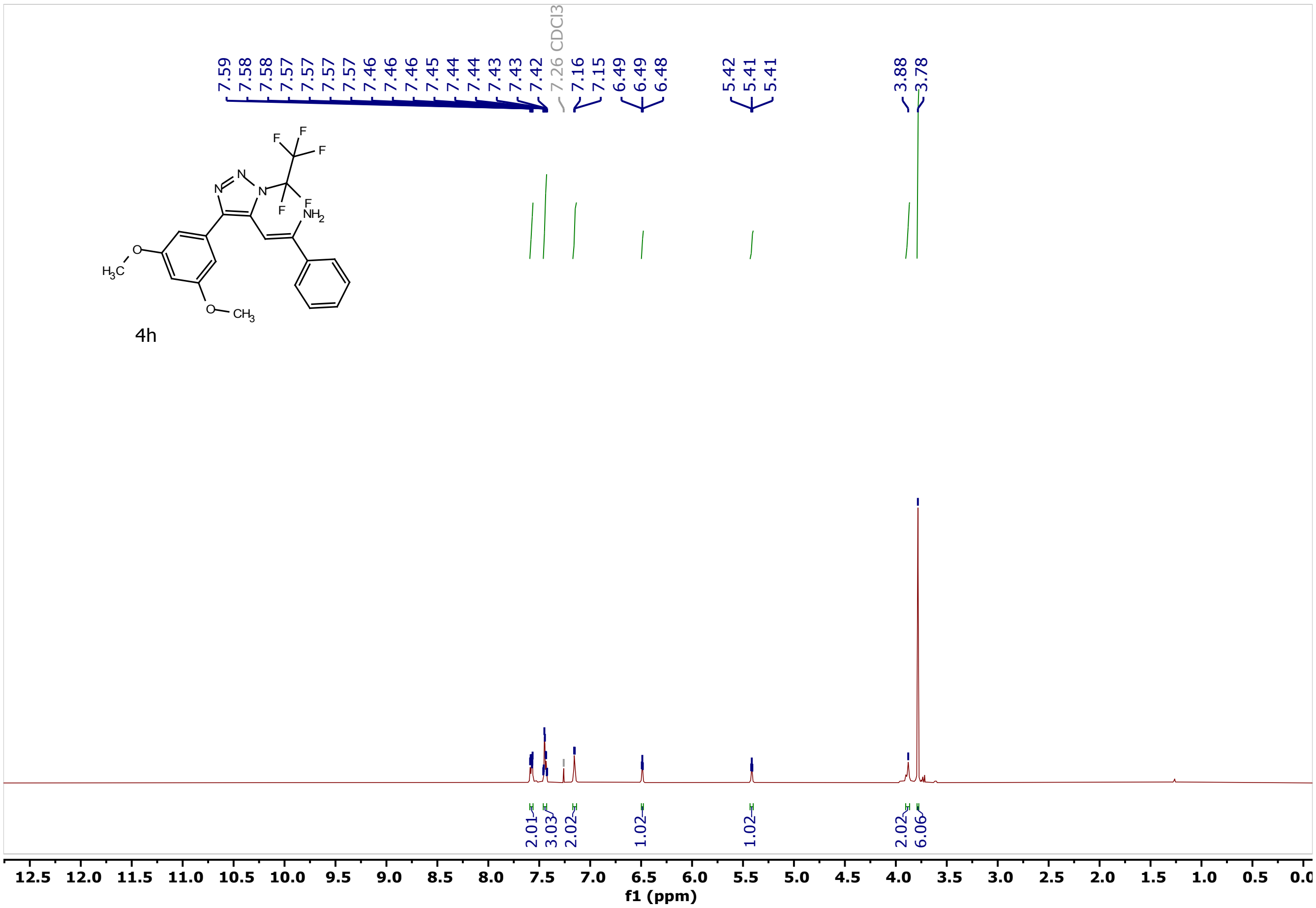

Figure 54. ^{13}C NMR spectrum of **4h** ($CDCl_3$, 101 MHz)

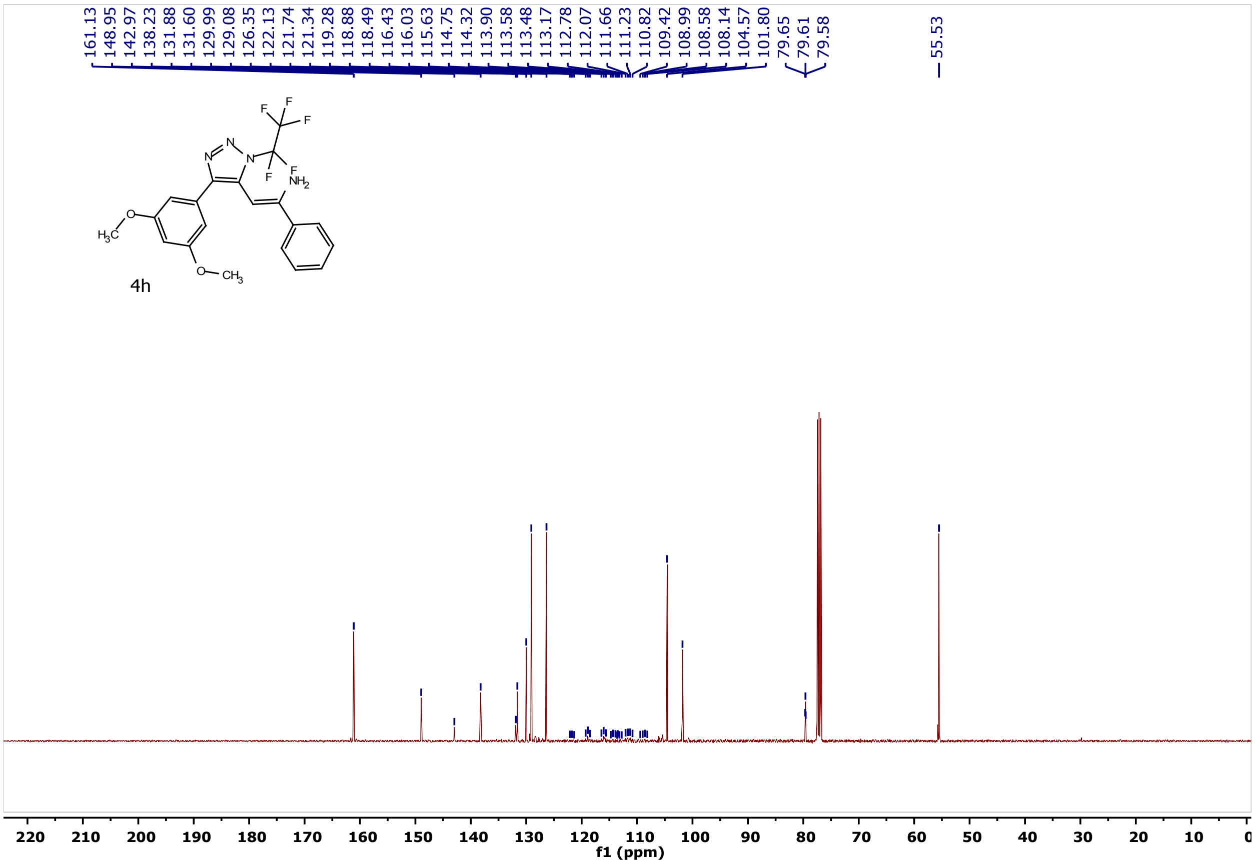

Figure 55. ^{19}F NMR spectrum of **4h** ($CDCl_3$, 377 MHz)

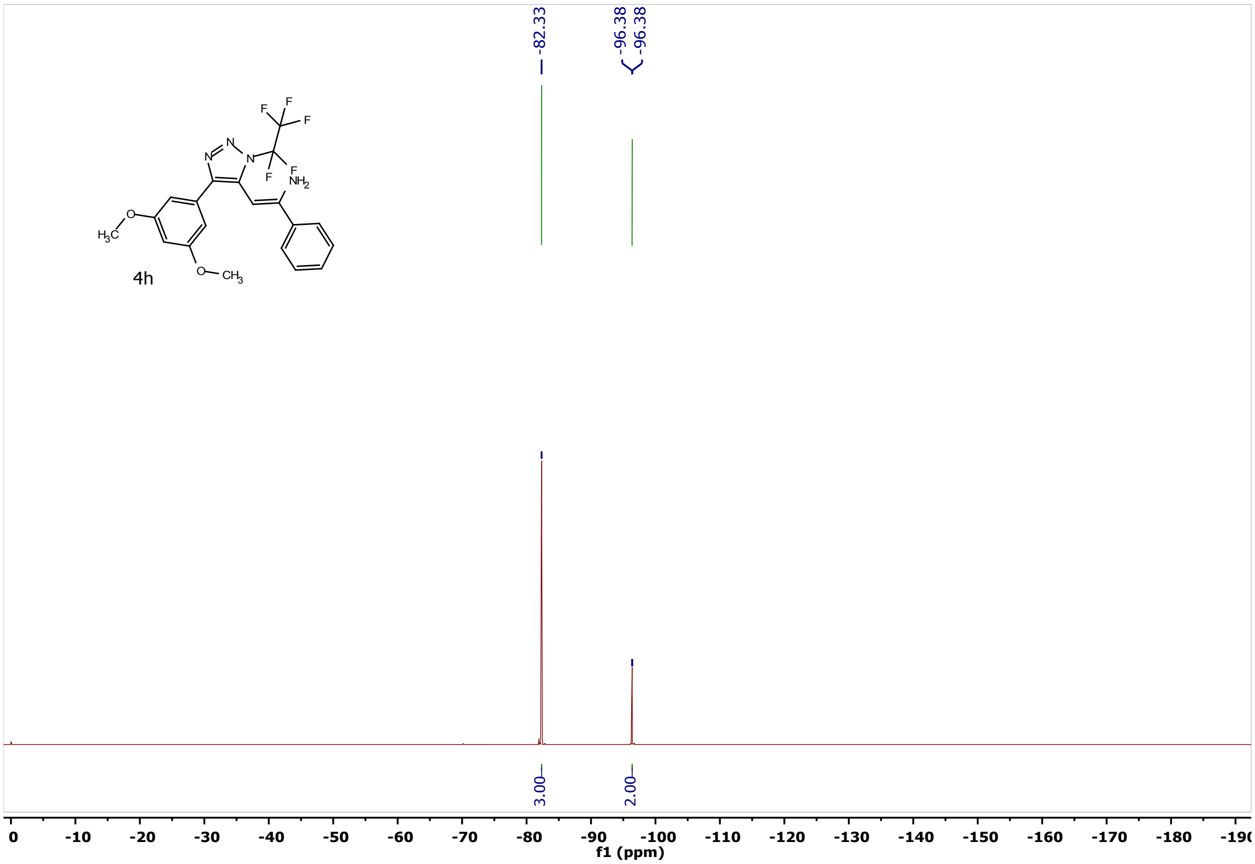

Figure 56. ^{1}H NMR spectrum of **4i** ($CDCl_3$, 400 MHz)

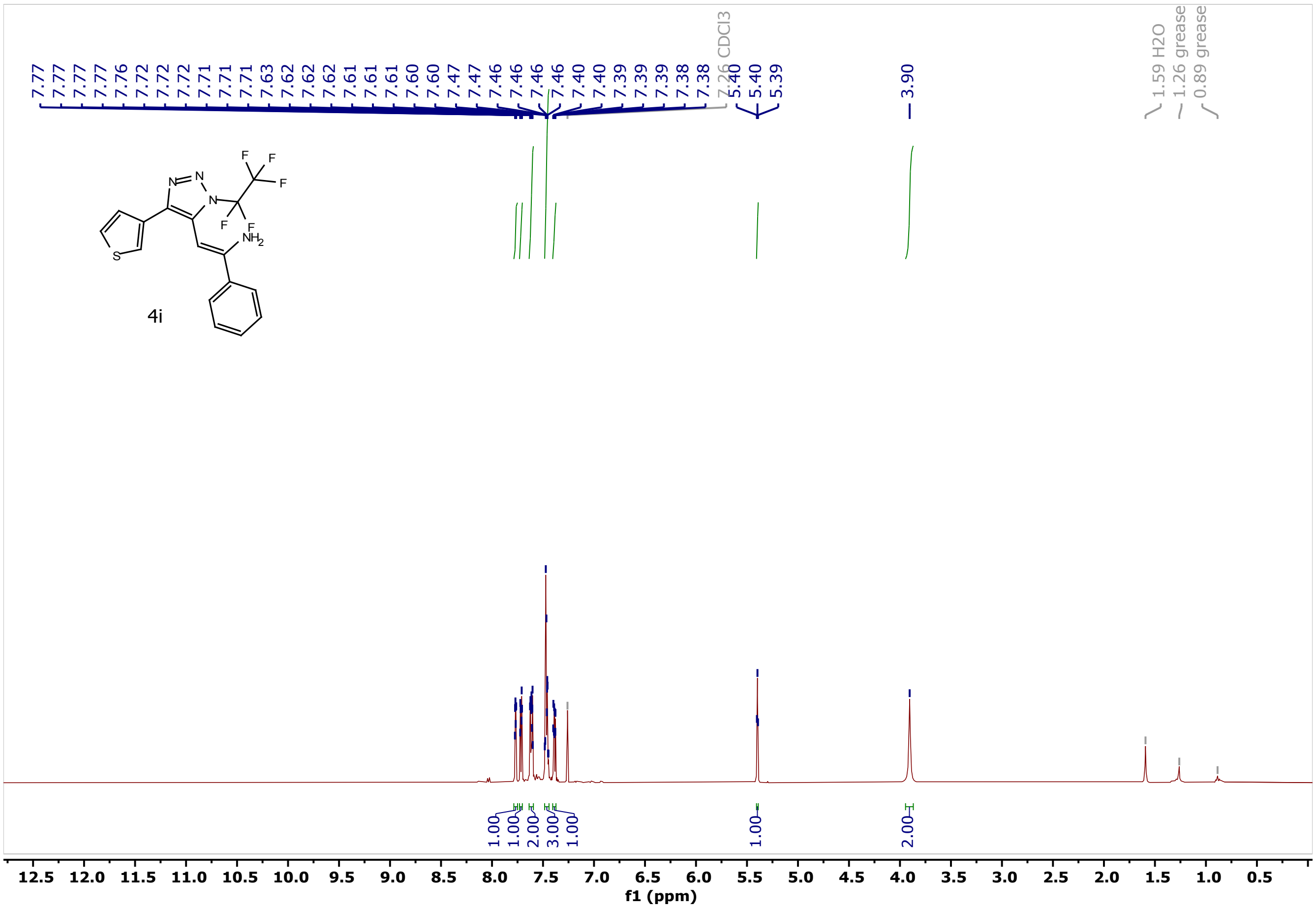

Figure 57. ^{13}C NMR spectrum of **4i** ($CDCl_3$, 101 MHz)

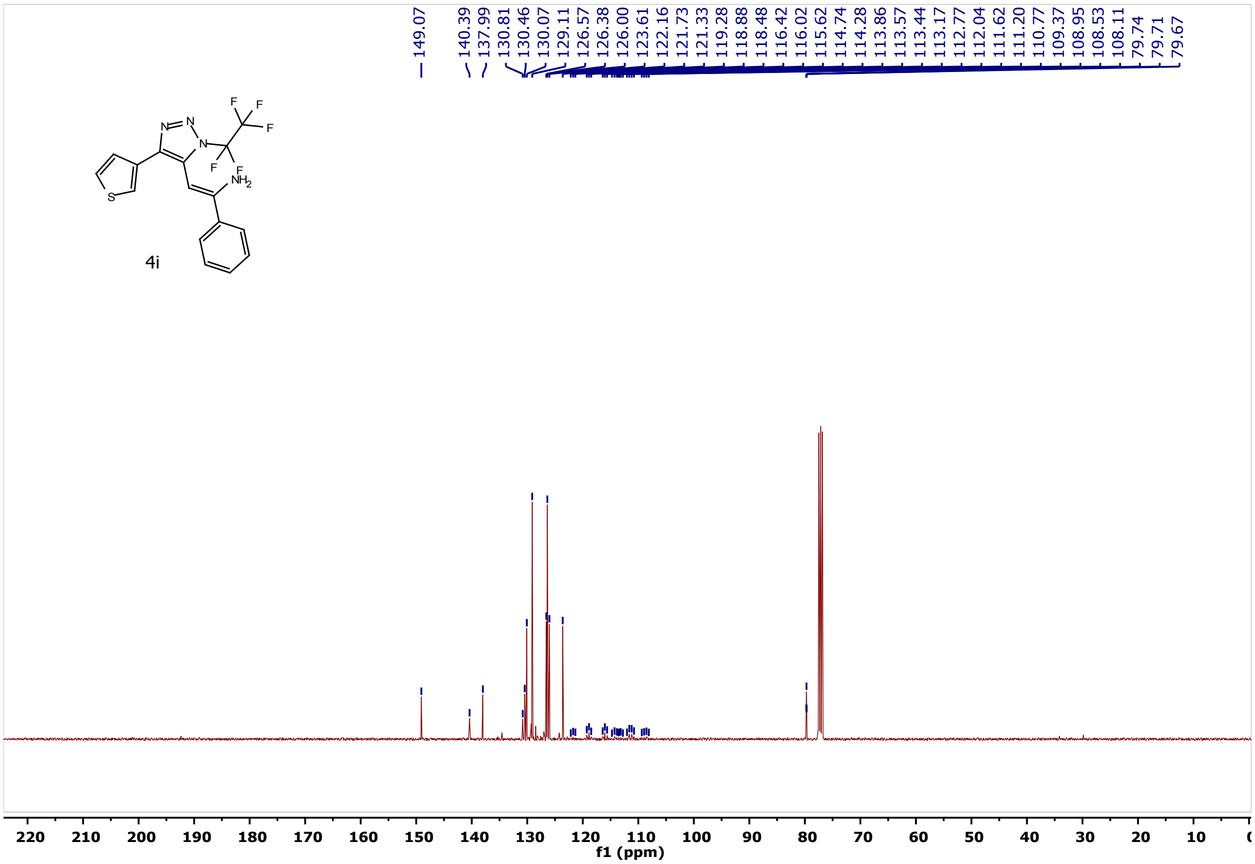

Figure 58. ^{19}F NMR spectrum of **4i** ($CDCl_3$, 377 MHz)

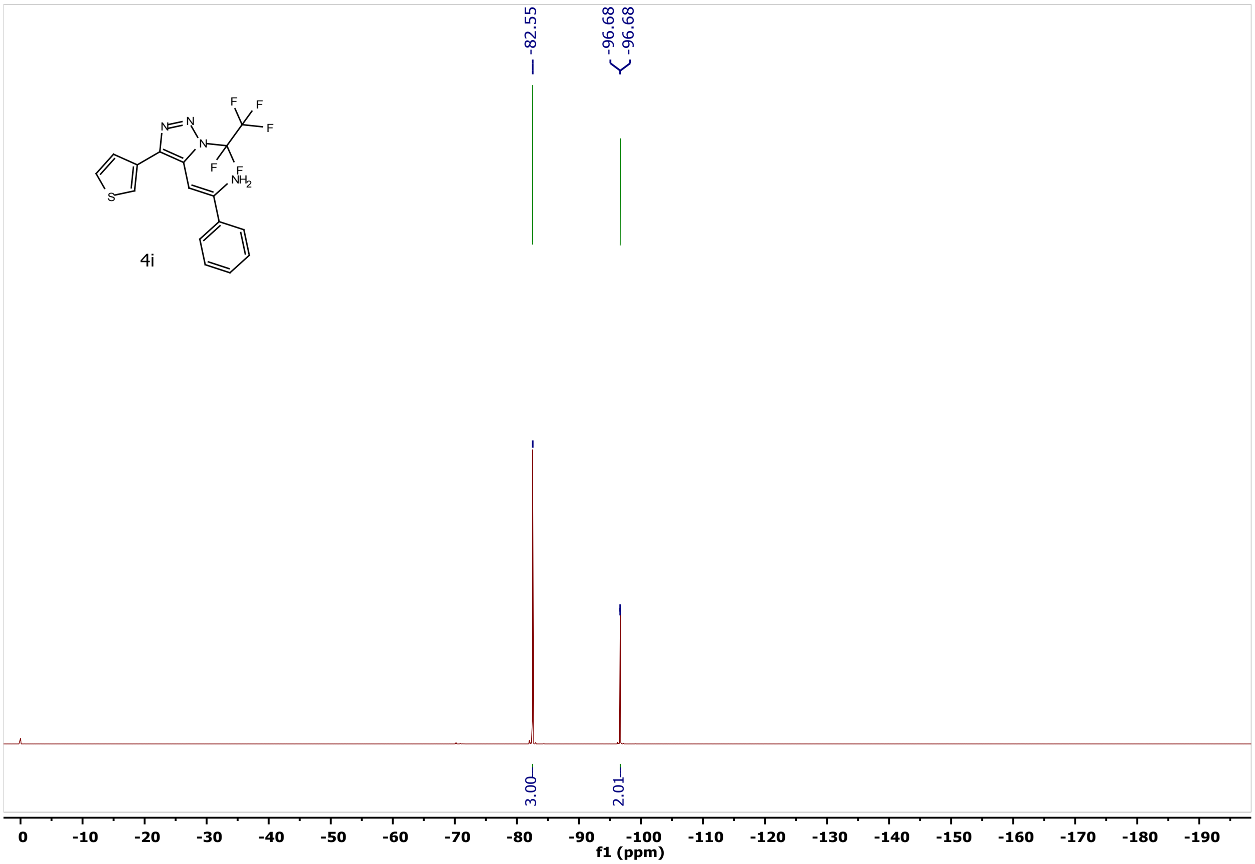

Figure 59. ^{1}H NMR spectrum of **4j** ($CDCl_3$, 400 MHz)

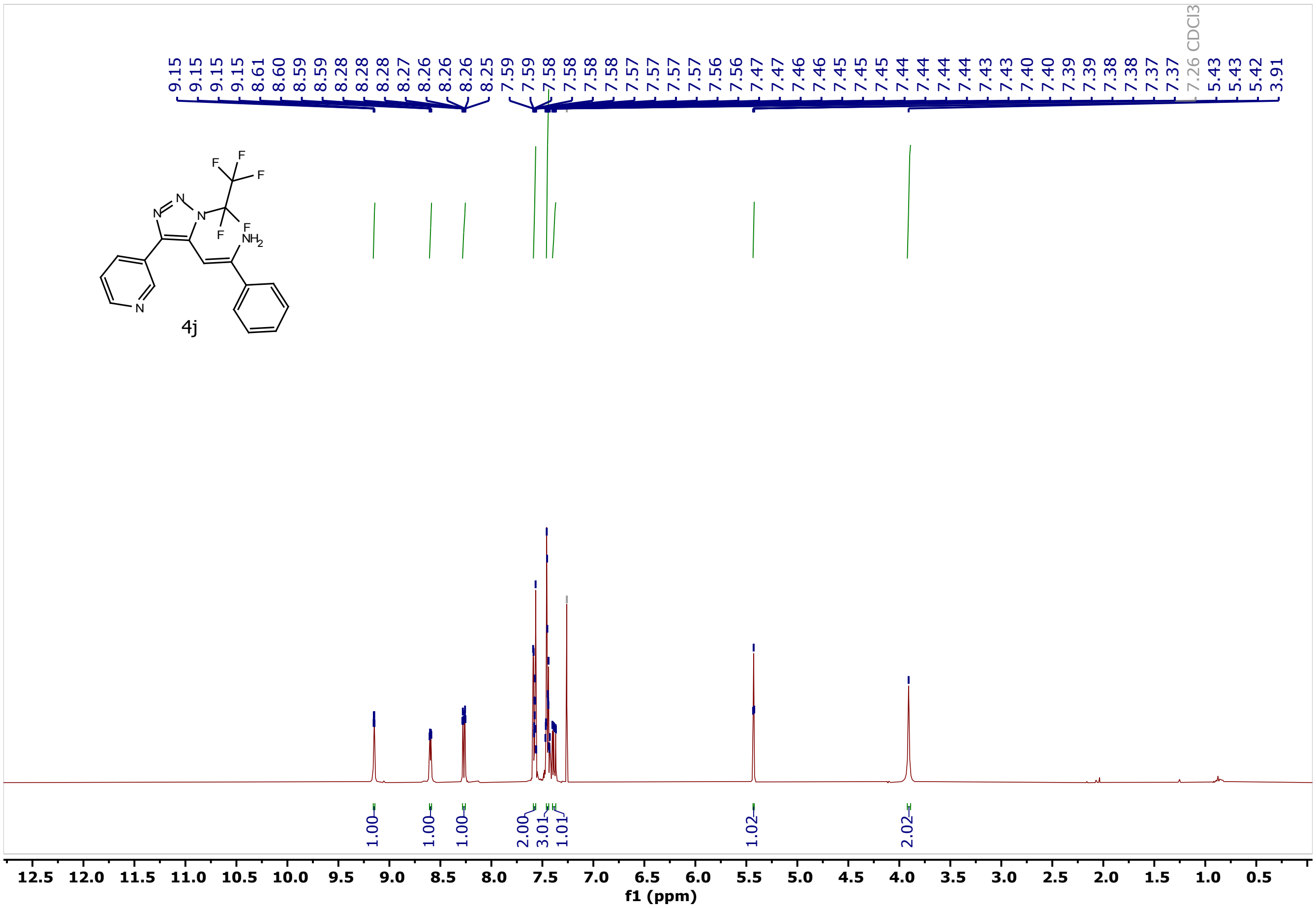

Figure 60. ^{13}C NMR spectrum of **4j** ($CDCl_3$, 101 MHz)

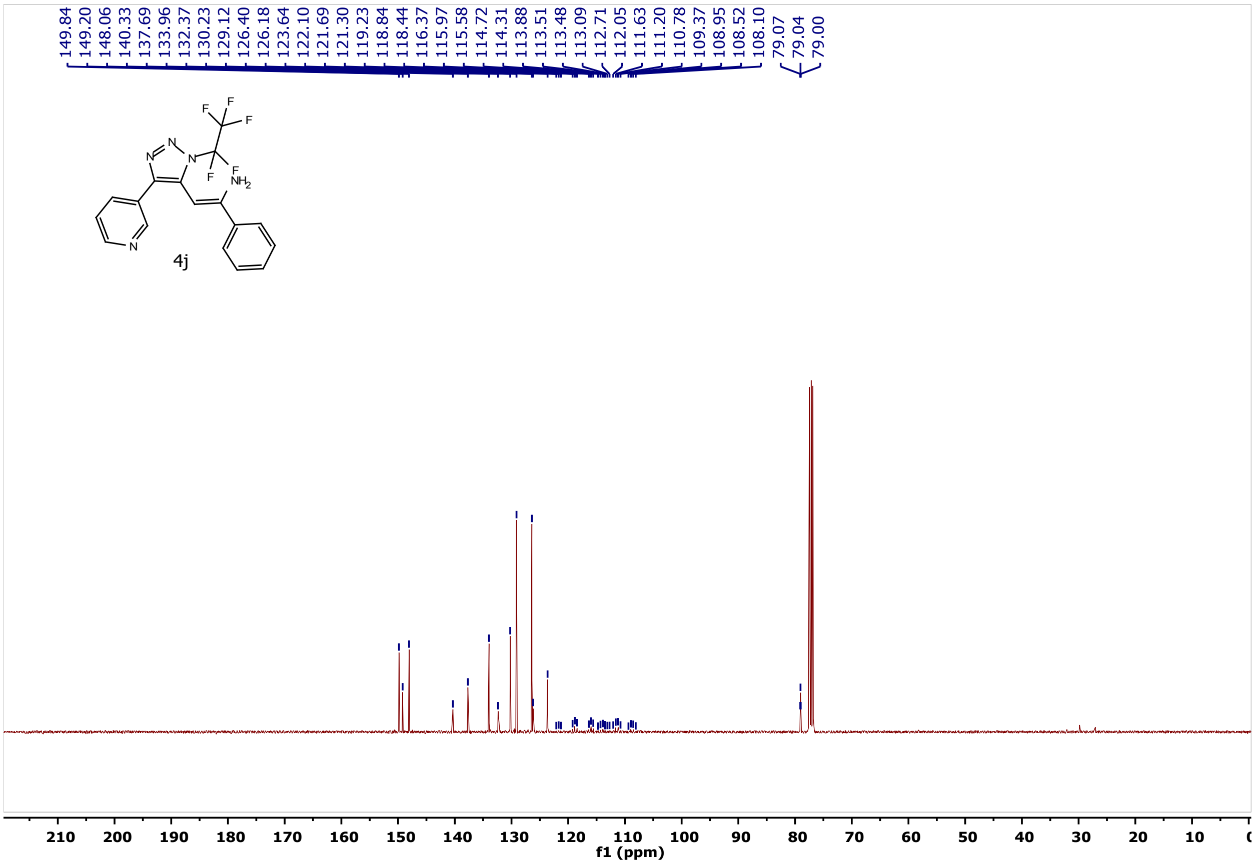

Figure 61. ^{19}F NMR spectrum of **4j** ($CDCl_3$, 377 MHz)

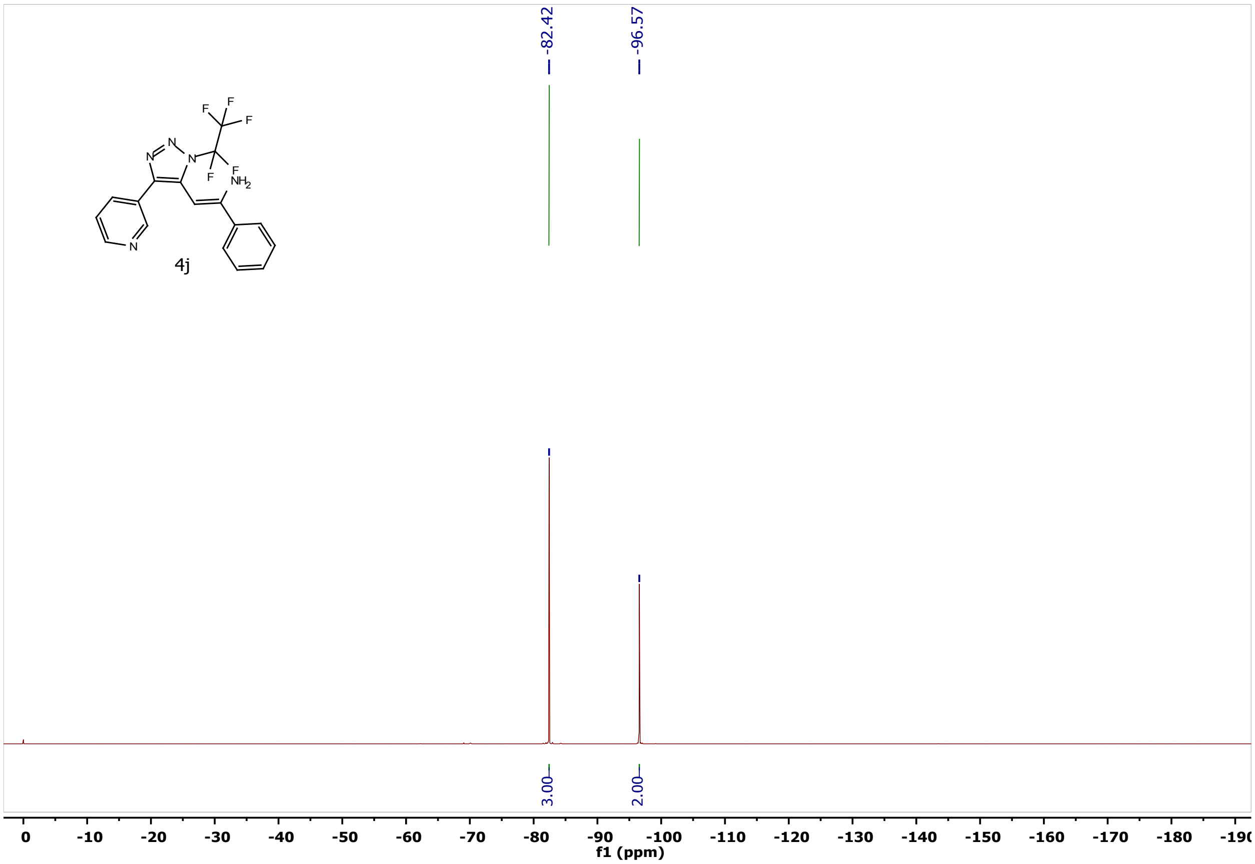

Figure 62. ^{1}H NMR spectrum of **4k** ($CDCl_3$, 400 MHz)

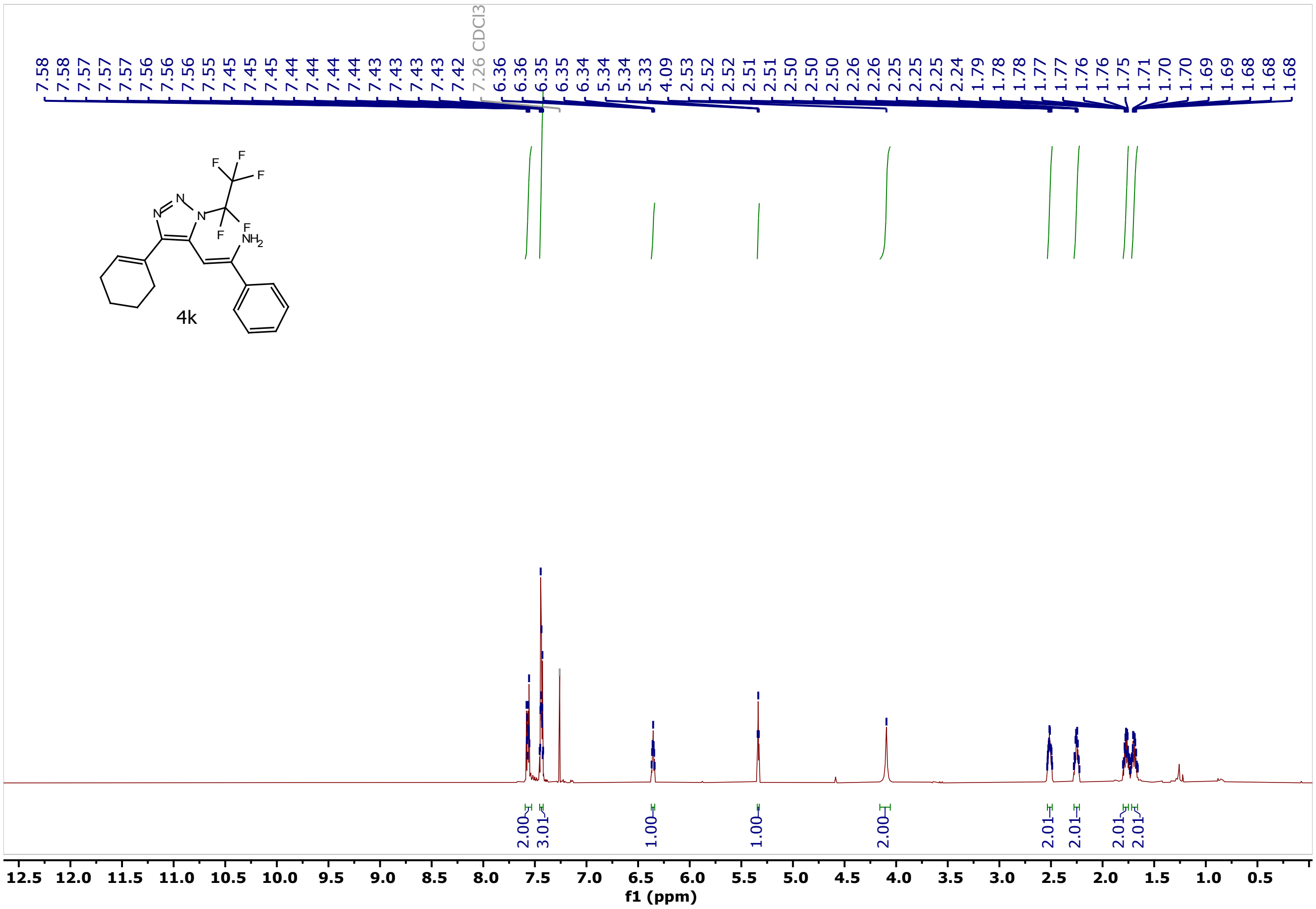

Figure 63. ^{13}C NMR spectrum of **4k** ($CDCl_3$, 101 MHz)

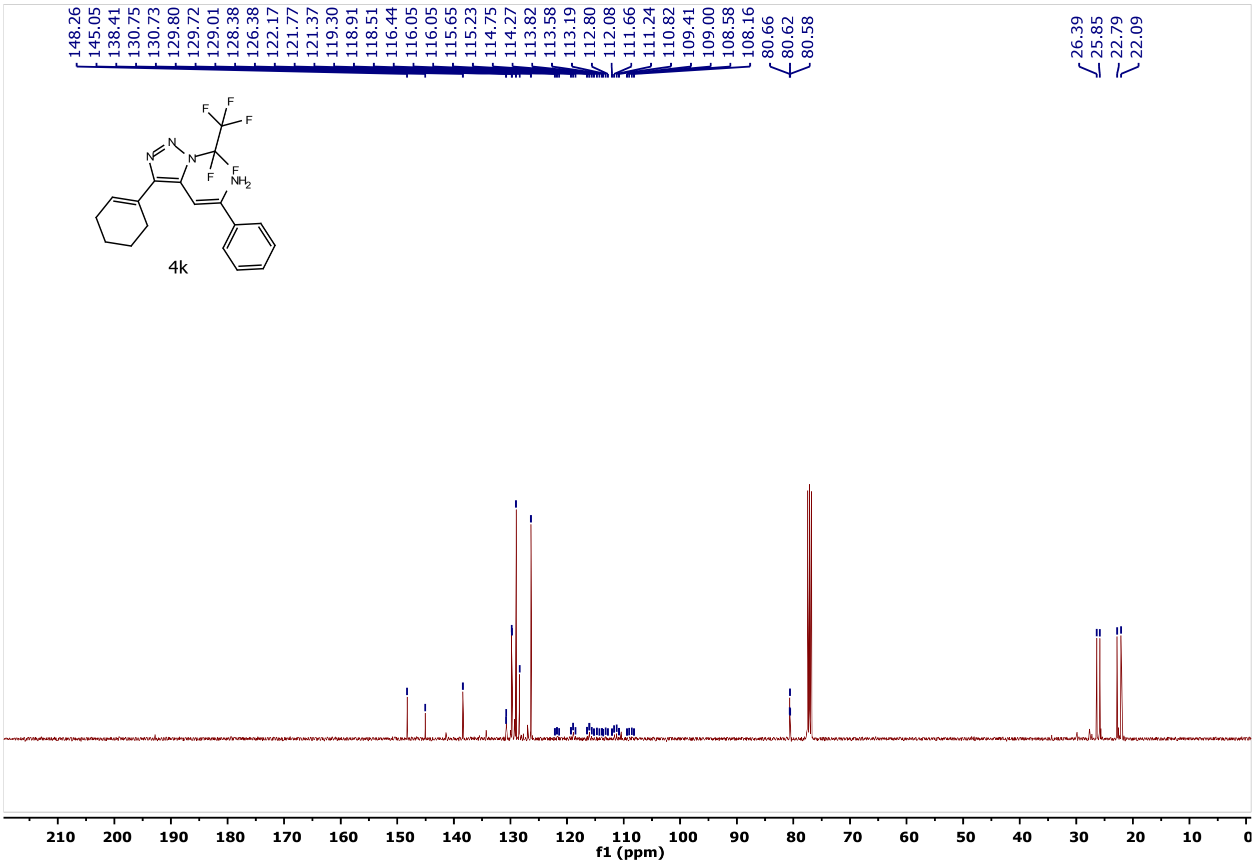

Figure 64. ^{19}F NMR spectrum of **4k** ($CDCl_3$, 377 MHz)

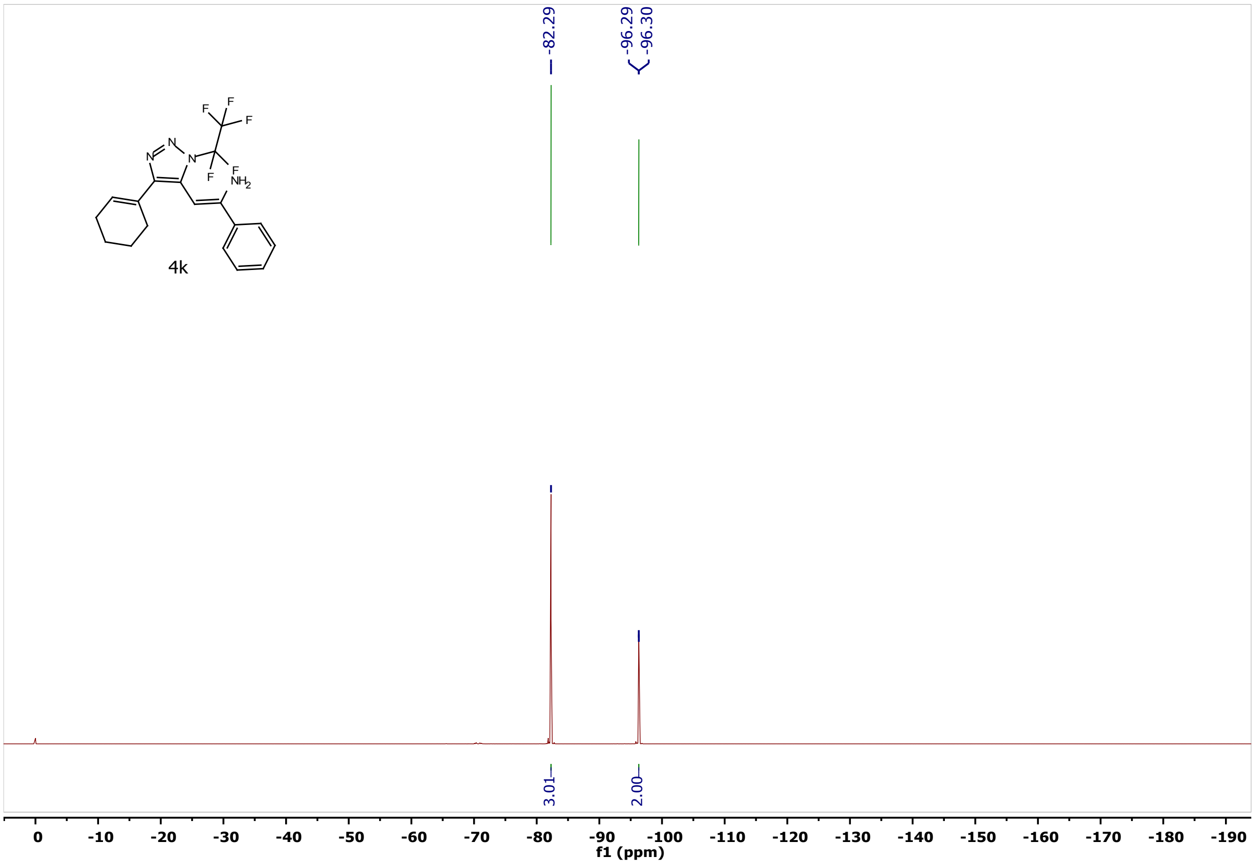

Figure 65. ^{1}H NMR spectrum of **4l** ($CDCl_3$, 400 MHz)

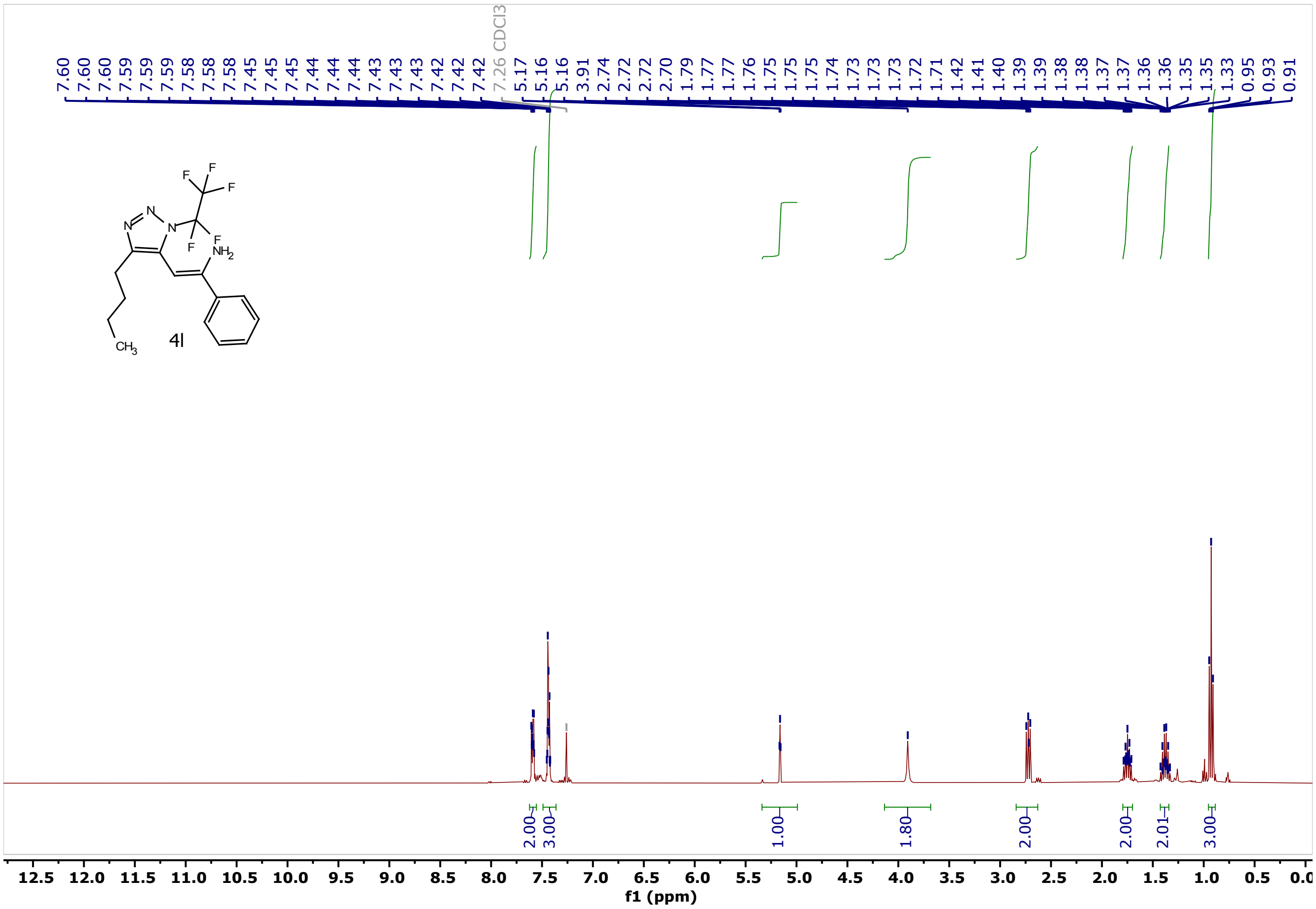

Figure 66. ^{13}C NMR spectrum of **4l** ($CDCl_3$, 101 MHz)

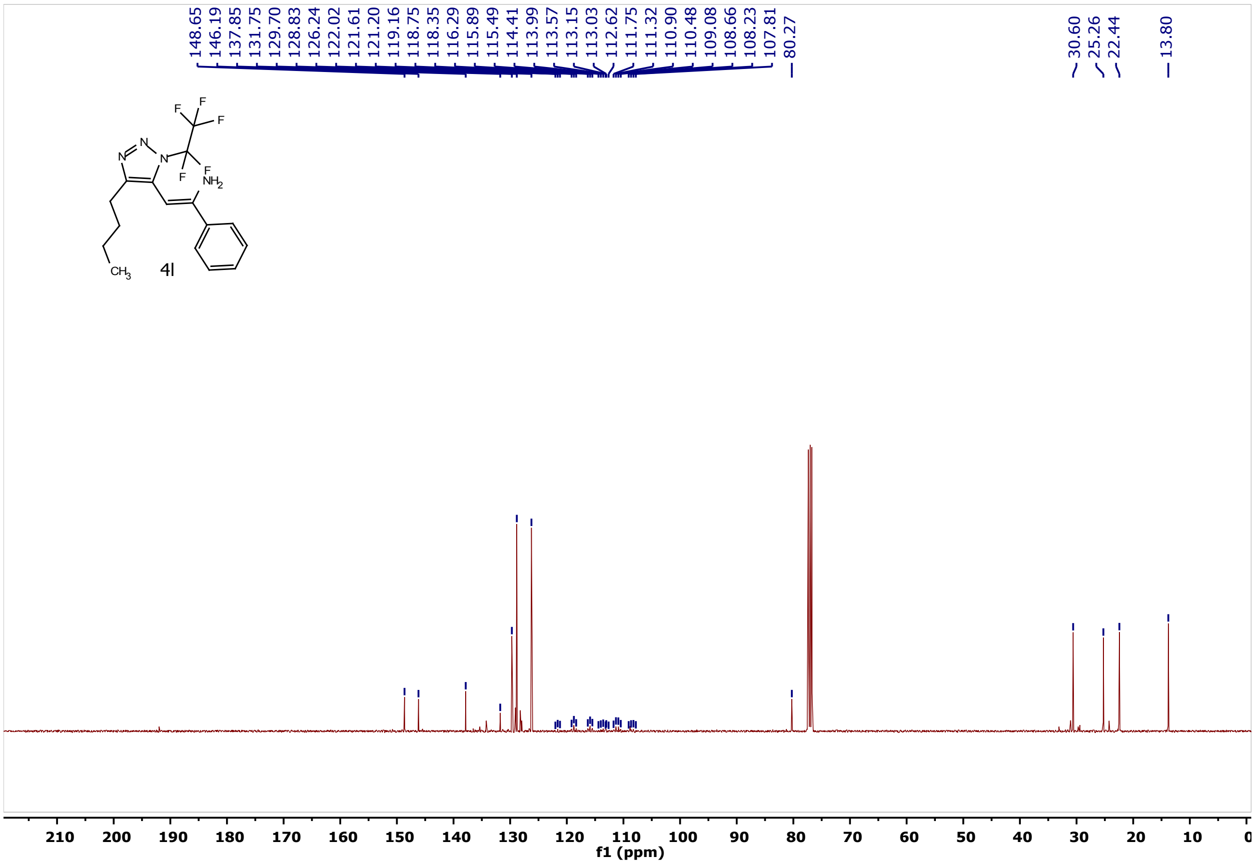

Figure 67. ^{19}F NMR spectrum of **4l** ($CDCl_3$, 377 MHz)

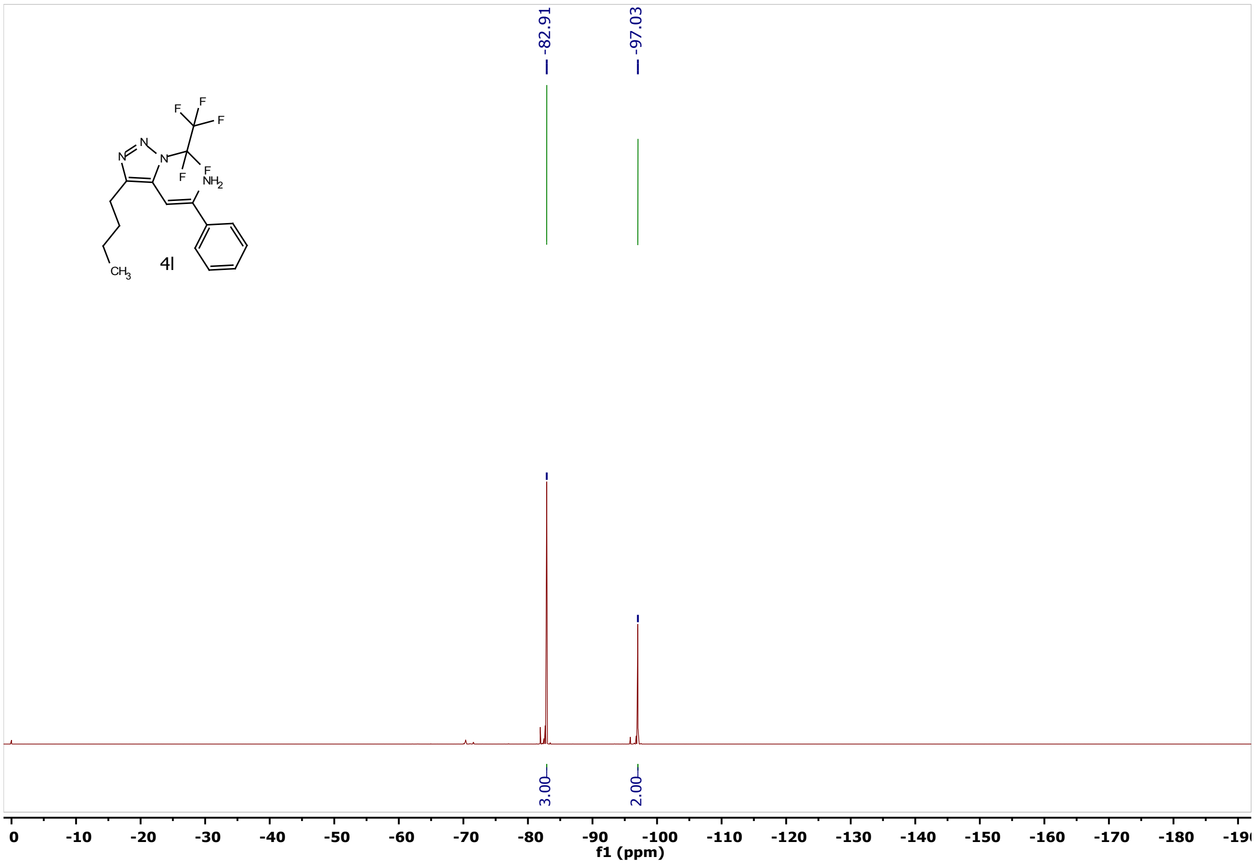

Figure 68. ^{1}H NMR spectrum of **4m** ($CDCl_3$, 400 MHz)

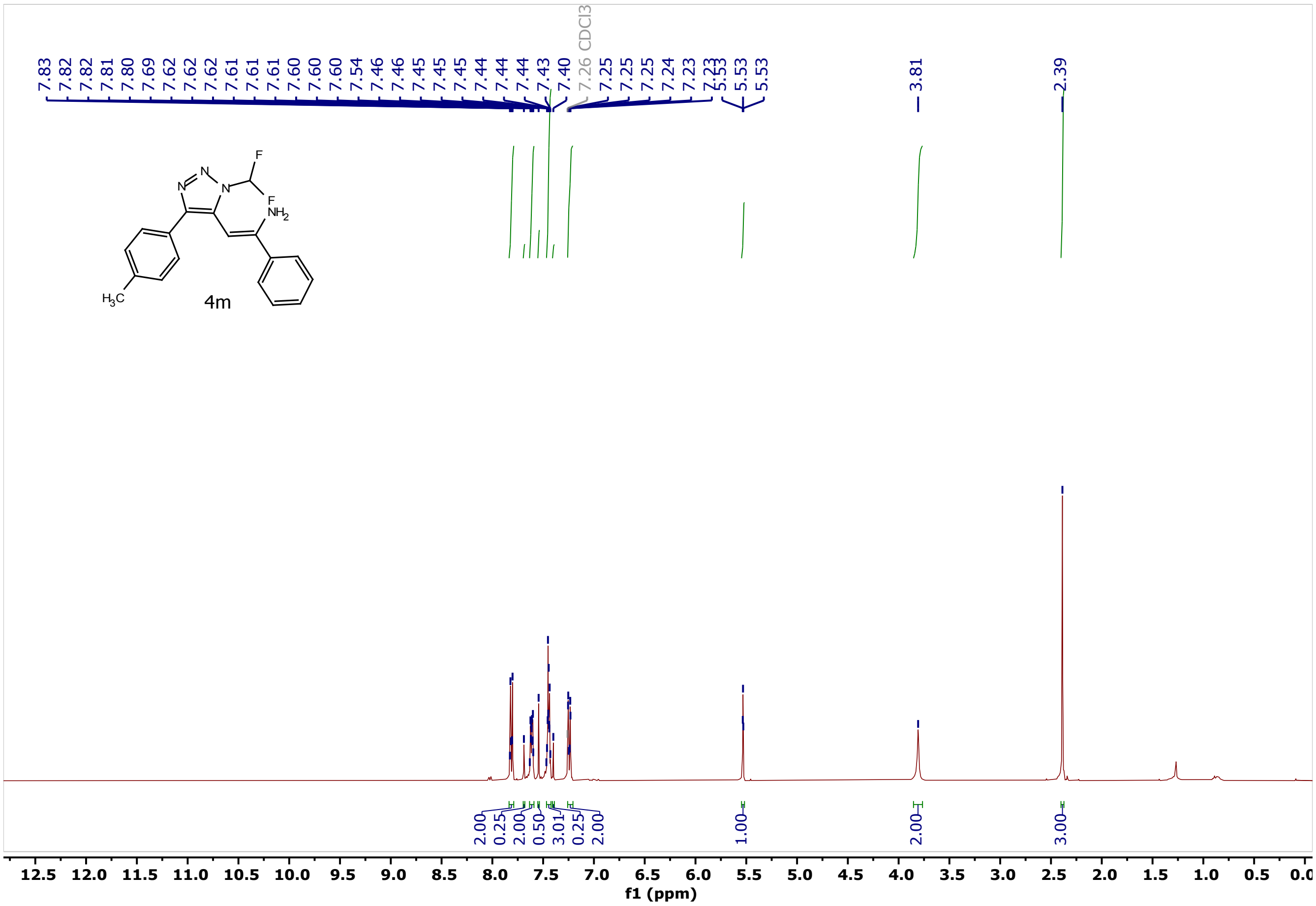

Figure 69. ^{13}C NMR spectrum of **4m** ($CDCl_3$, 101 MHz)

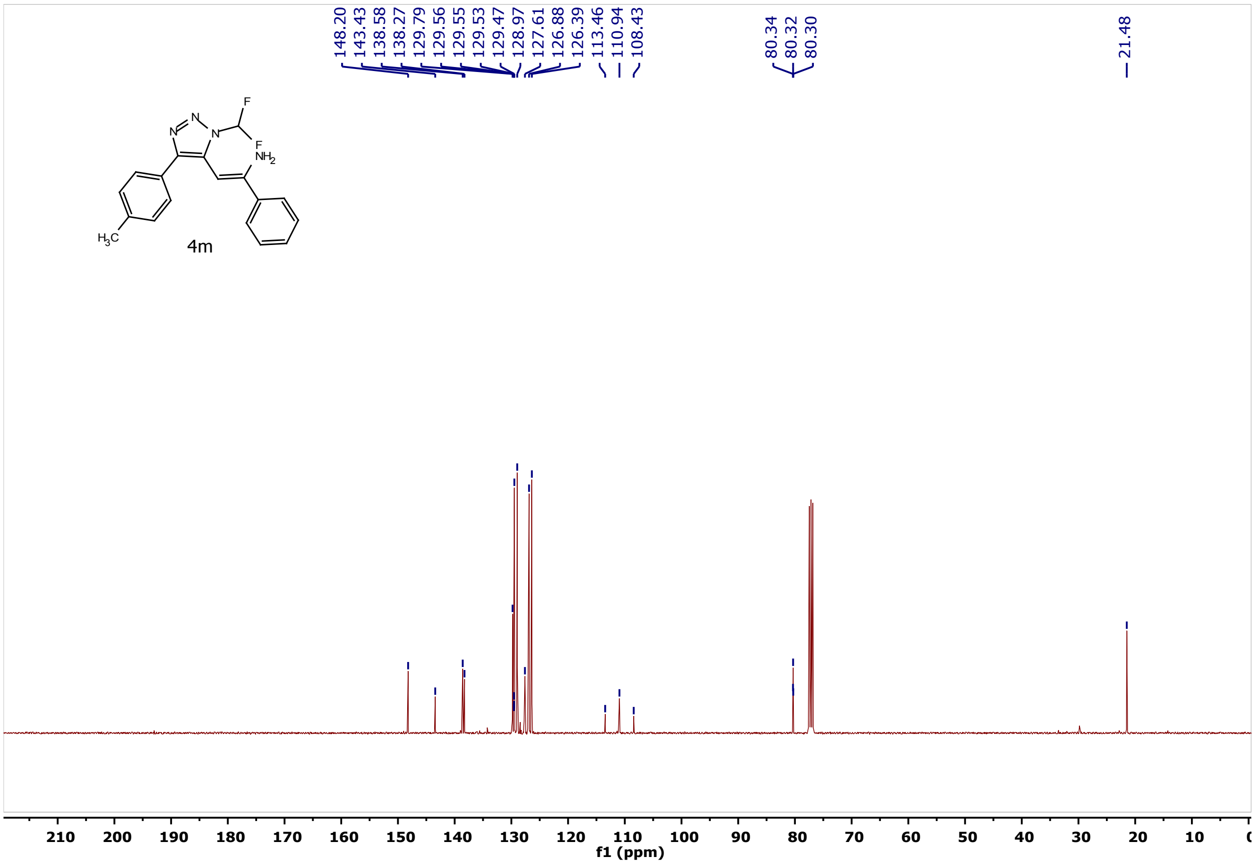

Figure 70. ^{19}F NMR spectrum of **4m** ($CDCl_3$, 377 MHz)

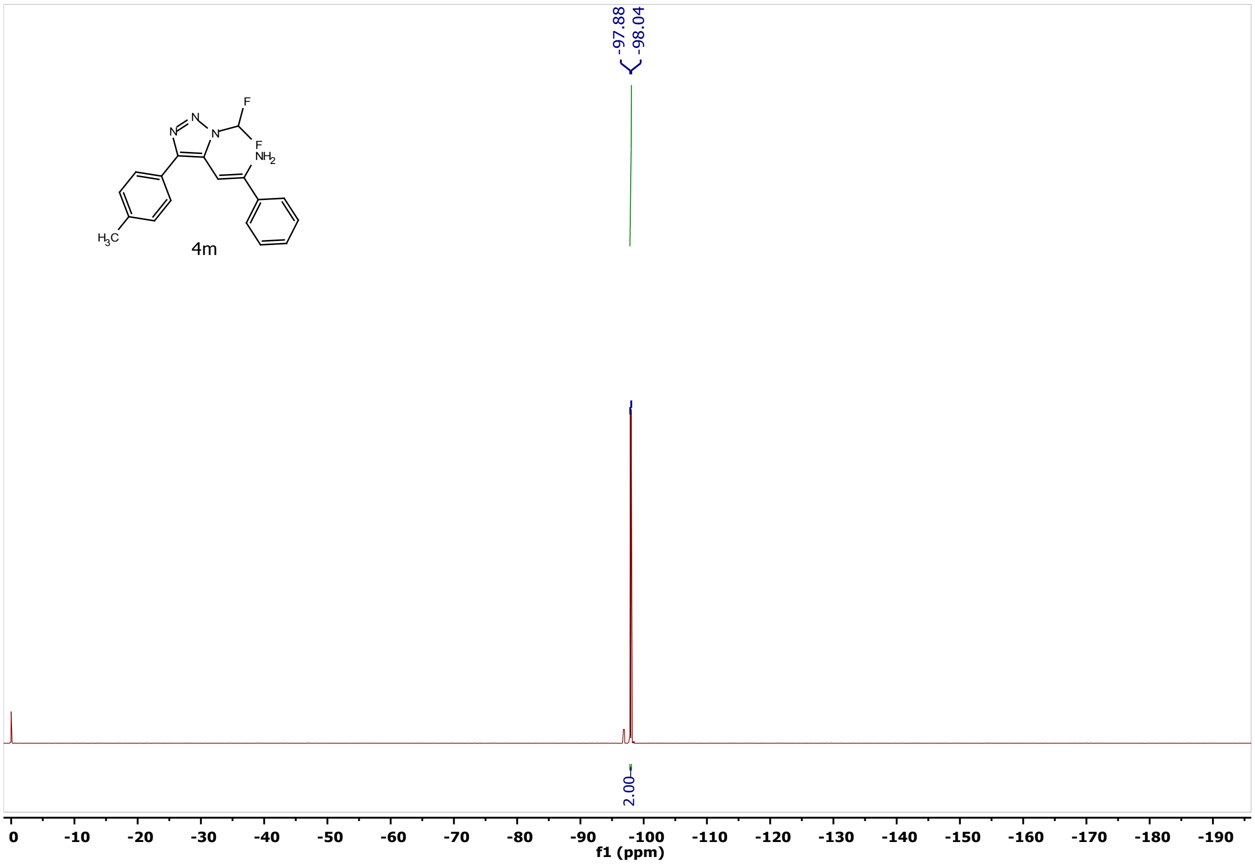

Figure 71. ^{1}H NMR spectrum of **4n** ($CDCl_3$, 400 MHz)

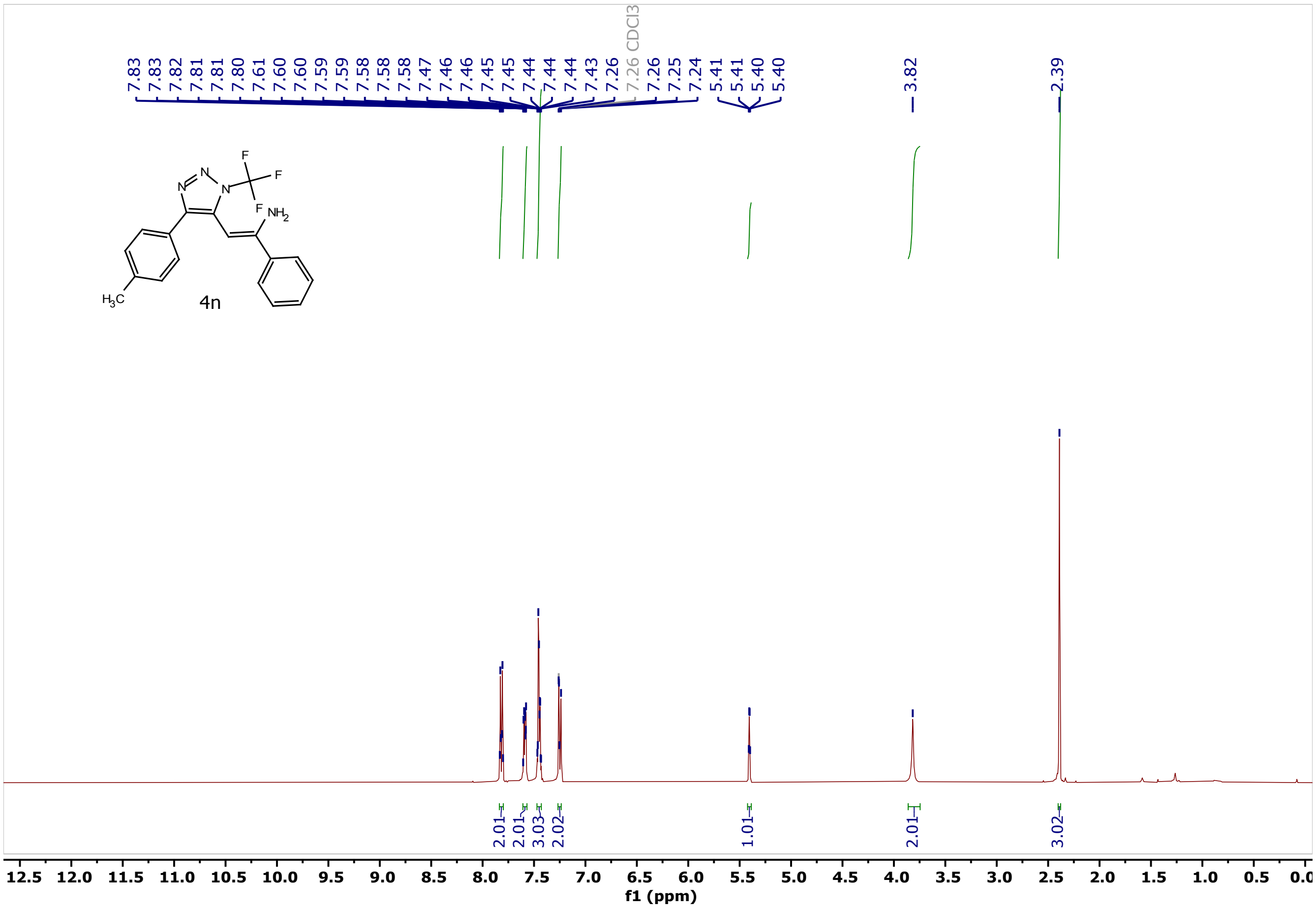

Figure 72. ^{13}C NMR spectrum of **4n** ($CDCl_3$, 101 MHz)

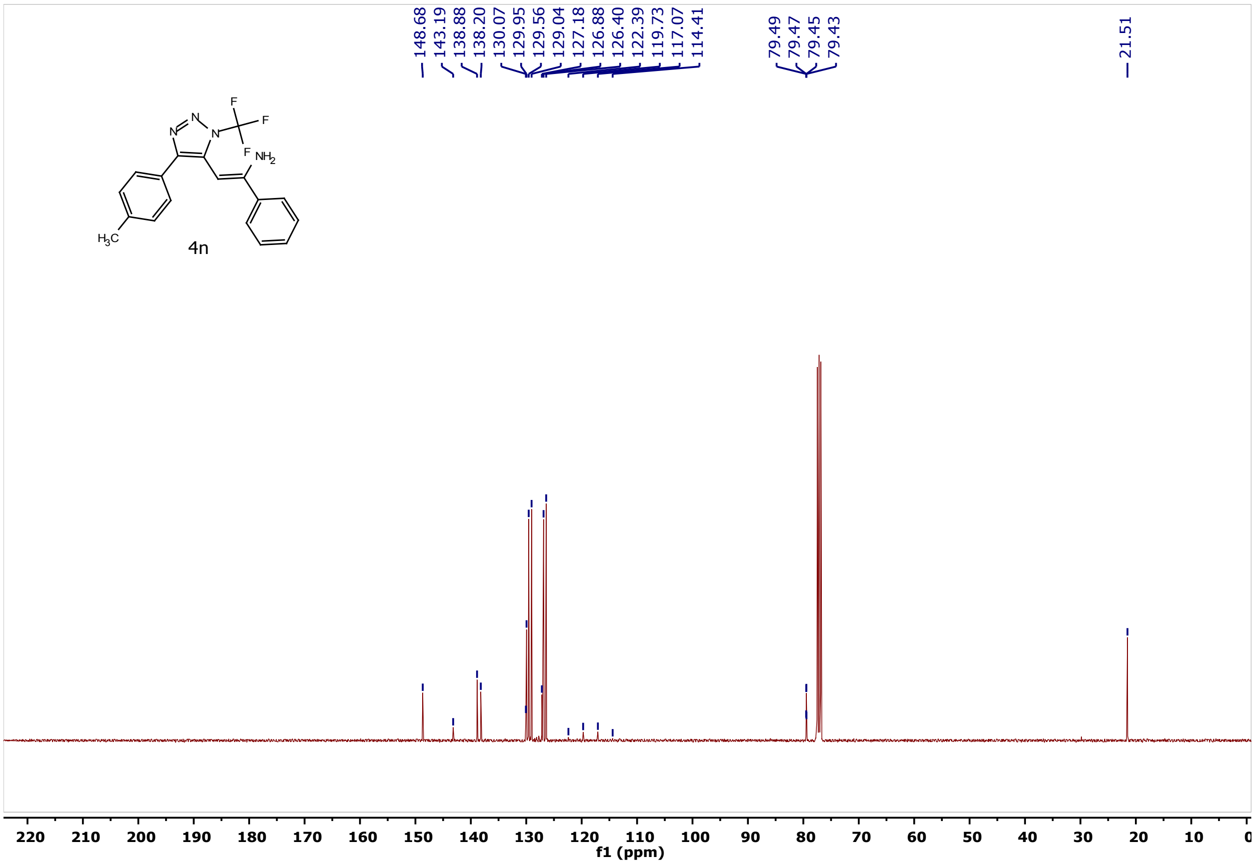

Figure 73. ^{19}F NMR spectrum of **4n** ($CDCl_3$, 377 MHz)

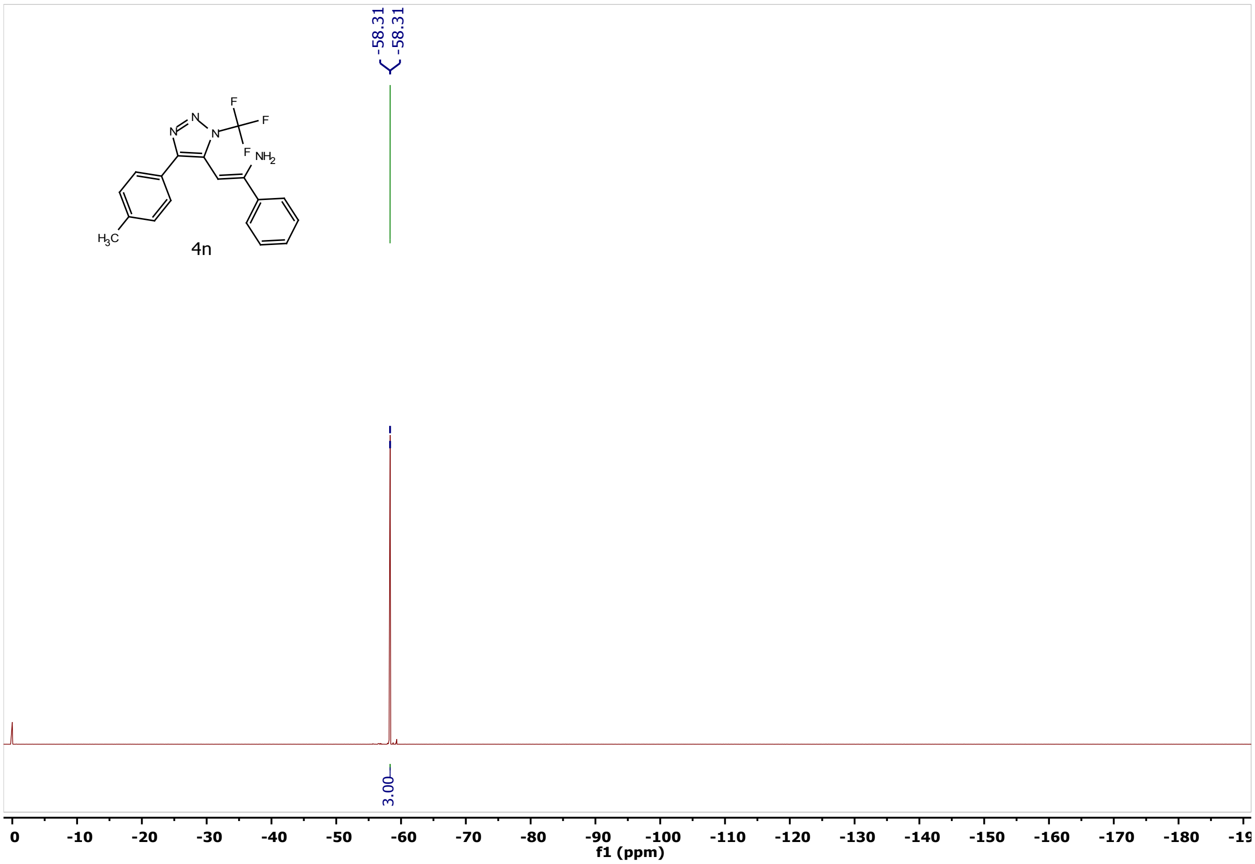

Figure 74. ^{1}H NMR spectrum of **4o** ($CDCl_3$, 400 MHz)

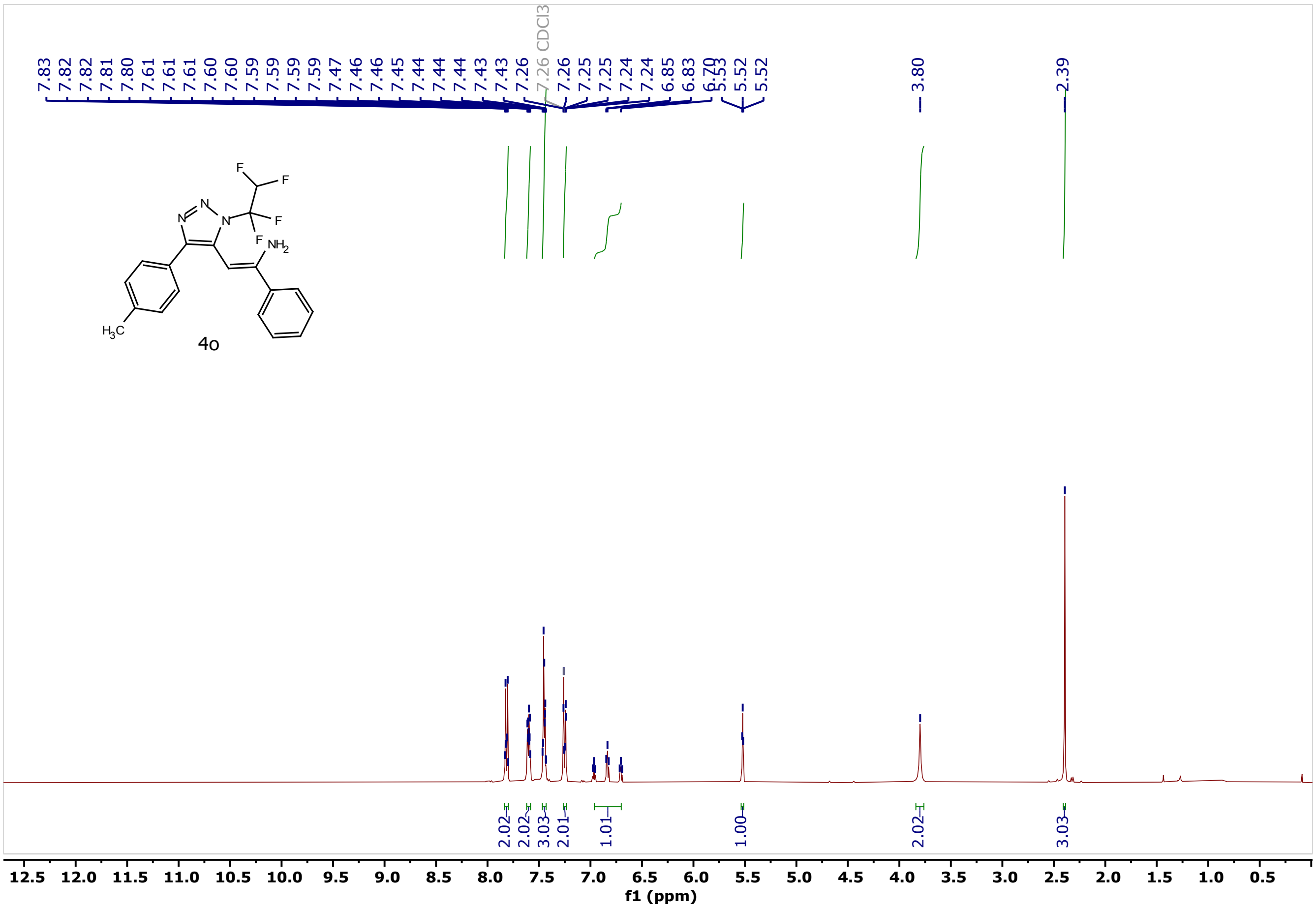

Figure 75. ^{13}C NMR spectrum of **4o** ($CDCl_3$, 101 MHz)

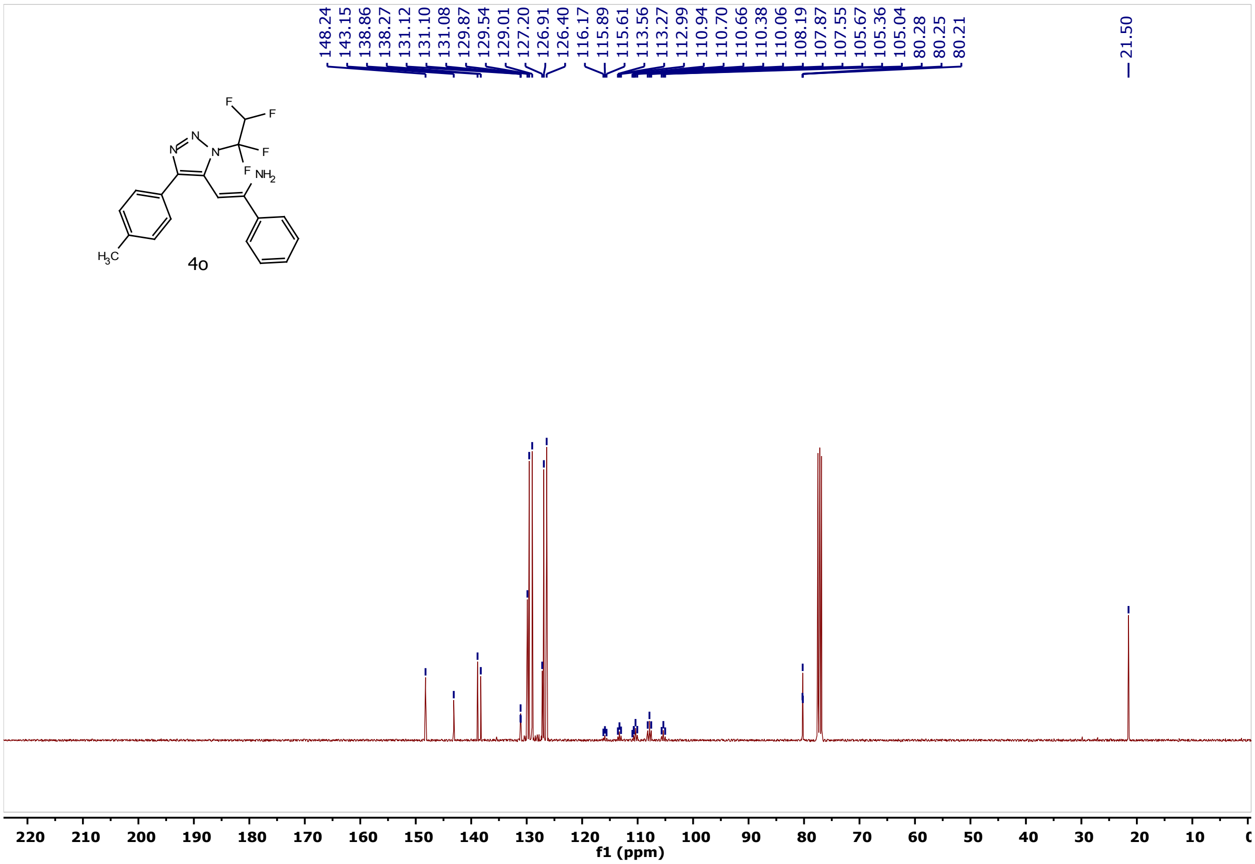

Figure 76. ^{19}F NMR spectrum of **4o** ($CDCl_3$, 377 MHz)

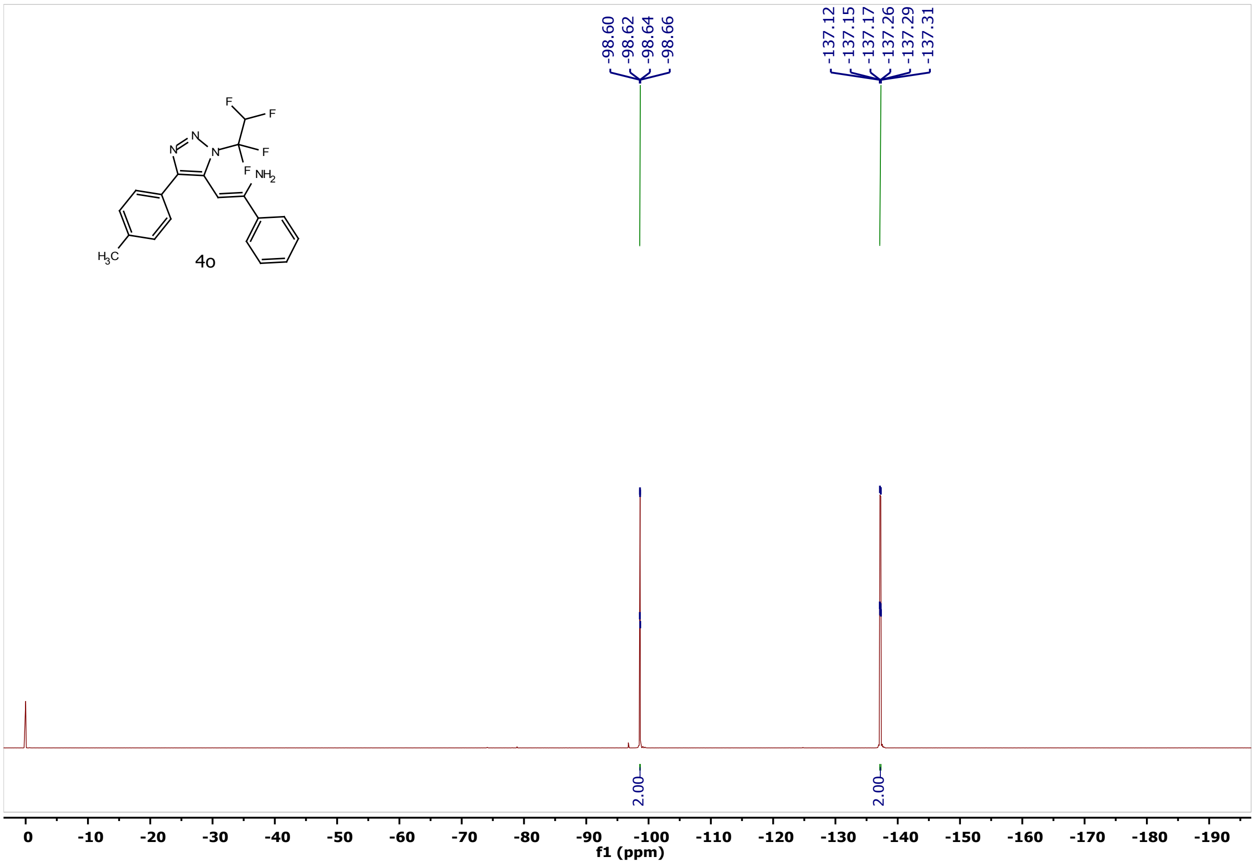

Figure 77. ^{1}H NMR spectrum of **4p** (Acetone-d_6, 400 MHz)

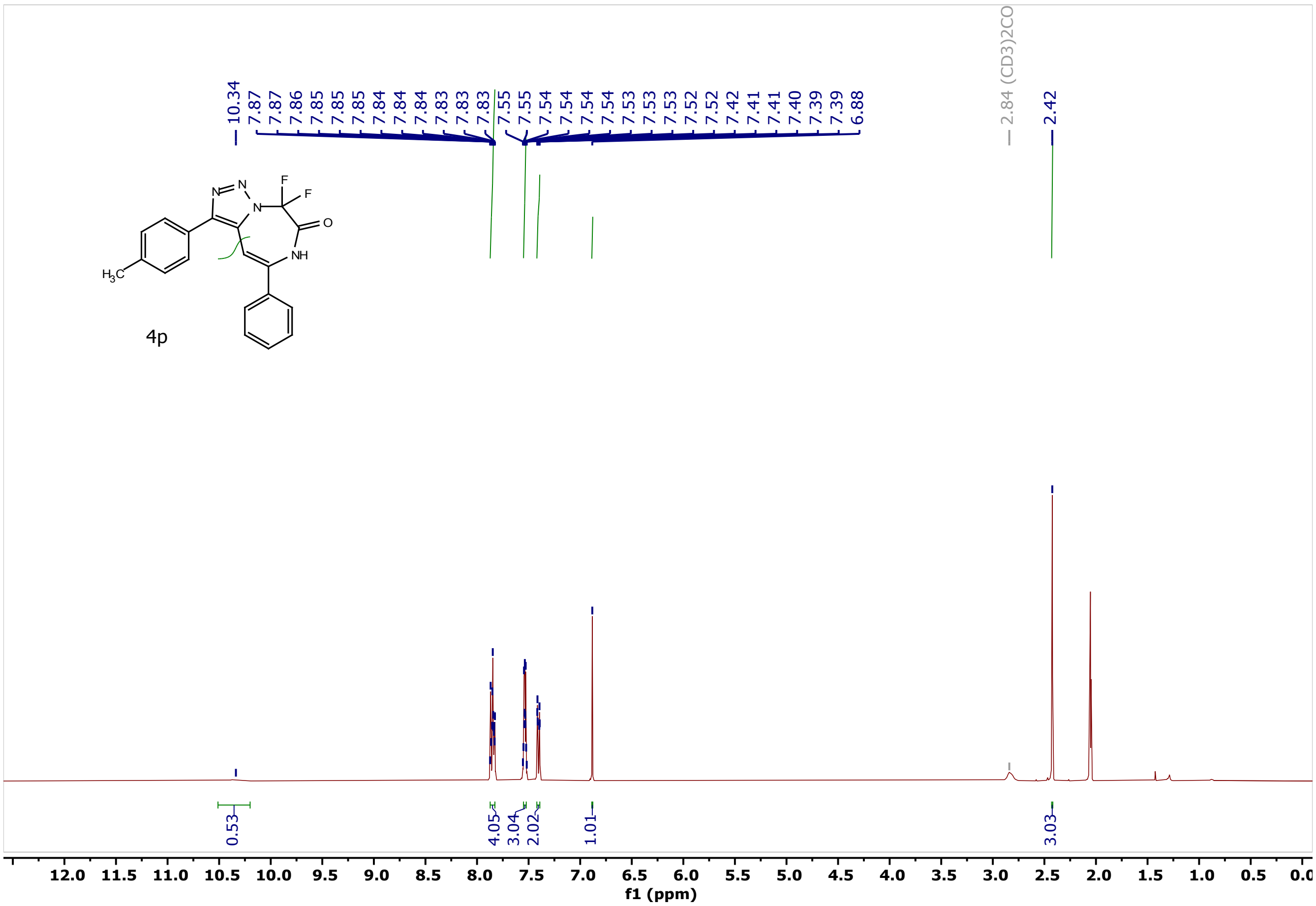

Figure 78. ^{13}C NMR spectrum of **4p** (Acetone-d$_6$, 101 MHz)

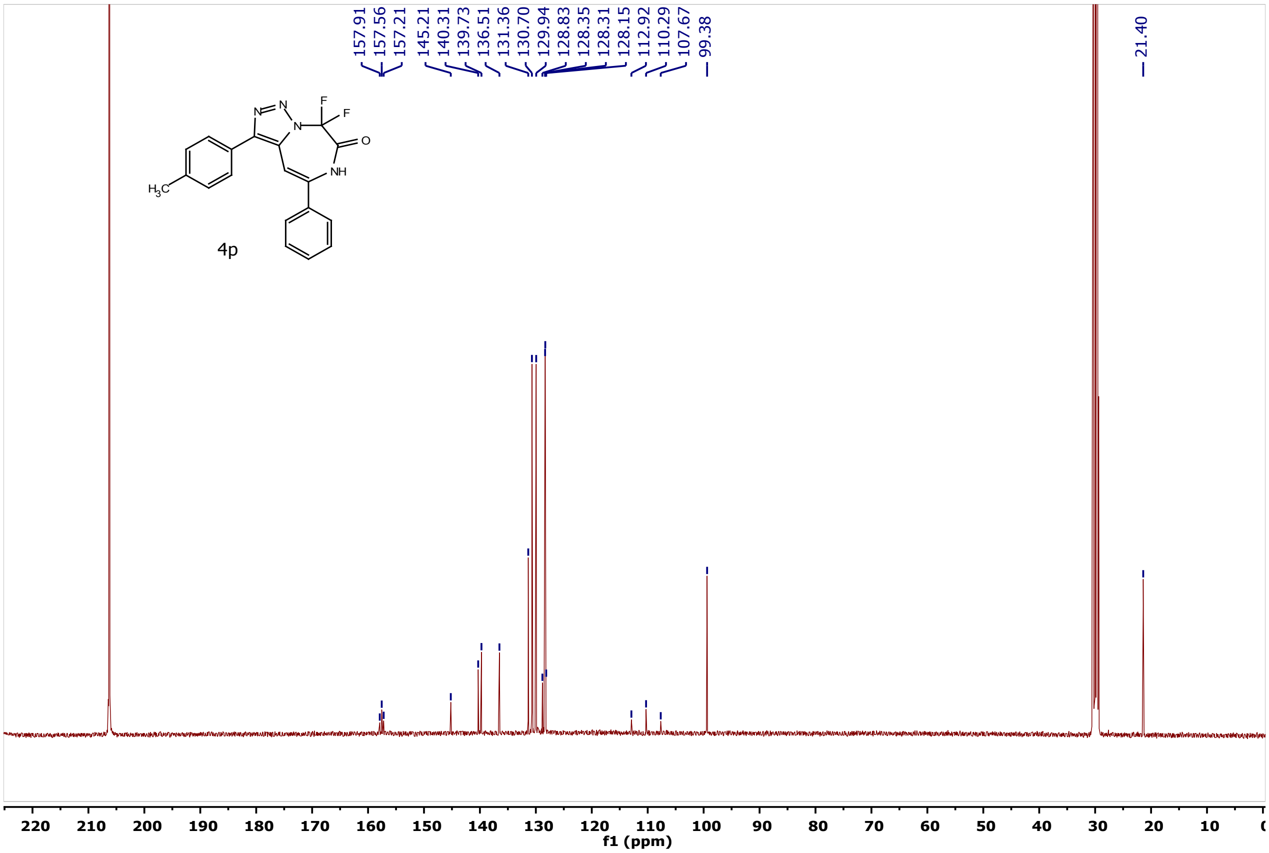

Figure 79. ^{19}F NMR spectrum of **4p** (Acetone-d_6, 377 MHz)

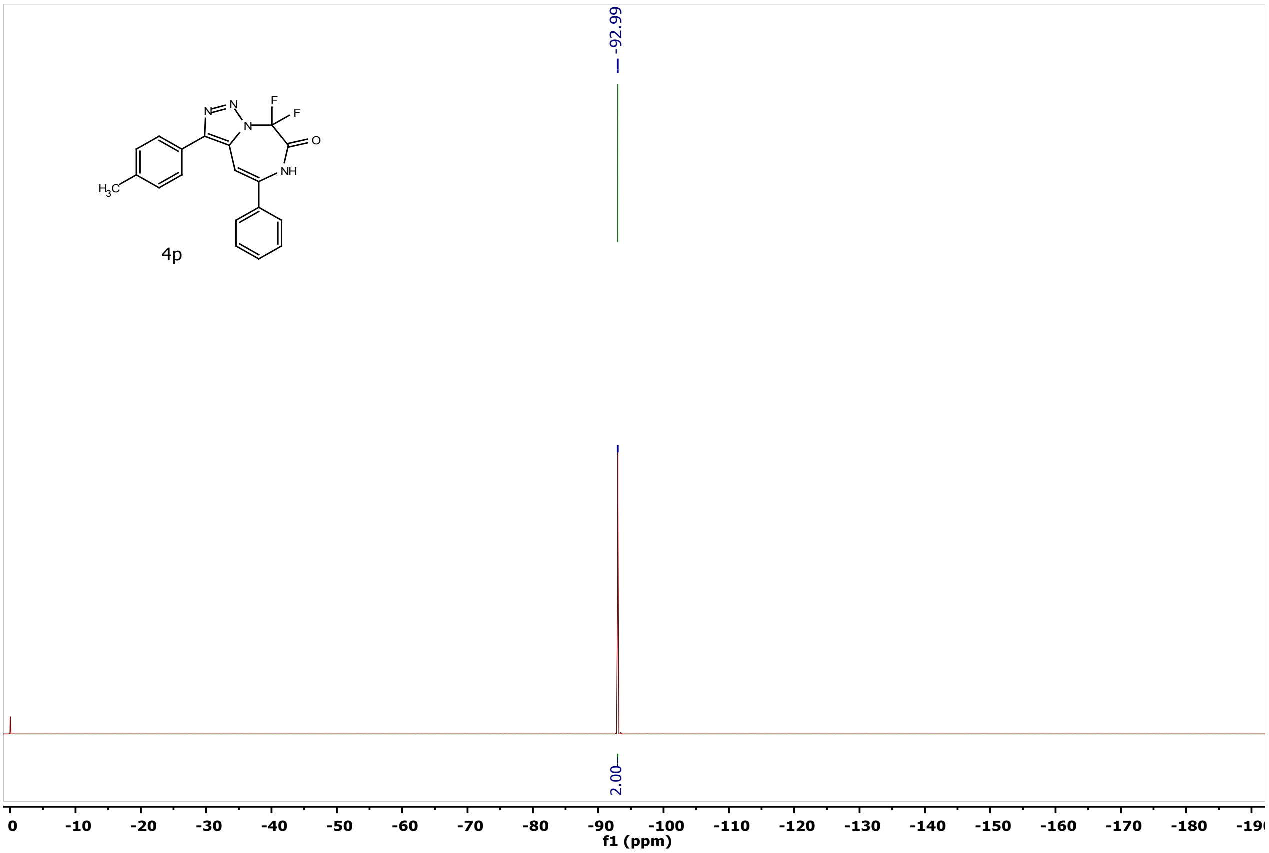

Figure 80. ^{1}H NMR spectrum of **4q** (DMSO-d_6, 400 MHz)

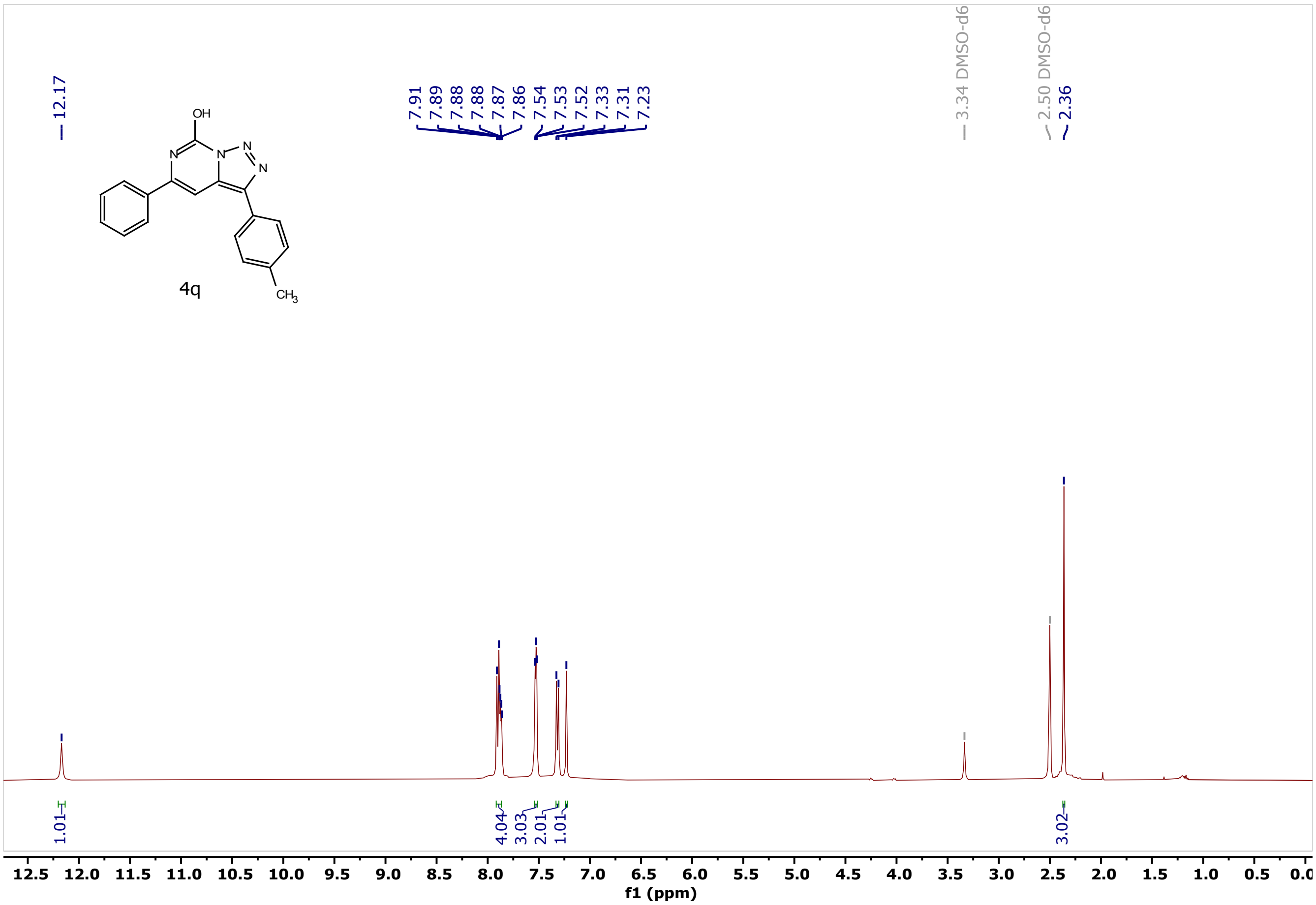

Figure 81. ^{13}C NMR spectrum of **4q** (DMSO-d_6, 101 MHz)

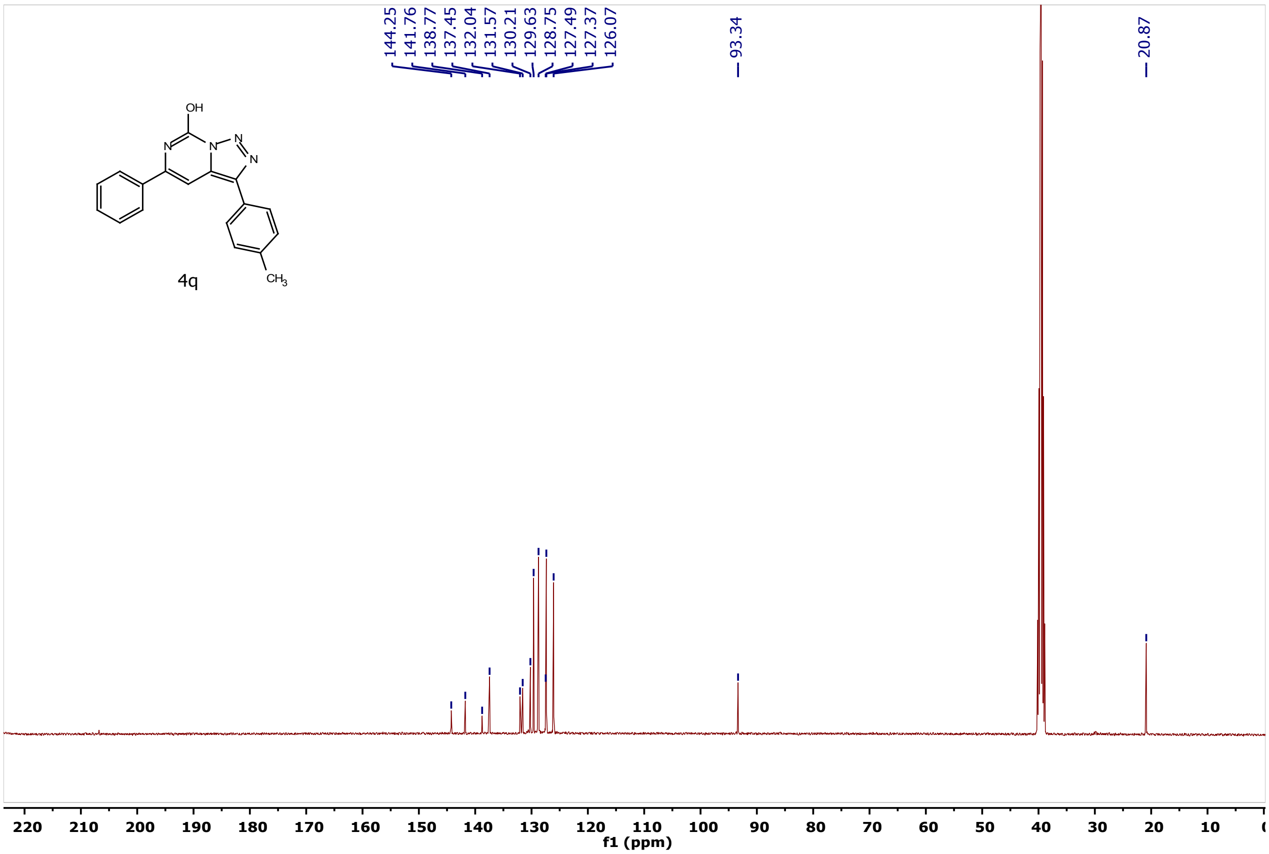

Figure 82. ^{1}H NMR spectrum of **4r** ($CDCl_3$, 400 MHz)

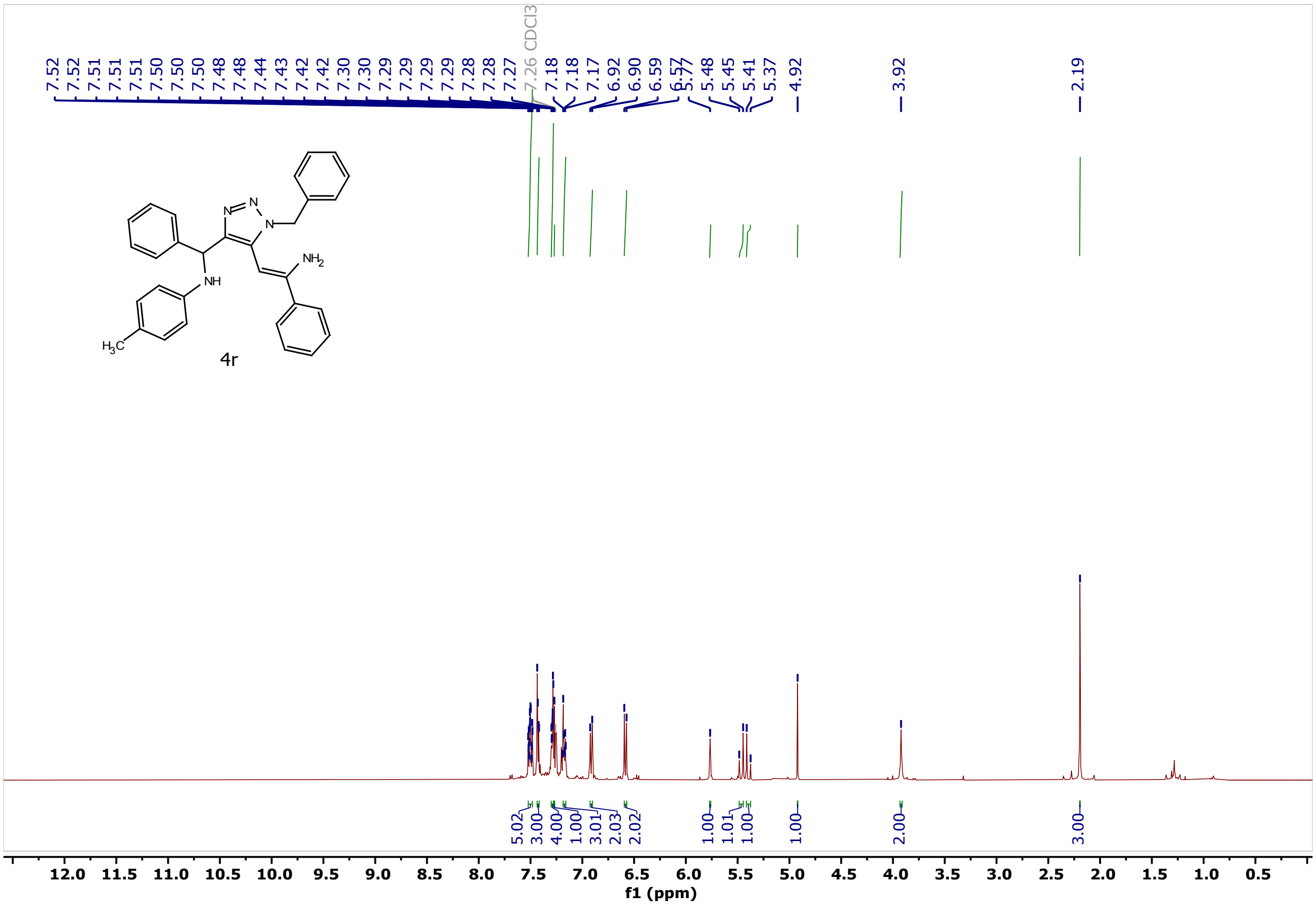

Figure 83. ^{13}C NMR spectrum of **4r** ($CDCl_3$, 101 MHz)

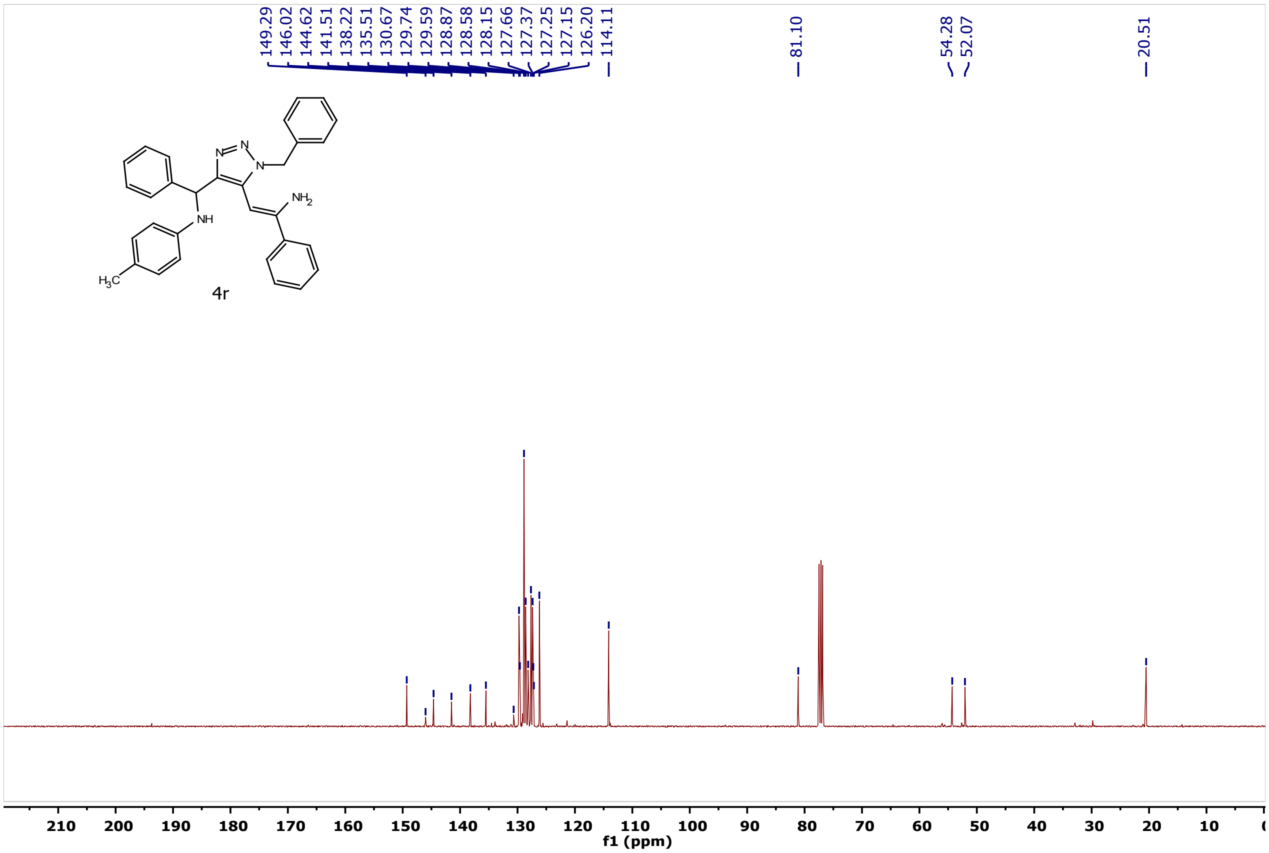

Figure 84. ^{13}C APT NMR spectrum of **4r** ($CDCl_3$, 101 MHz)

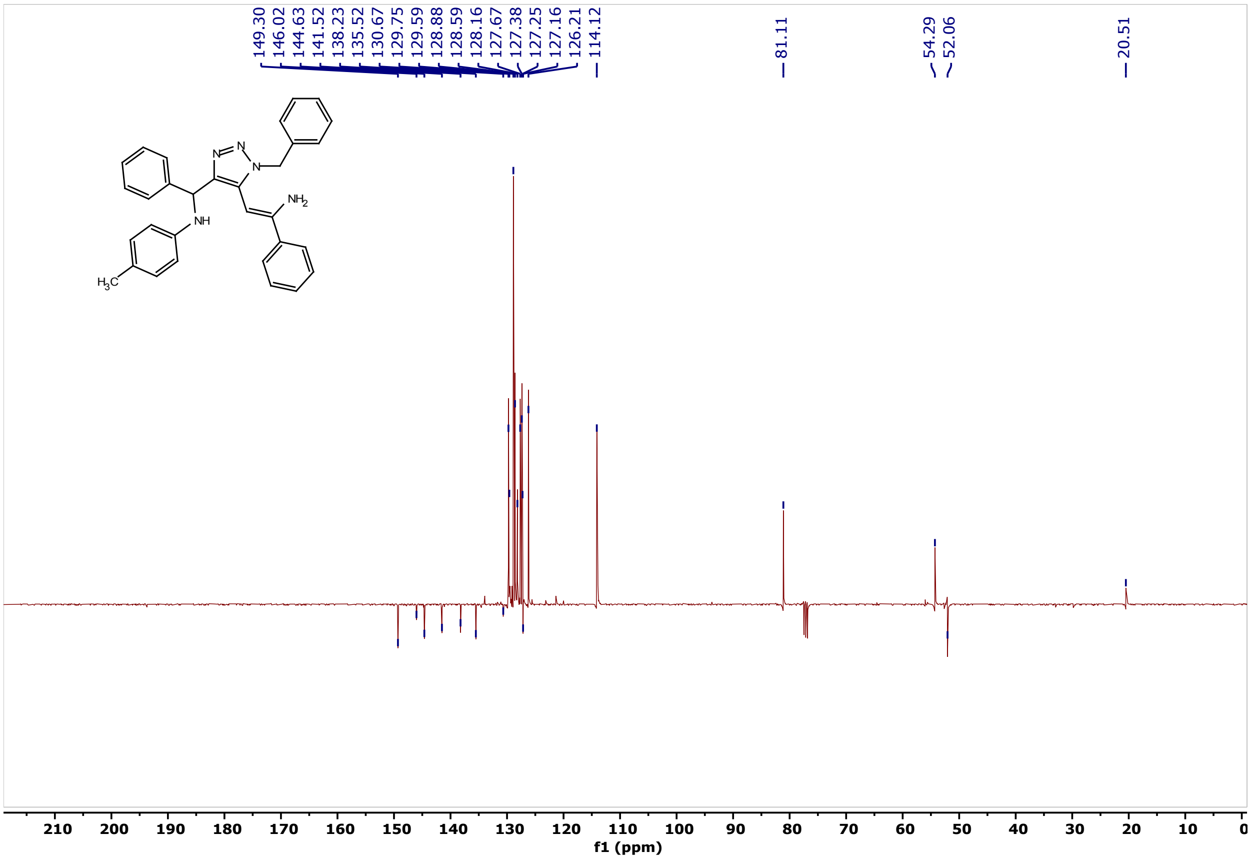

Figure 85. ^{1}H NMR spectrum of **4s** ($CDCl_3$, 400 MHz)

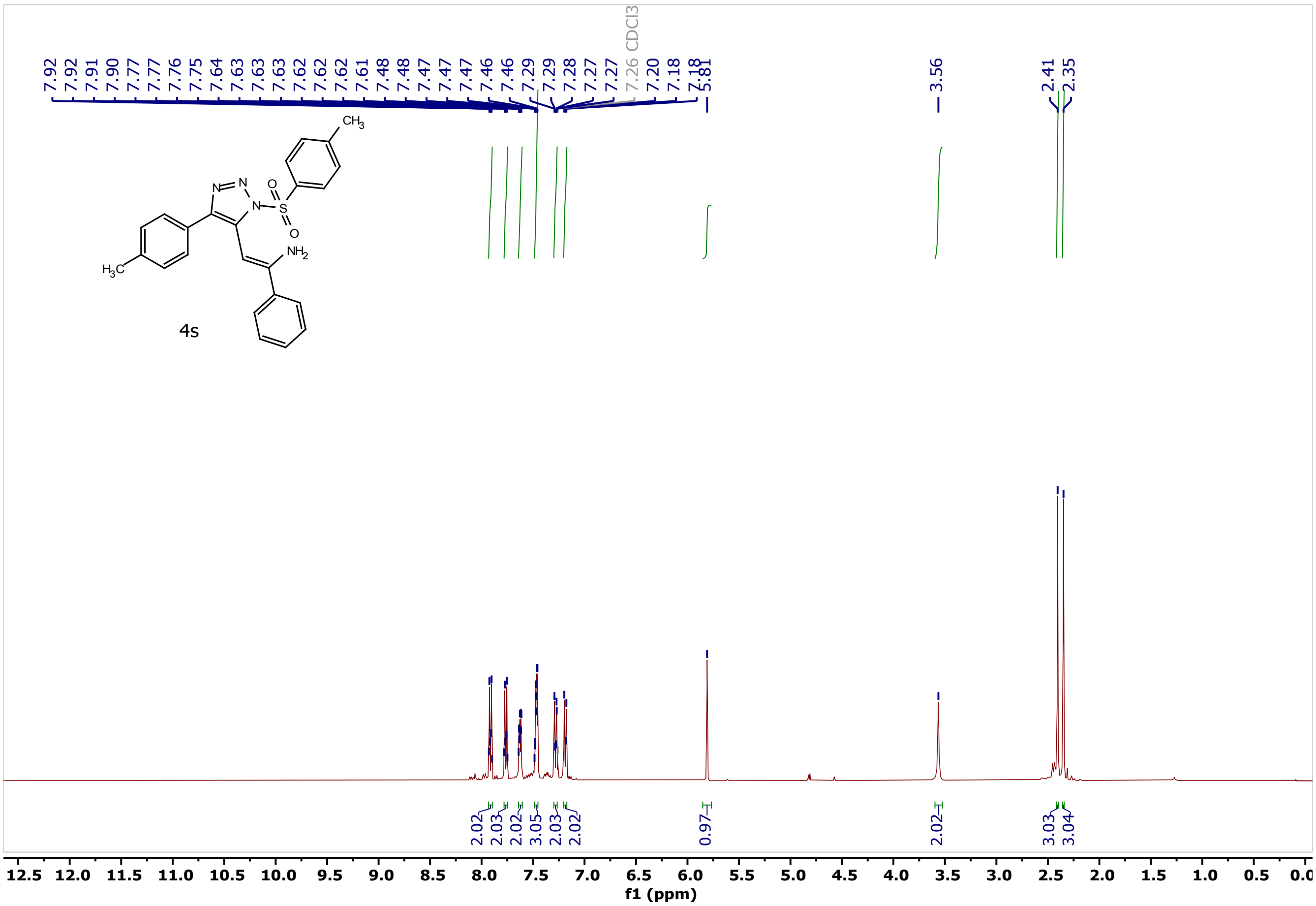

Figure 86. ^{13}C APT NMR spectrum of **4s** ($CDCl_3$, 101 MHz)

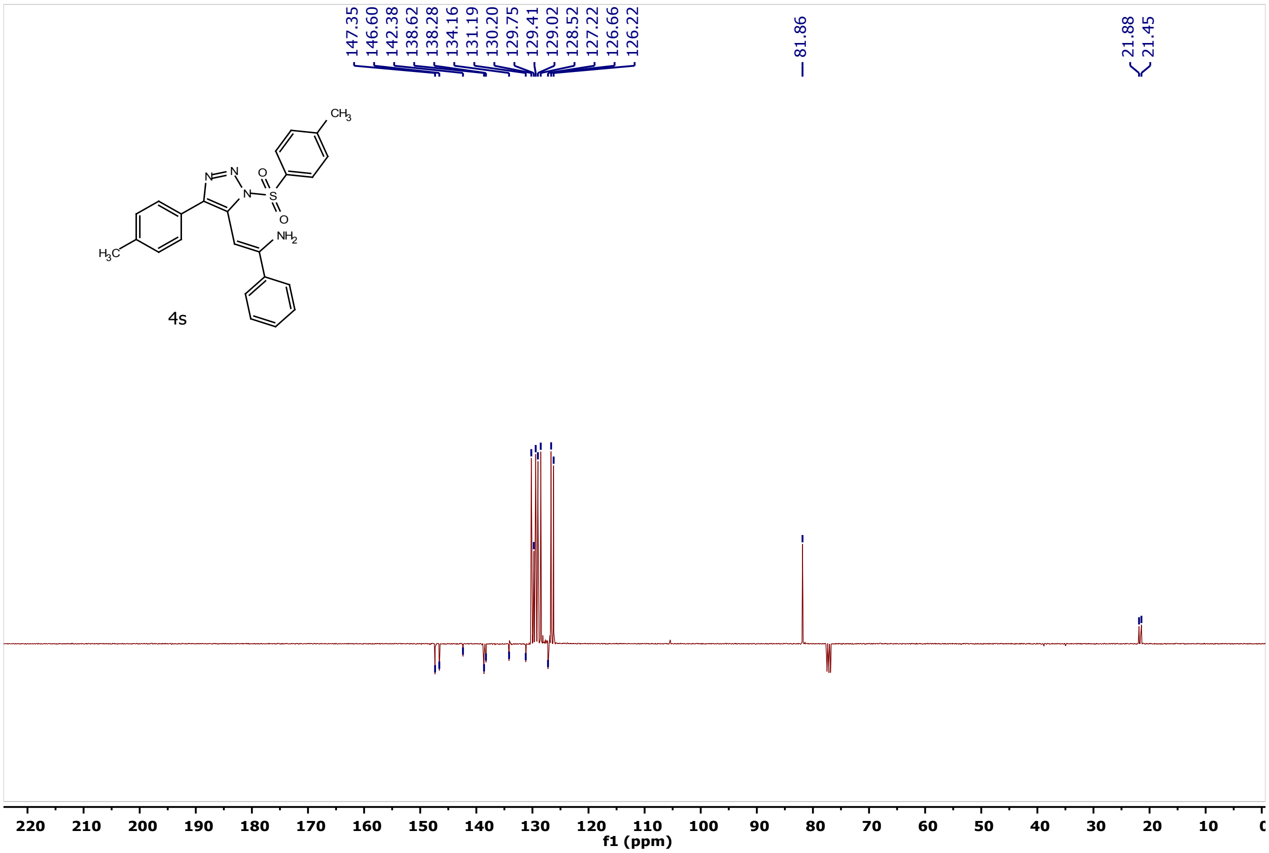

Figure 87. ^{1}H NMR spectrum of **4t** ($CDCl_3$, 400 MHz)

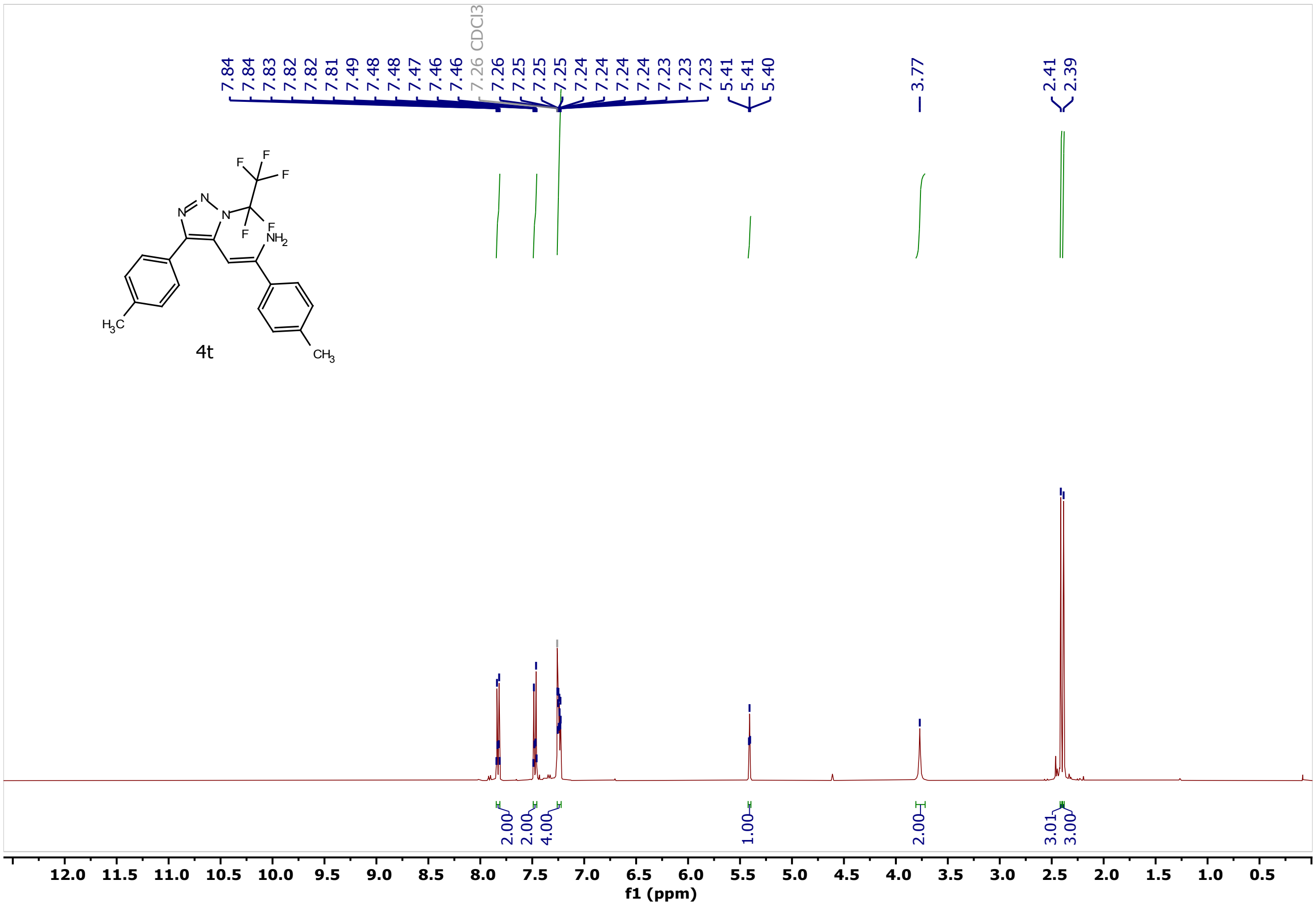

Figure 88. ^{13}C NMR spectrum of **4t** ($CDCl_3$, 101 MHz)

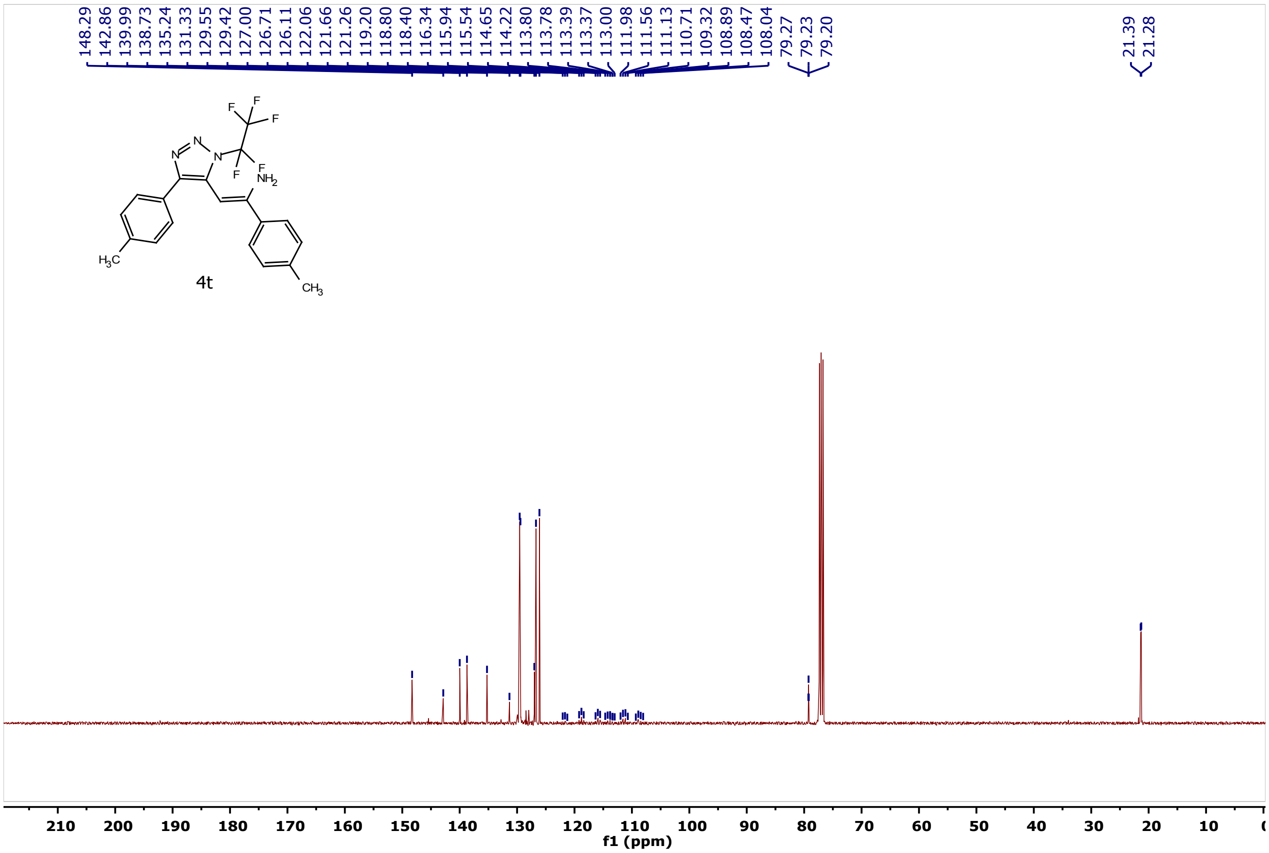

Figure 89. ^{19}F NMR spectrum of **4t** ($CDCl_3$, 377 MHz)

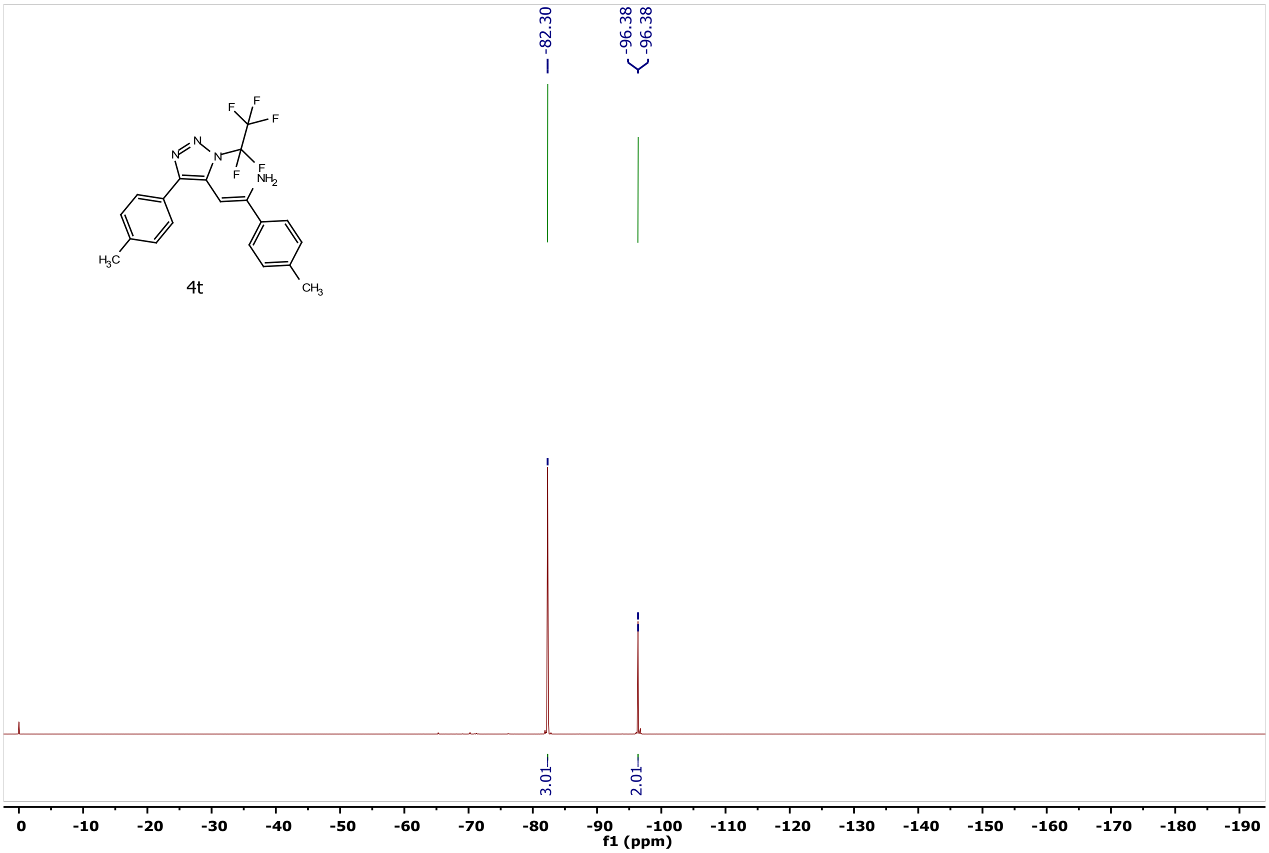

Figure 90. ^{1}H NMR spectrum of **4u** ($CDCl_3$, 400 MHz)

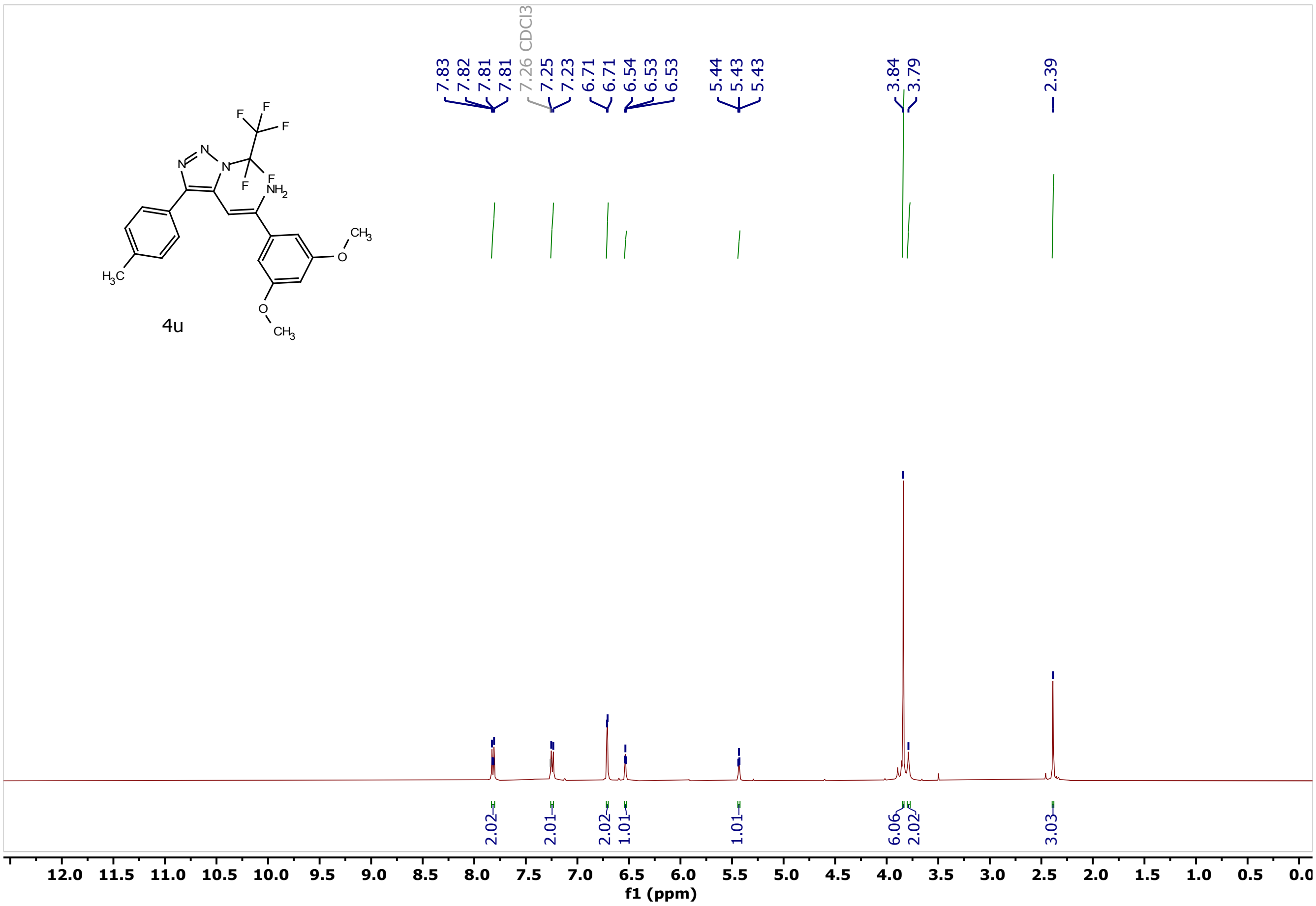

Figure 91. ^{13}C NMR spectrum of **4u** ($CDCl_3$, 101 MHz)

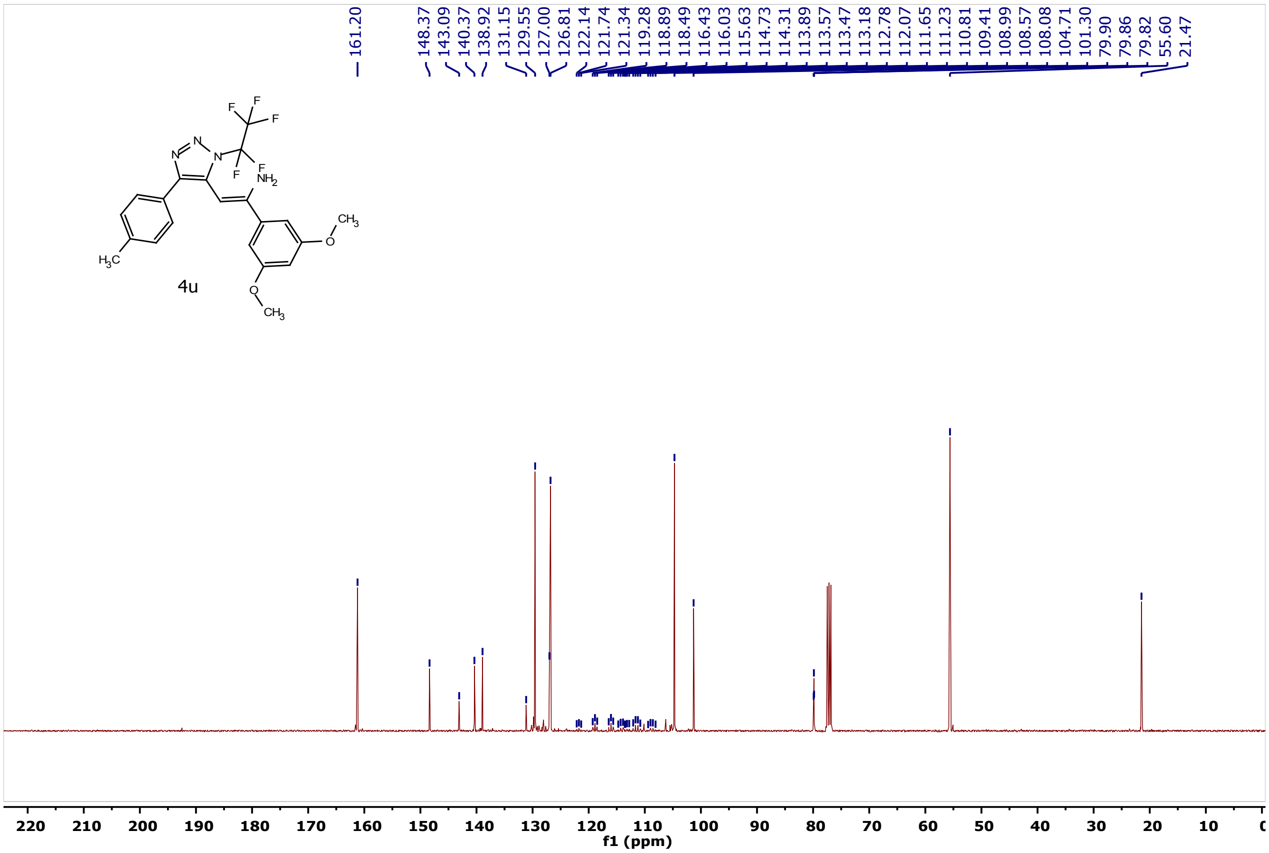

Figure 92. ^{19}F NMR spectrum of **4u** ($CDCl_3$, 377 MHz)

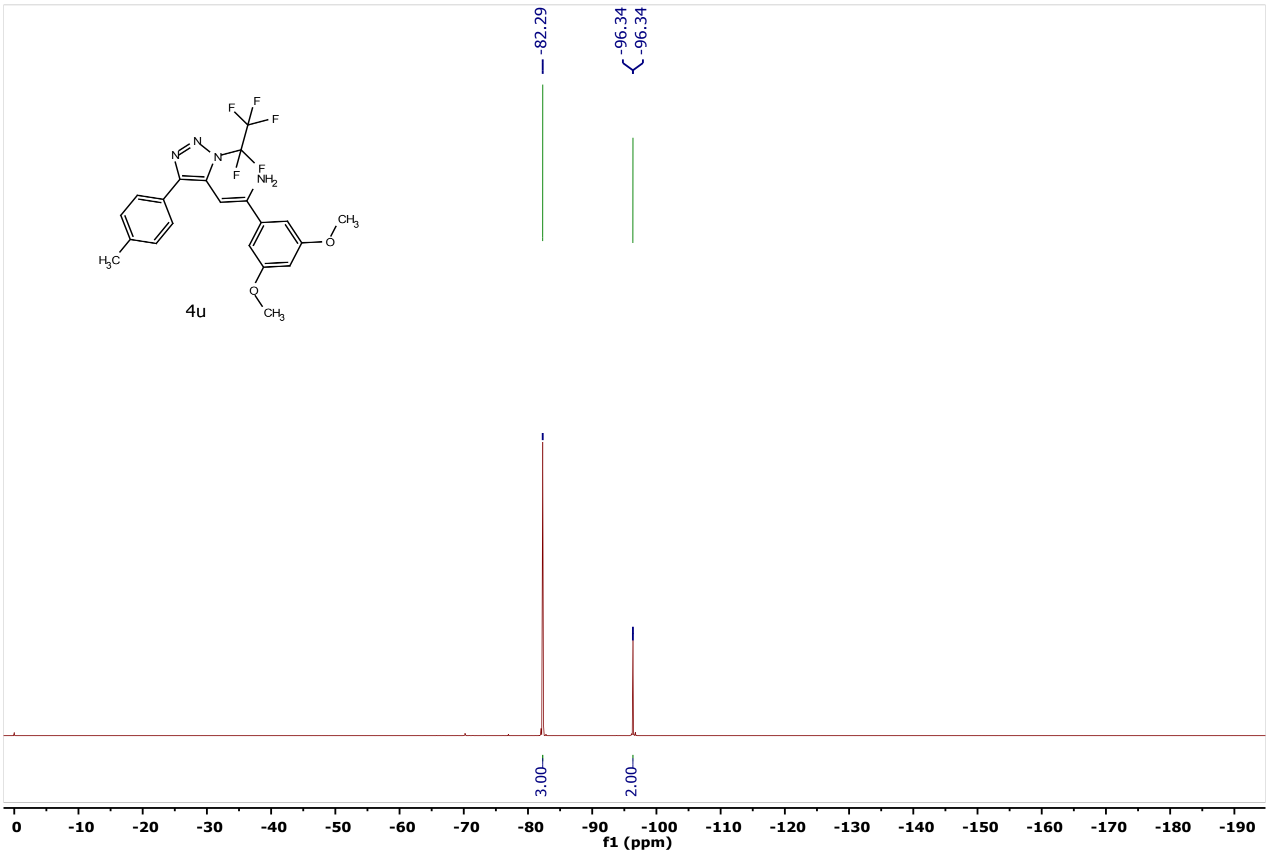

Figure 93. ^1H NMR spectrum of **4v** ($CDCl_3$, 400 MHz)

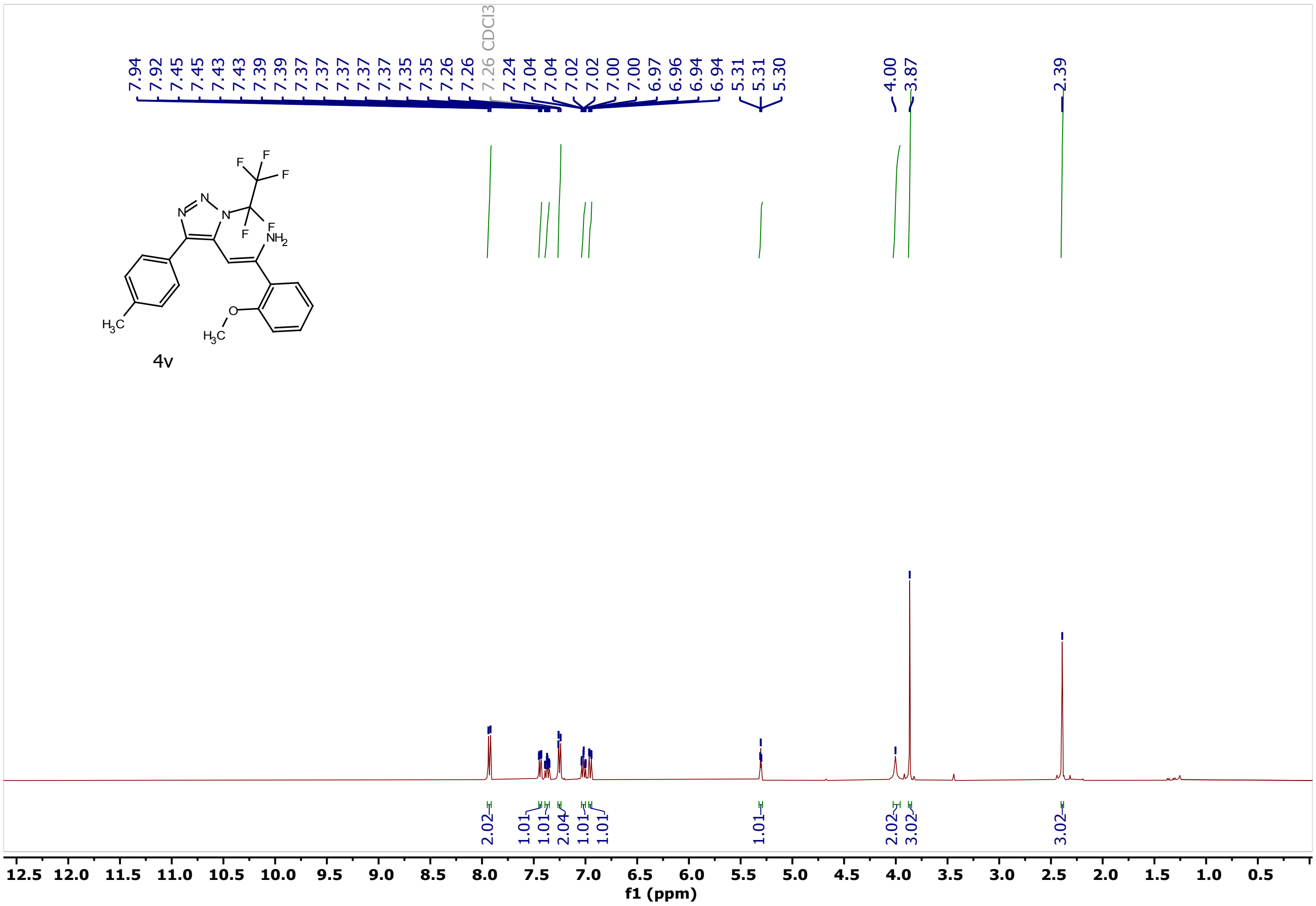

Figure 94. ^{13}C NMR spectrum of **4v** ($CDCl_3$, 101 MHz)

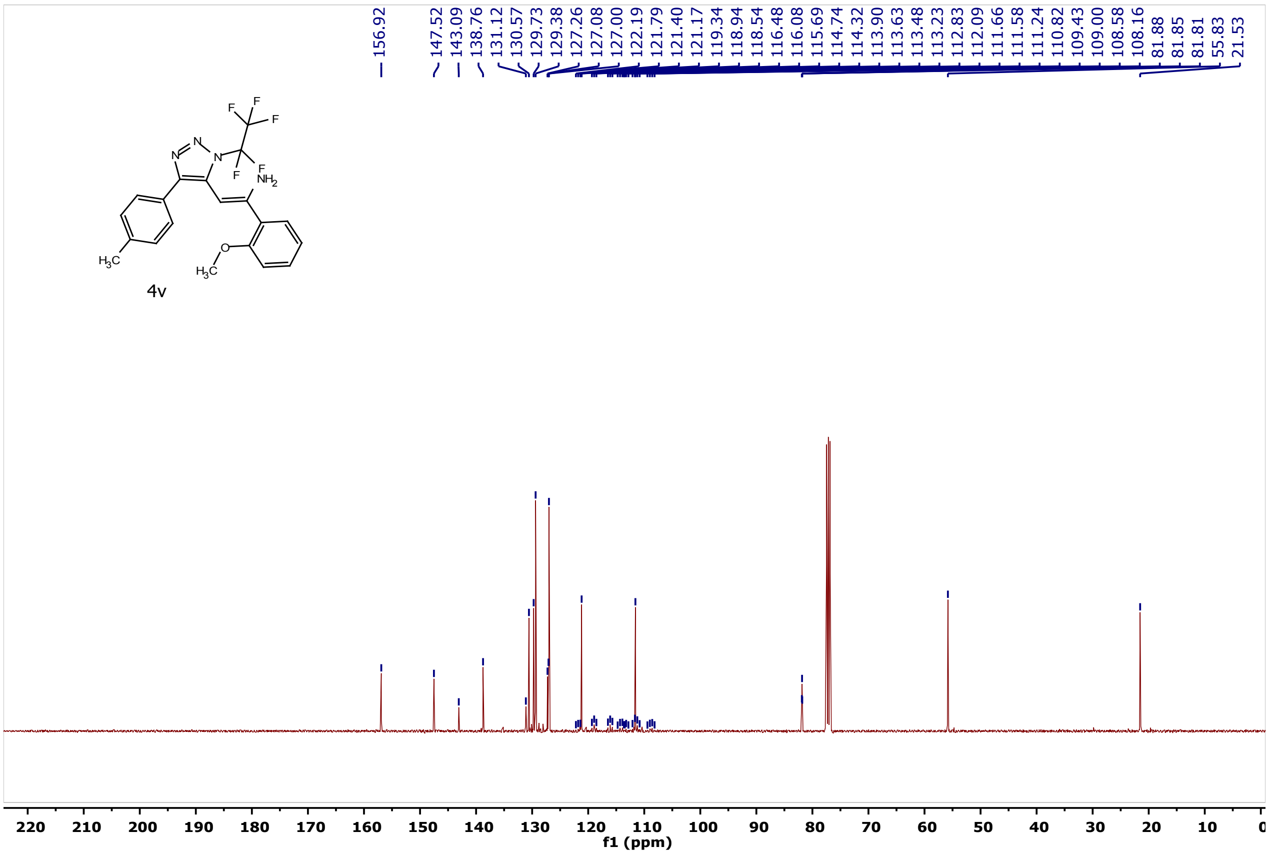

Figure 95. ^{19}F NMR spectrum of **4v** ($CDCl_3$, 377 MHz)

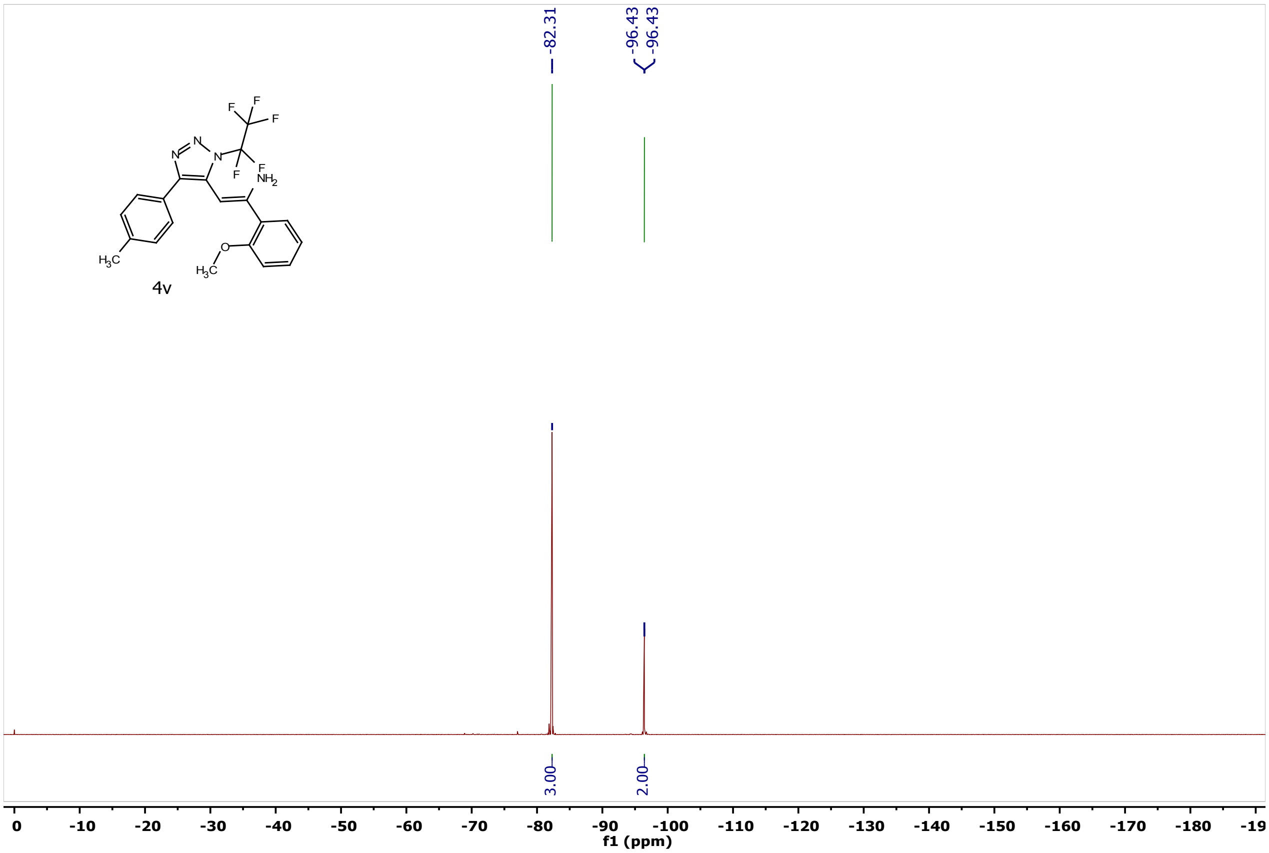

Figure 96. ^{1}H NMR spectrum of **4w** ($CDCl_3$, 400 MHz)

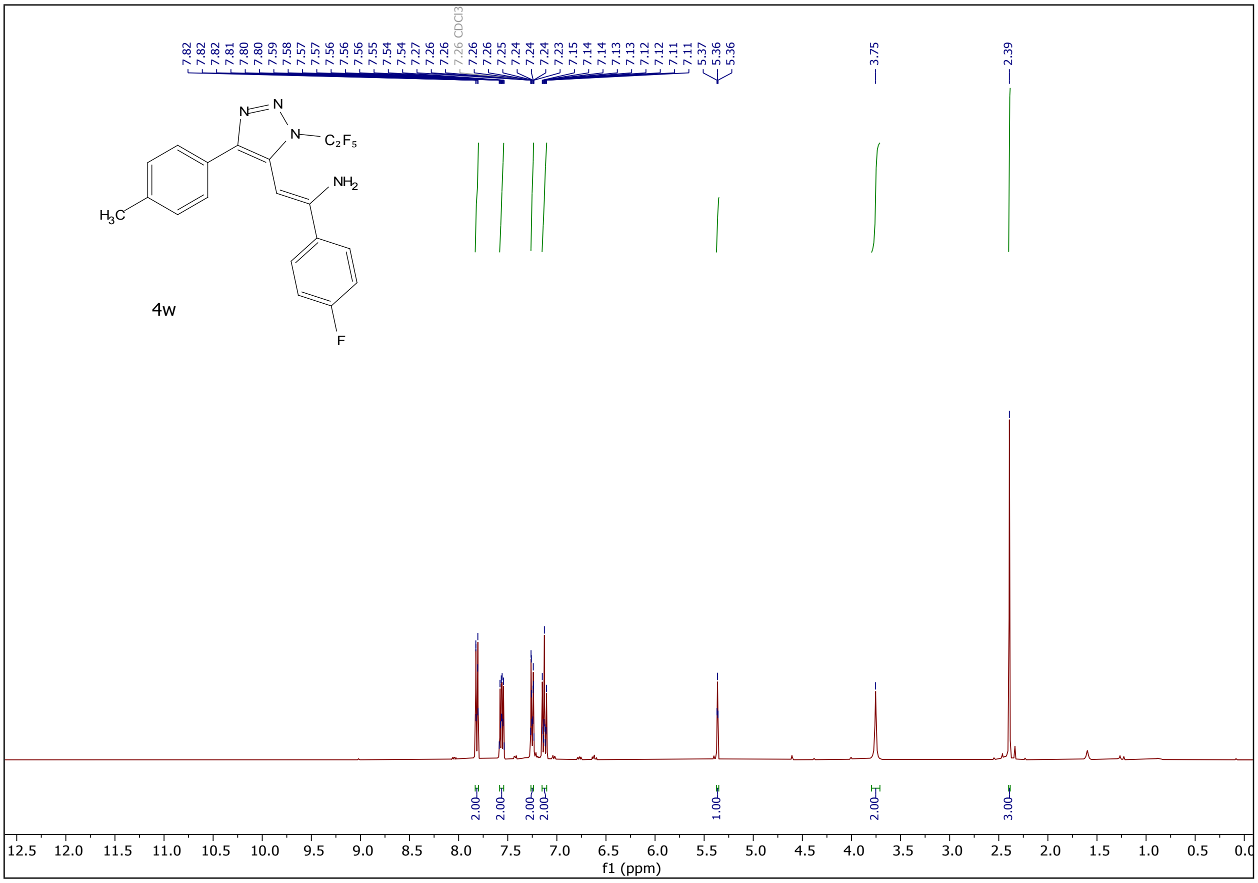

Figure 97. ^{13}C NMR spectrum of **4w** ($CDCl_3$, 101 MHz)

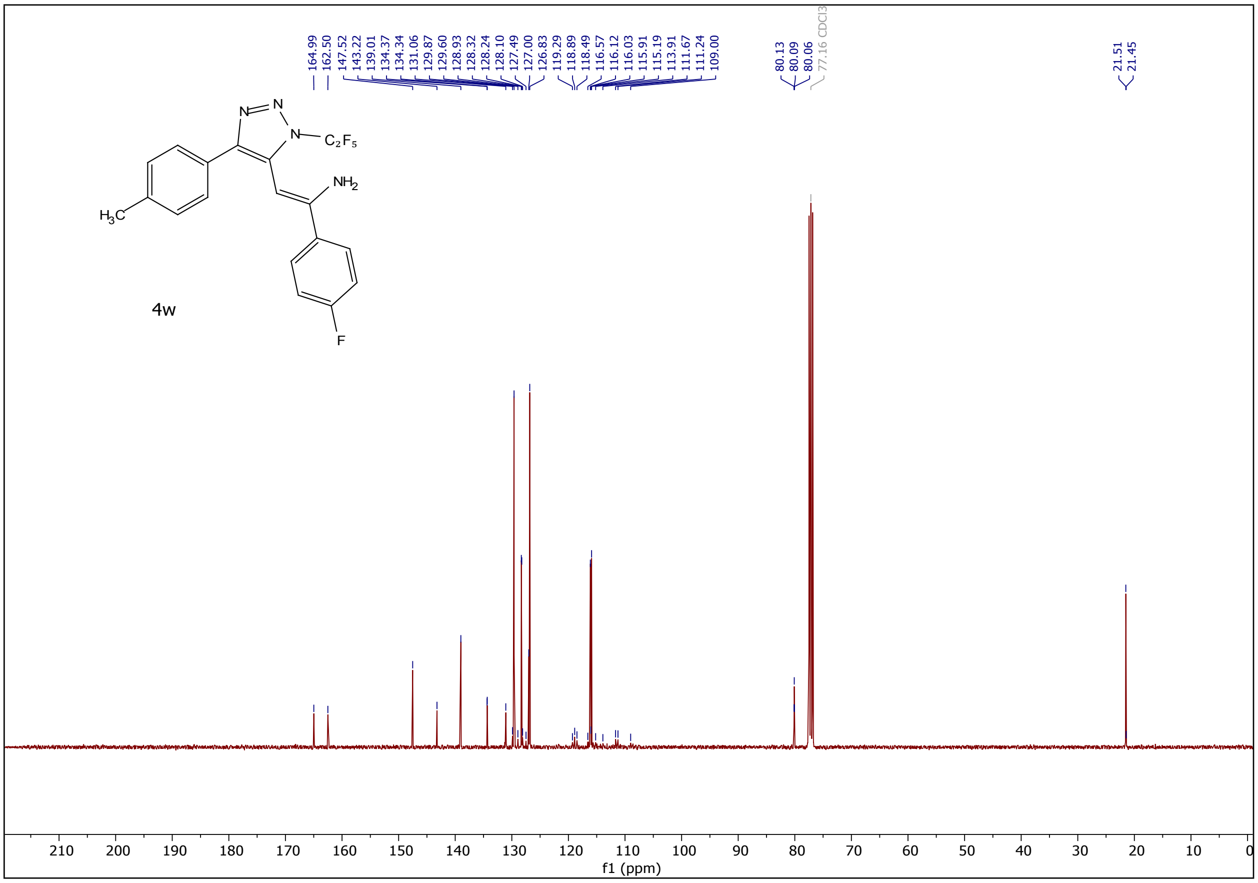

Figure 98. ^{19}F NMR spectrum of **4w** ($CDCl_3$, 377 MHz)

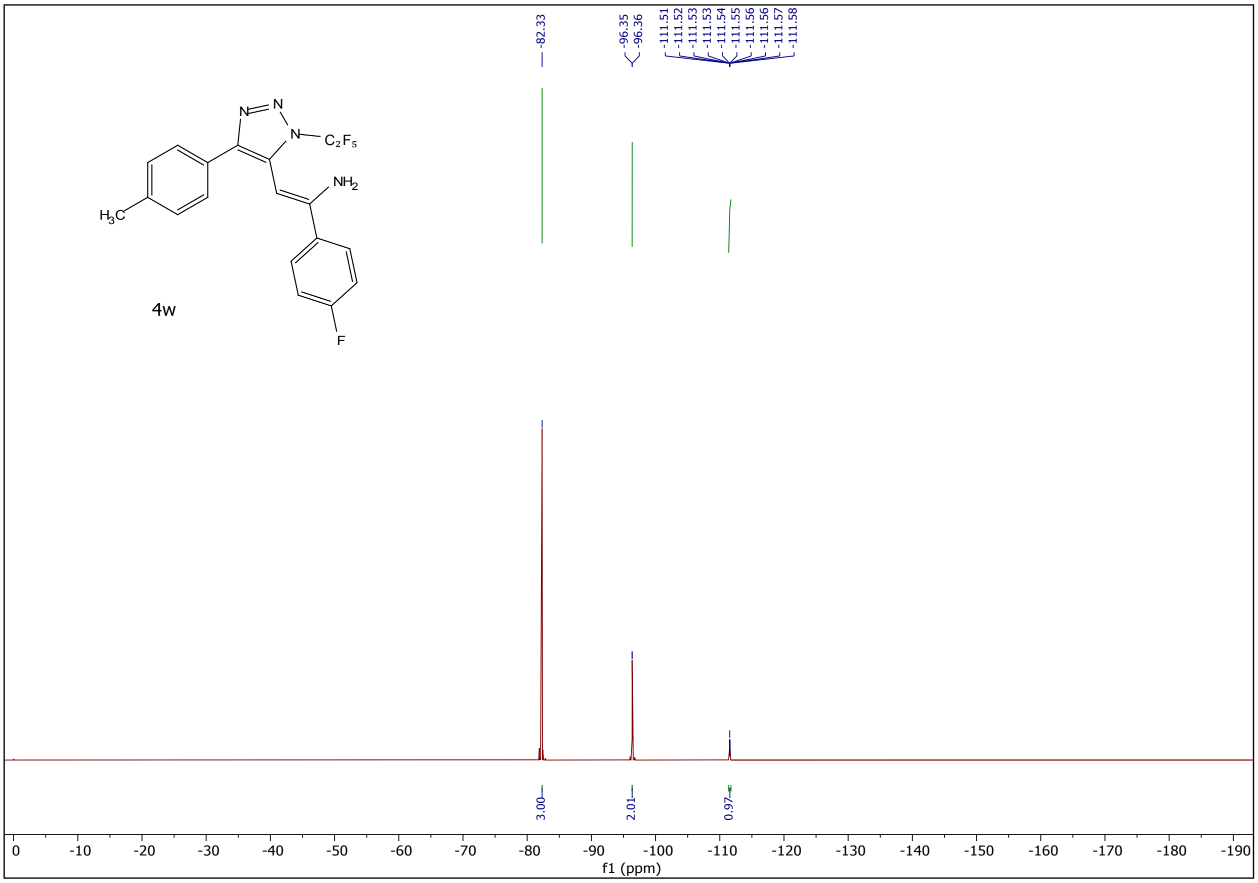

Figure 99. ^{1}H NMR spectrum of **6a** ($CDCl_3$, 400 MHz)

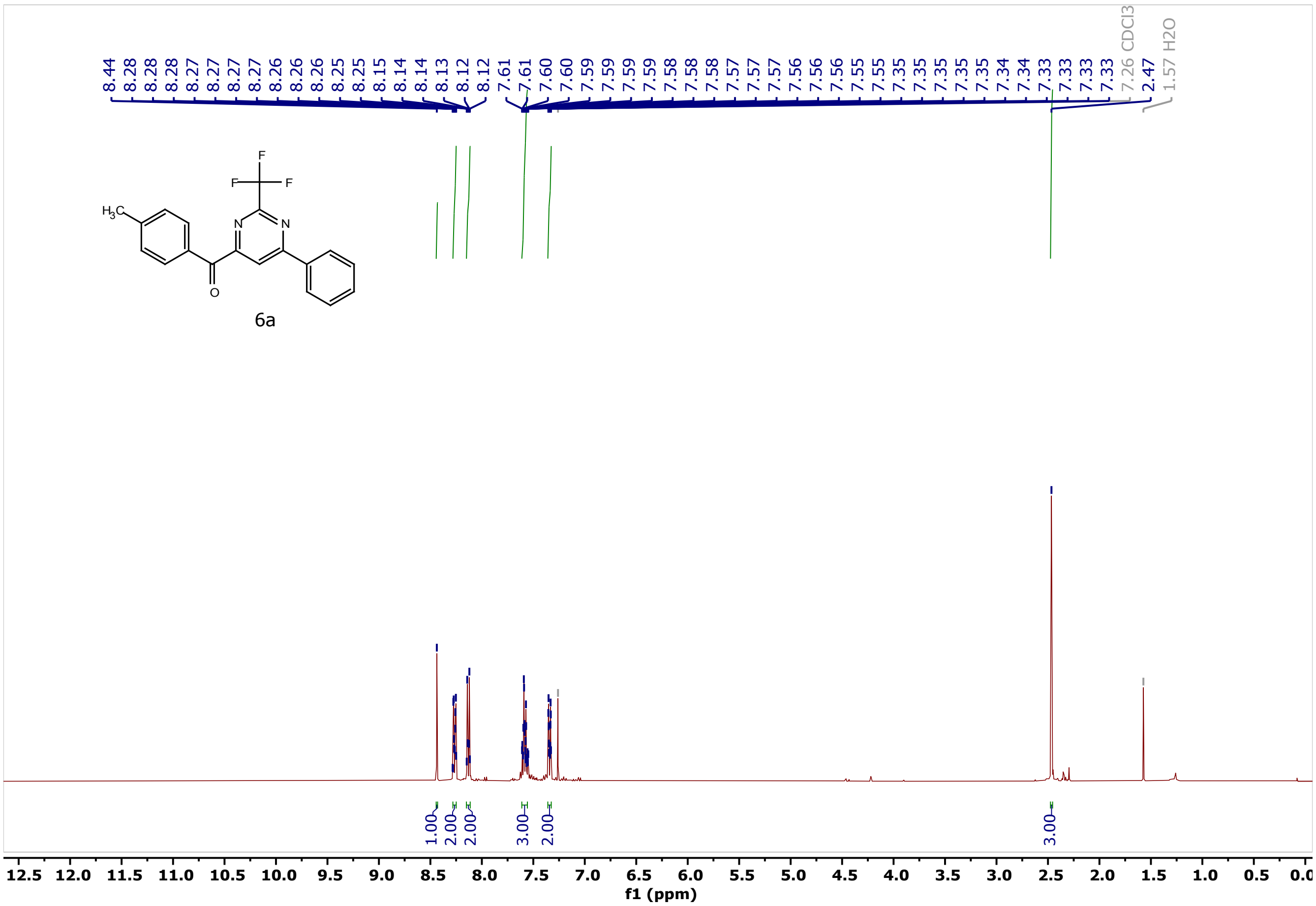

Figure 100. ^{13}C NMR spectrum of **6a** ($CDCl_3$, 101 MHz)

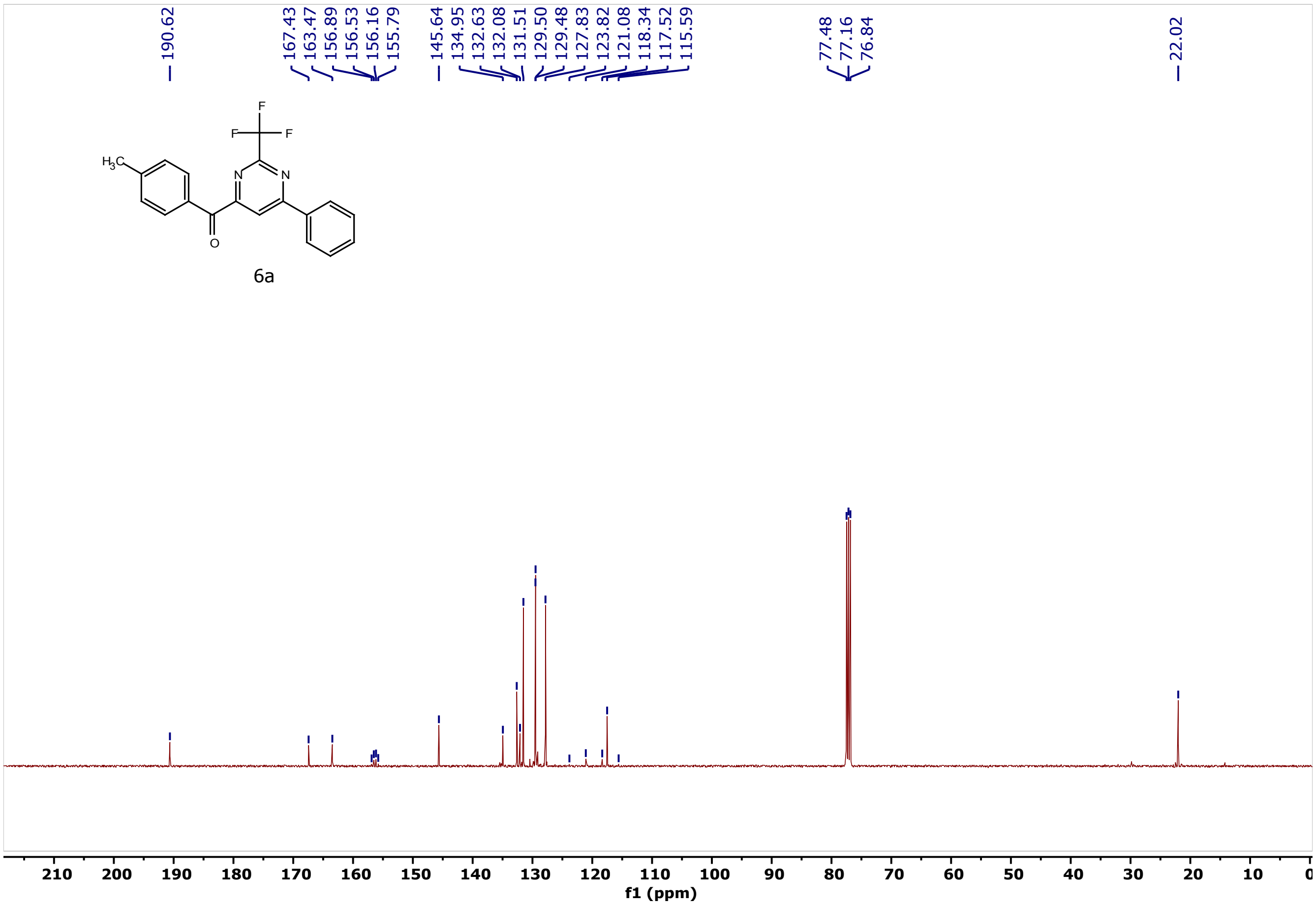

Figure 101. ^{19}F NMR spectrum of **6a** ($CDCl_3$, 377 MHz)

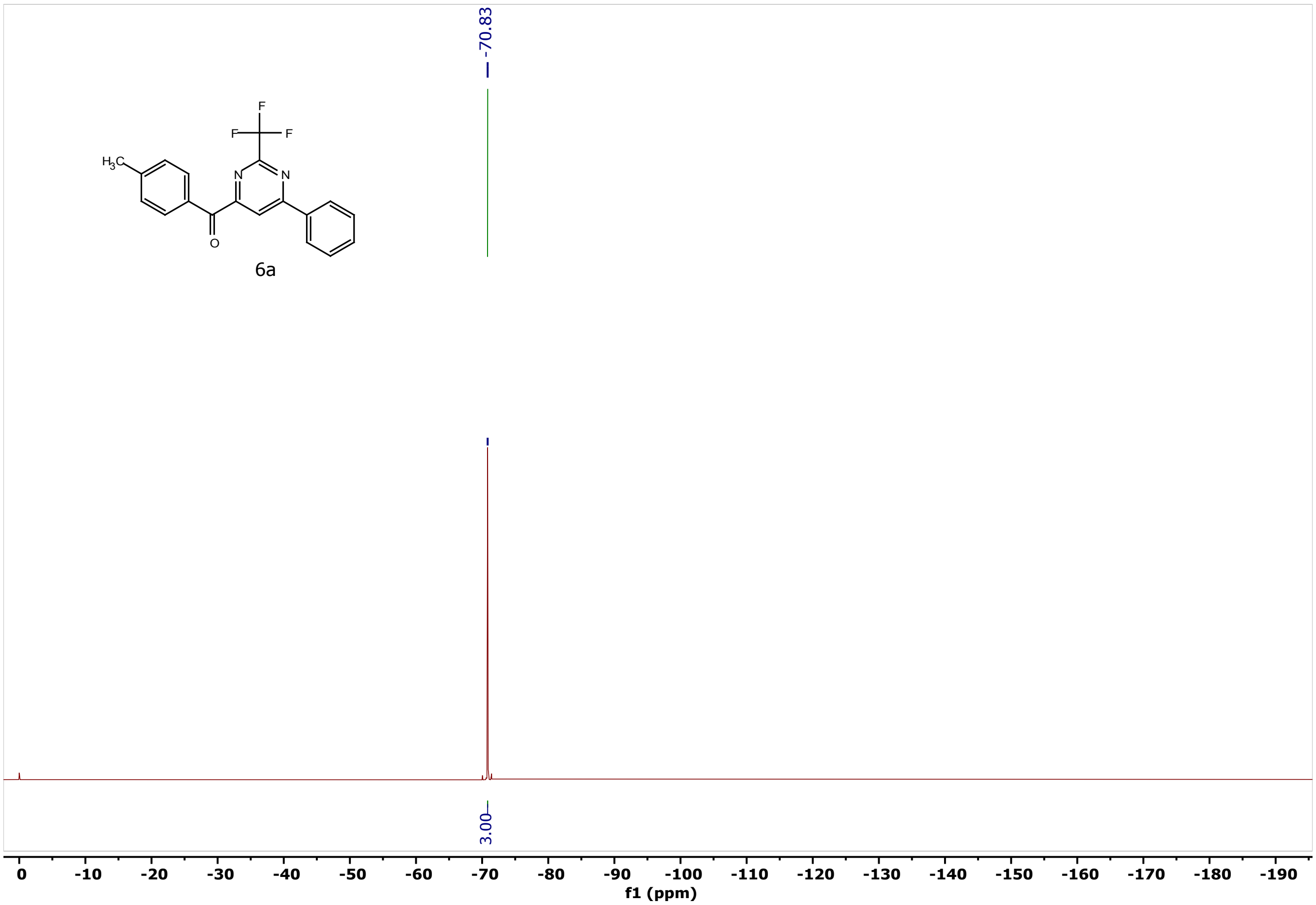

Figure 102. ^{1}H NMR spectrum of **7m** ($CDCl_3$, 400 MHz)

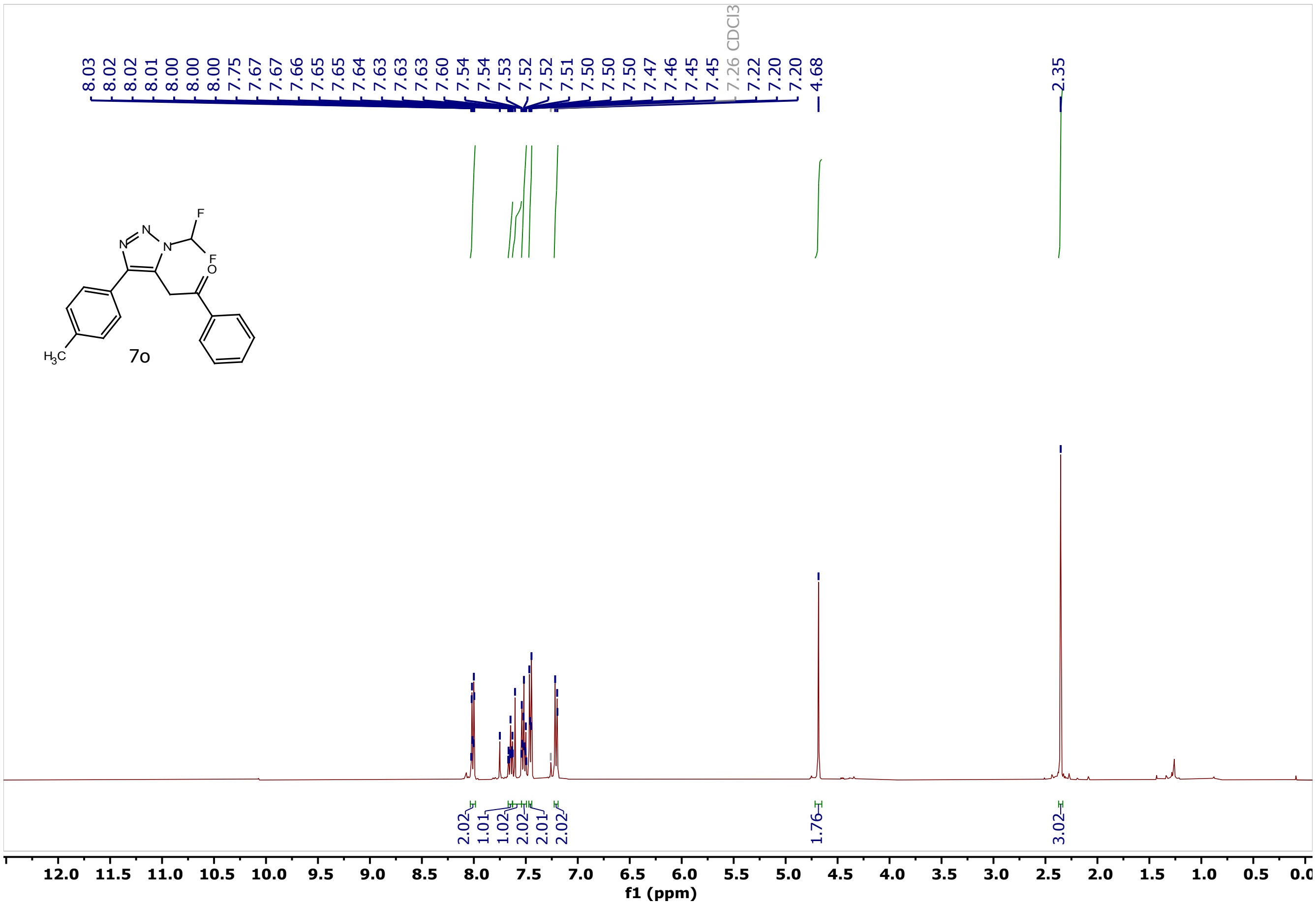

Figure 103. ^{13}C NMR spectrum of **7m** ($CDCl_3$, 101 MHz)

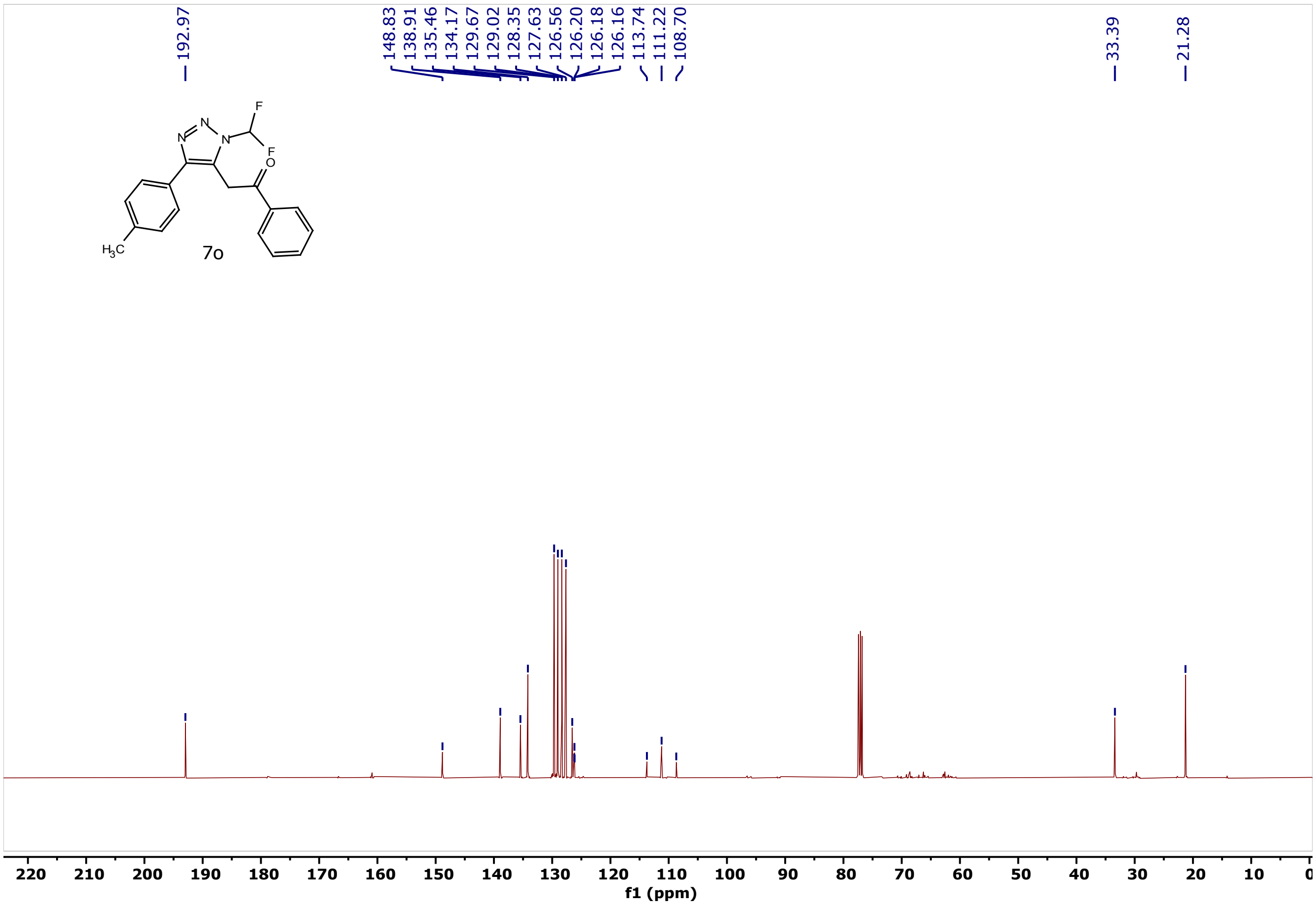

Figure 104. ^{19}F NMR spectrum of **7m** ($CDCl_3$, 377 MHz)

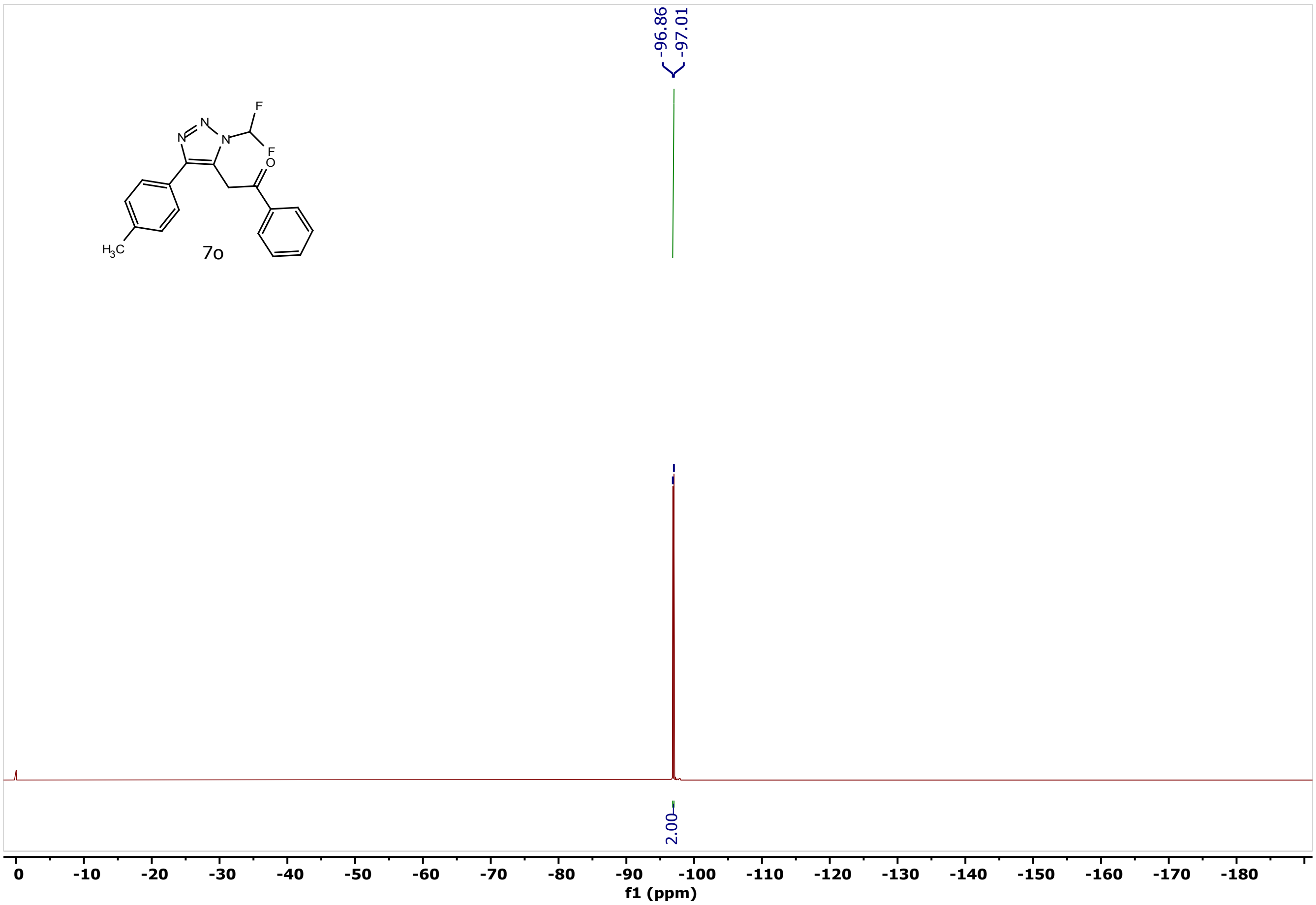

Figure 105. ^{1}H NMR spectrum of **7u** ($CDCl_3$, 400 MHz)

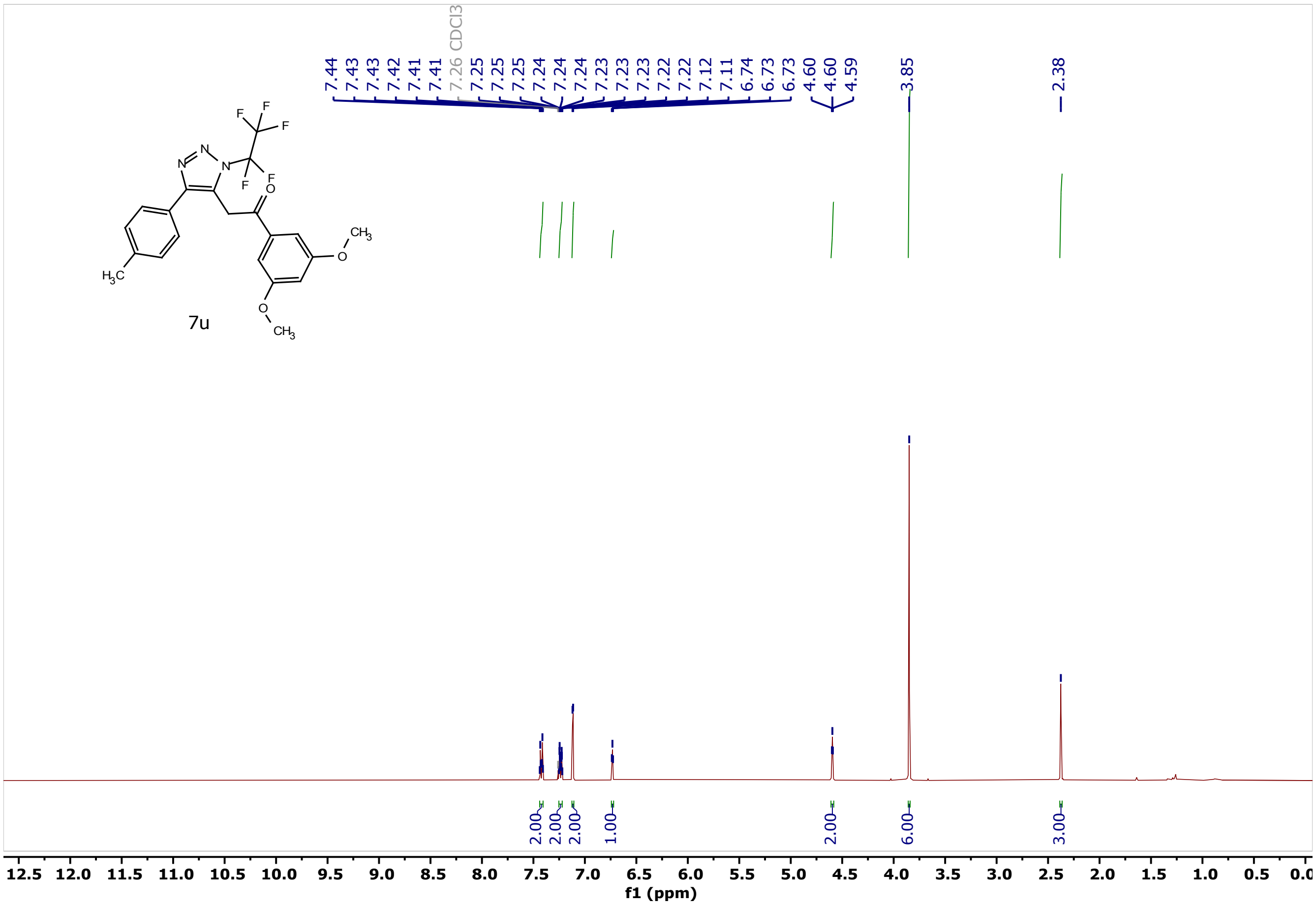

Figure 106. ^{13}C NMR spectrum of **7u** ($CDCl_3$, 101 MHz)

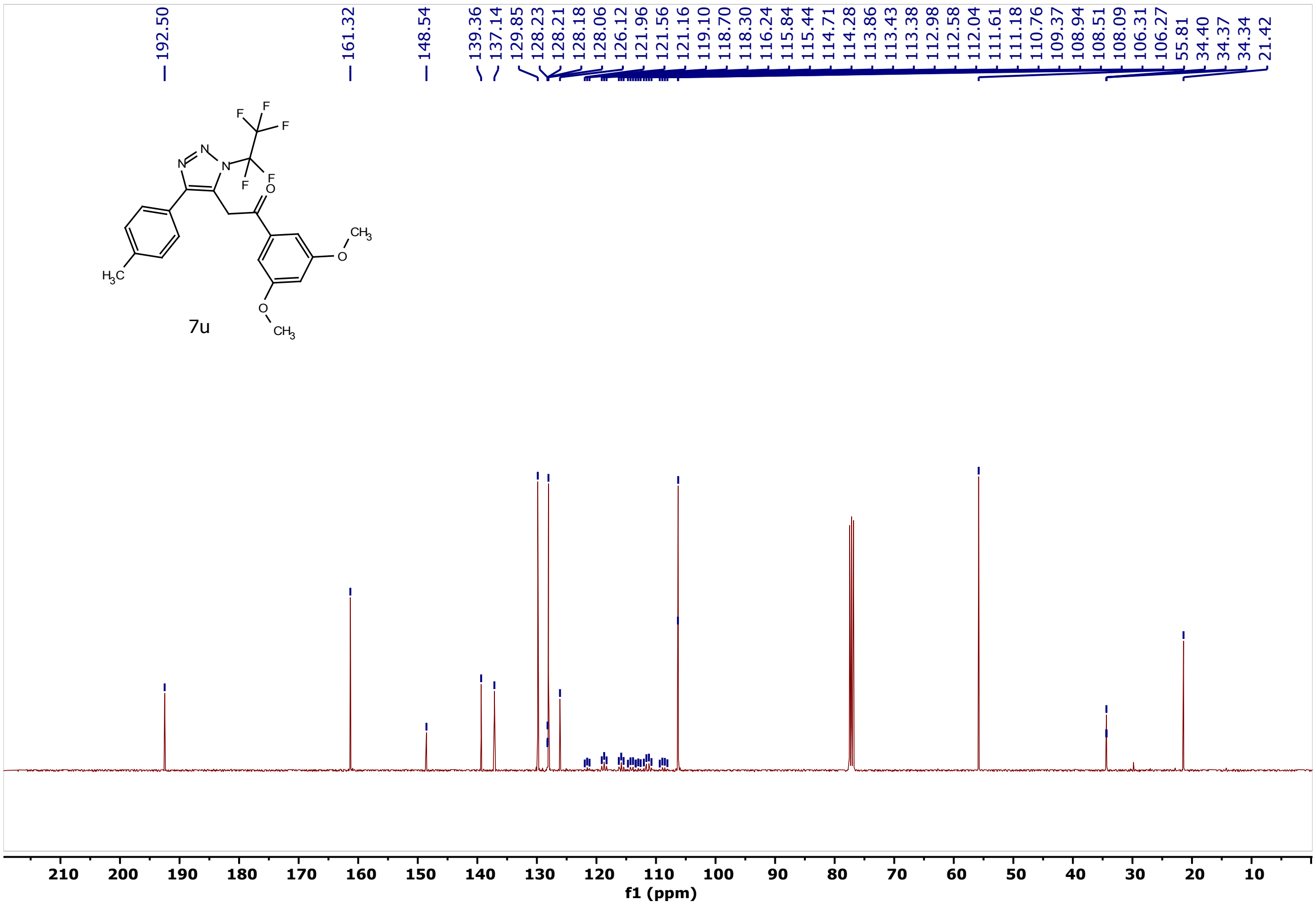

Figure 107. ^{19}F NMR spectrum of **7u** ($CDCl_3$, 377 MHz)

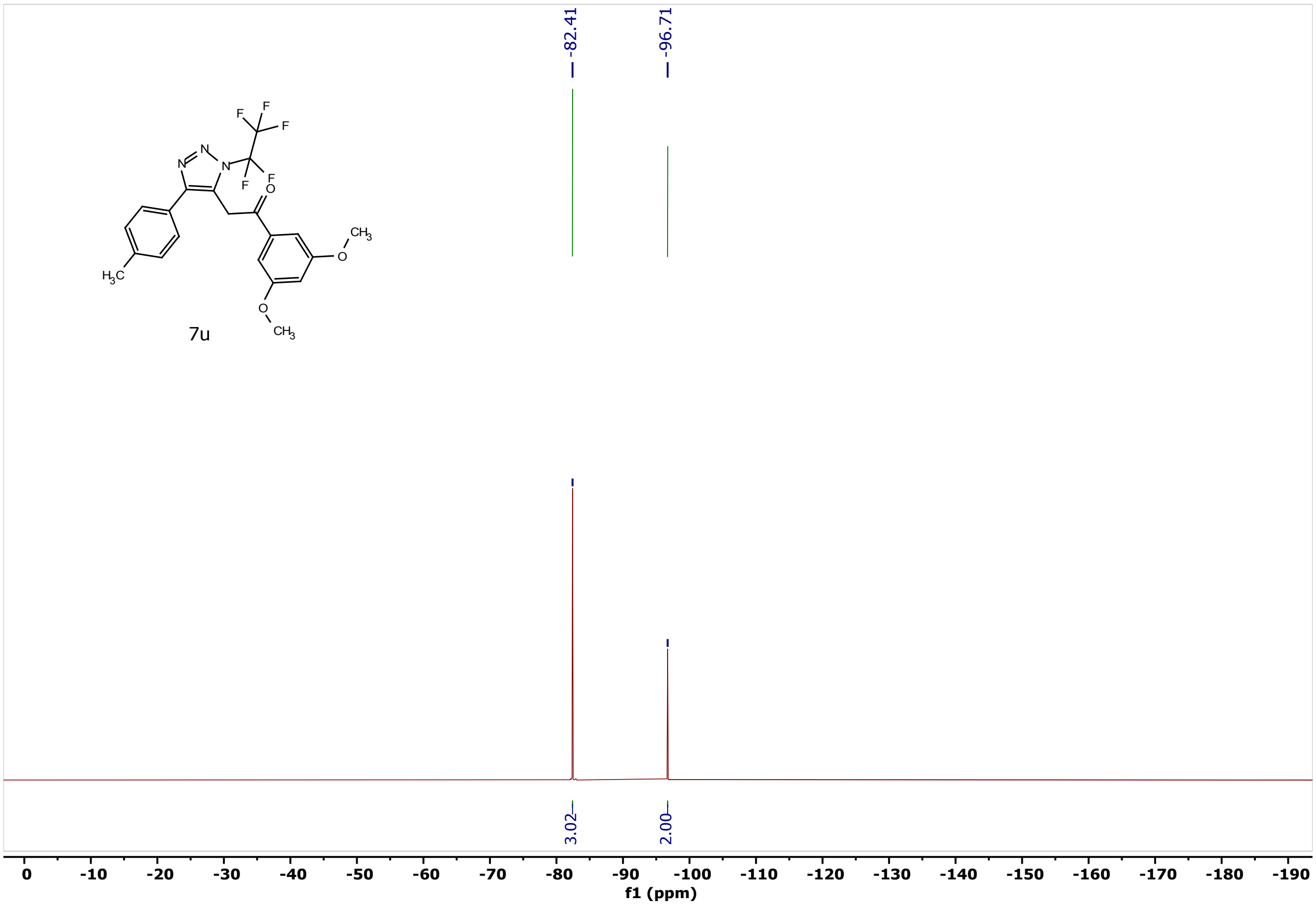

Figure 108. ^{1}H NMR spectrum of **8** ($CDCl_3$, 400 MHz)

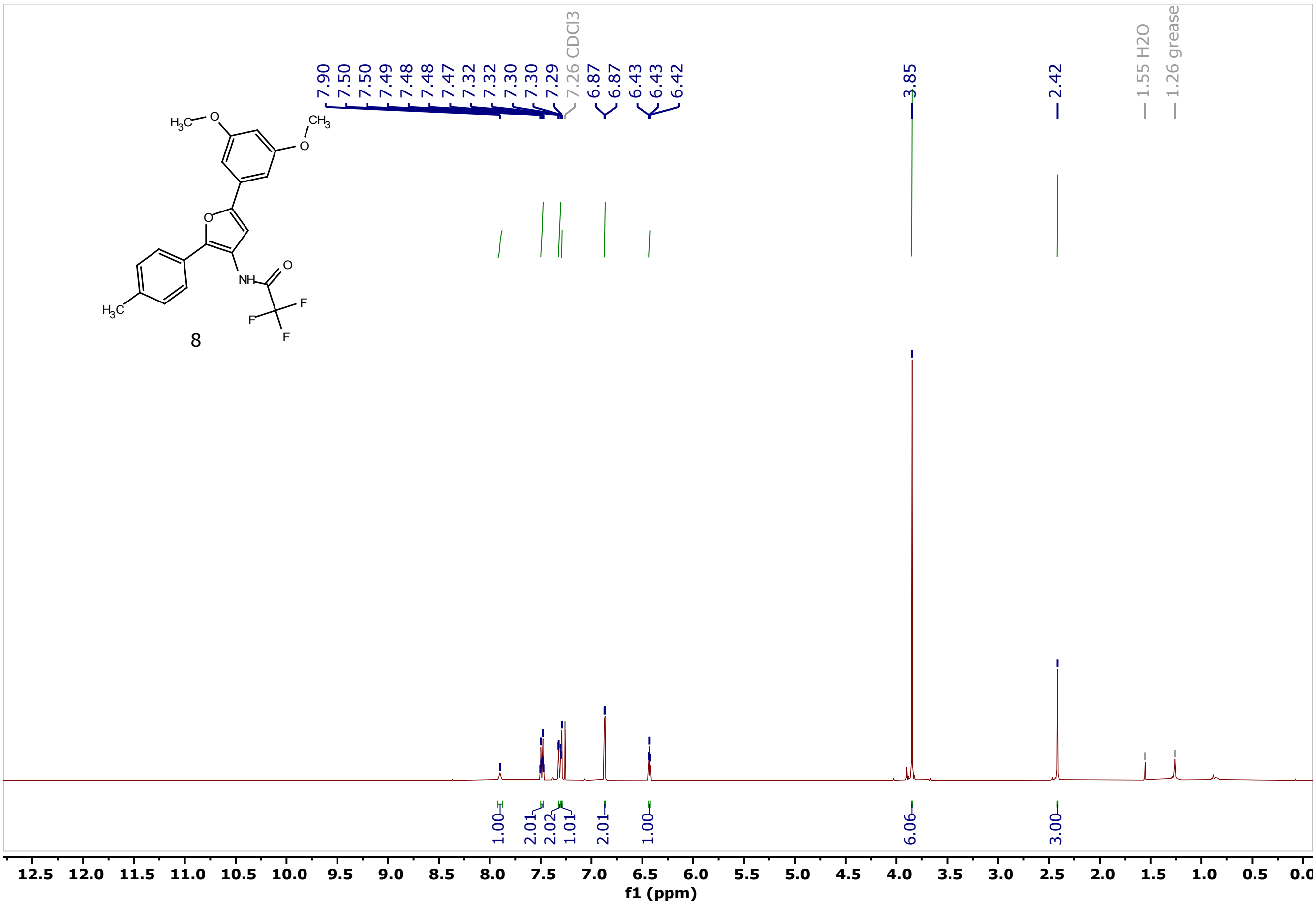

Figure 109. ^{13}C NMR spectrum of **8** ($CDCl_3$, 101 MHz)

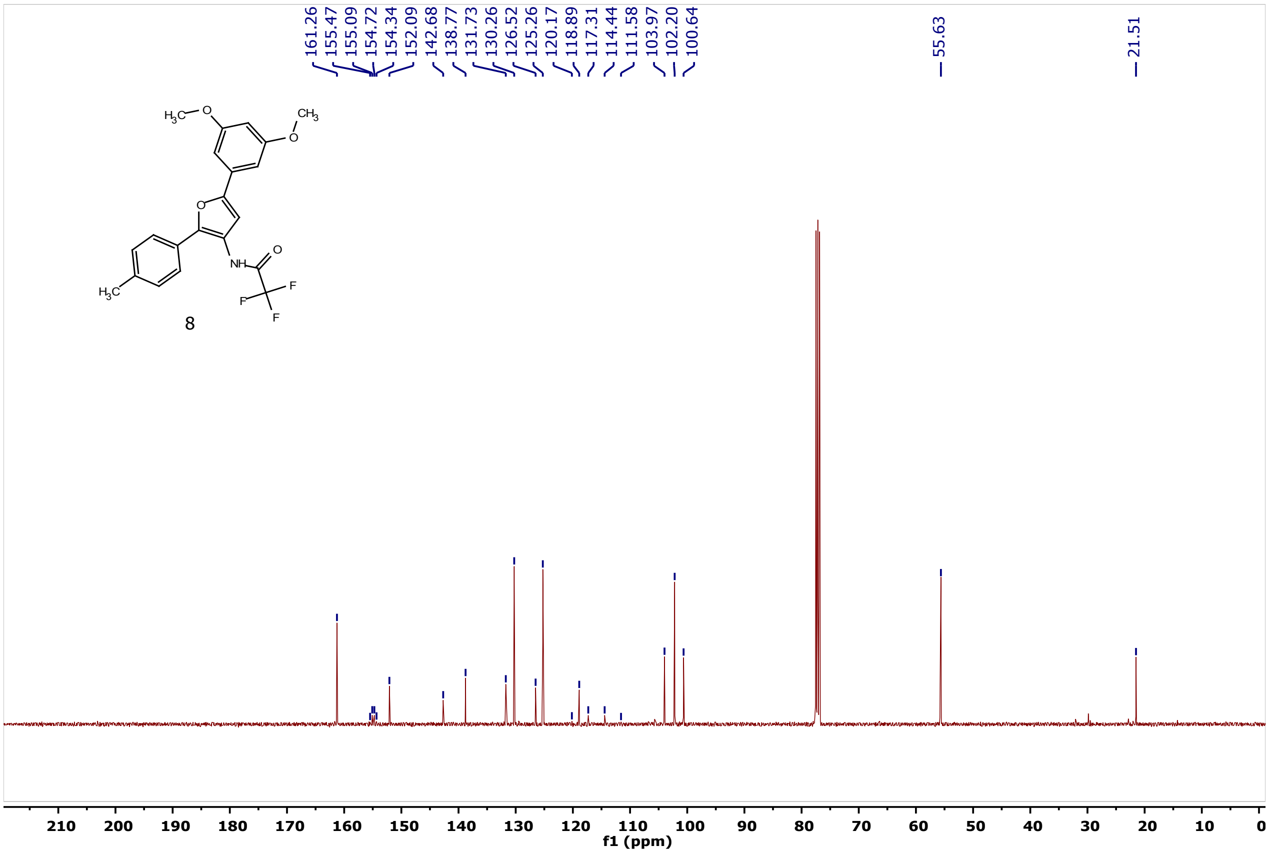

Figure 110. ^{19}F NMR spectrum of **8** ($CDCl_3$, 377 MHz)

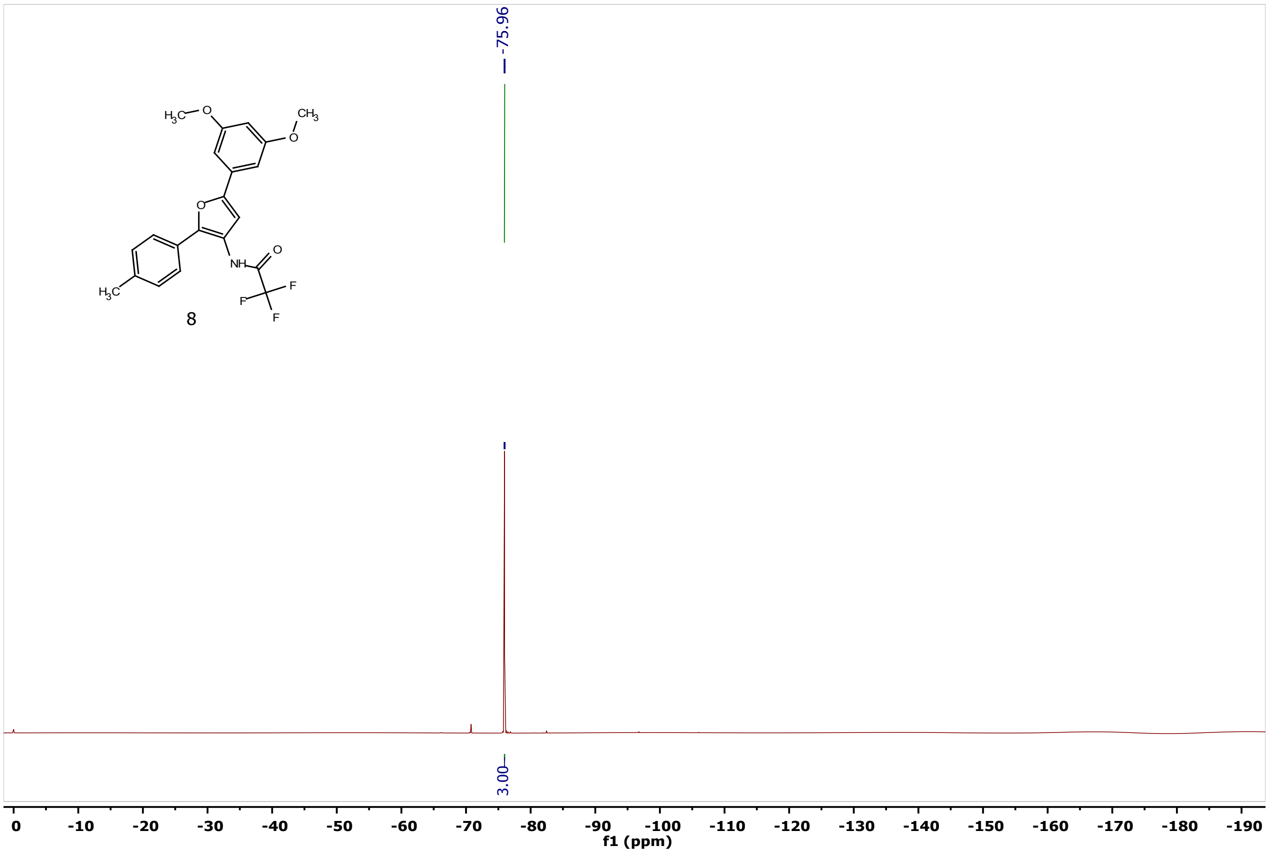

Figure 111. ^{1}H NMR spectrum of **9** ($CDCl_3$, 400 MHz)

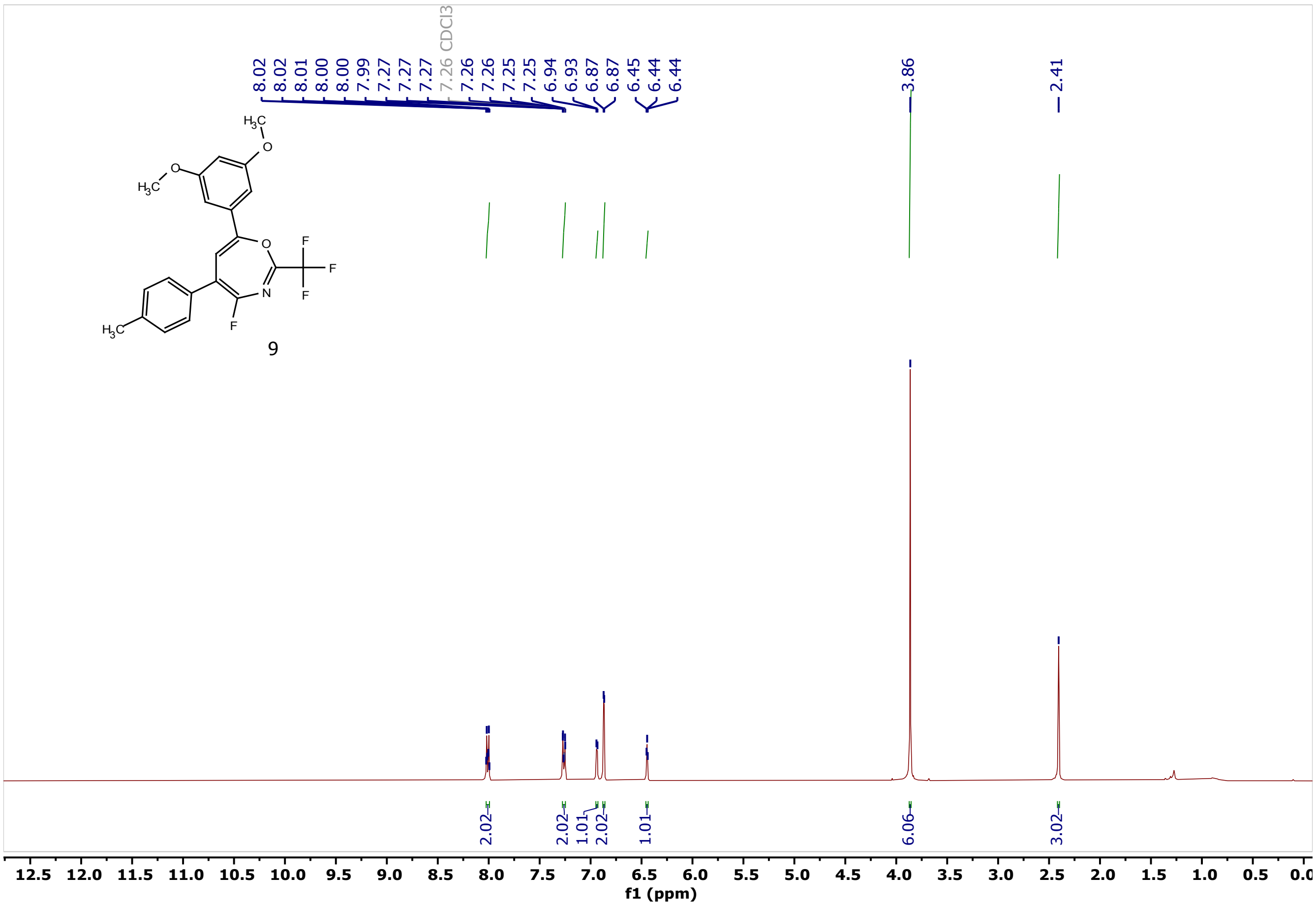

Figure 112. ^{13}C NMR spectrum of **9** ($CDCl_3$, 101 MHz)

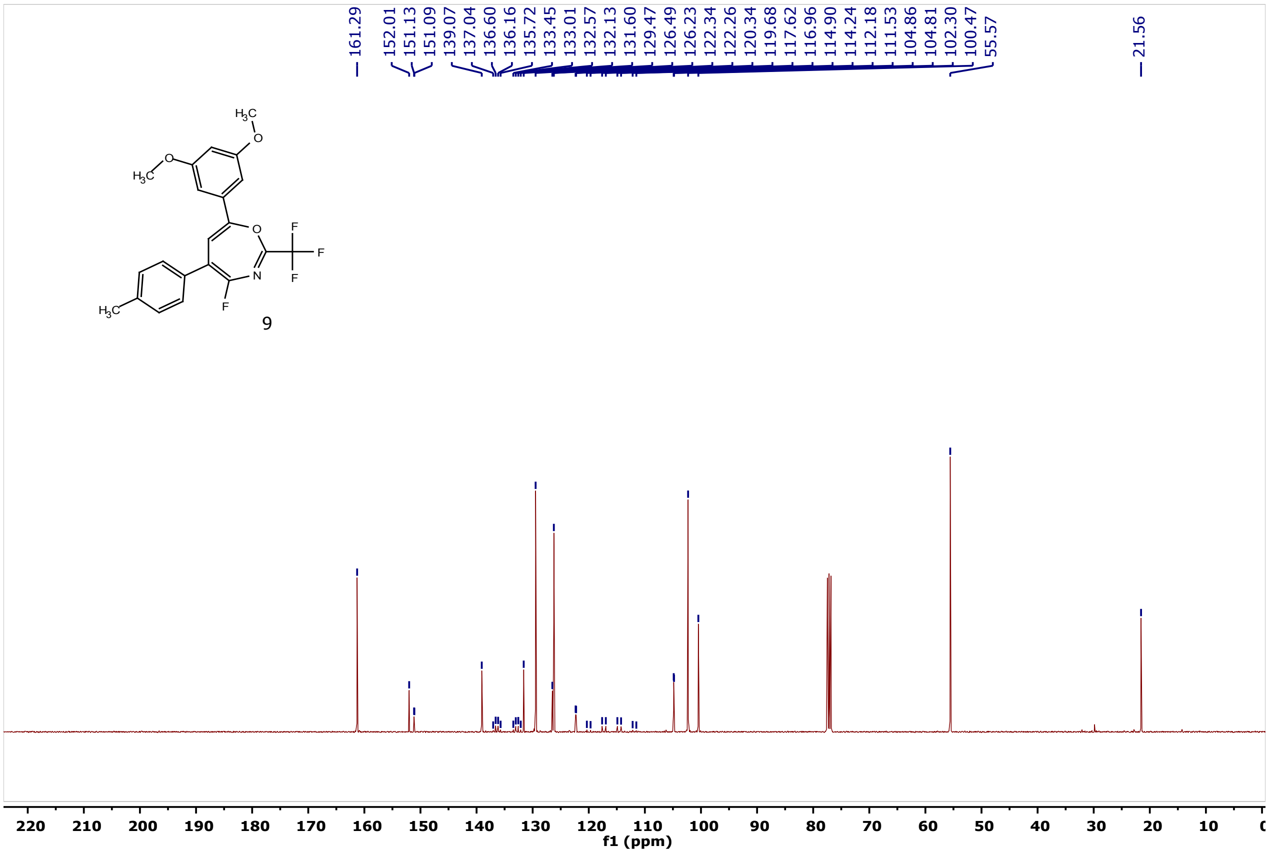

Figure 113. ^{19}F NMR spectrum of **9** ($CDCl_3$, 377 MHz)

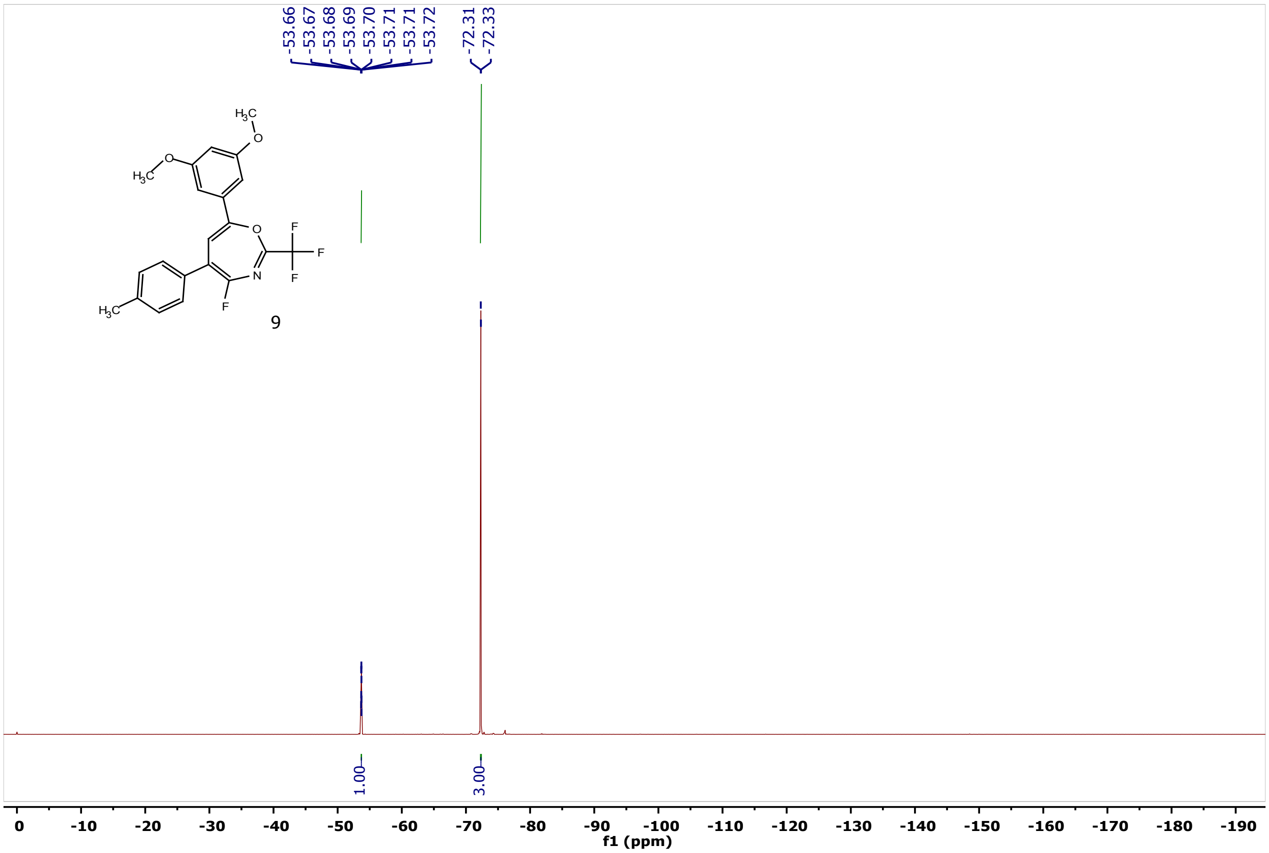

References

(1) Balasubramani, S. G.; Chen, G. P.; Coriani, S.; Diedenhofen, M.; Frank, M. S.; Franzke, Y. J.; Furche, F.; Grotjahn, R.; Harding, M. E.; Hättig, C.; Hellweg, A.; Helmich-Paris, B.; Holzer, C.; Huniar, U.; Kaupp, M.; Marefat Khah, A.; Karbalaei Khani, S.; Müller, T.; Mack, F.; Nguyen, B. D.; Parker, S. M.; Perlt, E.; Rappoport, D.; Reiter, K.; Roy, S.; Rückert, M.; Schmitz, G.; Sierka, M.; Tapavicza, E.; Tew, D. P.; Van Wüllen, C.; Voora, V. K.; Weigend, F.; Wodyński, A.; Yu, J. M. TURBOMOLE: Modular Program Suite for Ab Initio Quantum-Chemical and Condensed-Matter Simulations. *J. Chem. Phys.* **2020**, *152*.

(2) Tao, J.; Perdew, J. P.; Staroverov, V. N.; Scuseria, G. E. Climbing the Density Functional Ladder: Nonempirical Meta–Generalized Gradient Approximation Designed for Molecules and Solids. *Phys. Rev. Lett.* **2003**, *91*, 3–6.

(3) Staroverov, V. N.; Scuseria, G. E.; Tao, J.; Perdew, J. P. Comparative Assessment of a New Nonempirical Density Functional: Molecules and Hydrogen-Bonded Complexes. *J. Chem. Phys.* **2003**, *119*, 12129–12137.

(4) Weigend, F.; Ahlrichs, R. Balanced Basis Sets of Split Valence, Triple Zeta Valence and Quadruple Zeta Valence Quality for H to Rn: Design and Assessment of Accuracy. *Phys. Chem. Chem. Phys.* **2005**, *7*, 3297–3305.

(5) Grimme, S. Density Functional Theory with London Dispersion Corrections. *Wiley Interdiscip. Rev. Comput. Mol. Sci.* **2011**, *1*, 211–228.

(6) Grimme, S.; Antony, J.; Ehrlich, S.; Krieg, H. A Consistent and Accurate Ab Initio Parametrization of Density Functional Dispersion Correction (DFT-D) for the 94 Elements H-Pu. *J. Chem. Phys.* **2010**, *132*.

(7) Grimme, S. Accurate Description of van Der Waals Complexes by Density Functional Theory Including Empirical Corrections. *J. Comput. Chem.* **2004**, *25*, 1463–1473.

(8) Becke, A. D.; Johnson, E. R. A Density-Functional Model of the Dispersion Interaction. *J. Chem. Phys.* **2005**, *123*.

(9) Johnson, E. R.; Becke, A. D. A Post-Hartree-Fock Model of Intermolecular Interactions. *J. Chem. Phys.* **2005**, *123*.

(10) Klamt, A.; Schüürmann, G. COSMO: A New Approach to Dielectric Screening in Solvents with Explicit Expressions for the Screening Energy and Its Gradient. *J. Chem. Soc. Perkin Trans. 2* **1993**, *5*, 799–805.

(11) Bannwarth, C.; Ehlert, S.; Grimme, S. GFN2-XTB - An Accurate and Broadly Parametrized Self-Consistent Tight-Binding Quantum Chemical Method with Multipole Electrostatics and Density-Dependent Dispersion Contributions. *J. Chem. Theory Comput.* **2019**, *15*, 1652–1671.

(12) Pracht, P.; Bohle, F.; Grimme, S. Automated Exploration of the Low-Energy Chemical Space with Fast Quantum Chemical Methods. *Phys. Chem. Chem. Phys.* **2020**, *22*, 7169–7192.

(13) Lecklider, T. Maintainng a Heathy Rhythm. *EE Eval. Eng.* **2011**, *50*, 36–39.

(14) Becke, A. D. Density-functional Thermochemistry. III. The Role of Exact Exchange. *J. Chem. Phys* **1993**, *1998*, 5648–5652.

(15) Zhang, S.; Çelebi-Ölçüm, N.; Melzer, M. M.; Houk, K. N.; Warren, T. H. Copper(I) Nitrosyls from Reaction of Copper(II) Thiolates with S-Nitrosothiols: Mechanism of NO Release from RSNOs at Cu. *J. Am. Chem. Soc.* **2013**, *135*, 16746–16749.

(16) Debnath, S.; Laxmi, S.; McCubbin Stepanic, O.; Quek, S. Y.; van Gastel, M.; DeBeer, S.; Krämer, T.; England, J. A Four-Coordinate End-On Superoxocopper(II) Complex: Probing the Link between Coordination Number and Reactivity. *J. Am. Chem. Soc.* **2024**, *146*, 23704–23716.

(17) Ahlquist, M.; Fokin, V. V. Enhanced Reactivity of Dinuclear Copper(I) Acetylides in Dipolar Cycloadditions. *Organometallics* **2007**, *26*, 4389–4391.

(18) Himo, F.; Lovell, T.; Hilgraf, R.; Rostovtsev, V. V.; Noodleman, L.; Sharpless, K. B.; Fokin, V. V. Copper(I)-Catalyzed Synthesis of Azoles. DFT Study Predicts Unprecedented Reactivity and Intermediates. *J. Am. Chem. Soc.* **2005**, *127*, 210–216.

(19) Burow, A. M.; Sierka, M.; Mohamed, F. Resolution of Identity Approximation for the Coulomb Term in Molecular and Periodic Systems. *J. Chem. Phys.* **2009**, *131*.

(20) Grimme, S. Supramolecular Binding Thermodynamics by Dispersion-Corrected Density Functional Theory. *Chem. Eur. J.* **2012**, *18*, 9955–9964.

(21) Bannwarth, C.; Caldeweyher, E.; Ehlert, S.; Hansen, A.; Pracht, P.; Seibert, J.; Spicher, S.; Grimme, S. Extended Tight-Binding Quantum Chemistry Methods. *Wiley Interdiscip. Rev. Comput. Mol. Sci.* **2021**, *11*, 1–49.

(22) Ho, J.; Klamt, A.; Coote, M. L. Comment on the Correct Use of Continuum Solvent Models. *J. Phys. Chem. A* **2010**, *114*, 13442–13444.

(23) B. T. Worrell, J. A. Malik, V. V. F. Direct Evidence of a Dinuclear Copper Intermediate in Cu(I)-Catalyzed Azide-Alkyne Cycloadditions. *Science* **2010**, *340*, 457–461.

(24) Ahlquist, M.; Fokin, V. V. Enhanced Reactivity of Dinuclear Copper(I) Acetylides in Dipolar Cycloadditions. *Organometallics* **2007**, *26*, 4389–4391.

(25) Straub, B. F. *μ*-Acetylide and *μ*-Alkenylidene Ligands in “Click” Triazole Syntheses. *Chem. Commun.* **2007**, *37*, 3868–3870.

(26) SAINT. Bruker AXS Inc., Madison, Wisconsin, USA, 2015.

(27) Altomare, A.; Cascarano, G.; Giacovazzo G.; Guagliardi A.; Burla M. C.; Polidori, G.; Camalli, M. *J. Appl. Cryst.* **1994,** *27*, 435.

(28) Betteridge, P. W.; Carruthers, J. R.; Cooper, R. I.; Prout, K., Watkin, D. J. *J. Appl. Cryst.* **2003**, *36*, 1487.

(29) Farrugia, L. J. *J. Appl. Cryst.* **2012**, *45*, 849–854.

(30) Duan, X.; Zheng, N.; Liu, G.; Li, M.; Wu, Q.; Sun, X.; Song, W. *Org. Lett.* **2022**, *24*, 6006–6012.

(31) Zhou, W.; Zhang, M.; Li, H.; Chen, W. *Org. Lett.* **2017**, *19*, 10–13.
